# Efficacy of COVID-19 Treatments: A Bayesian Network Meta-Analysis of Randomized Controlled Trials

**DOI:** 10.3389/fpubh.2021.729559

**Published:** 2021-09-28

**Authors:** Chenyang Zhang, Huaqing Jin, Yi Feng Wen, Guosheng Yin

**Affiliations:** ^1^Department of Statistics and Actuarial Science, University of Hong Kong, Hong Kong SAR, China; ^2^Key Laboratory of Shaanxi Province for Craniofacial Precision Medicine Research, College of Stomatology, Xi'an Jiaotong University, Xi'an, China; ^3^Department of Biostatistics, MD Anderson Cancer Center, Houston, TX, United States

**Keywords:** COVID-19, network meta-analysis, mortality, mechanical ventilation, discharge, viral clearance

## Abstract

**Background:** We provided a comprehensive evaluation of efficacy of available treatments for coronavirus disease 2019 (COVID-19).

**Methods:** We searched for candidate COVID-19 studies in WHO COVID-19 Global Research Database up to August 19, 2021. Randomized controlled trials for suspected or confirmed COVID-19 patients published on peer-reviewed journals were included, regardless of demographic characteristics. Outcome measures included mortality, mechanical ventilation, hospital discharge and viral clearance. Bayesian network meta-analysis with fixed effects was conducted to estimate the effect sizes using posterior means and 95% equal-tailed credible intervals (CrIs). Odds ratio (OR) was used as the summary measure for treatment effect. Bayesian hierarchical models were used to estimate effect sizes of treatments grouped by the treatment classifications.

**Results:** We identified 222 eligible studies with a total of 102,950 patients. Compared with the standard of care, imatinib, intravenous immunoglobulin and tocilizumab led to lower risk of death; baricitinib plus remdesivir, colchicine, dexamethasone, recombinant human granulocyte colony stimulating factor and tocilizumab indicated lower occurrence of mechanical ventilation; tofacitinib, sarilumab, remdesivir, tocilizumab and baricitinib plus remdesivir increased the hospital discharge rate; convalescent plasma, ivermectin, ivermectin plus doxycycline, hydroxychloroquine, nitazoxanide and proxalutamide resulted in better viral clearance. From the treatment class level, we found that the use of antineoplastic agents was associated with fewer mortality cases, immunostimulants could reduce the risk of mechanical ventilation and immunosuppressants led to higher discharge rates.

**Conclusions:** This network meta-analysis identified superiority of several COVID-19 treatments over the standard of care in terms of mortality, mechanical ventilation, hospital discharge and viral clearance. Tocilizumab showed its superiority compared with SOC on preventing severe outcomes such as death and mechanical ventilation as well as increasing the discharge rate, which might be an appropriate treatment for patients with severe or mild/moderate illness. We also found the clinical efficacy of antineoplastic agents, immunostimulants and immunosuppressants with respect to the endpoints of mortality, mechanical ventilation and discharge, which provides valuable information for the discovery of potential COVID-19 treatments.

## Introduction

The pandemic of novel coronavirus disease 2019 (COVID-19) has become a global threat to public health. By August 27, 2021, over 214 million confirmed cases including 4.47 million deaths have been reported (1). Faced with such a global crisis, identifying effective treatments for COVID-19 is of urgent need and paramount importance for clinical researchers. Development of novel drugs typically takes years of concerted efforts and thus most of the research in COVID-19 treatment has been focused on drug repositioning, i.e., investigating the effectiveness of drugs approved for other diseases on COVID-19 patients. By August 18, 2021, over 11,000 clinical trials related to COVID-19 have been registered worldwide (2), while only dexamethasone (3, 4) and remdesivir (5, 6) were proven to be clinically effective.

With global efforts on pursuing effective treatments during the pandemic, a large number of short-term randomized controlled trials (RCTs) of small size were conducted and published at a high rate, and some trials were carried out in a rather rush manner which might cause deterioration of trial quality. Timely summaries and analyses of existing clinical trial results can help researchers to better understand various treatments, early terminate investigation on ineffective treatments and provide necessary guidelines for further research and discovery of new treatments. However, the conventional pairwise meta-analysis is limited in simultaneous comparisons among multiple trials and it often fails to capture indirect evidence for treatments that have not been tested in head-to-head comparisons. A network meta-analysis (NMA) which combines both direct and indirect information would be more appropriate to accommodate such a complex situation. Several NMA publications provided useful information on the comparative effectiveness of repurposed drugs for patients with COVID-19 (7, 8).

During the drug repurposing process, clinicians identify candidate drugs by estimating drug-disease or drug-drug similarities. Drugs with shared chemical structures and mechanisms of action are expected to deliver similar therapeutic applications (9). Not only should research focus on individual treatment for COVID-19, but it is also of great interest to evaluate a class of treatments with shared clinical properties and biochemical structures. For example, glucocorticoids including methylprednisolone, dexamethasone and hydrocortisone were reported to be associated with reduced 28-day mortality for critical COVID-19 patients (10).

This NMA aimed to provide a comprehensive evaluation of the efficacy of available treatments for patients with COVID-19. Not only does our NMA evaluate treatments at the drug level, but it also provides an overall estimated effect at the class level which may contain several drugs of similar types via a Bayesian hierarchical model using fixed-effects. Such class levels of treatment evaluation have not been explored in the literature.

## Materials and Methods

This systematic review and NMA were conducted and reported in accordance with the guidelines of Preferred Reporting Items for Systematic Reviews and Meta-Analyses for NMAs (11). A prespecified protocol can be found in Supplementary Materials.

### Information Sources and Eligibility Criteria

We performed an exhaustive online search for eligible studies in the WHO COVID-19 Global Research Database (12). The WHO COVID-19 Global Research Database is a global multilingual literature database which gathers the latest COVID-19 related studies as a composite of other databases (e.g., Medline, Global Health, PubMed Central, PsycInfo, Scopus, ProQuest Central, Embase, Web of Science and others). Supplementary Table S1 presents the detailed searching strategy. We updated the literature search weekly to include newly published trials in peer-reviewed journals. The current version of our manuscript includes studies from January 1, 2020 to August 19, 2021.

Original articles investigating treatment effects for suspected or confirmed COVID-19 were included. We considered appropriate COVID-19 treatments while excluding (i) herbal medicine; (ii) preventive interventions (e.g., vaccination and mask wearing); (iii) non-drug supportive care; (iv) exercise, psychological and educational treatments. We included studies that compared one intervention with other interventions or the standard of care (SOC).

The outcomes of interest in the NMA included overall mortality, requirement for mechanical ventilation (MV), discharge from hospital on day 14 or the day closest to that, and viral clearance on day 7 or the day closest to that. We evaluated only binary outcomes since most COVID-19 trials had <1-month follow-ups (7) and for such short-term studies, continuous or survival outcomes might not provide a clinically meaningful summary for treatment effect (13). In addition, clinical definitions of several continuous outcomes, e.g., time to clinical improvement or deterioration, were not consistent across trials. Different reporting patterns of point and interval estimates for continuous outcomes may also cause additional difficulties and biases in the NMA.

We only included RCTs in this NMA because non-randomized trials and observational studies were considered of low certainty from the causal inference perspective (14). We included trials published in peer-reviewed journals in English and Chinese regardless of ways of randomization (double-blind, single-blind or open-label) or demographic characteristics.

### Study Selection

Two reviewers independently screened the titles and abstracts using the inclusion criteria. Full texts of potentially eligible studies were further assessed for eligibility. Discrepancies were resolved by discussion and, if necessary, a third investigator was consulted.

### Data Collection Process

Data extraction was conducted by two investigators independently. For each eligible study, we collected trial characteristics, interventions, demographic characteristics and outcomes of interest. For binary outcomes of interest, numbers of events and overall numbers of patients were collected. Two reviewers resolved discrepancies via discussion and a third party adjudicated if any conflict arose. For multiple reports on the same trial, we adopted the latest peer-reviewed publication.

### Risk of Bias of Individual Studies

For each eligible trial, we used a revision (7) of version 2 of the Cochrane risk of bias tool (RoB 2.0) (15) to assess risk of bias in RCTs. Detailed RoB judgments were listed in Supplementary Materials. Two reviewers separately completed the RoB assessment and, in presence of any disagreement, a third party made the final decision.

### Data Synthesis and Statistical Analysis

In the network, each node represents a treatment, regardless of the dose or duration of administration. For studies involving different doses or durations of the same drug, we aggregated data of the same drug into one arm. Each multi-arm trial was treated as a single study in the network analysis, instead of being split into multiple two-arm sub-trials. Interventions comprising more than one drug (i.e., combination therapy) were treated as separate treatment nodes. For each clinical outcome, we excluded the treatments appearing in only one trial with fewer than 100 patients to alleviate potential risk caused by inadequate information. We plotted the network for each outcome of interest using the igraph (16) package of R version 4.0.3 (RStudio, Boston, MA).

We considered a hierarchical model structure for investigated interventions where the relative effects compared with SOC were nested within drug classifications. Based on the Anatomical Therapeutic Chemical Classification System with Defined Daily Doses (ATC/DDD) (17) published by WHO, we classified included drugs by the second level of their ATC/DDD codes. For investigational drugs without ATC/DDD codes, we determined their classifications according to the pharmacological mechanism and therapeutic use. The detailed Bayesian hierarchical model structure for the NMA is shown in Supplementary Materials.

We fitted the Bayesian NMA model and generated posterior samples of parameters using the Markov chain Monte Carlo (MCMC) algorithm. The treatment effects of eligible drugs were evaluated in terms of the odds ratio (OR) estimated by the posterior mean and corresponding equal-tailed 95% credible interval (CrI). To obtain direct and indirect estimates for treatment effects and assess local inconsistency in the network, we considered the node-splitting method (18). The MCMC sampling was performed using the jagsUI (19, 20) package, and further network analyses were performed using the gemtc (21) package of R.

### Certainty of the Evidence

The grading of recommendations assessment, development and evaluation (GRADE) approach for NMA (14) was used to rate the certainty of evidence of NMA estimates. Two investigators rated the certainty of each treatment comparison independently and resolved discrepancies by discussions and, if necessary, consulted with a third party. Detailed ratings and rationales for GRADE were provided in Supplementary Materials.

### Subgroup and Sensitivity Analysis

Planned subgroup analyses were conducted for mild/moderate vs. severe/critical COVID-19 patients. In addition to Bayesian fixed-effects NMA, we also performed Bayesian random-effects NMA. Several RCTs which were designed to be multi-arm trials but reported results for different interventions vs. SOC as if they has been compared in separated two-arm studies. In the primary analysis, we treated these RCTs as multi-arm, and the SOC group with the largest number of participants was used if the periods of patient enrolment of specified interventions had overlaps, otherwise we considered a new SOC group which combined the SOC groups of all studies for the same RCT. In addition, we performed a sensitivity analysis by treating these multi-arm RCTs as separated two-arm trials.

## Results

**Figure 1:**
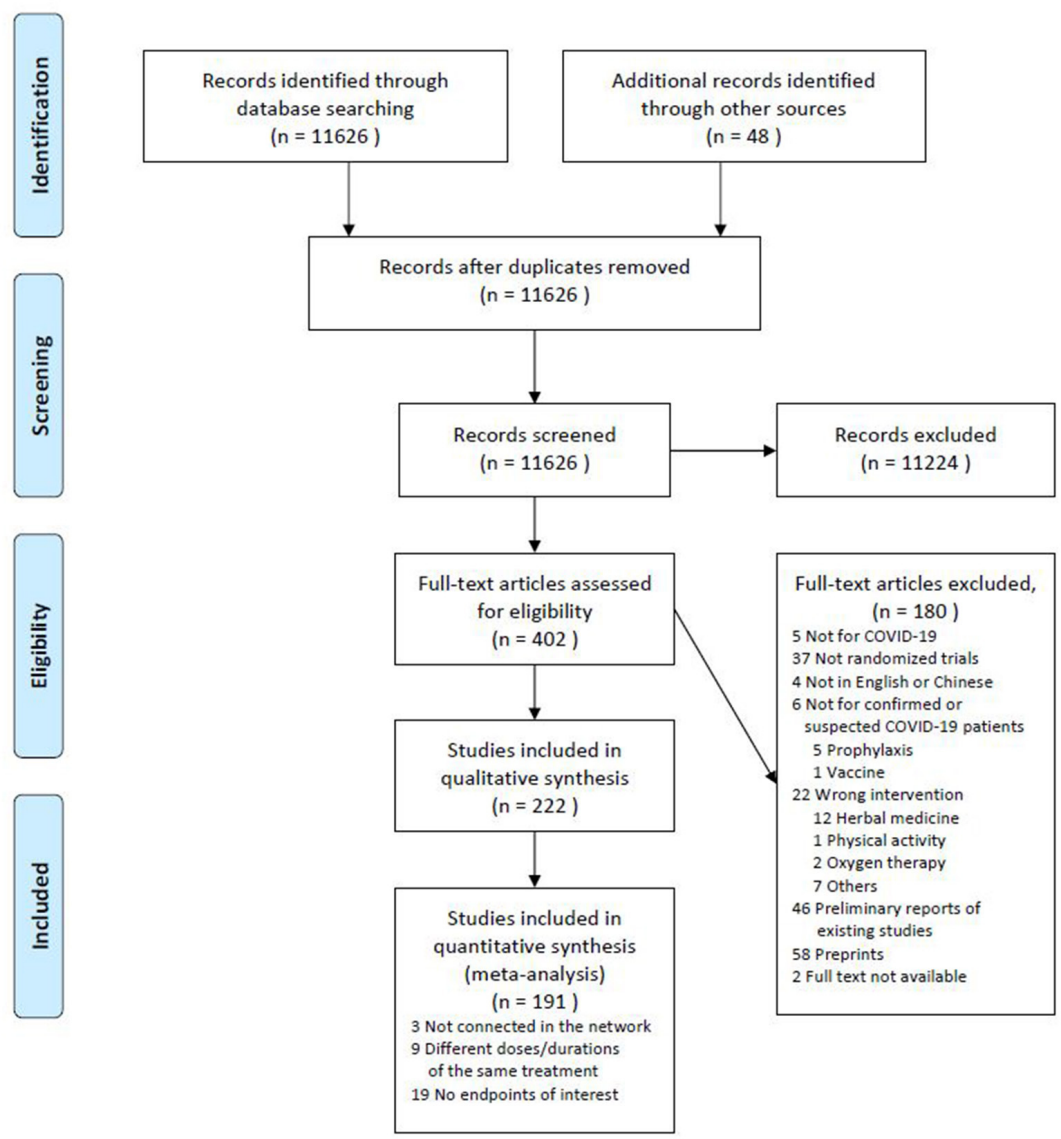
Flow diagram of the study selection.

According to the prespecified inclusion and exclusion criteria, we identified and screened titles and abstracts of 11,626 studies. Among these, 402 studies were further reviewed for full text and 222 eligible studies were included in the systematic review. Figure 1 summarizes the process of our study selection.

Out of these 222 studies, over half (131/222) of them were open-label, 81 were double-blind and the remaining 10 were single-blind in randomization. Most of the studies reported completed clinical trials (187/222) rather than ongoing (7/222) or early terminated trials (28/222), and they mainly investigated hospitalized COVID-19 patients (179/222). In terms of sites, 34 studies were conducted in Iran, 25 in China, 23 in Brazil, 22 in the USA and 23 in multi-sites across countries. Among the 222 studies, 30 were multi-arm and the rest were two-arm; 165 studies compared the investigated intervention with SOC, 35 with other active comparators and the other 22 with both SOC and other interventions. About 40% of the studies (96/222) enrolled <100 patients in the intention-to-treat population and 17 studies recruited over 1,000 patients. Of 181 studies which recorded the baseline severity of illness, 61 involved severe/critical COVID-19 patients while the remaining 120 trials primarily targeted at patients with mild/moderate illness. Detailed trial and patient characteristics are given in Supplementary Materials.

Out of the 222 studies, 31 studies were not considered in the meta-analysis. Among them, nine studies investigated different doses or durations of administration of the same intervention without comparisons with other interventions or SOC, 19 trials did not report outcomes of interest, and treatments in three trials were not connected in the network.

Among the 191 studies included in the quantitative synthesis, 179 unique RCTs were reported, which evaluated the efficacy of 94 different COVID-19 treatments from 41 classes. The RECOVERY trial (NCT04381936) was reported in six studies (3, 22–26). According to an early version of the protocol of the RECOVERY trial (27), the main randomization consisted of two parts: (A) lopinavir/ritonavir, hydroxychloroquine, azithromycin and dexamethasone vs. SOC; (B) convalescent plasma vs. SOC. Patients after the main randomization but with progressive COVID-19 would undergo a second randomization to either tocilizumab or SOC groups. Therefore, in the primary analysis we treated the studies of convalescent plasma (25) and tocilizumab (24) as separated two-arm trials and the four studies of lopinavir/ritonavir (22), hydroxychloroquine (26), azithromycin (23) and dexamethasone (3) vs. SOC as a five-arm trial. The SOC group with the largest number of patients (23) was used. Clinical results of the PRINCIPLE trial (ISRCTN86534580) were shown in three studies (28–30) comparing azithromycin, budesonide and doxycycline with SOC, respectively. Due to no overlap between the enrolment periods of the azithromycin and budesonide studies, we created a new SOC group by combining the two SOC groups and the PRINCIPLE trial was considered as a four-arm trial in the primary analysis. The REMAP-CAP trial (NCT02735707) was reported in three studies which investigated tocilizumab vs. sarilumab vs. SOC (31), hydrocortisone vs. SOC (32) and hydroxychloroquine vs. lopinavir/ritonavir vs. hydroxychloroquine plus lopinavir/ritonavir vs. SOC (33) with patients overlapped in the SOC arms. Thus, we treated it as a seven-arm trial and used the SOC group including the most patients (31). The DISCOVERY trial (34) was a participant of the WHO SOLIDARITY trial (35) while it reported additional endpoints of interest. The observed outcomes of the SOLIDARITY trial (35) were used in the NMA if existed, otherwise we considered those in the DISCOVERY trial (34). The phases II and III of the BLAZE-1 trial (NCT04427501) (36, 37) were reported in two separated articles and we simply merged results of the two stages as one RCT in the primary analysis. The REMAP-CAP, ACTIV-4a, and ATTACC Investigators examined the clinical effect of therapeutic-dose anticoagulation for patients with COVID-19 and presented their results for critically ill and non-critical patients in two publications, respectively (38, 39). In the primary analysis, we combined results of these two articles and in the subgroup analysis on baseline illness severity, they were treated separately.

### Mortality

**Figure 2:**
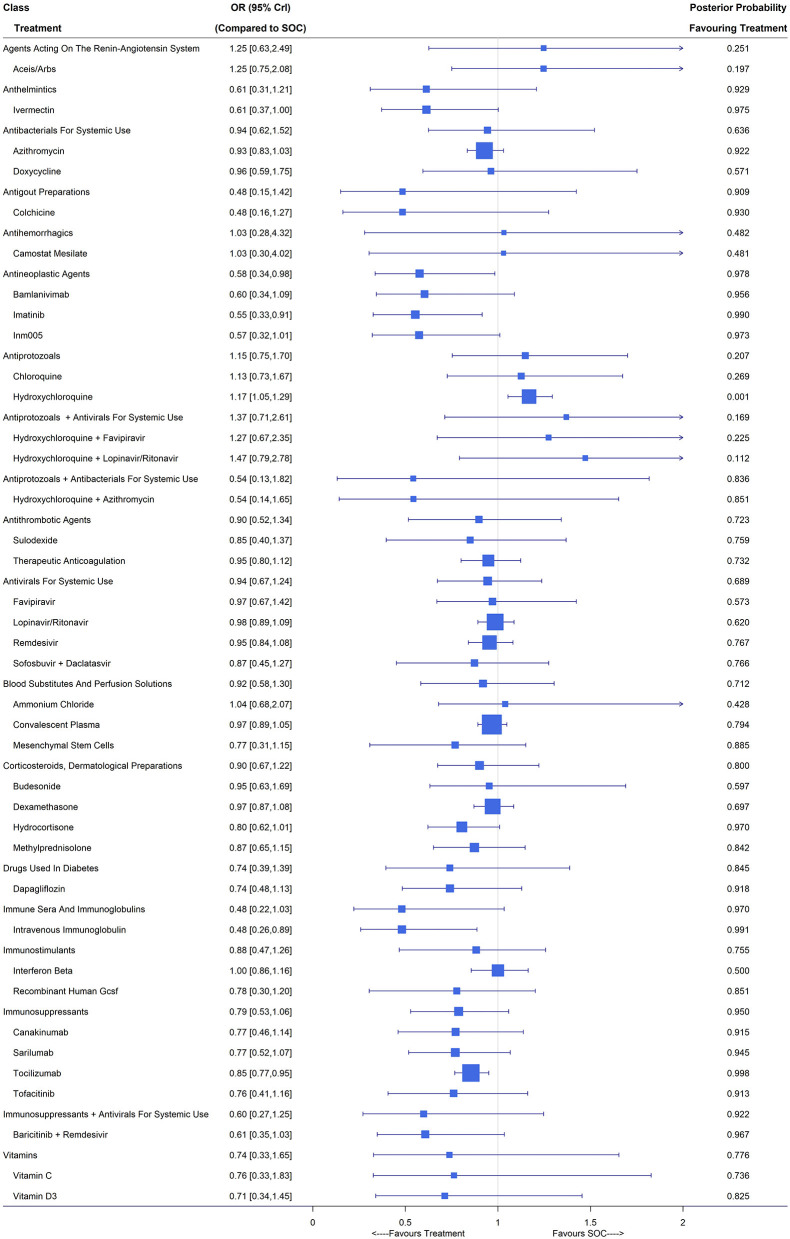
Mortality under treatments compared with the standard of care (SOC); OR is the odds ratio and CrI represents the credible interval.

A total of 179 studies including 96,872 patients reported all-cause mortality. After filtering out treatments with small sample sizes, 132 studies remained in the analysis (3, 6, 22–26, 28–33, 35–153) and the network included angiotensin-converting enzyme inhibitors (ACEIs)/angiotensin receptor blockers (ARBs), ammonium chloride, azithromycin, bamlanivimab, baricitinib plus remdesivir, budesonide, camostat mesilate, canakinumab, chloroquine, colchicine, convalescent plasma, dapagliflozin, dexamethasone, doxycycline, favipiravir, hydrocortisone, hydroxychloroquine, hydroxychloroquine plus azithromycin, hydroxychloroquine plus favipiravir, hydroxychloroquine plus lopinavir/ritonavir, imatinib, INM005, interferon beta, intravenous immunoglobulin, ivermectin, lopinavir/ritonavir, mesenchymal stem cells, methylprednisolone, recombinant human granulocyte colony stimulating factor (GCSF), remdesivir, sarilumab, sofosbuvir plus daclatasvir, sulodexide, therapeutic anticoagulation, tocilizumab, tofacitinib, vitamin C, vitamin D3 and SOC. Among these 132 studies, the risk of bias was accessed to be low for 44 trials and the other 88 were of high risk of bias (Supplementary Table 5). Compared with SOC, the Bayesian NMA with fixed-effects showed that only imatinib (OR 0.55, 95% CrI [0.33, 0.91]; 1 RCT, 197 patients), intravenous immunoglobulin (OR 0.48, 95% CrI [0.26, 0.89]; 5 RCTs, 188 patients) and tocilizumab (OR 0.85, 95% CrI [0.77, 0.95]; 10 RCTs, 3,401 patients) could reduce the mortality rate with statistical significance (Figure 2). Patients treated with hydroxychloroquine even suffered an increased risk of mortality (OR 1.17 [1.05, 1.29]; 24 RCTs, 4,543 patients) compared with those with SOC. The class of antineoplastic agents containing three treatments (bamlanivimab, imatinib and INM005) showed significant clinical benefit over SOC with an OR of 0.58 (95% CrI [0.34, 0.98]; posterior probability of 0.978 favoring treatment). The class of antigout preparations, immunosuppressants plus antivirals for systemic use, anthelmintics, immunosuppressants immune sera and immunoglobulins, might be of potential benefit due to their relatively large posterior probabilities (higher than 0.9) favoring treatment and the other classes showed no difference from SOC.

Under the random-effects model, the estimated treatment effects relative to SOC were similar to those under the fixed-effects model but with wider credible intervals, e.g., tocilizumab with an OR of 0.91 (95% CrI [0.74,1.16]). In the sensitivity analysis by treating part A in the main randomization of RECOVERY (3, 22, 23, 26), PRINCIPLE (28–30), REMAP-CAP (31, 32), BLAZE-1 and two therapeutic-dose anticoagulation trials for critical and noncritical patients (36, 37) as separated trials, all estimates were close to those in the primary analysis except for dexamethasone, which reported an OR of 0.97 (95% CrI [0.87,1.08]) in the primary analysis but 0.85 (95% CrI [0.76,0.95]) in the sensitivity analysis. The difference in the 28-day mortality rate between the SOC arm with the largest number of patients (22.4%, 1,162/5,181) and the SOC arm of the dexamethasone study (22) (25.7%, 1,110/4,321) mainly contributed to the discrepancy in the estimates of OR for dexamethasone vs. SOC. Subgroup analyses (see Supplementary Figures S6, S7) demonstrated that for mild/moderate COVID-19 patients, the use of ivermectin (OR 0.38, 95% CrI [0.18,0.76]) couldsignificantly reduce all-cause mortality and for severe/critical cases, imatinib (OR 0.48, 95% CrI [0.24, 0.94]), intravenous immunoglobulin (OR 0.50, 95% CrI [0.27,0.92]) and tocilizumab (OR 0.84, 95% CrI [0.76, 0.94]) performed well.

### Mechanical Ventilation

**Figure 3:**
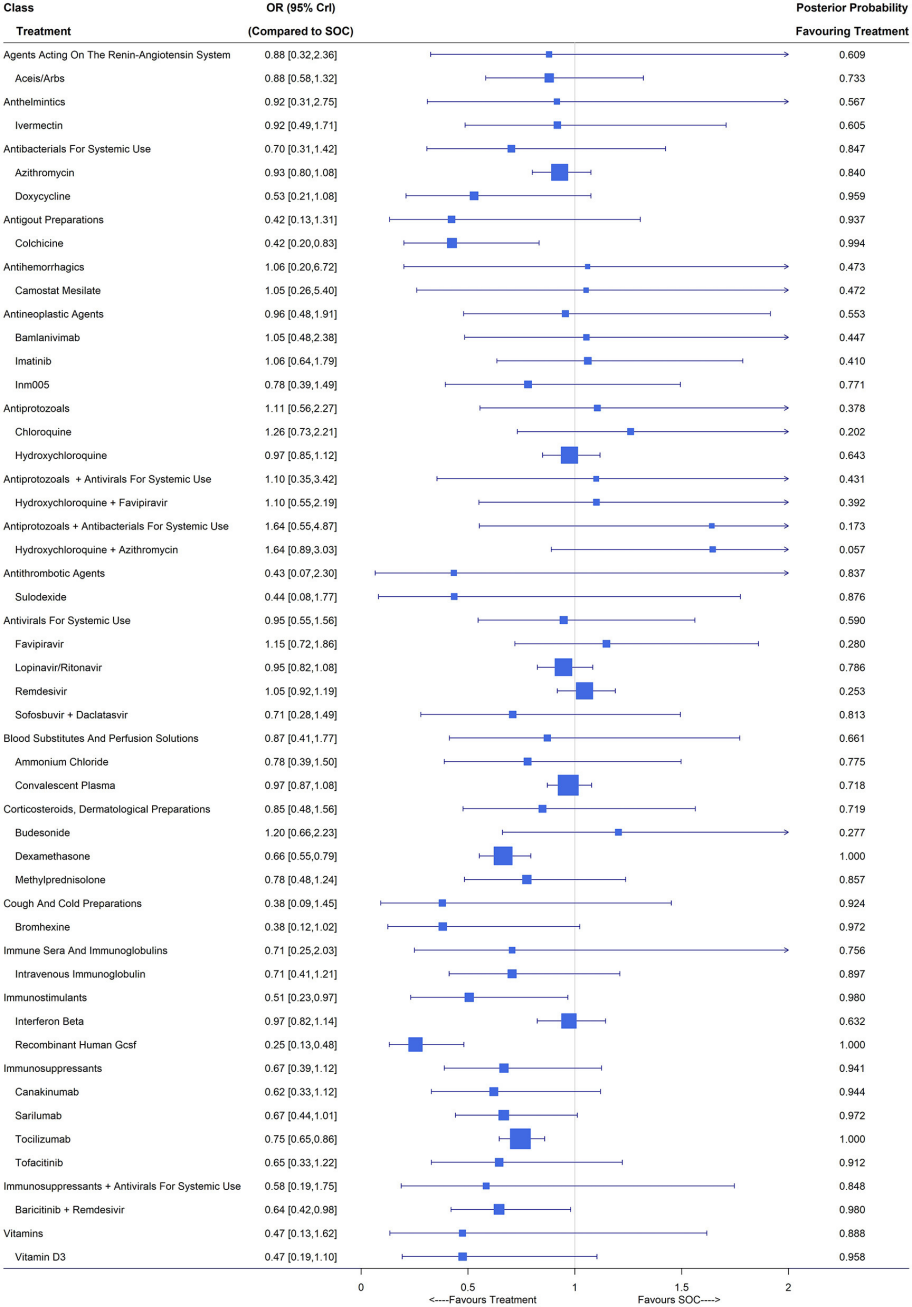
Requirement of mechanical ventilation under treatments compared with the standard of care (SOC); OR is the odds ratio and CrI represents the credible interval.

Overall, 115 studies with 77,128 patients reported the number of patients requiring mechanical ventilation during the study period. We included ACEIs/ARBs, ammonium chloride, azithromycin, bamlanivimab, baricitinib plus remdesivir, bromhexine, budesonide, camostat mesilate, canakinumab, chloroquine, colchicine, convalescent plasma, dexamethasone, doxycycline, favipiravir, hydroxychloroquine, hydroxychloroquine plus azithromycin, hydroxychloroquine plus favipiravir, imatinib, INM005, interferon beta, intravenous immunoglobulin, ivermectin, lopinavir/ritonavir, methylprednisolone, recombinant human GCSF, remdesivir, sarilumab, sofosbuvir plus daclatasvir, sulodexide, tocilizumab, tofacitinib, vitamin D3 and SOC as treatment nodes in the NMA, for which observations came from 84 studies (3, 6, 22–26, 28–31, 35, 42, 43, 46, 47, 50, 53, 55, 57–61, 63, 64, 66, 67, 71, 73–77, 79, 80, 82, 83, 85–87, 89, 92–94, 96–100, 102, 105–107, 109, 111–118, 120–124, 126, 128–132, 134, 135, 139, 140, 145, 151, 152, 154–156). About one-third (26/84) of the included studies were evaluated as low risk (Supplementary Table 6). Compared with SOC, baricitinib plus remdesivir (OR 0.64, 95% CrI [0.42,0.98]; 1 RCT, 461 patients), colchicine (OR 0.42, 95% CrI [0.20,0.83]; 2 RCTs, 2,290 patients), dexamethasone (OR 0.66, 95% CrI [0.55, 0.79]; 4 RCTs, 1,998 patients), recombinant human GCSF (OR 0.25, 95% CrI [0.13, 0.48]; 1 RCT, 100 patients) and tocilizumab (OR 0.75, 95% CrI [0.65,0.86]; 8 RCTs, 2,564 patients) had significantly lower rates of mechanical ventilation (Figure 3). Immunostimulants (interferon beta and recombinant human GCSF) showed significant benefit on the reduction of mechanical ventilation with an OR of 0.51 (95% CrI [0.23, 0.97]). The classes of antigout preparations containing colchicine, cough and cold preparations including only bromhexine and immunosuppressants consisting of canakinumab, sarilumab, tocilizumab and tofacitinib were of potential benefit compared with SOC due to their relatively large posterior probability (higher than 0.9) favoring treatment and the other classes showed no difference from SOC.

The Bayesian random-effects NMA reported similar point estimates but with substantial wider interval estimates. Under the random-effects model, baricitinib plus remdesivir (OR 0.57, 95% CrI [0.25, 1.23]) and dexamethasone (OR 0.82, 95% CrI [0.58,1.25]) reported no significant difference from SOC, while colchicine (OR 0.40, 95% CrI [0.17, 0.91]), recombinant human GSCF (OR 0.40, 95% CrI [0.16, 0.78]) and tocilizumab (OR 0.72, 95% CrI [0.53,0.95]) yielded a significantly lower mechanical ventilation rate compared with SOC. Whether RECOVERY and PRINCIPLE were treated as multi-arm trials or multiple two-arm trials had no influence on the estimates of relative effects since the mechanical ventilation rates in the SOC groups of the four RECOVERY studies (3, 22, 23, 26) were similar and so was the PRINCIPLE trial (28–30). For mild/moderate COVID-19 patients, colchicine (OR 0.43, 95% CrI [0.20, 0.84]), dexamethasone (OR 0.62, 95% CrI [0.51, 0.76]), recombinant human GCSF (OR 0.22, 95% CrI [0.12, 0.42]) and intravenous immunoglobulin (OR 0.44, 95% CrI [0.20, 0.95]) led to a lower mechanical ventilation rate. For patients with severe/critical COVID-19 illness, dexamethasone (OR 0.65, 95% CrI [0.54, 0.78]), sarilumab (OR 0.70, 95% CrI [0.50, 0.94]), canakinumab (OR 0.69, 95% CrI [0.45, 0.98]) and tocilizumab (OR 0.74, 95% CrI [0.64,0.85]) could significantly reduce the occurrence of mechanical ventilation.

### Hospital Discharge (Closest to 14 Days)

**Figure 4:**
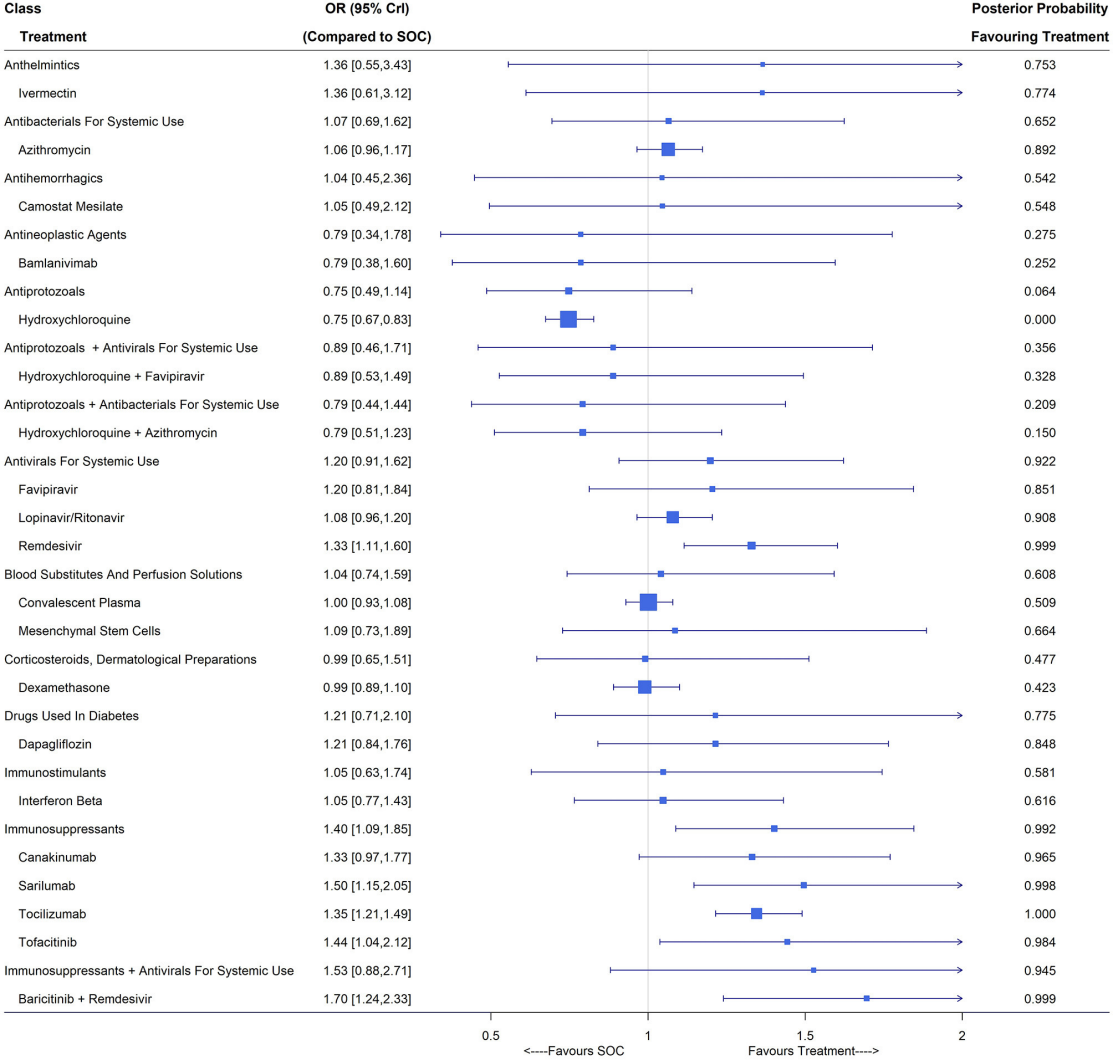
Discharge (closest to 14 days) under treatments compared with the standard of care (SOC); OR is the odds ratio and CrI represents the credible interval.

The hospital discharge rate was reported in 65 studies including 53,636 patients and 34,247 events. Treatment nodes included in the network were azithromycin, bamlanivimab, baricitinib plus remdesivir, camostat mesilate, canakinumab, convalescent plasma, dapagliflozin, dexamethasone, favipiravir, hydroxychloroquine, hydroxychloroquine plus azithromycin, hydroxychloroquine plus favipiravir, interferon beta, ivermectin, lopinavir/ritonavir, mesenchymal stem cells, remdesivir, sarilumab, tocilizumab, tofacitinib and SOC, which were investigated in 48 studies (3, 6, 22–26, 31, 43, 46, 55, 57, 64, 71–73, 76, 80, 82, 84, 89, 91, 93, 96–98, 100, 101, 106, 107, 109, 110, 115, 116, 121, 122, 124, 126, 128–132, 145–147, 157, 158). Out of the 48 studies included in the NMA, 19 were evaluated as low risk (Supplementary Table 7). Patients who received tofacitinib (OR 1.44, 95% CrI [1.04, 2.12]; 1 RCT, 144 patients), sarilumab (OR 1.50, 95% CrI [1.15,2.05]; 2 RCTs, 380 patients), remdesivir (OR 1.33, 95% CrI [1.11, 1.60]; 4 RCTs, 1,596 patients), tocilizumab (OR 1.35, 95% CrI [1.21, 1.49]; 7 RCTs, 3,014 patients) and baricitinib plus remdesivir (OR 1.70, 95% CrI [1.24, 2.33]; 1 RCTs, 515 patients) had a higher hospital discharge rate compared with those in the SOC arm. Hydroxychloroquine (OR 0.75, 95% CrI [0.67, 0.83]; 8 RCTs, 2,362 patients) was even inferior to SOC in terms of hospitalization at around 14 days (Figure 4). The use of immunosuppressants including canakinumab, sarilumab, tocilizumab and tofacitinib could significantly increase the discharge rate at day 14 (OR 1.40, 95% CrI [1.09, 1.85]). The classes of antivirals for systemic use (favipiravir, lopinavir/ritonavir and remdesivir) and the combination of immunosuppressants and antivirals for systemic use (baricitinib plus remdesivir) showed potential benefit in terms of hospital discharge with posterior probability favoring treatment larger than 0.9.

Under the random-effects model, with wider interval estimates, remdesivir (OR 1.31, 95% CrI [1.03,1.67]), tofacitinib (OR 1.44, 95% CrI [1.02, 2.15]), sarilumab (OR 1.50, 95% CrI [1.12,2.11]) and tocilizumab (OR 1.36, 95% CrI [1.13,1.64]) still maintained their significant benefit over SOC in terms of hospital discharge. The sensitivity analysis treating RECOVERY (3, 22, 26, 28) as four separated two-arm trials reported a significant OR of 1.19 (95% CrI [1.07,1.33]) for dexamethasone vs. SOC, in contrast to the primary analysis. Similar to the case when evaluating mortality, such discrepancy was caused by the different event rates in the two SOC arms used (3, 23). Evidence from subgroup analysis on patient illness indicated clinical benefit of baricitinib plus remdesivir (OR 1.61, 95% CrI [1.08, 2.45]) for non-severe COVID-19 patients, and remdesivir (OR 1.32, 95% CrI [1.08, 1.64]), interferon beta (OR 2.07, 95% CrI [1.21,3.59]), tofacitinib (OR 1.45, 95% CrI [1.04, 2.15]), sarilumab (OR 1.50, 95% CrI [1.15,2.06]) and tocilizumab (OR 1.35, 95% CrI [1.22, 1.50]) for patients with severe COVID-19.

### Viral Clearance (Closest to 7 Days)

**Figure 5:**
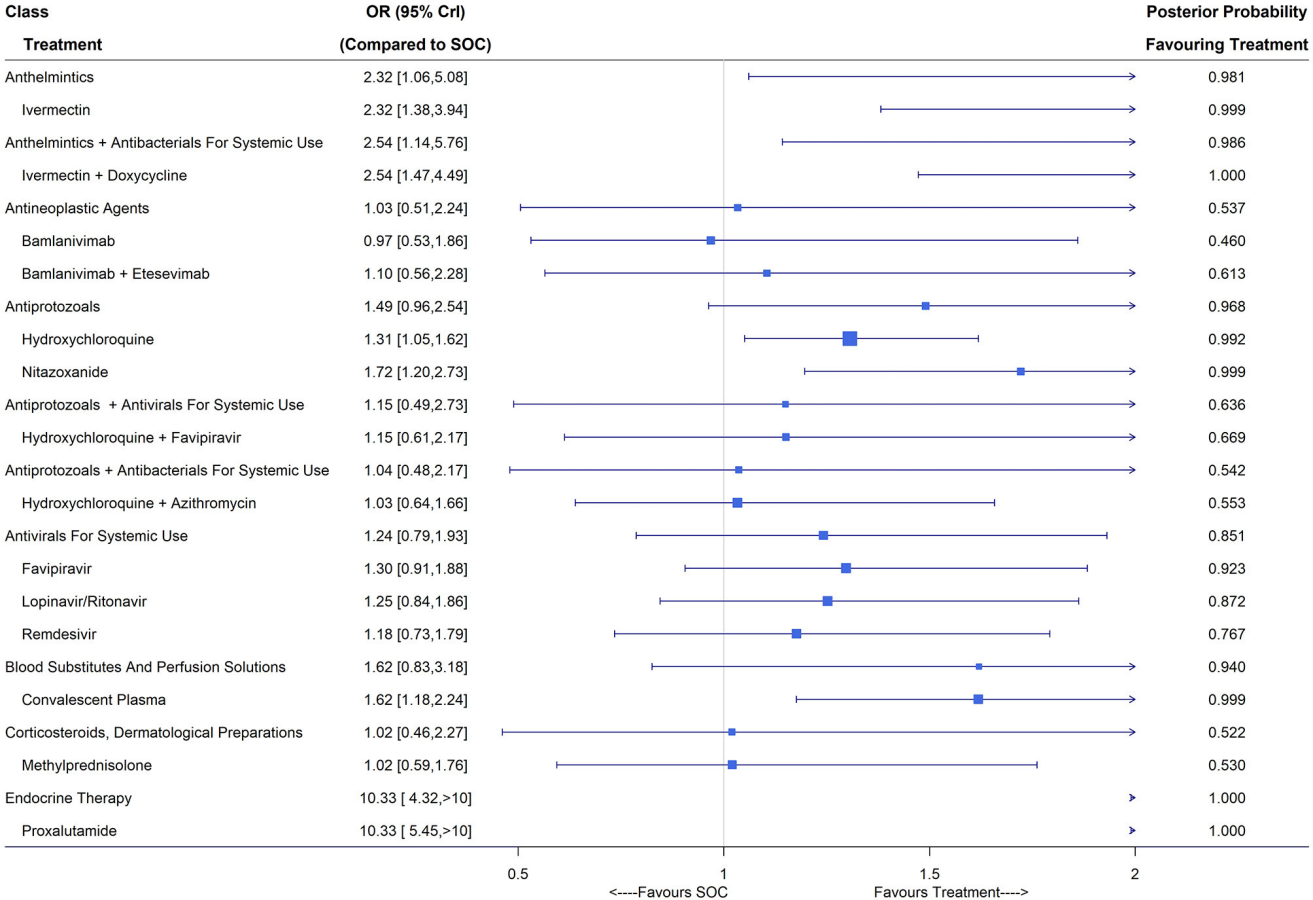
Viral clearance (closest to 7 days) of treatments compared with the standard of care (SOC); OR is the odds ratio and CrI represents the credible interval.

A total of 45 studies including 6,631 patients reported viral clearance rates and after eliminating treatments with inadequate numbers of patients, 32 studies were considered in the NMA (36, 53, 54, 56, 57, 59, 65, 68, 71, 72, 76, 78, 80, 91, 106, 109, 119, 132, 136, 137, 141, 153, 157–166), of which 10 were assessed as low risk (Supplementary Table 8). Treatment nodes in the network included bamlanivimab, bamlanivimab plus etesevimab, convalescent plasma, favipiravir, hydroxychloroquine, hydroxychloroquine plus azithromycin, hydroxychloroquine plus favipiravir, ivermectin, ivermectin plus doxycycline, lopinavir/ritonavir, methylprednisolone, nitazoxanide, proxalutamide, remdesivir and SOC. Under the fixed-effects NMA, convalescent plasma (OR 1.62, 95% CrI [1.18,2.24]; 4 RCTs, 344 patients), ivermectin (OR 2.32, 95% CrI [1.38,3.94]; 5 RCTs, 186 patients), ivermectin plus doxycycline (OR 2.54, 95% CrI [1.47, 4.49]; 2 RCTs, 206 patients), hydroxychloroquine (OR 1.31, 95% CrI [1.05,1.62]; 10 RCTs, 926 patients), nitazoxanide (OR 1.72, 95% CrI [1.20,2.73]; 2 RCTs, 217 patients) and proxalutamide (OR 10.33, 95% CrI [5.45, 20.36]; 1 RCT, 171 patients) showed significant improvements in the virologic cure (Figure 5). The classes of anthelmintics (ivermectin), anthelmintics plus antibacterials for systemic use (ivermectin plus doxycycline), antiprotozoals (hydroxychloroquine and nitazoxanide), blood substitutes and perfusion solutions (convalescent plasma) and endocrine therapy (proxalutamide) led to higher viral clearance rates compared with SOC with posterior probability favoring treatment larger than 0.9.

Under the random-effects model, convalescent plasma, ivermectin plus doxycycline, hydroxychloroquine and nitazoxanide did not show superiority over SOC, while ivermectin (OR 2.70, 95% CrI [1.24, 6.12]) and proxalutamide (OR 10.33, 95% CrI [2.72, 39.20]) were still effective in virus elimination. Trials published in multiple articles did not report viral clearance and thus no sensitivity analysis was carried out. Subgroup analysis (Supplementary Figure S12) revealed improved viral elimination using convalescent plasma, ivermectin, ivermectin plus doxycycline, hydroxychloroquine, nitazoxanide and proxalutamide compared with SOC for mild/moderate COVID-19 patients. For patients with severe/critical COVID-19, convalescent plasma (OR 2.74, 95% CrI [1.45, 5.27]) reported a higher viral clearance rate around day 7 after treatment compared with SOC (Supplementary Figure S13).

## Discussion

### Summary of Findings

In this systematic review and NMA, we provided a detailed summary of trial characteristics of published RCTs for confirmed COVID-19 patients up to August 19, 2021 and reported effectiveness of treatments at both the drug and class levels in terms of mortality, mechanical ventilation, hospital discharge and viral clearance. Compared with SOC, imatinib, intravenous immunoglobulin and tocilizumab were shown to reduce the risk of mortality; baricitinib plus remdesivir, colchicine, dexamethasone, recombinant human GCSF and tocilizumab resulted in fewer events of mechanical ventilation; patients who received convalescent plasma, ivermectin, ivermectin plus doxycycline, hydroxychloroquine, nitazoxanide and proxalutamide had a higher viral elimination rate; tofacitinib, sarilumab, remdesivir, tocilizumab and baricitinib plus remdesivir demonstrated their effectiveness with significantly higher 14-day hospital discharge rates.

At the class level of treatments, antineoplastic agents including bamlanivimab, imatinib and INM005 could reduce mortality; immunostimulants containing interferon beta and recombinant human GCSF showed clinical benefit over SOC in reducing mechanical ventilation; immunosuppressants consisting of canakinumab, sarilumab, tocilizumab and tofacitinib led to higher hospital discharge rates around 14 days, and the use of anthelmintics (ivermectin), anthelmintics plus antibacterials for systemic use (ivermectin plus doxycycline), endocrine therapy (proxalutamide) increased the rate of viral clearance on day 7. For other classes and outcomes, we observed no significant difference from SOC.

With an urgent need to identify effective treatments for COVID-19, researchers desired to aggregate information from individual trials investigating various interventions and, toward this goal, several NMAs for pharmacological interventions of COVID-19 have been published. Siemieniuk et al. (7) conducted a living systematic review and NMA for RCTs up to March 1, 2021 to evaluate the efficacy of potential COVID-19 treatments. They found corticosteroids (budesonide, dexamethasone, hydrocortisone and methylprednisolone) and Janus kinase inhibitors (baricitinib and ruxolitinib) could reduce death, mechanical ventilation, and increase the number of days free from mechanical ventilation; interleukin-6 inhibitors (tocilizumab and sarilumab) reduced mechanical ventilation and lengths of hospital stay. Kim et al. (167) reported improved outcomes for patients receiving anti-inflammatory agents (corticosteroids, tocilizumab, anakinra, and intravenous immunoglobulin), convalescent plasma, and remdesivir in their NMA including both RCTs and observational studies up to August 24, 2020. The majority of our findings are consistent with previous research except for the significant treatment effects of dexamethasone and corticosteroids due to the discrepancies among different SOC arms when we treated RECOVERY (3, 22, 23, 26) as one multi-arm trial. More treatments with clinical effectiveness against COVID-19 have been identified by including recently published studies in our NMA.

On the other hand, pairwise meta-analyses for a single drug vs. SOC have also revealed clinical benefits of potential COVID-19 treatments with accumulated evidence from completed studies. For example, tocilizumab led to reduction in mortality (168, 169), ventilation (170) and biomarkers of the COVID-19 infection (171); patients receiving ivermectin had a lower risk of death as well as an increase in the viral clearance rate (172, 173); the administration of colchicine resulted in a lower risk of mortality and improvements of clinical outcomes (174); remdesivir showed its superiority over SOC with faster recovery, shorter time to clinical improvement and reduction in mortality (175, 176).

### Strength and Limitations

Not only was this NMA timely conducted, but it also included a wide range of RCTs, which contained a large number of common drugs as well as interferons, blood products, mineral and vitamin supplementations. Treatment effects were evaluated in a network at both the individual drug level and class level. Using a hierarchical Bayesian model based on the WHO ATC/DDD classification rule, we grouped the treatments from a scientific and pharmacological perspective and provided a further guideline for discovery of new treatments on COVID-19.

This study has several limitations. One is the low certainty of evidence for many NMA estimates. At the early stage of COVID-19 pandemic, with limited clinical resources and urgent need to obtain trial results, many RCTs were conducted with simplified procedures, e.g., no placebo prepared, leading to downgrading of evidence due to study limitations (177). Over time, the situation has gradually improved and many double-blind RCTs have been conducted and published recently. Moreover, networks of treatments were sparse because most of the included studies evaluated interventions vs. SOC and there were few direct comparisons among interventions. As we considered COVID-19 RCTs regardless of demographic characteristics, intransitivity existed in many indirect comparisons. For example, hydroxychloroquine trials usually investigated patients with mild/moderate COVID-19, while patients treated by convalescent plasma were mainly of severe/critical illness. Detailed subgroup analysis might help to resolve such problems (see Supplementary Materials).

Another limitation of this study arises from the evaluation of NMA estimates at the class level. Many investigated classes contained only one treatment, leading to large variation and thus insignificant results. To confirm the superiority of a class of drugs, one should present evidence of stronger strengths. More treatments could have been included in the NMA if the exclusion criteria of treatment nodes were relaxed, while it would inevitably introduce additional bias due to treatments tested with small sample sizes.

In the primary analysis, we only included peer-reviewed studies to maintain the credibility of evidence. However, among such a large number of completed COVID-19 trials, studies reporting positive results or with large sample size were more likely to be published, leading to possible publication bias (178). To alleviate the potential publication bias, we conducted an exploratory analysis including both peer-reviewed papers and preprints from unpublished studies, for which results were shown in the Supplementary Figures S14–S17. While caution should be taken on the evidence implied by only preprints since clinical results without peer-reviews should not be trusted equally as those published.

In addition, we mainly focused on the efficacy of interventions in this NMA and did not evaluate the corresponding safety profiles. Evidence from other NMAs (7, 179) showed that most of investigated treatments in this NMA did not lead to increased adverse events, and remdesivir and lopinavir/ritonavir were associated with fewer occurrences of adverse events and serious adverse events, respectively.

Different approaches to dealing with the RECOVERY, REMAP-CAP, PRINCIPLE, BLAZE-1 and therapeutic anticoagulation trials led to discrepancies between results of the primary and sensitivity analyses, especially for dexamethasone. The RECOVERY trial was designed as a multi-arm trial (27) while the numbers of patients randomized to SOC and event rates of outcomes of interest were different across different reports (3, 22, 23, 26). Although we observed no clinical benefit on the reduction of mortality and increase of the hospital discharge rate for dexamethasone vs. SOC, the sensitivity analysis drew an opposite conclusion and credibility of this finding warrants extra caution.

### Conclusion

This systematic review and NMA showed that imatinib, intravenous immunoglobulin and tocilizumab could reduce the mortality. Patients receiving baricitinib plus remdesivir, colchicine, dexamethasone, recombinant human GCSF and tocilizumab had a lower risk of mechanical ventilation. Administration of tofacitinib, sarilumab, remdesivir, tocilizumab and baricitinib plus remdesivir led to higher hospital discharge rates. Convalescent plasma, ivermectin, ivermectin plus doxycycline, hydroxychloroquine, nitazoxanide and proxalutamide could improve the viral elimination.

At the treatment class level, compared with SOC, patients receiving antineoplastic agents had a lower risk of death; immunostimulants tended to reduce the need of mechanical ventilation; the use of immunosuppressants led to an increased hospital discharge rate; anthelmintics, anthelmintics plus antibacterials for systemic use and endocrine therapy showed clinical improvements on viral clearance, while these three classes contained only one treatment, for which the evidence might not be sound.

The clinical benefits of several treatments on confirmed COVID-19 patients have been reported in this study. The endpoints of mortality and mechanical ventilation can be viewed as the deterioration of COVID-19 illness, and for clinicians and patients with severe COVID-19, these effective treatments (e.g., tocilizumab, imatinib, intravenous immunoglobulin, dexamethasone) can prevent or alleviate the progression of disease. Hospital discharge and viral clearance represent the recovery from COVID-19, and patients with mild or moderate illness might suffer less from the SARS-COV2 infection. Overall, tocilizumab performed the best against COVID-19 compared with SOC, which showed its superiority in terms of lower mortality and mechanical ventilation rates as well as a higher hosptical discharge rate.

On the other hand, we found the significance of classes of treatments on each investigated endpoint. The discovery of effective treatments on COVID-19 is still an essential issue, especially after the occurrence of more infective and fatal variants. The efficacy of antineoplastic agents, immunostimulants and immunosuppressants on reduced risk of death, mechanical ventilation and increased hosptical discharge, respectively, was shown by our NMA with sound statistical evidence, which shed new light on further research and discovery of potential COVID-19 treatments. Further large clinical trials are still needed to confirm these results.

## Data Availability Statement

The original contributions presented in the study are included in the article/Supplementary Material, further inquiries can be directed to the corresponding author/s.

## Author Contributions

CZ and GY contributed to the conception and design of the work. CZ, HJ, and YW collected information and analyzed data used in the systematic review and meta-analysis. CZ and HJ drafted the work. YW and GY substantively revised it. All authors read and approved the submitted version and agreed to be personally accountable for the authors' own contributions and to ensure the accuracy and integrity of the work.

## Funding

This study was supported by the Research Grants Council of Hong Kong (17308420).

## Conflict of Interest

The authors declare that the research was conducted in the absence of any commercial or financial relationships that could be construed as a potential conflict of interest.

## Publisher's Note

All claims expressed in this article are solely those of the authors and do not necessarily represent those of their affiliated organizations, or those of the publisher, the editors and the reviewers. Any product that may be evaluated in this article, or claim that may be made by its manufacturer, is not guaranteed or endorsed by the publisher.

## References

[1] Coronavirus resource center JHU. COVID-19 Dashboard by the Center for Systems Science and Engineering (CSSE) at Johns Hopkins University (JHU) (2021). Available online at: https://coronavirus.jhu.edu/map.html

[2] World Health Organization. International Clinical Trials Registry Platform (ICTRP) (2021) Available online at: https://www.who.int/clinical-trials-registry-platform

[3] Recovery Collaborative Group Horby P Lim WS Emberson JR Mafham M Bell JL . Dexamethasone in hospitalized patients with covid-19. N Engl J Med. (2021) 384:693–704. 10.1056/NEJMoa2021436 32678530 PMC7383595

[4] World Health Organization. Corticosteroids for COVID-19 (2020). Available from: https://www.who.int/publications/i/item/WHO-2019-nCoV-Corticosteroids-2020.1

[5] Rochwerg B Agarwal A Zeng L Leo YS Appiah JA Agoritsas T . Remdesivir for severe covid-19: a clinical practice guideline. BMJ. (2020) 370:m2924. 10.1136/bmj.m2924 32732352

[6] Beigel JH Tomashek KM Dodd LE Mehta AK Zingman BS Kalil AC . Remdesivir for the treatment of covid-19 - final report. N Engl J Med. (2020) 383:1813–26. 10.1056/NEJMoa2007764 32445440 PMC7262788

[7] Siemieniuk RA Bartoszko JJ Ge L Zeraatkar D Izcovich A Kum E . Drug treatments for covid-19: living systematic review and network meta-analysis. BMJ. (2020) 370:m2980. 10.1136/bmj.m2980 32732190 PMC7390912

[8] Yokoyama Y Briasoulis A Takagi H Kuno T. Effect of remdesivir on patients with COVID-19: A network meta-analysis of randomized control trials. Virus Res. (2020) 288:198137. 10.1016/j.virusres.2020.198137 32827627 PMC7437510

[9] Pushpakom S Iorio F Eyers PA Escott KJ Hopper S Wells A . Drug repurposing: progress, challenges and recommendations. Nat Rev Drug Discov. (2019) 18:41–58. 10.1038/nrd.2018.168 30310233

[10] The WHO. Rapid Evidence Appraisal for COVID-19 therapies working group. Association between administration of systemic corticosteroids and mortality among critically Ill patients with COVID-19: a meta-analysis. JAMA. (2020) 324:1330–41. 10.1001/jama.2020.17023 32876694 PMC7489434

[11] Hutton B Salanti G Caldwell DM Chaimani A Schmid CH Cameron C . The PRISMA extension statement for reporting of systematic reviews incorporating network meta-analyses of health care interventions: checklist and explanations. Ann Intern Med. (2015) 162:777–84. 10.7326/M14-2385 26030634

[12] World Health Organization. Global research on coronavirus disease (COVID-19) (2020) Available from: https://www.who.int/emergencies/diseases/novel-coronavirus-2019/global-research-on-novel-coronavirus-2019-ncov

[13] McCaw ZR Tian L Kim DH Localio AR Wei LJ. Survival analysis of treatment efficacy in comparative COVID-19 studies. Clin Infect Dis. (2021) 72:e887–9. 10.1093/cid/ciaa1563 33053155 PMC7665361

[14] Puhan MA Schunemann HJ Murad MH Li T Brignardello-Petersen R Singh JA . A GRADE Working Group approach for rating the quality of treatment effect estimates from network meta-analysis. BMJ. (2014) 349:g5630. 10.1136/bmj.g5630 25252733

[15] Sterne JAC Savovic J Page MJ Elbers RG Blencowe NS Boutron I . RoB 2: a revised tool for assessing risk of bias in randomised trials. BMJ. (2019) 366:l4898. 10.1136/bmj.l4898 31462531

[16] Csardi G Nepusz T. The igraph software package for complex network research. IntJ Complex Syst. (2006) 1695:1–9.

[17] World Health Organization Collaborating Centre for Drug Statistics Methodology. Guidelines for ATC classification and DDD assignment (2020). Available online at: https://www.whocc.no/filearchive/publications/2020_guidelines_web.pdf

[18] van Valkenhoef G Dias S Ades AE Welton NJ. Automated generation of node-splitting models for assessment of inconsistency in network meta-analysis. Res Synth Methods. (2016) 7:80–93. 10.1002/jrsm.1167 26461181 PMC5057346

[19] Plummer M. JAGS: A program for analysis of Bayesian graphical models using Gibbs sampling. Proceedings of the 3rd international workshop on distributed statistical computing. Vienna, Austria. (2003)

[20] Kellner K. jagsUI: a wrapper around rjags to streamline JAGS analyses. R package version 151. (2019). Available online at: https://cran.r-project.org/web/packages/jagsUI

[21] van Valkenhoef G Kuiper J. gemtc: Network Meta-Analysis Using Bayesian Methods. R package version 08-7. (2020). Available online at: https://cran.r-project.org/web/packages/gemtc/

[22] RECOVERY Collaborative Group. Lopinavir-ritonavir in patients admitted to hospital with COVID-19 (RECOVERY): a randomised, controlled, open-label, platform trial. Lancet. (2020) 396:1345–52. 10.1016/S0140-6736(20)32013-4 33031764 PMC7535623

[23] RECOVERY Collaborative Group. Azithromycin in patients admitted to hospital with COVID-19 (RECOVERY): a randomised, controlled, open-label, platform trial. Lancet. (2021) 397:605–12. 10.1016/S0140-6736(21)00149-5 33545096 PMC7884931

[24] RECOVERY Collaborative Group. Tocilizumab in patients admitted to hospital with COVID-19 (RECOVERY): a randomised, controlled, open-label, platform trial. Lancet. (2021) 397:1637–45. 10.1016/S0140-6736(21)00676-0 33933206 PMC8084355

[25] RECOVERY Collaborative Group. Convalescent plasma in patients admitted to hospital with COVID-19 (RECOVERY): a randomised controlled, open-label, platform trial. Lancet. (2021) 397:2049–59. 10.1016/S0140-6736(21)01064-3 34000257 PMC8121538

[26] RECOVERY Collaborative group. effect of hydroxychloroquine in hospitalized patients with covid-19. N Eng J Med. (2020) 383:2030–40. 10.1056/NEJMoa2022926 33031652 PMC7556338

[27] RECOVERY Collaborative Group. Study protocol & statistical analysis plan archive 2020. Available online from: https://www.recoverytrial.net/files/protocol-archive/recovery-protocol-v6-0-2020-05-14.pdf

[28] Principle Trial Collaborative Group. Azithromycin for community treatment of suspected COVID-19 in people at increased risk of an adverse clinical course in the UK (PRINCIPLE): a randomised, controlled, open-label, adaptive platform trial. Lancet. (2021) 397:1063–74. 10.1016/S0140-6736(21)00461-X 33676597 PMC7972318

[29] Yu L-M Bafadhel M Dorward J Hayward G Saville BR Gbinigie O . Inhaled budesonide for COVID-19 in people at high risk of complications in the community in the UK (PRINCIPLE): a randomised, controlled, open-label, adaptive platform trial. Lancet. (2021) 398:843–55. 10.1016/S0140-6736(21)01744-X 34388395 PMC8354567

[30] Butler CC Yu L-M Dorward J Gbinigie O Hayward G Saville BR . Doxycycline for community treatment of suspected COVID-19 in people at high risk of adverse outcomes in the UK (PRINCIPLE): a randomised, controlled, open-label, adaptive platform trial. Lancet Respir Med. (2021) 9:1010–20. 10.1016/S2213-2600(21)00310-6 34329624 PMC8315758

[31] REMAP-CAP Investigators Gordon AC Mouncey PR Al-Beidh F Rowan KM Nichol AD . Interleukin-6 receptor antagonists in critically Ill Patients with Covid-19. N Engl J Med. (2021) 384:1491–502. 10.1056/NEJMoa2100433 33631065 PMC7953461

[32] Angus DC Derde L Al-Beidh F Annane D Arabi Y Beane A . Effect of hydrocortisone on mortality and organ support in patients with severe COVID-19: The REMAP-CAP COVID-19 corticosteroid domain randomized clinical trial. JAMA. (2020) 324:1317–29. 10.1001/jama.2020.17022 32876697 PMC7489418

[33] Arabi YM Gordon AC Derde LPG Nichol AD Murthy S Beidh FA . Lopinavir-ritonavir and hydroxychloroquine for critically ill patients with COVID-19: REMAP-CAP randomized controlled trial. Intensive Care Med. (2021) 47:867–86. 10.1007/s00134-021-06448-5 34251506 PMC8274471

[34] Ader F Peiffer-Smadja N Poissy J Bouscambert-Duchamp M Belhadi D Diallo A . Antiviral drugs in hospitalized patients with COVID-19 - the DisCoVeRy trial. medRxiv [Preprint]. (2021). 10.1101/2021.01.08.2024814932958495

[35] WHO Solidarity Trial Consortium Pan H Peto R Henao-Restrepo AM Preziosi MP Sathiyamoorthy V . Repurposed antiviral drugs for covid-19 - interim WHO solidarity trial results. N Engl J Med. (2021) 384:497–511. 10.1056/NEJMoa2023184 33264556 PMC7727327

[36] Gottlieb RL Nirula A Chen P Boscia J Heller B Morris J . Effect of bamlanivimab as monotherapy or in combination with etesevimab on viral load in patients with mild to moderate COVID-19: a randomized clinical trial. JAMA. (2021) 325:632–44. 10.1001/jama.2021.0202 33475701 PMC7821080

[37] Dougan M Nirula A Azizad M Mocherla B Gottlieb RL Chen P . Bamlanivimab plus etesevimab in mild or moderate covid-19. N Engl J Med. (2021). 10.1056/NEJMoa2102685 34260849 PMC8314785

[38] ATTACC Investigators ACTIV-4a Investigators REMAP-CAP Investigators Lawler PR Goligher EC Berger JS . Therapeutic anticoagulation with heparin in noncritically Ill patients with covid-19. N Engl J Med. (2021) 385:790–802. 10.1056/NEJMoa2105911 34351721 PMC8362594

[39] ATTACC Investigators ACTIV-4a Investigators REMAP-CAP Investigators Goligher EC Bradbury CA McVerry BJ . Therapeutic Anticoagulation with heparin in critically Ill patients with covid-19. N Engl J Med. (2021) 385:777–89. 10.1056/NEJMoa2103417 34351722 PMC8362592

[40] Munch MW Meyhoff TS Helleberg M Kjaer MN Granholm A Hjortso CJS . Low-dose hydrocortisone in patients with COVID-19 and severe hypoxia: the COVID STEROID randomised, placebo-controlled trial. Acta Anaesthesiol Scand. (2021). 10.1111/aas.13941. [Epub ahead of print]. 34138478 PMC8441888

[41] Najmeddin F Solhjoo M Ashraf H Salehi M Rasooli F Ghoghaei M . Effects of renin-angiotensin-aldosterone inhibitors on early outcomes of hypertensive COVID-19 Patients: a randomized triple-blind clinical trial. Am J Hypertens. (2021). 10.1093/ajh/hpab111. [Epub ahead of print]. 34265044 PMC8344947

[42] Abd-Elsalam S Esmail ES Khalaf M Abdo EF Medhat MA Abd El Ghafar MS . Hydroxychloroquine in the treatment of COVID-19: a multicenter randomized controlled study. Am J Trop Med Hyg. (2020) 103:1635–9. 10.4269/ajtmh.20-0873 32828135 PMC7543820

[43] Brown SM Peltan I Kumar N Leither L Webb BJ Starr N . Hydroxychloroquine vs. azithromycin for hospitalized patients with COVID-19 (hahps): results of a randomized, active comparator trial. Ann Am Thorac Soc. (2020) 18:590–7. 10.1513/AnnalsATS.202004-309SD 33166179 PMC8009003

[44] Barratt-Due A Olsen IC Nezvalova-Henriksen K Kasine T Lund-Johansen F Hoel H . Evaluation of the effects of remdesivir and hydroxychloroquine on viral clearance in COVID-19: A randomized trial. Ann Intern Med. (2021). 10.7326/M21-0653. [Epub ahead of print]. 34251903 PMC8279143

[45] Skipper CP Pastick KA Engen NW Bangdiwala AS Abassi M Lofgren SM . Hydroxychloroquine in nonhospitalized adults with early covid-19: a randomized trial. Ann Intern Med. (2020) 173:623–31. 10.7326/M20-4207 32673060 PMC7384270

[46] Davoudi-Monfared E Rahmani H Khalili H Hajiabdolbaghi M Salehi M Abbasian L . A randomized clinical trial of the efficacy and safety of interferon β-1a in treatment of severe COVID-19. Antimicrobial Agents and Chemotherapy. (2020) 64:e01061–20. 10.1128/AAC.01061-20 32661006 PMC7449227

[47] Dabbous HM Abd-Elsalam S El-Sayed MH Sherief AF Ebeid FFS El Ghafar MSA . Efficacy of favipiravir in COVID-19 treatment: a multi-center randomized study. Arch Virol. (2021) 166:949–54. 10.1007/s00705-021-04956-9 33492523 PMC7829645

[48] Shakhsi Niaee M Namdar P Allami A Zolghadr L Javadi A Karampour A . Ivermectin as an adjunct treatment for hospitalized adult COVID-19 patients: A randomized multi-center clinical trial. Asian Pac J Trop Med. (2021) 14:266–73. 10.4103/1995-7645.318304

[49] Okumus N Demirturk N Cetinkaya RA Guner R Avci IY Orhan S . Evaluation of the effectiveness and safety of adding ivermectin to treatment in severe COVID-19 patients. BMC Infect Dis. (2021) 21:411. 10.1186/s12879-021-06104-9 33947344 PMC8093585

[50] Ranjbar K Moghadami M Mirahmadizadeh A Fallahi MJ Khaloo V Shahriarirad R . Methylprednisolone or dexamethasone, which one is superior corticosteroid in the treatment of hospitalized COVID-19 patients: a triple-blinded randomized controlled trial. BMC Infect Dis. (2021) 21:337. 10.1186/s12879-021-06045-3 33838657 PMC8035859

[51] Vallejos J Zoni R Bangher M Villamandos S Bobadilla A Plano F . Ivermectin to prevent hospitalizations in patients with COVID-19 (IVERCOR-COVID19) a randomized, double-blind, placebo-controlled trial. BMC Infect Dis. (2021) 21:635. 10.1186/s12879-021-06348-5 34215210 PMC8250562

[52] Gharebaghi N Nejadrahim R Mousavi SJ Sadat-Ebrahimi S-R Hajizadeh R. The use of intravenous immunoglobulin gamma for the treatment of severe coronavirus disease 2019: a randomized placebo-controlled double-blind clinical trial. BMC Infect Dis. (2020) 20:786. 10.1186/s12879-020-05507-4 33087047 PMC7576972

[53] Agarwal A Mukherjee A Kumar G Chatterjee P Bhatnagar T Malhotra P. Convalescent plasma in the management of moderate covid-19 in adults in India: open label phase II multicentre randomised controlled trial (PLACID Trial). BMJ. (2020) 371:m3939. 10.1136/bmj.m3939 33093056 PMC7578662

[54] Tang W Cao Z Han M Wang Z Chen J Sun W . Hydroxychloroquine in patients with mainly mild to moderate coronavirus disease 2019: open label, randomised controlled trial. BMJ. (2020) 369:m1849. 10.1136/bmj.m1849 32409561 PMC7221473

[55] Veiga VC Prats JAGG Farias DLC Rosa RG Dourado LK Zampieri FG . Effect of tocilizumab on clinical outcomes at 15 days in patients with severe or critical coronavirus disease 2019: randomised controlled trial. BMJ. (2021) 372:n84. 10.1136/bmj.n84 33472855 PMC7815251

[56] Kirenga B Byakika-Kibwika P Muttamba W Kayongo A Loryndah NO Mugenyi L . Efficacy of convalescent plasma for treatment of COVID-19 in Uganda. BMJ Open Respir Res. (2021) 8:e001017. 10.1136/bmjresp-2021-001017 34376401 PMC8354811

[57] Dubee V Roy PM Vielle B Parot-Schinkel E Blanchet O Darsonval A . Hydroxychloroquine in mild-to-moderate COVID-19: a placebo-controlled double blind trial. Clin Microbiol Infect. (2021) 27:1124–30. 10.1101/2020.10.19.20214940 33813110 PMC8015393

[58] Shahbaznejad L Davoudi A Eslami G Markowitz JS Navaeifar MR Hosseinzadeh F . Effects of ivermectin in patients with COVID-19: a multicenter, double-blind, randomized, controlled clinical trial. Clin Ther. (2021). 10.1016/j.clinthera.2021.04.007 34052007 PMC8101859

[59] Jeronimo CMP Farias MEL Val FFA Sampaio VS Alexandre MAA Melo GC . Methylprednisolone as Adjunctive Therapy for Patients Hospitalized With Coronavirus Disease 2019 (COVID-19; Metcovid): A Randomized, Double-blind, Phase IIb, Placebo-controlled Trial. Clin Infect Dis. (2021) 72:e373–81. 10.1093/cid/ciaa1177 32785710 PMC7454320

[60] Mitjà O Corbacho-Monné M Ubals M Tebe C Peñafiel J Tobias A . BCN PEP-CoV-2 RESEARCH GROUP. hydroxychloroquine for early treatment of adults with mild covid-19: a randomized-controlled trial. Clin Infect Dis. (2020). 10.1093/cid/ciaa1009. [Epub ahead of print]. 32674126 PMC7454406

[61] Schwartz I Boesen ME Cerchiaro G Doram C Edwards BD Ganesh A . Assessing the efficacy and safety of hydroxychloroquine as outpatient treatment of COVID-19: a randomized controlled trial. CMAJ Open. (2021) 9:e693–702. 10.9778/cmajo.20210069 34145052 PMC8248582

[62] Bennett-Guerrero E Romeiser JL Talbot LR Ahmed T Mamone LJ Singh SM . Severe acute respiratory syndrome coronavirus 2 convalescent plasma vs. standard plasma in coronavirus disease 2019 infected hospitalized patients in New York: A double-blind randomized trial. Crit Care Med. (2021) 49:1015–25. 10.1097/CCM.0000000000005066 33870923 PMC9658886

[63] Ali S Uddin SM Shalim E Sayeed MA Anjum F Saleem F . Hyperimmune anti-COVID-19 IVIG (C-IVIG) treatment in severe and critical COVID-19 patients: A phase I/II randomized control trial. EClinicalMedicine. (2021) 36:100926. 10.1016/j.eclinm.2021.100926 34109306 PMC8177439

[64] Gunst JD Staerke NB Pahus MH Kristensen LH Bodilsen J Lohse N . Efficacy of the TMPRSS2 inhibitor camostat mesilate in patients hospitalized with Covid-19-a double-blind randomized controlled trial. EClinicalMedicine. (2021) 35:100849. 10.1016/j.eclinm.2021.100849 33903855 PMC8060682

[65] Johnston C Brown ER Stewart J Karita HCS Kissinger PJ Dwyer J . Hydroxychloroquine with or without azithromycin for treatment of early SARS-CoV-2 infection among high-risk outpatient adults: A randomized clinical trial. EClinicalMedicine. (2021) 33:100773. 10.1016/j.eclinm.2021.100773 33681731 PMC7912360

[66] Krolewiecki A Lifschitz A Moragas M Travacio M Valentini R Alonso DF . Antiviral effect of high-dose ivermectin in adults with COVID-19: A proof-of-concept randomized trial. EClinicalMedicine. (2021) 37:100959. 10.1016/j.eclinm.2021.100959 34189446 PMC8225706

[67] Lopardo G Belloso WH Nannini E Colonna M Sanguineti S Zylberman V . RBD-specific polyclonal F(ab)2 fragments of equine antibodies in patients with moderate to severe COVID-19 disease: A randomized, multicenter, double-blind, placebo-controlled, adaptive phase 2/3 clinical trial. EClinicalMedicine. (2021) 34:100843. 10.1016/j.eclinm.2021.100843 33870149 PMC8037439

[68] Omrani AS Pathan SA Thomas SA Harris TRE Coyle PV Thomas CE . Randomized double-blinded placebo-controlled trial of hydroxychloroquine with or without azithromycin for virologic cure of non-severe Covid-19. EClinicalMedicine. (2020) 29–30:100645. 10.1016/j.eclinm.2020.100645 33251500 PMC7678437

[69] Elamir Y Amir H Feliciano N Gonzalez C Grist W Lim S . Abstract #1002919: endocrine therapy for COVID19: A randomized pilot study using calcitriol. Endocr Pract. (2021) 27:S79. 10.1016/j.eprac.2021.04.636

[70] JamaliMoghadamSiahkali S Zarezade B Koolaji S SeyedAlinaghi S Zendehdel A Tabarestani M . Safety and effectiveness of high-dose vitamin C in patients with COVID-19: a randomized open-label clinical trial. Eur J Med Res. (2021) 26:20. 10.1186/s40001-021-00490-1 33573699 PMC7877333

[71] Lou Y Liu L Yao H Hu X Su J Xu K . Clinical outcomes and plasma concentrations of baloxavir marboxil and favipiravir in COVID-19 patients: an exploratory randomized, controlled trial. Eur J Pharm Sci. (2020) 157:105631. 10.1101/2020.04.29.20085761 33115675 PMC7585719

[72] Sekine L Arns B Fabro BR Cipolatt MM Machado RRG Durigon EL . Convalescent plasma for COVID-19 in hospitalised patients: an open-label, randomised clinical trial. Eur Respir J. (2021). 10.1183/13993003.01471-2021. [Epub ahead of print]. 34244316 PMC8287736

[73] Sivapalan P Suppli Ulrik C Sophie Lapperre T Dahlin Bojesen R Eklof J Browatzki A . Azithromycin and hydroxychloroquine in hospitalised patients with confirmed COVID-19-a randomised double-blinded placebo-controlled trial. Eur Respir J. (2021). 10.1183/13993003.00752-2021. [Epub ahead of print]. 34083403 PMC8186006

[74] Jamaati H Hashemian SM Farzanegan B Malekmohammad M Tabarsi P Marjani M . No clinical benefit of high dose corticosteroid administration in patients with COVID-19: A preliminary report of a randomized clinical trial. Eur J Pharmacol. (2021) 897:173947. 10.1016/j.ejphar.2021.173947 33607104 PMC7885705

[75] Mahajan L Singh AP. Gifty. Clinical outcomes of using remdesivir in patients with moderate to severe COVID-19: A prospective randomised study. Indian J Anaesth. (2021) 65:S41–6. 10.4103/ija.IJA_149_21 33814589 PMC7993042

[76] Bosaeed M Mahmoud E Alharbi A Altayib H Albayat H Alharbi F . Favipiravir and hydroxychloroquine combination therapy in patients with moderate to severe COVID-19 (FACCT Trial): an open-label, multicenter, randomized, controlled trial. Infect Dis Ther. (2021). 10.1007/s40121-021-00496-6. [Epub ahead of print]. 34319552 PMC8316887

[77] Solaymani-Dodaran M Ghanei M Bagheri M Qazvini A Vahedi E Hassan Saadat S . Safety and efficacy of Favipiravir in moderate to severe SARS-CoV-2 pneumonia. Int Immunopharmacol. (2021) 95:107522. 10.1016/j.intimp.2021.107522 33735712 PMC7951885

[78] Zhao H Zhang C Zhu Q Chen X Chen G Sun W . Favipiravir in the treatment of patients with SARS-CoV-2 RNA recurrent positive after discharge: A multicenter, open-label, randomized trial. Int Immunopharmacol. (2021) 97:107702. 10.1016/j.intimp.2021.107702 33930706 PMC8059985

[79] Siami Z Aghajanian S Mansouri S Mokhames Z Pakzad R Kabir K . Effect of Ammonium Chloride in addition to standard of care in outpatients and hospitalized COVID-19 patients: a randomized clinical trial. Int J Infect Dis. (2021) 108:306–8. 10.1016/j.ijid.2021.04.043 33878462 PMC8053358

[80] Udwadia ZF Singh P Barkate H Patil S Rangwala S Pendse A . Efficacy and safety of favipiravir, an oral RNA-dependent RNA polymerase inhibitor, in mild-to-moderate COVID-19: A randomized, comparative, open-label, multicenter, phase 3 clinical trial. Int J Infect Dis. (2021) 103:62–71. 10.1016/j.ijid.2020.11.142 33212256 PMC7668212

[81] Pouladzadeh M Safdarian M Eshghi P Abolghasemi H Bavani AG Sheibani B . A randomized clinical trial evaluating the immunomodulatory effect of convalescent plasma on COVID-19-related cytokine storm. Intern Emerg Med. (2021). 10.1007/s11739-021-02734-8. [Epub ahead of print]. 33837906 PMC8035885

[82] Rahmani H Davoudi-Monfared E Nourian A Khalili H Hajizadeh N Jalalabadi NZ . Interferon β-1b in treatment of severe COVID-19: A randomized clinical trial. Int Immunopharmacol. (2020) 88:106903. 10.1016/j.intimp.2020.106903 32862111 PMC7445008

[83] Tabarsi P Barati S Jamaati H Haseli S Marjani M Moniri A . Evaluating the effects of Intravenous Immunoglobulin (IVIg) on the management of severe COVID-19 cases: A randomized controlled trial. Int Immunopharmacol. (2020) 90:107205. 10.1016/j.intimp.2020.107205 33214093 PMC7665876

[84] O'Donnell MR Grinsztejn B Cummings MJ Justman JE Lamb MR Eckhardt CM . A randomized double-blind controlled trial of convalescent plasma in adults with severe COVID-19. J Clin Invest. (2021) 131:e150646. 10.1172/JCI150646 33974559 PMC8245169

[85] Raman RS Bhagwan Barge V Anil Kumar D Dandu H Rakesh Kartha R Bafna V . A phase II safety and efficacy study on prognosis of moderate pneumonia in coronavirus disease 2019 patients with regular intravenous immunoglobulin therapy. J Infect Dis. (2021) 223:1538–43. 10.1093/infdis/jiab098 33585890 PMC7928808

[86] Abbass S Kamal E Salama M Salman T Sabry A Abdel-Razek W . Efficacy and safety of sofosbuvir plus daclatasvir or ravidasvir in patients with COVID-19: A randomized controlled trial. J Med Virol. (2021). 10.1002/jmv.27264. [Epub ahead of print]. 34379337 PMC8426808

[87] Abd-Elsalam S Noor RA Badawi R Khalaf M Esmail ES Soliman S . Clinical study evaluating the efficacy of ivermectin in COVID-19 treatment: A randomized controlled study. J Med Virol. (2021) 93:5833–8. 10.1002/jmv.27122 34076901 PMC8242425

[88] Entrenas Castillo M Entrenas Costa LM Vaquero Barrios JM Alcala Diaz JF Lopez Miranda J Bouillon R . Effect of calcifediol treatment and best available therapy vs. best available therapy on intensive care unit admission and mortality among patients hospitalized for COVID-19: A pilot randomized clinical study. J Steroid Biochem Mol Biol. (2020) 203:105751. 10.1016/j.jsbmb.2020.105751 32871238 PMC7456194

[89] Caricchio R Abbate A Gordeev I Meng J Hsue PY Neogi T . Effect of canakinumab vs placebo on survival without invasive mechanical ventilation in patients hospitalized with severe COVID-19: a randomized clinical trial. JAMA. (2021) 326:230–9. 10.1001/jama.2021.9508 34283183 PMC8293025

[90] Dequin P-F Heming N Meziani F Plantefève G Voiriot G Badié J . Effect of hydrocortisone on 21-day mortality or respiratory support among critically ill patients with COVID-19: a randomized clinical trial. JAMA. (2020) 324:1298–306. 10.1001/jama.2020.16761 32876689 PMC7489432

[91] Li L Zhang W Hu Y Tong X Zheng S Yang J . Effect of convalescent plasma therapy on time to clinical improvement in patients with severe and life-threatening COVID-19: a randomized clinical trial. JAMA. (2020) 324:460–70. 10.1001/jama.2020.12607 32492084 PMC7270883

[92] Lopes RD Macedo AVS de Barros ESPGM Moll-Bernardes RJ Dos Santos TM Mazza L . Effect of discontinuing vs continuing angiotensin-converting enzyme inhibitors and angiotensin ii receptor blockers on days alive and out of the hospital in patients admitted with COVID-19: a randomized clinical trial. JAMA. (2021) 325:254–64. 10.1001/jama.2020.25864 33464336 PMC7816106

[93] López-Medina E López P Hurtado IC Dávalos DM Ramirez O Martínez E . Effect of ivermectin on time to resolution of symptoms among adults with mild COVID-19: a randomized clinical trial. JAMA. (2021) 325:1426–35. 10.1001/jama.2021.3071 33662102 PMC7934083

[94] Murai IH Fernandes AL Sales LP Pinto AJ Goessler KF Duran CSC . Effect of a single high dose of vitamin D3 on hospital length of stay in patients with moderate to severe COVID-19: a randomized clinical trial. JAMA. (2021) 325:1053–60. 10.1001/jama.2020.26848 33595634 PMC7890452

[95] Oldenburg CE Pinsky BA Brogdon J Chen C Ruder K Zhong L . Effect of oral azithromycin vs placebo on COVID-19 symptoms in outpatients with SARS-CoV-2 infection: a randomized clinical trial. JAMA. (2021) 326:490–8. 10.1001/jama.2021.11517 34269813 PMC8285753

[96] Self WH Semler MW Leither LM Casey JD Angus DC Brower RG . Effect of hydroxychloroquine on clinical status at 14 days in hospitalized patients with COVID-19: a randomized clinical trial. JAMA. (2020) 324:2165–76. 10.1001/jama.2020.22240 33165621 PMC7653542

[97] Spinner CD Gottlieb RL Criner GJ Arribas López JR Cattelan AM Soriano Viladomiu A . Effect of remdesivir vs standard care on clinical status at 11 days in patients with moderate COVID-19: a randomized clinical trial. JAMA. (2020) 324:1048–57. 10.1001/jama.2020.16349 32821939 PMC7442954

[98] Tomazini BM Maia IS Cavalcanti AB Berwanger O Rosa RG Veiga VC . Effect of dexamethasone on days alive and ventilator-free in patients with moderate or severe acute respiratory distress syndrome and COVID-19: The CoDEX randomized clinical trial. JAMA. (2020) 324:1307–16. 10.1001/jama.2020.17021 32876695 PMC7489411

[99] Cheng LL Guan WJ Duan CY Zhang NF Lei CL Hu Y . Effect of recombinant human granulocyte colony-stimulating factor for patients with coronavirus disease 2019 (COVID-19) and lymphopenia: a randomized clinical trial. JAMA Intern Med. (2021) 181:71–8. 10.1001/jamainternmed.2020.5503 32910179 PMC7489414

[100] Mariette X Hermine O Tharaux PL Resche-Rigon M Steg PG Porcher R . Effectiveness of tocilizumab in patients hospitalized with COVID-19: a follow-up of the CORIMUNO-TOCI-1 randomized clinical trial. JAMA Intern Med. (2021) 181:1241–43. 10.1001/jamainternmed.2021.2209 34028504 PMC8145157

[101] Salvarani C Dolci G Massari M Merlo DF Cavuto S Savoldi L . Effect of tocilizumab vs standard care on clinical worsening in patients hospitalized with COVID-19 pneumonia: a randomized clinical trial. JAMA Intern Med. (2021) 181:24–31. 10.1001/jamainternmed.2020.6615 33080005 PMC7577199

[102] Deftereos SG Giannopoulos G Vrachatis DA Siasos GD Giotaki SG Gargalianos P . Effect of colchicine vs standard care on cardiac and inflammatory biomarkers and clinical outcomes in patients hospitalized with coronavirus disease 2019: the GRECCO-19 randomized clinical trial. JAMA Netw Open. (2020) 3:e2013136. 10.1001/jamanetworkopen.2020.13136 32579195 PMC7315286

[103] Thomas S Patel D Bittel B Wolski K Wang Q Kumar A . Effect of high-dose zinc and ascorbic acid supplementation vs usual care on symptom length and reduction among ambulatory patients with SARS-CoV-2 infection: the COVID A to Z randomized clinical trial. JAMA Netw Open. (2021) 4:e210369. 10.1001/jamanetworkopen.2021.0369 33576820 PMC7881357

[104] Reis G Moreira Silva EAdS Medeiros Silva DC Thabane L Singh G Park JJH . Effect of early treatment with hydroxychloroquine or lopinavir and ritonavir on risk of hospitalization among patients with COVID-19: The TOGETHER randomized clinical trial. JAMA Network Open. (2021) 4:e216468. 10.1001/jamanetworkopen.2021.6468 33885775 PMC8063069

[105] Sadeghi A Ali Asgari A Norouzi A Kheiri Z Anushirvani A Montazeri M . Sofosbuvir and daclatasvir compared with standard of care in the treatment of patients admitted to hospital with moderate or severe coronavirus infection (COVID-19): a randomized controlled trial. J Antimicrobial Chemother. (2020) 75:3379–85. 10.1093/jac/dkaa334 32812039 PMC7454592

[106] Ravikirti Roy R Pattadar C Raj R Agarwal N Biswas B . Evaluation of ivermectin as a potential treatment for mild to moderate COVID-19: A Double-blind randomized placebo controlled trial in Eastern India. J Pharm Pharm Sci. (2021) 24:343–50. 10.18433/jpps32105 34265236

[107] Furtado RHM Berwanger O Fonseca HA Corrêa TD Ferraz LR Lapa MG . Azithromycin in addition to standard of care vs. standard of care alone in the treatment of patients admitted to the hospital with severe COVID-19 in Brazil (COALITION II): a randomised clinical trial. Lancet. (2020) 396:959–67. 10.1016/S0140-6736(20)31862-6 32896292 PMC7836431

[108] Lopes RD de Barros ESPGM Furtado RHM Macedo AVS Bronhara B Damiani LP . Therapeutic vs. prophylactic anticoagulation for patients admitted to hospital with COVID-19 and elevated D-dimer concentration (ACTION): an open-label, multicentre, randomised, controlled trial. Lancet. (2021) 397:2253–63. 10.1016/S0140-6736(21)01203-4 34097856 PMC8177770

[109] Wang Y Zhang D Du G Du R Zhao J Jin Y . Remdesivir in adults with severe COVID-19: a randomised, double-blind, placebo-controlled, multicentre trial. Lancet. (2020) 395:1569–78. 10.1016/S0140-6736(20)31022-9 32423584 PMC7190303

[110] Kosiborod MN Esterline R Furtado RHM Oscarsson J Gasparyan SB Koch GG . Dapagliflozin in patients with cardiometabolic risk factors hospitalised with COVID-19 (DARE-19): a randomised, double-blind, placebo-controlled, phase 3 trial. Lancet Diabetes Endocrinol. (2021) 9:586–94. 10.1016/S2213-8587(21)00180-7 34302745 PMC8294807

[111] Aman J Duijvelaar E Botros L Kianzad A Schippers JR Smeele PJ . Imatinib in patients with severe COVID-19: a randomised, double-blind, placebo-controlled, clinical trial. Lancet Respir Med. (2021) 9:957–68. 10.1016/S2213-2600(21)00237-X 34147142 PMC8232929

[112] Bauer A Schreinlechner M Sappler N Dolejsi T Tilg H Aulinger BA . Discontinuation vs. continuation of renin-angiotensin-system inhibitors in COVID-19 (ACEI-COVID): a prospective, parallel group, randomised, controlled, open-label trial. Lancet Respir Med. (2021) 9:863–72. 10.1016/S2213-2600(21)00214-9 34126053 PMC8195495

[113] Cohen JB Hanff TC William P Sweitzer N Rosado-Santander NR Medina C . Continuation vs. discontinuation of renin-angiotensin system inhibitors in patients admitted to hospital with COVID-19: a prospective, randomised, open-label trial. Lancet Respir Med. (2021) 9:275–84. 10.1016/S2213-2600(20)30558-0 33422263 PMC7832152

[114] Hinks TSC Cureton L Knight R Wang A Cane JL Barber VS . Azithromycin vs. standard care in patients with mild-to-moderate COVID-19 (ATOMIC2): an open-label, randomised trial. Lancet Respir Med. (2021). 10.1016/S2213-2600(21)00263-0. [Epub ahead of print]. 34252378 PMC8270523

[115] Lescure FX Honda H Fowler RA Lazar JS Shi G Wung P . Sarilumab in patients admitted to hospital with severe or critical COVID-19: a randomised, double-blind, placebo-controlled, phase 3 trial. Lancet Respir Med. (2021) 9:522–32. 10.1016/S2213-2600(21)00099-0 33676590 PMC8078879

[116] Monk PD Marsden RJ Tear VJ Brookes J Batten TN Mankowski M . Safety and efficacy of inhaled nebulised interferon beta-1a (SNG001) for treatment of SARS-CoV-2 infection: a randomised, double-blind, placebo-controlled, phase 2 trial. Lancet Respir Med. (2020) 9:196–206. 10.1016/S2213-2600(20)30511-7 33189161 PMC7836724

[117] Soin AS Kumar K Choudhary NS Sharma P Mehta Y Kataria S . Tocilizumab plus standard care vs. standard care in patients in India with moderate to severe COVID-19-associated cytokine release syndrome (COVINTOC): an open-label, multicentre, randomised, controlled, phase 3 trial. Lancet Respir Med. (2021) 9:511–21. 10.1016/S2213-2600(21)00081-3 33676589 PMC8078880

[118] Tardif JC Bouabdallaoui N L'Allier PL Gaudet D Shah B Pillinger MH . Colchicine for community-treated patients with COVID-19 (COLCORONA): a phase 3, randomised, double-blinded, adaptive, placebo-controlled, multicentre trial. Lancet Respir Med. (2021) 9:924–32. 10.1016/S2213-2600(21)00222-8 34051877 PMC8159193

[119] Li Y Xie Z Lin W Cai W Wen C Guan Y . Efficacy and safety of lopinavir/ritonavir or arbidol in adult patients with mild/moderate COVID-19: an exploratory randomized controlled trial. Med. (2020) 1:105–13. 10.1016/j.medj.2020.04.001 32838353 PMC7235585

[120] Gupta S Dixit PK Ghana P Abhisheka K Khurana H Jha VK . Open-label randomized control trial of hydroxychloroquine in patients with moderate to severe coronavirus disease 2019 infection. Med J Armed Forces India. (2021) 77:S305–11. 10.1016/j.mjafi.2021.02.007 34334898 PMC8313076

[121] Activ-Tico LY-CoV555 Study Group Lundgren JD Grund B Barkauskas CE Holland TL Gottlieb RL . A neutralizing monoclonal antibody for hospitalized patients with Covid-19. N Engl J Med. (2021) 384:905–14. 10.1056/NEJMoa2033130 33356051 PMC7781100

[122] Guimaraes PO Quirk D Furtado RH Maia LN Saraiva JF Antunes MO . Tofacitinib in patients hospitalized with covid-19 pneumonia. N Engl J Med. (2021) 385:406–15. 10.1056/NEJMoa2101643 34133856 PMC8220898

[123] Libster R Perez Marc G Wappner D Coviello S Bianchi A Braem V . Early high-titer plasma therapy to prevent severe Covid-19 in older adults. N Engl J Med. (2021) 384:610–8. 10.1056/NEJMoa2033700 33406353 PMC7793608

[124] Rosas IO Brau N Waters M Go RC Hunter BD Bhagani S . Tocilizumab in hospitalized patients with severe Covid-19 pneumonia. N Engl J Med. (2021) 384:1503–16. 10.1056/NEJMoa2028700 33631066 PMC7953459

[125] Salama C Han J Yau L Reiss WG Kramer B Neidhart JD . Tocilizumab in patients hospitalized with Covid-19 pneumonia. N Engl J Med. (2021) 384:20–30. 10.1056/NEJMoa2030340 33332779 PMC7781101

[126] Stone JH Frigault MJ Serling-Boyd NJ Fernandes AD Harvey L Foulkes AS . Efficacy of tocilizumab in patients hospitalized with Covid-19. N Engl J Med. (2020) 383:2333–44. 10.1056/NEJMoa2028836 33085857 PMC7646626

[127] Gharbharan A Jordans CCE GeurtsvanKessel C den Hollander JG Karim F Mollema FPN . Effects of potent neutralizing antibodies from convalescent plasma in patients hospitalized for severe SARS-CoV-2 infection. Nat Commun. (2021) 12:3189. 10.1038/s41467-021-23469-2 34045486 PMC8160346

[128] Cao B Wang Y Wen D Liu W Wang J Fan G . A trial of lopinavir–ritonavir in adults hospitalized with severe Covid-19. N Engl J Med. (2020) 382:1787–99. 10.1056/NEJMoa2001282 32187464 PMC7121492

[129] Cavalcanti AB Zampieri FG Rosa RG Azevedo LCP Veiga VC Avezum A . Hydroxychloroquine with or without Azithromycin in Mild-to-Moderate Covid-19. N Engl J Med. (2020) 383:2041–52. 10.1056/NEJMoa2019014 32706953 PMC7397242

[130] Kalil AC Patterson TF Mehta AK Tomashek KM Wolfe CR Ghazaryan V . Baricitinib plus remdesivir for hospitalized adults with covid-19. N Engl J Med. (2021) 384:795–807. 10.1056/NEJMoa2031994 33306283 PMC7745180

[131] Simonovich VA Burgos Pratx LD Scibona P Beruto MV Vallone MG Vázquez C . A Randomized Trial of Convalescent Plasma in Covid-19 Severe Pneumonia. N Engl J Med. (2020) 384:619–29. 10.1056/NEJMoa2031304 33232588 PMC7722692

[132] Ulrich RJ Troxel AB Carmody E Eapen J Backer M DeHovitz JA . Treating COVID-19 With Hydroxychloroquine (TEACH): A Multicenter, Double-Blind Randomized Controlled Trial in Hospitalized Patients. Open Forum Infect Dis. (2020) 7:ofaa446. 10.1093/ofid/ofaa446 33134417 PMC7543602

[133] Criner GJ Criner GJ Ahn MY Huhn G Subramanian A Lumbreras C . 561. Safety of remdesivir vs standard care in patients with moderate covid-19. Open Forum Infect Dis. (2020) 7:S345–6. 10.1093/ofid/ofaa439.755

[134] Sakoulas G Geriak M Kullar R Greenwood K Habib M Vyas A . 71 Use of intravenous immunoglobulin therapy reduces progression to mechanical ventilation in COVID-19 patients with moderate to severe hypoxia. Open Forum Infect Dis. (2020) 7:S166. 10.1093/ofid/ofaa439.381

[135] Galan LEB Santos NMD Asato MS Araujo JV de Lima Moreira A Araujo AMM . Phase 2 randomized study on chloroquine, hydroxychloroquine or ivermectin in hospitalized patients with severe manifestations of SARS-CoV-2 infection. Pathog Glob Health. (2021) 115:4,235–42. 10.1080/20477724.2021.1890887 33682640 PMC7938655

[136] Chen CP Lin YC Chen TC Tseng TY Wong HL Kuo CY . A multicenter, randomized, open-label, controlled trial to evaluate the efficacy and tolerability of hydroxychloroquine and a retrospective study in adult patients with mild to moderate coronavirus disease 2019 (COVID-19). PLoS ONE. (2020) 15:e0242763. 10.1371/journal.pone.0242763 33264337 PMC7710068

[137] Babalola OE Bode CO Ajayi AA Alakaloko FM Akase IE Otrofanowei E . Ivermectin shows clinical benefits in mild to moderate COVID19: a randomized controlled double-blind, dose-response study in Lagos. QJM: Int J Med. (2021). 10.1093/qjmed/hcab035. [Epub ahead of print]. 33599247 PMC7928689

[138] Tang X Feng YM Ni JX Zhang JY Liu LM Hu K . Early Use of corticosteroid may prolong SARS-CoV-2 shedding in non-intensive care unit patients with COVID-19 pneumonia: a multicenter, single-blind, randomized control trial. Respiration. (2021) 100:116–26. 10.1159/000512063 33486496 PMC7900459

[139] Alavi Darazam I Shokouhi S Pourhoseingholi MA Naghibi Irvani SS Mokhtari M Shabani M . Role of interferon therapy in severe COVID-19: the COVIFERON randomized controlled trial. Sci Rep. (2021) 11:8059. 10.1038/s41598-021-86859-y 33850184 PMC8044200

[140] AlQahtani M Abdulrahman A Almadani A Alali SY Al Zamrooni AM Hejab AH . Randomized controlled trial of convalescent plasma therapy against standard therapy in patients with severe COVID-19 disease. Sci Rep. (2021) 11:9927. 10.1038/s41598-021-89444-5 33976287 PMC8113529

[141] Dabbous HM El-Sayed MH El Assal G Elghazaly H Ebeid FFS Sherief AF . Safety and efficacy of favipiravir vs. hydroxychloroquine in management of COVID-19: A randomised controlled trial. Sci Rep. (2021) 11:7282. 10.1038/s41598-021-85227-0 33790308 PMC8012649

[142] Lakkireddy M Gadiga SG Malathi RD Karra ML Prasad Murthy ISSV . Impact of daily high dose oral vitamin D therapy on the inflammatory markers in patients with COVID 19 disease. Sci Rep. (2021) 11:10641. 10.1038/s41598-021-90189-4 34017029 PMC8138022

[143] Rashad A Mousa S Nafady-Hego H Nafady A Elgendy H. Short term survival of critically ill COVID-19 Egyptian patients on assisted ventilation treated by either Dexamethasone or Tocilizumab. Sci Rep. (2021) 11:8816. 10.1038/s41598-021-88086-x 33893337 PMC8065149

[144] Rashad A Nafady A Hassan MH Mansour H Taya U Bazeed SES . Therapeutic efficacy of macrolides in management of patients with mild COVID-19. Sci Rep. (2021) 11:16361. 10.1038/s41598-021-95900-z 34381155 PMC8357809

[145] Rea-Neto A Bernardelli RS Camara BMD Reese FB Queiroga MVO Oliveira MC. An open-label randomized controlled trial evaluating the efficacy of chloroquine/hydroxychloroquine in severe COVID-19 patients. Sci Rep. (2021) 11:9023. 10.1038/s41598-021-88509-9 33907251 PMC8079411

[146] Shi L Huang H Lu X Yan X Jiang X Xu R . Effect of human umbilical cord-derived mesenchymal stem cells on lung damage in severe COVID-19 patients: a randomized, double-blind, placebo-controlled phase 2 trial. Signal Transduct Target Ther. (2021) 6:58. 10.1038/s41392-021-00488-5 33568628 PMC7873662

[147] Shu L Niu C Li R Huang T Wang Y Huang M . Treatment of severe COVID-19 with human umbilical cord mesenchymal stem cells. Stem Cell Res Ther. (2020) 11:361. 10.1186/s13287-020-01875-5 32811531 PMC7432540

[148] Lanzoni G Linetsky E Correa D Messinger Cayetano S Alvarez RA Kouroupis D . Umbilical cord mesenchymal stem cells for COVID-19 acute respiratory distress syndrome: A double-blind, phase 1/2a, randomized controlled trial. Stem Cells Transl Med. (2021) 10:660–73. 10.1002/sctm.20-0472 33400390 PMC8046040

[149] Dilogo IH Aditianingsih D Sugiarto A Burhan E Damayanti T Sitompul PA . Umbilical cord mesenchymal stromal cells as critical COVID-19 adjuvant therapy: A randomized controlled trial. Stem Cells Transl Med. (2021) 10:1279–87. 10.1002/sctm.21-0046 34102020 PMC8242692

[150] Lemos ACB do Espirito Santo DA Salvetti MC Gilio RN Agra LB Pazin-Filho A . Therapeutic vs. prophylactic anticoagulation for severe COVID-19: A randomized phase II clinical trial (HESACOVID). Thromb Res. (2020) 196:359–66. 10.1016/j.thromres.2020.09.026 32977137 PMC7503069

[151] Gonzalez-Ochoa AJ Raffetto JD Hernández AG Zavala N Gutiérrez O Vargas A . Sulodexide in the treatment of patients with early stages of COVID-19: a randomized controlled trial. Thromb Haemost. (2021) 121:944–54. 10.1055/a-1414-5216 33677827

[152] Corral-Gudino L Bahamonde A Arnaiz-Revillas F Gomez-Barquero J Abadia-Otero J Garcia-Ibarbia C . Methylprednisolone in adults hospitalized with COVID-19 pneumonia: An open-label randomized trial (GLUCOCOVID). Wien Klin Wochenschr. (2021) 133:303–11. 10.1007/s00508-020-01805-8 33534047 PMC7854876

[153] Chen J Liu D Liu L Liu P Xu Q Xia L . A pilot study of hydroxychloroquine in treatment of patients with moderate COVID-19. Zhejiang Da Xue Xue Bao Yi Xue Ban. (2020) 49:215–9. 10.3785/j.issn.1008-9292.2020.03.03 32391667 PMC8800713

[154] Ansarin K Tolouian R Ardalan M Taghizadieh A Varshochi M Teimouri S . Effect of bromhexine on clinical outcomes and mortality in COVID-19 patients: A randomized clinical trial. Bioimpacts. (2020) 10:209–15. 10.34172/bi.2020.27 32983936 PMC7502909

[155] Zhao H Zhu Q Zhang C Li J Wei M Qin Y . Tocilizumab combined with favipiravir in the treatment of COVID-19: A multicenter trial in a small sample size. Biomed Pharmacother. (2021) 133:110825. 10.1016/j.biopha.2020.110825 33378989 PMC7524677

[156] Tolouian R Mulla ZD Jamaati H Babamahmoodi A Marjani M Eskandari R . Effect of bromhexine in hospitalized patients with COVID-19. J Investig Med. (2021). 10.1136/jim-2020-001747. [Epub ahead of print]. 33722999

[157] Ader F Peiffer-Smadja N Poissy J Bouscambert-Duchamp M Belhadi D Diallo A . An open-label randomized controlled trial of the effect of lopinavir/ritonavir, lopinavir/ritonavir plus IFN-beta-1a and hydroxychloroquine in hospitalized patients with COVID-19. Clin Microbiol Infect. (2021). 10.1016/j.cmi.2021.05.020. [Epub ahead of print]. 34048876 PMC8149166

[158] Ivashchenko AA Dmitriev KA Vostokova NV Azarova VN Blinow AA Egorova AN . AVIFAVIR for treatment of patients with moderate COVID-19: interim results of a phase II/III multicenter randomized clinical trial. Clin Infect Dis. (2021) 73:531–4. 10.1093/cid/ciaa1176 32770240 PMC7454388

[159] Cadegiani FA McCoy J Gustavo Wambier C Vaño-Galván S Shapiro J Tosti A . Proxalutamide significantly accelerates viral clearance and reduces time to clinical remission in patients with mild to moderate COVID-19: results from a randomized, double-blinded, placebo-controlled trial. Cureus. (2021) 13:e13492. 10.7759/cureus.13492 33633920 PMC7899267

[160] Kamran SM Moeed HA Mirza ZE Naseem A Azam R Ullah N . Clearing the fog: is hydroxychloroquine effective in reducing coronavirus disease-2019 progression? A randomized controlled trial. Cureus. (2021) 13:e14186. 10.7759/cureus.14186 33936897 PMC8083993

[161] Blum VF Cimerman S Hunter JR Tierno P Lacerda A Soeiro A . Nitazoxanide superiority to placebo to treat moderate COVID-19 - A Pilot prove of concept randomized double-blind clinical trial. EClinicalMedicine. (2021) 37:100981. 10.1016/j.eclinm.2021.100981 34222847 PMC8235996

[162] Chaccour C Casellas A Blanco-Di Matteo A Pineda I Fernandez-Montero A Ruiz-Castillo P . The effect of early treatment with ivermectin on viral load, symptoms and humoral response in patients with non-severe COVID-19: A pilot, double-blind, placebo-controlled, randomized clinical trial. EClinicalMedicine. (2021) 32:100720. 10.1016/j.eclinm.2020.100720 33495752 PMC7816625

[163] Rocco PRM Silva PL Cruz FF Junior MACM Tierno PFGMM Moura MA . Early use of nitazoxanide in mild Covid-19 disease: randomised, placebo-controlled trial. Eur Respir J. (2020) 58:2003725. 10.1101/2020.10.21.20217208 33361100 PMC7758778

[164] Aref ZF Bazeed S Hassan MH Hassan AS Rashad A Hassan RG . Clinical, biochemical and molecular evaluations of ivermectin mucoadhesive nanosuspension nasal spray in reducing upper respiratory symptoms of mild COVID-19. Int J Nanomedicine. (2021) 16:4063–72. 10.2147/IJN.S313093 34163159 PMC8215847

[165] Ahmed S Karim MM Ross AG Hossain MS Clemens JD Sumiya MK . A five day course of ivermectin for the treatment of COVID-19 may reduce the duration of illness. Int J Infect Dis. (2021) 103:214–6. 10.1016/j.ijid.2020.11.191 33278625 PMC7709596

[166] Mahmud R Rahman MM Alam I Ahmed KGU Kabir A Sayeed S . Ivermectin in combination with doxycycline for treating COVID-19 symptoms: a randomized trial. J Int Med Res. (2021) 49:3000605211013550. 10.1177/03000605211013550 33983065 PMC8127799

[167] Kim MS An MH Kim WJ Hwang TH. Comparative efficacy and safety of pharmacological interventions for the treatment of COVID-19: A systematic review and network meta-analysis. PLoS Med. (2020) 17:e1003501. 10.1371/journal.pmed.1003501 33378357 PMC7794037

[168] Hariyanto TI Hardyson W Kurniawan A. Efficacy and safety of tocilizumab for coronavirus disease 2019 (Covid-19) Patients: a systematic review and meta-analysis. Drug Res (Stuttg). (2021) 71:265–74. 10.1055/a-1336-2371 33401328

[169] Boregowda U Perisetti A Nanjappa A Gajendran M Kutti Sridharan G Goyal H. Addition of tocilizumab to the standard of care reduces mortality in severe COVID-19: a systematic review and meta-analysis. Front Med (Lausanne). (2020) 7:586221. 10.3389/fmed.2020.586221 33123544 PMC7566918

[170] Kotak S Khatri M Malik M Malik M Hassan W Amjad A . Use of tocilizumab in COVID-19: a systematic review and meta-analysis of current evidence. Cureus. (2020) 12:e10869. 10.7759/cureus.10869 33178522 PMC7652362

[171] Ivan Hariyanto T Kurniawan A. Tocilizumab administration is associated with the reduction in biomarkers of coronavirus disease 2019 infection. J Med Virol. (2021) 93:1832–6. 10.1002/jmv.26698 33241872 PMC7753293

[172] Hariyanto TI Halim DA Rosalind J Gunawan C Kurniawan A. Ivermectin and outcomes from Covid-19 pneumonia: A systematic review and meta-analysis of randomized clinical trial studies. Rev Med Virol. (2021). 10.1002/rmv.2265. [Epub ahead of print].

[173] Padhy BM Mohanty RR Das S Meher BR. Therapeutic potential of ivermectin as add on treatment in COVID 19: A systematic review and meta-analysis: Ivermectin in COVID-19: A meta-analysis. J Pharm Pharm Sci. (2020) 23:462–9. 10.18433/jpps31457 33227231

[174] Hariyanto TI Halim DA Jodhinata C Yanto TA Kurniawan A. Colchicine treatment can improve outcomes of coronavirus disease 2019 (COVID-19): A systematic review and meta-analysis. Clin Exp Pharmacol Physiol. (2021) 48:823–30. 10.1111/1440-1681.13488 33719081 PMC8250626

[175] Hariyanto TI Kwenandar F Japar KV Damay V Kurniawan A. The Effectiveness and safety of remdesivir for the treatment of patients with COVID-19: a systematic review and meta-analysis. Anti-Infect Agents. (2021) 19:333–40. 10.2174/2211352518999201009124433

[176] Elsawah HK Elsokary MA Abdallah MS ElShafie AH. Efficacy and safety of remdesivir in hospitalized Covid-19 patients: Systematic review and meta-analysis including network meta-analysis. Rev Med Virol. (2021) 31:e2187. 10.1002/rmv.2187 33128490

[177] Guyatt GH Oxman AD Vist G Kunz R Brozek J Alonso-Coello P . GRADE guidelines: 4. Rating the quality of evidence–study limitations (risk of bias). J Clin Epidemiol. (2011) 64:407–15. 10.1016/j.jclinepi.2010.07.017 21247734

[178] Guyatt GH Oxman AD Montori V Vist G Kunz R Brozek J . GRADE guidelines: 5. Rating the quality of evidence–publication bias. J Clin Epidemiol. (2011) 64:1277–82. 10.1016/j.jclinepi.2011.01.011 21802904

[179] De Crescenzo F Amato L Cruciani F Moynihan LP D'Alò GL Vecchi S . Comparative effectiveness of pharmacological interventions for Covid-19: a systematic review and network meta-analysis. Front Pharmacol. (2021) 12:649472. 10.3389/fphar.2021.649472 34012398 PMC8126885

