# Supplementary material for: Efficacy of COVID-19 Treatments: A Bayesian Network Meta-Analysis of Randomized Controlled Trials
Source: Front Public Health. 2021 Sep 28;9:729559. doi: 10.3389/fpubh.2021.729559 (PMC8506153; doi:10.3389/fpubh.2021.729559)
Supplement: Supplementary file 1 [file Data_Sheet_1.docx]

***Supplementary Materials of “Efficacy of COVID-19 Treatments: A Bayesian Network Meta-Analysis of Randomized Controlled Trials”***

Contents

[**Supplementary Methods** 1](#_Toc81009381)

[**Model structure of network meta-analysis** 1](#_Toc81009382)

[**Supplementary Tables** 2](#_Toc81009383)

[**Table S1. Detailed search strategy** 2](#_Toc81009384)

[**Table S2. Treatments included and their classifications based on Anatomical Therapeutic Chemical Classification System with Defined Daily Doses (ATC/DDD).** 3](#_Toc81009385)

[**Table S3. Detailed trial characteristics** 12](#_Toc81009386)

[**Table S4. Detailed patient characteristics for included studies.** 24](#_Toc81009387)

[**Table S5. Evaluation of risk of bias (mortality) (1: low; 2: probably low; 3: probably high, 4: high).** 56](#_Toc81009388)

[**Table S6. Evaluation of risk of bias (mechanical ventilation) (1: low; 2: probably low; 3: probably high, 4: high).** 62](#_Toc81009389)

[**Table S7. Evaluation of risk of bias (discharge) (1: low; 2: probably low; 3: probably high, 4: high).** 66](#_Toc81009390)

[**Table S8. Evaluation of risk of bias (viral clearance) (1: low; 2: probably low; 3: probably high, 4: high).** 68](#_Toc81009391)

[**Table S9. Network meta-analysis results of the primary analysis (log odds ratio and corresponding 95% equal-tailed Bayesian credible intervals) (mortality).** 69](#_Toc81009392)

[**Table S10. Network meta-analysis results of the primary analysis (log odds ratio, log OR and corresponding 95% equal-tailed Bayesian credible intervals) (mechanical ventilation).** 114](#_Toc81009393)

[**Table S11. Network meta-analysis results of the primary analysis (log odds ratio, log OR and corresponding 95% equal-tailed Bayesian credible intervals) (discharge).** 141](#_Toc81009394)

[**Table S12. Network meta-analysis results of the primary analysis (log odds ratio, log OR and corresponding 95% equal-tailed Bayesian credible intervals) (viral clearance).** 154](#_Toc81009395)

[**Table S13. Sensitivity analysis: fixed-effects model versus fixed-effects model which treated trials with multiple publications as multiple separated trials versus random-effects model (mortality).** 162](#_Toc81009396)

[**Table S14. Sensitivity analysis: fixed-effects model versus fixed-effects model which treated trials with multiple publications as multiple separated trials versus random-effects model (mechanical ventilation).** 168](#_Toc81009397)

[**Table S15. Sensitivity analysis: fixed-effects model versus fixed-effects model which treated trials with multiple publications as multiple separated trials versus random-effects model (discharge).** 173](#_Toc81009398)

[**Table S16. Sensitivity analysis: fixed-effects model versus random-effects model (viral clearance).** 176](#_Toc81009399)

[**Supplementary Figures** 178](#_Toc81009400)

[**Figure S1. Bayesian hierarchical framework for trial in the network meta-analysis,** 178](#_Toc81009401)

[**Figure S2. Network plot for mortality.** 179](#_Toc81009402)

[**Figure S3. Network plot for mechanical ventilation.** 180](#_Toc81009403)

[**Figure S4. Network plot for discharge.** 181](#_Toc81009404)

[**Figure S5. Network plot for viral clearance.** 182](#_Toc81009405)

[**Figure S6. Subgroup analysis for mild/moderate COVID-19 patients: mortality under treatments compared with the standard of care (SOC).** 183](#_Toc81009406)

[**Figure S7. Subgroup analysis for severe COVID-19 patients: mortality under treatments compared with the standard of care (SOC).** 184](#_Toc81009407)

[**Figure S8. Subgroup analysis for mild/moderate COVID-19 patients: mechanical ventilation under treatments compared with the standard of care (SOC).** 185](#_Toc81009408)

[**Figure S9. Subgroup analysis for severe COVID-19 patients: mechanical ventilation under treatments compared with the standard of care (SOC).** 186](#_Toc81009409)

[**Figure S10. Subgroup analysis for mild/moderate COVID-19 patients: discharge under treatments compared with the standard of care (SOC).** 187](#_Toc81009410)

[**Figure S11. Subgroup analysis for severe COVID-19 patients: discharge under treatments compared with the standard of care (SOC).** 188](#_Toc81009411)

[**Figure S12. Subgroup analysis for mild/moderate COVID-19 patients: viral clearance under treatments compared with the standard of care (SOC).** 189](#_Toc81009412)

[**Figure S13. Subgroup analysis for severe COVID-19 patients: viral clearance under treatments compared with the standard of care (SOC).** 190](#_Toc81009413)

[**Figure S14. Exploratory analysis for both peer-reviewed and preprint studies: mortality under treatments compared with the standard of care (SOC).** 191](#_Toc81009414)

[**Figure S15. Exploratory analysis for both peer-reviewed and preprint studies: mechanical ventilation under treatments compared with the standard of care (SOC).** 192](#_Toc81009415)

[**Figure S16. Exploratory analysis for both peer-reviewed and preprint studies: discharge under treatments compared with the standard of care (SOC).** 193](#_Toc81009416)

[**Figure S17. Exploratory analysis for both peer-reviewed and preprint studies: viral clearance under treatments compared with the standard of care (SOC).** 194](#_Toc81009417)

# **Supplementary Methods**

## **Model structure of network meta-analysis**

For the $i$-th trial, suppose the sample size is $n_{i,x}$ and the number of events is $y_{i,x}$ for treatment $x$. We assume a binomial model,

$$y_{i,x}\sim\mathrm{Binomial}\left( p_{i,x}, n_{i,x} \right),$$

where $p_{i,x}$ is the probability of the event of interest.

Taking arm $b_{i}$ as the baseline treatment for trial $i$, under the logit link function, our fixed-effects model is formulated as

$$\mathrm{logit}\left( p_{i,x} \right)=\mu_{i}+d_{b_{i},x},$$

$$d_{b_{i},x}=d_{0,x}-d_{0,b_{i}},$$

where $\mu_{i}$ is the effect size of baseline treatment $b_{i}$, $d_{b_{i},x}$ is the relative effect of treatment $x$ compared with $b_{i}$ in the $i$-th trial, $d_{0,x}$ is the relative treatment effect of $x$ compared with the standard of care (SOC) labelled by 0. Under a Bayesian hierarchical model, we assume the relative treatment effect as

$$d_{0,x}\sim N\left( \theta_{c_{x}}, \sigma^{2} \right),$$

$$d_{0,b_{i}}=0 \mathrm{if} b_{i}=0,$$

$$d_{0,b_{i}}\sim N\left( \theta_{c_{b_{i}}}, \sigma^{2} \right) \mathrm{if} b_{i}\neq0,$$

where $c_{x}$ is the class that treatment $x$ belongs to, and $\theta_{c_{x}}$ is the relative treatment effect of class $c_{x}$ compared with SOC.

For the random-effects model, we specify a Bayesian hierarchical structure as follows,

$$\mathrm{logit}\left( p_{i,x} \right)=\mu_{i}+\delta_{i,b_{i},x},$$

$$\delta_{i,b_{i},x}\sim N\left( d_{b_{i},x},\tau^{2} \right),$$

$$d_{b_{i},x}=d_{0,x}-d_{0,b_{i}},$$

$$d_{0,x}\sim N\left( \theta_{c_{x}}, \sigma^{2} \right),$$

$$d_{0,b_{i}}=0 \mathrm{if} b_{i}=0,$$

$$d_{0,b_{i}}\sim N\left( \theta_{c_{b_{i}}}, \sigma^{2} \right) \mathrm{if} b_{i}\neq0,$$

Our prior distributions are given as follows:

- $\mu_{i}\sim N\left( 0, {10}^{2} \right), i=1,\ldots,n,$ where $n$ is the number of trials.
- $\theta_{c}\sim N\left( 0, {10}^{2} \right), c=1,\ldots,m,$ where $m$ is the number of classes.
- $\sigma^{2}\sim\mathrm{InverseGamma}\left( 0.01,0.01 \right).$
- $\tau^{2}\sim\mathrm{InverseGamma}\left( 0.01,0.01 \right)$

# **Supplementary Tables**

## **Table S1. Detailed search strategy**

| **Database** | **Search strategy** |
| --- | --- |
| WHO COVID-19 Global Research Database | (tw:(randomly)) OR (tw:(randomisation)) OR (tw:(randomization)) OR (tw:(randomized)) OR (tw:(randomised)) |

## **Table S2. Treatments included and their classifications based on Anatomical Therapeutic Chemical Classification System with Defined Daily Doses (ATC/DDD).**

| **Treatment** | **Classification (ATC/DDD First Level)** | **ATC.Code** | **ATC/DDD**  **First Level** |
| --- | --- | --- | --- |
| Aceis/Arbs | Agents Acting On The Renin-Angiotensin System | C09AA + C09CA | Agents Acting On The Renin-Angiotensin System |
| Adalimumab | Immunosuppressants | L04AB04 | Antineoplastic And Immunomodulating Agents |
| Alpha-Lipoic Acid | Other Alimentary Tract And Metabolism Products | A16AX01 | Alimentary Tract And Metabolism |
| Amlodipine | Calcium Channel Blockers | C08CA01 | Cardiovascular System |
| Ammonium Chloride | Blood Substitutes And Perfusion Solutions | B05XA04 | Blood And Blood Forming Organs |
| Anakinra | Immunosuppressants | L04AC03 | Antineoplastic And Immunomodulating Agents |
| Arbidol | Antivirals For Systemic Use | J05AX13 | Antiinfectives For Systemic Use |
| Aspirin | Antithrombotic Agents | B01AC06 | Blood And Blood Forming Organs |
| Auxora | Calcium Channel Blockers | NA | Cardiovascular System |
| Azithromycin | Antibacterials For Systemic Use | J01FA10 | Antiinfectives For Systemic Use |
| Baloxavir Marboxil | Antivirals For Systemic Use | J05AX25 | Antiinfectives For Systemic Use |
| Bamlanivimab | Antineoplastic Agents | L01XC (Monoclonal antibodies) | Antineoplastic And Immunomodulating Agents |
| Bamlanivimab + Etesevimab | Antineoplastic Agents | L01XC (Monoclonal antibodies) | Antineoplastic And Immunomodulating Agents |
| Baricitinib | Immunosuppressants | L04AA37 | Antineoplastic And Immunomodulating Agents |
| Baricitinib + Remdesivir | Immunosuppressants + Antivirals For Systemic Use | L04AA37 + NA | Antineoplastic And Immunomodulating Agents + Antiinfectives For Systemic Use |
| Bromhexine | Cough And Cold Preparations | R05CB02 | Respiratory System |
| Budesonide | Corticosteroids, Dermatological Preparations | D07AC09 | Dermatologicals |
| C21 | Agents Acting On The Renin-Angiotensin System | NA | Cardiovascular System |
| Camostat Mesilate | Antihemorrhagics | B02AB04 | Blood And Blood Forming Organs |
| Canakinumab | Immunosuppressants | L04AC08 | Antineoplastic And Immunomodulating Agents |
| Chloroquine | Antiprotozoals | P01BA01 | Antiparasitic Products, Insecticides And Repellents |
| Ciclesonide | Nasal Preparations | R01AD13 | Respiratory System |
| Clarithromycin | Antibacterials For Systemic Use | J01FA09 | Antiinfectives For Systemic Use |
| Colchicine | Antigout Preparations | M04AC01 | Musculo-Skeletal System |
| Colchicine + Rosuvastatin | Antigout Preparations + Lipid Modifying Agents | M04AC01 + C10AA07 | Musculo-Skeletal System + Cardiovascular System |
| Convalescent Plasma | Blood Substitutes And Perfusion Solutions | B05AX03 (Blood plasma) | Blood And Blood Forming Organs |
| Cytokine Adsorption | Cytokine Adsorption | NA | Cytokine Adsorption |
| Dapagliflozin | Drugs Used In Diabetes | A10BK01 | Alimentary Tract And Metabolism |
| Dexamethasone | Corticosteroids, Dermatological Preparations | D07AB19 | Dermatologicals |
| Doxycycline | Antibacterials For Systemic Use | J01AA02 | Antiinfectives For Systemic Use |
| Dutasteride | Urologicals | G04CB02 | Genito Urinary System And Sex Hormones |
| Emtricitabine/Tenofovir | Antivirals For Systemic Use | J05AR17 | Antiinfectives For Systemic Use |
| Emtricitabine/Tenofovir + Colchicine + Rosuvastatin | Antivirals For Systemic Use + Antigout Preparations + Lipid Modifying Agents | J05AR17 + M04AC01 + C10AA07 | Antiinfectives For Systemic Use+ Musculo-Skeletal System + Cardiovascular System |
| Favipiravir | Antivirals For Systemic Use | J05AX27 | Antiinfectives For Systemic Use |
| Favipiravir + Interferon Beta | Antivirals For Systemic Use + Immunostimulants | J05AX27 + L03AB04 | Antiinfectives For Systemic Use + Antineoplastic And Immunomodulating Agents |
| Febuxostat | Antigout Preparations | M04AA03 | Musculo-Skeletal System |
| Finasteride | Urologicals | G04CB01 | Genito Urinary System And Sex Hormones |
| Fluvoxamine | Psychoanaleptics | N06AB08 | Nervous System |
| Hydrocortisone | Corticosteroids, Dermatological Preparations | D07AA02 | Dermatologicals |
| Hydroxychloroquine | Antiprotozoals | P01BA02 | Antiparasitic Products, Insecticides And Repellents |
| Hydroxychloroquine + Azithromycin | Antiprotozoals + Antibacterials For Systemic Use | P01BA02 + J01FA10 | Antiparasitic Products, Insecticides And Repellents + Antiinfectives For Systemic Use |
| Hydroxychloroquine + Darunavir | Antiprotozoals + Antivirals For Systemic Use | P01BA02 + J05AE10 | Antiparasitic Products, Insecticides And Repellents + Antiinfectives For Systemic Use |
| Hydroxychloroquine + Favipiravir | Antiprotozoals + Antivirals For Systemic Use | P01BA02 + J05AX27 | Antiparasitic Products, Insecticides And Repellents + Antiinfectives For Systemic Use |
| Hydroxychloroquine + Lopinavir/Ritonavir | Antiprotozoals + Antivirals For Systemic Use | P01BA02 + J05AR10 | Antiparasitic Products, Insecticides And Repellents + Antiinfectives For Systemic Use |
| Hydroxychloroquine + Ribavirin | Antiprotozoals + Antivirals For Systemic Use | P01BA02 + J05AP01 | Antiparasitic Products, Insecticides And Repellents + Antiinfectives For Systemic Use |
| Hydroxychloroquine + Zinc | Antiprotozoals + Mineral Supplements | P01BA02 + A12CB | Antiparasitic Products, Insecticides And Repellents + Alimentary Tract And Metabolism |
| Ifx | Antineoplastic Agents | L01XC (Monoclonal antibodies) | Antineoplastic And Immunomodulating Agents |
| Imatinib | Antineoplastic Agents | L01EA01 | Antineoplastic And Immunomodulating Agents |
| Indomethacin | Antiinflammatory And Antirheumatic Products | M01AB01 | Musculo-Skeletal System |
| Infliximab | Immunosuppressants | L04AB02 | Antineoplastic And Immunomodulating Agents |
| Inm005 | Antineoplastic Agents | L01XC (Monoclonal antibodies) | Antineoplastic And Immunomodulating Agents |
| Interferon Alpha | Immunostimulants | L03AB04, L03AB05 | Antineoplastic And Immunomodulating Agents |
| Interferon Alpha + Interferon Gamma | Immunostimulants | L03AB04 + L03AB03 | Antineoplastic And Immunomodulating Agents |
| Interferon Beta | Immunostimulants | L03AB07, L03AB08 | Antineoplastic And Immunomodulating Agents |
| Interferon Beta + Hydroxychloroquine + Lopinavir/Ritonavir | Immunostimulants + Antiprotozoals + Antivirals For Systemic Use | L03AB07, L03AB08 + P01BA02 + J05AR10 | Antineoplastic And Immunomodulating Agents + Antiparasitic Products, Insecticides And Repellents + Antiinfectives For Systemic Use |
| Interferon Beta + Hydroxychloroquine + Lopinavir/Ritonavir + Umifenovir | Immunostimulants + Antiprotozoals + Antivirals For Systemic Use | L03AB07, L03AB08 + P01BA02 + J05AR10 + J05AX13 | Antineoplastic And Immunomodulating Agents + Antiparasitic Products, Insecticides And Repellents + Antiinfectives For Systemic Use |
| Interferon Beta + Lopinavir/Ritonavir + Ribavirin | Antivirals For Systemic Use + Immunostimulants | J05AP01 + J05AR10 + L03AB07 | Antiinfectives For Systemic Use + Antineoplastic And Immunomodulating Agents |
| Interferon Gamma | Immunostimulants | L03AB03 | Antineoplastic And Immunomodulating Agents |
| Intravenous Immunoglobulin | Immune Sera And Immunoglobulins | J06B | Antiinfectives For Systemic Use |
| Itolizumab | Antineoplastic Agents | L01XC (Monoclonal antibodies) | Antineoplastic And Immunomodulating Agents |
| Ivermectin | Anthelmintics | P02CF01 | Antiparasitic Products, Insecticides And Repellents |
| Ivermectin + Doxycycline | Anthelmintics + Antibacterials For Systemic Use | P02CF01 + J01AA02 | Antiparasitic Products, Insecticides And Repellents + Antiinfectives For Systemic Use |
| Leflunomide + Interferon Alpha | Immunosuppressants + Immunostimulants | L04AA13 + L03AB04 | Antineoplastic And Immunomodulating Agents + Antineoplastic And Immunomodulating Agents |
| Lenzilumab | Antineoplastic Agents | L01XC (Monoclonal antibodies) | Antineoplastic And Immunomodulating Agents |
| Lopinavir/Ritonavir | Antivirals For Systemic Use | J05AR10 | Antiinfectives For Systemic Use |
| Lopinavir/Ritonavir + Interferon Alpha | Antivirals For Systemic Use + Immunostimulants | J05AR10 + L03AB04 | Antiinfectives For Systemic Use + Antineoplastic And Immunomodulating Agents |
| Lopinavir/Ritonavir + Novaferon | Antivirals For Systemic Use + Immunostimulants | J05AR10 + NA | Antiinfectives For Systemic Use + Antineoplastic And Immunomodulating Agents |
| Lopinavir/Ritonavir + Ribavirin | Antivirals For Systemic Use | J05AR10 + J05AP01 | Antiinfectives For Systemic Use |
| Losartan | Agents Acting On The Renin-Angiotensin System | C09CA01 | Cardiovascular System |
| Mavrilimumab | Antineoplastic Agents | L01XC (Monoclonal antibodies) | Antineoplastic And Immunomodulating Agents |
| Melatonin | Psycholeptics | N05CH01 | Nervous System |
| Mesenchymal Stem Cells | Blood Substitutes And Perfusion Solutions | B05AX04 | Blood And Blood Forming Organs |
| Methylene Blue | Antidotes (V03ab) | V03AB17 | Various |
| Methylprednisolone | Corticosteroids, Dermatological Preparations | D07AA01 | Dermatologicals |
| Methylprednisolone + Tacrolimus | Corticosteroids, Dermatological Preparations + Immunosuppressants | D07AA01 + L04AD02 | Dermatologicals + Antineoplastic And Immunomodulating Agents |
| Molnupiravir | Antivirals For Systemic Use | J05 | Antiinfectives For Systemic Use |
| Mycobacterium W | Mycobacterium W | NA | Mycobacterium W |
| N-Acetylcysteine | Cough And Cold Preparations | R05CB01 | Respiratory System |
| Namilumab | Antineoplastic Agents | L01XC (Monoclonal antibodies) | Antineoplastic And Immunomodulating Agents |
| Niclosamide | Anthelmintics | P02DA01 | Antiparasitic Products, Insecticides And Repellents |
| Nitazoxanide | Antiprotozoals | P01AX11 | Antiparasitic Products, Insecticides And Repellents |
| Nitric Oxide | Other Respiratory System Products | R07AX01 | Respiratory System |
| Novaferon | Immunostimulants | NA | Antineoplastic And Immunomodulating Agents |
| Omega-3 Supplementation | Lipid Modifying Agents | C10A (LIPID MODIFYING AGENTS, PLAIN) | Cardiovascular System |
| Otilimab | Antineoplastic Agents | L01XC (Monoclonal antibodies) | Antineoplastic And Immunomodulating Agents |
| Paracetamol | Analgesics | N02BE01 | Nervous System |
| Pnb001 | Endocrine Therapy | L02B (HORMONE ANTAGONISTS AND RELATED AGENTS) | Antineoplastic And Immunomodulating Agents |
| Probiotic | Microorganisms | NA | Microorganisms |
| Progesterone | Sex Hormones And Modulators Of The Genital System | G03DA04 | Genito Urinary System And Sex Hormones |
| Proxalutamide | Endocrine Therapy | L02BB (Anti-androgens) | Antineoplastic And Immunomodulating Agents |
| Pyridostigmine | Other Nervous System Drugs | N07AA02 | Nervous System |
| Quercetin | Vasoprotectives | C05CA | Cardiovascular System |
| Recombinant Human Gcsf | Immunostimulants | L03AA | Antineoplastic And Immunomodulating Agents |
| Regen-Cov | Antineoplastic Agents | L01XC (Monoclonal antibodies) | Antineoplastic And Immunomodulating Agents |
| Remdesivir | Antivirals For Systemic Use | NA | Antiinfectives For Systemic Use |
| Ribavirin + Interferon Alpha | Antivirals For Systemic Use + Immunostimulants | J05AP01 + L03AB04 | Antiinfectives For Systemic Use + Antineoplastic And Immunomodulating Agents |
| Ribavirin + Lopinavir/Ritonavir + Interferon Alpha | Antivirals For Systemic Use + Immunostimulants | J05AP01 + J05AR10 + L03AB04 | Antiinfectives For Systemic Use + Antiinfectives For Systemic Use + Antineoplastic And Immunomodulating Agents |
| Ruxolitinib | Antineoplastic Agents | L01XE18 | Antineoplastic And Immunomodulating Agents |
| Sarilumab | Immunosuppressants | L04AC14 | Antineoplastic And Immunomodulating Agents |
| Secukinumab | Immunosuppressants | L04AC10 | Antineoplastic And Immunomodulating Agents |
| Sitagliptin | Drugs Used In Diabetes | A10BH01 | Alimentary Tract And Metabolism |
| Soc | Soc |  | Soc |
| Sofosbuvir + Daclatasvir | Antivirals For Systemic Use | J05AP08 + J05AP07 | Antiinfectives For Systemic Use |
| Sofosbuvir + Daclatasvir + Ribavirin | Antivirals For Systemic Use | J05AP08 + J05AP07 + J05AP01 | Antiinfectives For Systemic Use |
| Sofosbuvir + Ledipasvir | Antivirals For Systemic Use | J05AP51 | Antiinfectives For Systemic Use |
| Sofosbuvir + Ravidasvir | Antivirals For Systemic Use | J05AP08 + NA | Antiinfectives For Systemic Use |
| Sofosbuvir + Velpatasvir | Antivirals For Systemic Use | J05AP55 | Antiinfectives For Systemic Use |
| Sotrovimab | Antineoplastic Agents | L01XC (Monoclonal antibodies) | Antineoplastic And Immunomodulating Agents |
| Spironolactone | Diuretics | C03DA01 | Cardiovascular System |
| Spironolactone + Sitagliptin | Diuretics + Drugs Used In Diabetes | C03DA01 + A10BH01 | Cardiovascular System + Alimentary Tract And Metabolism |
| Sulodexide | Antithrombotic Agents | B01AB11 | Blood And Blood Forming Organs |
| Td-0903 | Antineoplastic Agents | L01EJ (Janus-associated kinase (JAK) inhibitors) | Antineoplastic And Immunomodulating Agents |
| Telmisartan | Agents Acting On The Renin-Angiotensin System | C09CA07 | Cardiovascular System |
| Therapeutic Anticoagulation | Antithrombotic Agents | B01AB01 | Blood And Blood Forming Organs |
| Tocilizumab | Immunosuppressants | L04AC07 | Antineoplastic And Immunomodulating Agents |
| Tocilizumab + Favipiravir | Immunosuppressants + Antivirals For Systemic Use | L04AC07 + J05AX27 | Antineoplastic And Immunomodulating Agents + Antiinfectives For Systemic Use |
| Tofacitinib | Immunosuppressants | L04AA29 | Antineoplastic And Immunomodulating Agents |
| Triazavirin | Antivirals For Systemic Use | NA | Antiinfectives For Systemic Use |
| Vitamin C | Vitamins | A11GA01 | Alimentary Tract And Metabolism |
| Vitamin D3 | Vitamins | A11CC | Alimentary Tract And Metabolism |
| Xav-19 | Antineoplastic Agents | L01XC (Monoclonal antibodies) | Antineoplastic And Immunomodulating Agents |
| Zinc | Mineral Supplements | A12CB | Alimentary Tract And Metabolism |
| Zinc + Vitamin C | Mineral Supplements + Vitamins | A12CB + A11GA01 | Alimentary Tract And Metabolism + Alimentary Tract And Metabolism |

## **Table S3. Detailed trial characteristics**

| **Registration number** | **Publication status (Published 1 or preprinted 0)** | **Study status (Completed; Interim analysis; Terminated early)** | **Randomizaton (1=double-blinded; 2=single-blined;3=open-label)** | **isMultiarm** | **Control type (1: compared with SOC; 2: compared with other interventions; 3: compared to both other interventions and SOC)** | **Design (1=parallel group 2=cluster randomized)** | **Funding resources (1=Industry 2=Government 3=Institutional 4=Not-for-profit foundation 0=None)** | **Intervention** | **Geographies** | **No. of patients** |
| --- | --- | --- | --- | --- | --- | --- | --- | --- | --- | --- |
| NCT04384380 | 1 | Completed | 3 | 0 | 1 | 1 | 2 | Hydroxychloroquine vs SOC | China | 33 |
| NCT04261517 | 1 | Completed | 3 | 0 | 1 | 1 | 2 | Hydroxychloroquine vs SOC | China | 30 |
| jRCTs041190120 | 1 | Completed | 3 | 0 | 2 | 1 | 1 | Early favipiravir vs late favipiravir | Japan | 88 |
| IRCT20100228003449N28 | 1 | Completed | 3 | 0 | 1 | 1 | 0 | Interferon beta-1a vs SOC | Iran | 81 |
| NCT04383535 | 1 | Completed | 1 | 0 | 1 | 1 | 3 | Convalescent plasma vs SOC | Europe | 333 |
| ChiCTR2000029853 | 1 | Completed | 3 | 0 | 1 | 1 | NA | Azvudine vs SOC | China | 20 |
| ChiCTR2000029308 | 1 | Completed | 3 | 0 | 1 | 1 | 2 | lopinavir–ritonavir vs SOC | China | 199 |
| ChiCTR2000030262 | 1 | Completed | 3 | 0 | 1 | 1 | 2 | Interferon kappa + TFF2 vs SOC | China | 80 |
| NCT04333420 | 1 | Completed | 3 | 0 | 1 | 1 | 1 | IFX-1 vs SOC | Netherland | 30 |
| NCT04345614 | 1 | Completed | 3 | 0 | 1 | 1 | 1 | Auxora vs SOC | USA | 30 |
| NCT04434248 | 1 | Interim analysis | 3 | 0 | 1 | 1 | 2 | Favipiravir vs SOC | Russia | 60 |
| NCT04321278 | 1 | Completed | 3 | 0 | 1 | 1 | 3 | Azithromycin vs SOC | Brazil | 397 |
| ChiCTR2000029544 | 1 | Completed | 3 | 1 | 3 | 1 | NA | Baloxavir marboxil vs favipiravir vs SOC | China | 29 |
| CTRI202004024775 | 1 | Completed | 3 | 0 | 1 | 1 | 2 | Convalescent plasma vs SOC | India | 464 |
| NCT04381936_Dex | 1 | Completed | 3 | 0 | 1 | 1 | 2 | Dexamethasone vs SOC | UK | 6424 |
| RBR8969zg | 1 | Completed | 1 | 0 | 1 | 1 | 2 | N-acetylcysteine vs SOC | Brazil | 135 |
| NCT04366908 | 1 | Completed | 3 | 0 | 1 | 1 | NAA | Calcifediol vs SOC | Spain | 76 |
| NCT04326790 | 1 | Completed | 3 | 0 | 1 | 1 | 3 | Colchicine vs SOC | Greece | 105 |
| ChiCTR2000029757 | 1 | Completed | 3 | 0 | 1 | 1 | 2 | Convalescent plasma vs SOC | China | 103 |
| NCT04327401 | 1 | Completed | 3 | 0 | 1 | 1 | NA | Dexamethasone vs SOC | Brazil | 299 |
| NCT042323527 | 1 | Completed | 1 | 0 | 2 | 1 | 2 | High-dose chloroquine vs low-dose chloroquine | Brazil | 81 |
| NCT02517489 | 1 | Terminated early | 1 | 0 | 1 | 1 | 2 | Hydrocortisone vs SOC | France | 149 |
| NCT02735707_hyd | 1 | Terminated early | 3 | 0 | 1 | 1 | 2,3,4 | Hydrocortisone vs SOC | Multisite | 384 |
| NCT04381936_Hydro | 1 | Completed | 3 | 0 | 1 | 1 | 2 | Hydroxychloroquine vs SOC | UK | 4716 |
| NCT04332991 | 1 | Completed | 1 | 0 | 1 | 1 | 2 | Hydroxychloroquine vs SOC | USA | 479 |
| ChiCTR2000030007 | 1 | Completed | 3 | 0 | 1 | 1 | 1, 2 | Recombinant human granulocyte colony-stimulating factor vs SOC | China | 200 |
| NCT04292730 | 1 | Completed | 3 | 0 | 1 | 1 | 1 | Remdesivir vs SOC | Multisite | 584 |
| NCT04346355 | 1 | Completed | 3 | 0 | 1 | 1 | 2 | Tocilizumab vs SOC | Italy | 126 |
| NCT04331808 | 1 | Completed | 3 | 0 | 1 | 1 | 2 | Tocilizumab vs SOC | France | 130 |
| NCT04449718 | 1 | Completed | 1 | 0 | 1 | 1 | 2 | Vitamin D3 vs SOC | Brazil | 237 |
| NCT04252885 | 1 | Completed | 2 | 1 | 3 | 1 | 2 | Lopinavir/ritonavir vs Arbidol vs SOC | China | 86 |
| ChiCTR20000300001 | 1 | Terminated early | 1 | 0 | 1 | 1 | NA | Triazavirin vs SOC | China | 52 |
| NCT04356937 | 1 | Completed | 3 | 0 | 1 | 1 | 1 | Tocilizumab vs SOC | USA | 243 |
| IRCT20151227025726N20 | 1 | Completed | 3 | 0 | 1 | 1 | NA | Intravenous Immunoglobulin vs SOC | Iran | 84 |
| IRCT20200328046886N1 | 1 | Completed | 3 | 0 | 1 | 1 | 3 | Sofosbuvir + Daclatasvir + Ribavirin vs SOC | Iran | 48 |
| IRCT2019072704434N1 | 1 | Completed | 3 | 0 | 2 | 1 | 3 | Febuxostat vs Hydroxychloroquine | Iran | 54 |
| NCT04342663 | 1 | Completed | 1 | 0 | 1 | 1 | 3 | Fluvoxamine vs SOC | USA | 152 |
| EudraCT202000193437 | 1 | Completed | 3 | 0 | 1 | 1 | 0 | Methylprednisolone vs SOC | Spain | 64 |
| NCT04304053 | 1 | Completed | 3 | 1 | 3 | 2 | 1,2,3 | Hydroxychloroquine vs Hydroxychloroquine + Darunavir vs SOC in combination with DRVc | Spain | 307 |
| NCT04308668 | 1 | Completed | 1 | 0 | 1 | 1 | Private donors | Hydroxychloroquine vs SOC | Multisite | 423 |
| ChiCTR2000029868 | 1 | Completed | 3 | 0 | 1 | 1 | 2 | Hydroxychloroquine vs SOC | China | 150 |
| NCT04353336 | 1 | Completed | 3 | 0 | 1 | 1 | NA | Hydroxychloroquine vs SOC | Egypt | 175 |
| NCT04329832 | 1 | Terminated early | 3 | 0 | 2 | 1 | 2,3 | Hydroxychloroquine vs Azithromycin | USA | 85 |
| NCT04322123 | 1 | Completed | 3 | 1 | 3 | 1 | 3 | Hydroxychloroquine + Azithromycin vs Hydroxychloroquine vs SOC | Brazil | 665 |
| IRCT20100228003449N27 | 1 | Completed | 3 | 0 | 1 | 1 | 0 | Interferon β-1b vs SOC | Iran | 66 |
| NCT04381936_Lopi | 1 | Completed | 3 | 0 | 1 | 1 | 2 | Lopinavir/ritonavir vs SOC | UK | 5040 |
| NCT04343729 | 1 | Completed | 1 | 0 | 1 | 1 | 2, 3 | Methylprednisolone vs SOC | Brazil | 393 |
| ChiCTR2000029387 | 1 | Completed | 3 | 1 | 2 | 1 | 0 | Ribavirin + Interferon-Alpha vs Lopinavir/Ritonavir + Interferon-Alpha vs Ribavirin + Lopinavir/Ritonavir + Interferon-Alpha | China | 101 |
| NCT04331899 | 1 | Completed | 2 | 0 | 1 | 1 | 1, 3 | Interferon lambda vs SOC | USA | 120 |
| NCT04354259 | 1 | Completed | 1 | 0 | 1 | 1 | 3 | Interferon lambda vs SOC | Canada | 60 |
| Oman | 1 | Completed | 3 | 0 | 2 | 1 | 0 | Favipiravir + Interferon beta vs Hydroxychloroquine | Oman | 89 |
| NCT04356534 | 1 | Completed | 3 | 0 | 1 | 1 | 2, 3 | Convalescent plasma vs SOC | Bahrain | 40 |
| NCT04349592 | 1 | Completed | 1 | 1 | 3 | 1 | 2 | Hydroxychloroquine vs Hydroxychloroquine + Azithromycin vs SOC | Qatar | 456 |
| NCT04280705 | 1 | Completed | 1 | 0 | 1 | 1 | 2 | Remdesivir vs SOC | Multisite | 1062 |
| NCT04257656 | 1 | Completed | 1 | 0 | 1 | 1 | 2 | Remdesivir vs SOC | China | 236 |
| NCT04315948 | 1 | Interim analysis | 3 | 1 | 3 | 1 | 3 | Hydroxychloroquine vs Interferon alpha-2b vs Lopinavir/ritonavir vs Remdesivir vs SOC | Multisite | 11266 |
| JAK | 1 | Completed | 2 | 0 | 1 | 1 | 2, 3 | Ruxolitinib vs SOC | China | 41 |
| ChiCTR2000029496 | 1 | Completed | 3 | 1 | 2 | 1 | 2 | Novaferon vs Lopinavir/ritonavir vs Lopinavir/ritonavir + Novaferon | China | 89 |
| IRCT20200128046294N2 | 1 | Completed | 3 | 0 | 1 | 1 | 3 | Sofosbuvir + Daclatasvir vs SOC | Iran | 66 |
| RBR949z6v | 1 | Completed | 3 | 0 | 2 | 1 | 0 | Prophylactic anticoagulation vs Therapeutic enoxaparin | Brazil | 20 |
| ChiCTR2000030058 | 1 | Completed | 3 | 0 | 2 | 1 | 2 | Leflunomide + IFN alpha-2a vs IFN alpha-2a | China | 48 |
| NCT04276688 | 1 | Completed | 3 | 0 | 2 | 1 | 4 | Interferon beta-1b + Lopinavir–ritonavir + Ribavirin vs Lopinavir–ritonavir | China | 127 |
| NCT04447534 | 1 | Completed | 1 | 0 | 2 | 2 | NA | Zinc + HCQ vs HCQ | Egypt | 191 |
| NCT04385095 | 1 | Completed | 1 | 0 | 1 | 1 | 1 | Interferon beta vs SOC | UK | 98 |
| NCT04372186 | 1 | Completed | 1 | 0 | 1 | 1 | 1 | Tocilizumab vs SOC | Multisite | 377 |
| IRCT20200501047259N1 | 1 | Completed | 1 | 0 | 1 | 1 | 3 | Intravenous immunoglobulin vs SOC | Iran | 59 |
| NCT04552483 | 1 | Completed | 1 | 0 | 1 | 1 | 2 | Nitazoxanide vs SOC | Brazil | 392 |
| NCT04325893 | 1 | Completed | 1 | 0 | 1 | 1 | 2 | Hydroxychloroquine vs SOC | France | 250 |
| NCT04288102 | 1 | Completed | 1 | 0 | 1 | 1 | 2 | Mesenchymal stem cells vs SOC | China | 100 |
| Umi | 1 | Completed | 3 | 0 | 1 | 1 | 0 | umifenovir vs SOC | Kyrgyzstan | 30 |
| IRCT202003117046797N4 | 1 | Completed | 3 | 0 | 1 | 1 | 3 | Bromhexine vs SOC | Iran | 78 |
| NCT04369742 | 1 | Completed | 1 | 0 | 1 | 1 | 2, 3 | Hydroxychloroquine vs SOC | USA | 128 |
| NCT04375098 | 1 | Completed | 3 | 0 | 2 | 1 | 4 | Early plasma vs deferred plasma | Chile | 58 |
| RPCEC00000317-En | 1 | Completed | 3 | 0 | 1 | 1 | NA | Intravenous CIGB-325 vs SOC | Cuba | 20 |
| NCT04273763 | 1 | Completed | 3 | 0 | 1 | 1 | 2 | Bromhexine vs SOC | China | 18 |
| NCT04320615 | 1 | Completed | 1 | 0 | 1 | 1 | 2 | Tocilizumab vs SOC | Multisite | 438 |
| Linazi | 1 | Completed | 3 | 0 | 2 | 1 | 0 | LINCOCIN® vs AZITRO® | Turkey | 24 |
| NCT04292899 | 1 | Completed | 3 | 0 | 2 | 1 | 1 | Remdesivir for 5 day vs Remdesivir for 10 days | Multisite | 397 |
| Iver_Ahmed | 1 | Completed | 1 | 1 | 3 | 1 | 1 | Ivermectin vs Ivermectin + Doxycycline vs SOC | Bangladesh | 72 |
| NCT04381936_Azi | 1 | Completed | 3 | 0 | 1 | 1 | 2,3 | Azithromycin vs SOC | UK | 7763 |
| NCT04401579 | 1 | Completed | 1 | 0 | 2 | 1 | 2 | Baricitinib + Remdesivir vs Remdesivir | Multisite | 1033 |
| IRCT20180725040596N2 | 1 | Completed | 3 | 0 | 2 | 1 | 3 | Hydroxychloroquine + Lopinavir/Ritonavir vs Hydroxychloroquine + Arbidol | Iran | 100 |
| ISRCTN59048638 | 1 | Completed | 1 | 0 | 1 | 1 | 1 | Sulodexide vs SOC | Mexico | 243 |
| NCT04420247 | 1 | Completed | 3 | 0 | 1 | 1 | 3 | Hydroxychloroquine or Chloroquine vs SOC | Brazil | 105 |
| IRCT20200403046926N1 | 1 | Completed | 1 | 0 | 2 | 1 |  | Sofosbuvir/Daclatasvir + Hydroxychloroquine vs Hydroxychloroquine | Iran | 55 |
| NCT04355728 | 1 | Completed | 1 | 0 | 1 | 1 | 2 | Mesenchymal stem cells vs SOC | USA | 24 |
| NCT04484493 | 1 | Completed | 3 | 0 | 1 | 1 | 0 | Mometasone vs SOC | Egypt | 100 |
| NCT04365153 | 1 | Interim analysis | 1 | 1 | 3 | 1 | 1 | Canakinumab vs SOC | USA | 45 |
| CTRI-2020-08-027225 | 1 | Completed | 1 | 0 | 1 | 1 | NA | Ivermectin vs SOC | India | 112 |
| NCT04315948_Discovery | 1 | Completed | 3 | 1 | 3 | 1 | 2 | SOC vs Lopinavir/ritonavir vs Lopinavir/ritonavir plus IFN-beta-1a vs Hydroxychloroquine vs Remdesivir | France | 583 |
| NCT04479163 | 1 | Completed | 1 | 0 | 1 | 1 | 4 | convalescent plasma vs SOC | Argentina | 160 |
| ChiCTR2000029765 | 1 | Completed | 3 | 0 | 1 | 1 | 2,3 | tocilizumab vs SOC | China | 65 |
| NCT04390022 | 1 | Completed | 1 | 0 | 1 | 1 | 4 | ivermectin vs SOC | Spanish | 24 |
| NCT04403685 | 1 | Completed | 3 | 0 | 1 | 1 | 3 | tocilizumab vs SOC | Brazil | 129 |
| CTRI-2020-04-024729 | 1 | Completed | 2 | 0 | 1 | 1 | 2,3 | Chloroquine vs SOC | India | 60 |
| NCT04273321 | 1 | Terminated early | 2 | 0 | 1 | 1 | 2 | Methylprednisolone vs SOC | China | 86 |
| NCT04322682 | 1 | Terminated early | 1 | 0 | 1 | 1 | 2,3,4 | Colchicine vs placebo | Canada | 4488 |
| NCT04351295 | 1 | Completed | 3 | 0 | 2 | 1 | 3 | Chloroquine vs Favipiravir | Egypt | 92 |
| NCT04341584 | 1 | Completed | 3 | 0 | 1 | 1 | 2,4 | Anakinra vs SOC | France | 114 |
| NCT04327388 | 1 | Completed | 1 | 1 | 1 | 1 | 1 | Sarilumab 400 mg vs Sarilumab 200 mg vs placebo | Multisite | 416 |
| IRCT20100228003449N29 | 1 | Completed | 3 | 0 | 1 | 1 | 0 | Sofosbuvir + Ledipasvir vs SOC | Iran | 82 |
| CTRI-2020-05-025114 | 1 | Completed | 3 | 0 | 1 | 1 | 1 | Favipiravir vs SOC | India | 147 |
| NCT04381936_Toci | 1 | Completed | 3 | 0 | 1 | 1 | 2 | Tocilizumab vs SOC | UK | 4116 |
| NCT04321616 | 1 | Completed | 3 | 1 | 3 | 1 | 2 | Remdesivir vs Hydroxycholoroquine vs SOC | Norway | 181 |
| NCT04416399 | 1 | Terminated early | 3 | 0 | 1 | 1 | 1,2 | Budesonide vs SOC | UK | 139 |
| IRCT20200411047025N1 | 1 | Completed | 3 | 0 | 1 | 1 | 3 | Vitamin C IV vs SOC | Iran | 60 |
| CTRI-2020-06-026222 | 1 | Completed | 3 | 0 | 1 | 1 | 1 | Immunoglobulin vs SOC | India | 100 |
| NCT04342728 | 1 | Terminated early | 3 | 1 | 3 | 1 | 3 | Ascorbic acid vs Zinc vs Ascorbic acid + Zinc vs usual care | USA | 214 |
| INA-TX6YYSS | 1 | Completed | 1 | 1 | 3 | 1 | 2 | Azithromycin (500 mg once a day) vs 200/50 mg lopinavir/ritonavir twice a day and 500 mg azithromycin once a day vs 200/50 mg lopinavir/ritonavir and 100 mg doxycycline twice a day vs 200 mg hydroxychloroquine twice a day and 500 mg azithromycin once a day vs 400/100 mg lopinavir/ritonavir twice a day and 500 mg azithromycin once a day vs 400/100 mg lopinavir/ritonavir and 100 mg doxycycline twice a day | Indonesia | 751 |
| NCT04364893 | 1 | Completed | 3 | 0 | 1 | 1 | 3 | Discontinuing vs Continuing Angiotensin-Converting Enzyme Inhibitors and Angiotensin II Receptor Blockers | Brazil | 659 |
| ISRCTN40302986 | 1 | Completed | 1 | 1 | 1 | 1 | 0 | Low-dose Ivermectin vs high-dose Ivermectin 12mg vs Lopinavir / ritonavir | Nigeria | 62 |
| NCT04365127 | 1 | Completed | 3 | 0 | 1 | 1 | 3 | Progesterone vs SOC | USA | 40 |
| ChiCTR2000029638 | 1 | Completed | 2 |  | 2 | 1 | 3 | Nebulized rSIFN-co vs Nebulized interferon-alpha | China | 94 |
| NCT04329923 | 1 | Interim analysis | 1 | 0 | 1 | 1 | 4 | Hydroxychloroquine vs SOC | USA | 34 |
| NCT04354428 | 1 | Terminated early | 1 | 1 | 3 | 1 | 2,3,4 | Hydroxychloroquine vs Hydroxychloroquine + Azithromycin vs SOC | USA | 231 |
| Brazilian 4.173.074 | 1 | Completed | 1 | 0 | 1 | 1 | 0 | Proxalutamide vs SOC | Brazil | 236 |
| EAT-DUTA AndroCoV | 1 | Completed | 1 | 0 | 1 | 1 | 0 | Dutasteride vs SOC | Brazil | 87 |
| NCT04405843 | 1 | Completed | 1 | 0 | 1 | 1 | 3 | Ivermectin vs SOC | Colombia | 398 |
| ISRCTN86534580_Azi | 1 | Completed | 3 | 0 | 1 | 1 | 2 | Azithromycin vs SOC | UK | 1388 |
| NCT04338009 | 1 | Completed | 3 | 0 | 2 | 1 | 3,4 | Continue vs discontinue renin-angiotensin system inhibitor therapy | Multisite | 152 |
| CTRI-2020-05-025369 | 1 | Completed | 3 | 0 | 1 | 1 | 3 | Tocilizumab vs SOC | India | 179 |
| NCT04381936_CP | 1 | Completed | 3 | 0 | 1 | 1 | 2 | Convalescent plasma vs SOC | UK | 11558 |
| RBR-8h7q82 | 1 | Completed | 1 | 1 | 2 | 1 | NA | Chloroquine vs Hydroxychloroquine vs Ivermectin | Brazil | 168 |
| ACTRN12620000454976 | 1 | Completed | 1 | 0 | 1 | 1 | NA | Zinc vs SOC | Australia | 33 |
| NCT02735707, NCT04505774, NCT04359277, NCT04372589_Critical | 1 | Terminated early | 3 | 0 | 2 | 1 | 2,3,4 | Therapeutic anticoagulation vs usual care pharmacological thromboprophylaxis | Multisite | 1103 |
| NCT04359810 | 1 | Completed | 1 | 0 | 1 | 1 | 3 | Convalescent plasma vs SOC | Multisite | 223 |
| NCT04427501_P2 | 1 | Completed | 1 | 1 | 3 | 1 | 1 | Bamlanivimab vs Bamlanivimab + etesevimab vs SOC | USA | 577 |
| CTRI-2020-06-026087 | 1 | Completed | 3 | 0 | 1 | 1 | 3 | PEG IFN-α2b vs SOC | India | 40 |
| IRCT20200505047318N1 | 1 | Completed | 1 | 0 | 1 | 1 | 3 | Finasteride vs SOC | Iran | 80 |
| IRCT20180802040678N4 | 1 | Completed |  | 0 | 2 | 1 | 3 | Losartan vs Amlodipine | Iran | 80 |
| NCT04431466 | 1 | Completed | 3 | 1 | 3 | 1 | 3 | Ivermectin vs SOC | Brazil | 31 |
| Tolouian_Bromhexine | 1 | Completed | 3 | 0 | 1 | 1 | 0 | Bromhexine vs SOC | Iran | 100 |
| NCT04486508 | 1 | Completed | 3 | 0 | 2 | 1 | 3 | Intermediate-dose vs standard-dose prophylactic anticoagulation | Iran | 562 |
| IRCT20200318046812N1 | 1 | Completed | 3 | 0 | 2 | 1 | 3 | Favipiravir vs Lopinavir/Ritonavir | Iran | 373 |
| NCT04399980 | 1 | Terminated early | 1 | 0 | 1 | 1 | 1 | Mavrilimumab vs SOC | USA | 40 |
| IRCT20190810044500N7 | 1 | Completed | 1 | 0 | 1 | 1 | 3 | Levamisole vs SOC | Iran | 50 |
| Mahajan_Remdesivir | 1 | Completed | 3 | 0 | 1 | 1 | NA | Remdesivir vs SOC | India | 70 |
| IRCT20151226025699N3 | 1 | Completed | 1 | 0 | 1 | 1 | 3 | Omega-3 vs SOC | Iran | 101 |
| NCT04349241 | 1 | Completed | 3 | 0 | 2 | 1 | 1 | Favipiravir vs Hydroxychloroquine | Egypt | 100 |
| NCT04569825 | 1 | Terminated early | 1 | 0 | 1 | 1 | 0 | Betamethasone sodium phosphate drops vs SOC | Iran | 276 |
| ISRCTN86534580_Budesonide | 1 | Terminated early | 3 | 0 | 1 | 1 | 2 | Ihaled budesonide vs SOC | UK | 1959 |
| IRCT20200310046736N1 | 1 | Completed | 2 | 0 | 1 | 1 | 3 | Convalescent plasma vs SOC | Iran | 60 |
| NCT04494984 | 1 | Completed | 1 | 0 | 1 | 1 | 1,2 | INM005 vs SOC | Argentina | 241 |
| NCT04128579 | 1 | Completed | 3 | 0 | 1 | 1 | 1 | Itolizumab vs SOC | India | 30 |
| IRCT20200204046369N1 | 1 | Completed | 1 | 0 | 2 | 1 | 0 | Methylprednisolone vs Dexamethasone | Iran | 86 |
| NCT04343768 | 1 | Completed | 3 | 1 | 1 | 1 | 0 | IFNβ1a vs IFNβ1b vs SOC | Iran | 60 |
| Sakoulas_IVIG | 1 | Completed | 3 | 0 | 1 | 1 | 3 | Itravenous immunoglobulin s SOC | USA | 33 |
| NCT04252664 | 1 | Completed | 3 | 1 | 1 | 1 | 3 | Remdesivir vs SOC | Multisite | 584 |
| NCT04453384 | 1 | Completed | 1 | 0 | 1 | 1 | 3 | XAV-19 vs SOC | France | 17 |
| IRCT20151227025726N17 | 1 | Interim analysis | 3 | 0 | 1 | 1 | 0 | Dexamethasone vs SOC | Iran | 50 |
| NCT04491994 | 1 | Completed | 3 | 0 | 1 | 1 | 3,4 | Hydroxychloroquine vs SOC | Pakistan | 500 |
| NCT04344535 | 1 | Terminated early | 1 | 0 | 1 | 1 | 3 | Convalescent Plasma vs Standard Plasma | USA | 74 |
| NCT04403100 | 1 | Terminated early | 1 | 1 | 3 | 1 | 4 | Hydroxychloroquine vs Lopinavir-ritonavir vs SOC | Brazil | 685 |
| NCT04381962 | 1 | Completed | 3 | 0 | 1 | 1 | 1,3 | Aithromycin vs SOC | UK | 295 |
| NCT04321096 | 1 | Completed | 1 | 0 | 1 | 1 | 4 | Camostat mesilate vs SOC | Multisite | 205 |
| NCT04333589 | 1 | Completed | 3 | 0 | 1 | 1 | 2 | Favipiravir vs SOC | China | 55 |
| NCT04646109 | 1 | Completed | 2 | 0 | 1 | 1 | 3 | Ivermectin vs SOC | Turkey | 60 |
| NCT02735707_toci | 1 | Completed | 3 | 1 | 3 | 1 | 2,3,4 | Tocilizumab vs Sarilumab vs SOC | Multisite | 865 |
| NCT04519385 | 1 | Completed | 3 | 0 | 2 | 1 | 3 | Dexamethasone vs Tocilizumab | Egypt | 109 |
| NCT04310228 | 1 | Completed | 3 | 1 | 2 | 1 | 2,3 | Tocilizumab + Favipiravir vs Favipiravir vs Tocilizumab | China | 26 |
| NCT04501978 | 1 | Terminated early | 1 | 0 | 1 | 1 | 1,2,3,4 | LY-CoV555 vs SOC | Multisite | 314 |
| ChiCTR2000031494 | 1 | Completed | 3 | 0 | 1 | 1 | 2 | Mesenchymal stem cells vs SOC | China | 41 |
| RBR-6sx3sz | 1 | Interim analysis | 1 | 0 | 1 | 1 | 2 | Hydrogen Peroxide vs SOC | Brazil | 35 |
| NCT04340557 | 1 | Completed | 3 | 0 | 1 | 1 | 4 | Losartan vs SOC | USA | 31 |
| NCT04523831 | 1 | Completed | 1 | 0 | 1 | 1 | 0 | Ivermectin + Doxycycline vs SOC | Bangladesh | 400 |
| NCT04323228 | 1 | Completed | 1 | 0 | 1 | 1 | 2 | Antioxidant supplements vs SOC | Saudi Arabia | 44 |
| NCT04549376 | 1 | Completed | 3 | 0 | 1 | 1 | 3 | Povidone-iodine vs SOC | Bangladesh | 189 |
| CTRI202012030083 | 1 | Completed | 3 | 0 | 1 | 1 | 1 | Vitamin D vs SOC | India | 87 |
| NCT04370288 | 1 | Completed | 3 | 0 | 1 | 1 | 3 | Methylene blue vs SOC | Iran | 80 |
| NCT04352959 | 1 | Completed | 1 | 0 | 1 | 1 | 1,3 | Beta-cyclodextrin and citrox vs SOC | France | 176 |
| NCT04347174 | 1 | Completed | 1 | 0 | 1 | 1 | 2,3 | Mycobacterium w (0.3 mL per day) vs SOC | India | 42 |
| IRCT20130812014333N145 | 1 | Completed | 3 | 0 | 1 | 1 | 3 | Sofosbuvir/velpatasvir vs SOC | Iran | 80 |
| ChiCTR2000033627 | 1 | Completed | 3 | 0 | 1 | 1 | 0 | Ivermectin vs SOC | Lebanon | 100 |
| IRCT20111224008507N3 | 1 | Completed | 1 | 0 | 1 | 1 | NA | Ivermectin vs SOC | Iran | 69 |
| NCT04342182 | 1 | Terminated early | 3 | 0 | 1 | 1 | 1,3 | Convalescent plasma vs SOC | Netherlands | 86 |
| NCT04403555 | 1 | Completed | 3 | 0 | 1 | 1 | 3 | Ivermectin vs SOC | Egypt | 164 |
| NCT04394377 | 1 | Completed | 3 | 0 | 2 | 1 | 1,3 | Therapeutic vs Prophylactic anticoagulation | Brazil | 615 |
| VD_Elamir | 1 | Completed | 3 | 0 | 1 | 1 | NA | Vitamin D3 vs SOC | Israel | 50 |
| NCT04457609 | 1 | Completed | 1 | 0 | 1 | 1 | 2 | Mesenchymal stromal cells vs SOC | Indonesia | 40 |
| NCT04521309 | 1 | Completed | 2 | 0 | 1 | 1 | 2 | IVIG vs SOC | Pakistan | 50 |
| NCT04330586 | 1 | Completed | 3 | 0 | 1 | 1 | 2,3 | Ciclesonide vs SOC | South Korea | 61 |
| NCT04622891 | 1 | Completed | 2 | 1 | 3 | 1 | 3 | Azithromycin vs Clarithromycin vs SOC | Egypt | 305 |
| IRCT20200504047298N3 | 1 | Completed | 1 | 0 | 1 | 1 | 3 | Ammonium Chloride vs SOC | Iran | 120 |
| ISRCTN21085622 | 1 | Completed | 3 | 1 | 3 | 1 | 1 | Sofosbuvir + Daclatasvir vs Sofosbuvir + Ravidasvir vs SOC | Egypt | 120 |
| NCT04542941 | 1 | Completed | 3 | 0 | 1 | 1 | 2 | Convalescent plasma vs SOC | Uganda | 136 |
| NCT04446429 | 1 | Completed | 1 | 0 | 1 | 1 | 1 | Proxalutamide vs SOC | Brazil | 268 |
| NCT04372589, NCT04505774, NCT02735707, NCT04359277_Noncritical | 1 | Terminated early | 3 | 0 | 1 | 1 | 2,3,4 | therapeutic-dose anticoagulation with heparin vs usual-care pharmacologic thromboprophylaxis | Multisite | 2231 |
| NCT04353596 | 1 | Completed | 3 | 0 | 2 | 1 | 2,3 | Discontinuation vs continuation of renin-angiotensin-system inhibitors | Multisite | 204 |
| NCT04362813 | 1 | Completed | 1 | 0 | 1 | 1 | 1 | Canakinumab vs SOC | Multisite | 454 |
| CTRI-2020-04-024479 | 1 | Completed | 3 | 0 | 1 | 1 | NA | Hydroxychloroquine vs SOC | India | 110 |
| NCT04324528 | 1 | Completed | 3 | 0 | 1 | 1 | 0 | Cytokine adsorption vs SOC | Germany | 34 |
| NCT04521400 | 1 | Completed | 3 | 0 | 2 | 1 | 3 | High-dose Interferon beta 1-a vs low-dose Infereron beta 1-a | Iran | 168 |
| ISRCTN86534580_Doxy | 1 | Terminated early | 3 | 0 | 1 | 1 | 2,3 | Doxycycline vs SOC | UK | 1792 |
| NCT04392973 | 1 | Terminated early | 3 | 0 | 1 | 1 | 3 | Hydroxychloroquine + favipiravir vs SOC | Saudi Arabia | 254 |
| NCT04498936 | 1 | Completed | 3 | 1 | 3 | 1 | 3 | Sofosbuvir + Ledipasvir vs Nitazoxanide vs SOC | Egypt | 190 |
| NCT04350593 | 1 | Completed | 1 | 0 | 1 | 1 | 1 | Dapagliflozin vs SOC | Multisite | 1250 |
| NCT04332107 | 1 | Terminated early | 1 | 0 | 1 | 1 | 4 | Azithromycin vs SOC | USA | 263 |
| IRCT20151113025025N3 | 1 | Completed | 1 | 0 | 1 | 1 | 3 | ACEis/ARBs vs SOC | Iran | 64 |
| IRCT20200408046987N1 | 1 | Completed | 1 | 1 | 3 | 1 | NA | hydroxychloroquine vs hydroxychloroquine + placebo vs single dose ivermectin (200 μg/kg) vs three low interval doses of ivermectin (200, 200, 200 μg/kg) vs single dose ivermectin (400 μg/kg) vs three high interval doses of ivermectin (400, 200, 200 μg/kg). | Iran | 180 |
| NCT04427501_P3 | 1 | Completed | 1 | 0 | 1 | 1 | 1 | Bamlanivimab + Etesevimab vs SOC | USA | 1035 |
| NCT04350684 | 1 | Completed | 3 | 0 | 2 | 1 | 0 | Interferon beta + Hydroxychloroquine + Lopinavir/Ritonavir + Umifenovir vs Interferon beta + Hydroxychloroquine + Lopinavir/Ritonavir | Iran | 101 |
| NCT02735707_LPH | 1 | Terminated early | 3 | 1 | 3 | 1 | 2,3,4 | lopinavir-ritonavir vs hydroxychloroquine vs lopinavir-ritonavir + hydroxychloroquine vs SOC | Multisite | 694 |
| NCT04547660 | 1 | Completed | 3 | 0 | 1 | 1 | 2,3 | Convalescent Plasma vs SOC | Brazil | 160 |
| IRCT20151227025726N23 | 1 | Completed | 3 | 0 | 1 | 1 | 3 | Adalimumab vs SOC | Iran | 68 |
| NCT04360824 | 1 | Completed | 3 | 0 | 2 | 1 | 2 | Standard prophylactic versus intermediate dose enoxaparin | USA | 173 |
| NCT04409522 | 1 | Completed | 1 | 0 | 1 | 1 | 0 | Melatonin vs SOC | Iran | 44 |
| NCT04529525 | 1 | Completed | 1 | 0 | 1 | 1 | 0 | Ivermectin vs SOC | Argentina | 501 |
| NCT04341038 | 1 | Terminated early | 3 | 0 | 1 | 1 | 2,3 | Methylprednisolone + Tacrolimus vs SOC | Spain | 55 |
| NCT04861298 | 1 | Interim analysis | 3 | 0 | 1 | 1 | NA | Quercetin vs SOC | Pakistan | 42 |
| SCTR20061006 | 1 | Completed | 3 | 0 | 2 | 1 | 3 | 5000 IU versus 1000 IU Vitamin D3 | Saudi Arabia | 69 |
| NCT04685512 | 1 | Completed | 3 | 0 | 1 | 1 | 3 | Emtricitabine/Tenofovir vs SOC | France | 60 |
| NCT04348409 | 1 | Completed | 1 | 0 | 1 | 1 | 1 | Nitazoxanide vs SOC | Brazil | 50 |
| NCT04578158 | 1 | Completed | 3 | 0 | 1 | 1 | 1 | Quercetin vs SOC | Pakistan | 152 |
| NCT04355936 | 1 | Terminated early | 3 | 0 | 1 | 1 | 3 | Telmisartan vs SOC | Argentina | 158 |
| NCT04381884 | 1 | Completed | 3 | 0 | 1 | 1 | 2 | Ivermectin vs SOC | Argentina | 45 |
| EudraCT2020-001236-10 | 1 | Completed | 1 | 0 | 1 | 1 | 1,2,3 | Imatinib vs SOC | Netherlands | 385 |
| NCT04329611 | 1 | Terminated early | 1 | 0 | 1 | 1 | 2,3 | Hydroxychloroquine vs SOC | Canada | 148 |
| NCT04716569 | 1 | Completed | 3 | 0 | 1 | 1 | 3 | Ivermectin vs SOC | Egypt | 114 |
| NCT04311177 | 1 | Terminated early | 1 | 0 | 1 | 1 | 2 | losartan vs SOC | USA | 117 |
| NCT04348305 | 1 | Terminated early | 1 | 0 | 1 | 1 | 1,2 | Hydrocortisone vs SOC | Multisite | 30 |
| NCT04469114 | 1 | Completed | 1 | 0 | 1 | 1 | 1 | Tofacitinib vs SOC | Brazil | 289 |
| NCT04322396 | 1 | Terminated early | 1 | 0 | 1 | 1 |  | Hydroxychloroquine + Azithromycin vs SOC | Denmark | 117 |

## **Table S4. Detailed patient characteristics for included studies.**

| **Registration number** | **Age** | **Male %** | **Mechanical ventilation at baseline (%)** | **Patient Type (Severe to Critical, Mild to Moderate)** | **Severity** | **Current or unspecified smokers (%)** | **Former smokers (%)** | **Pregnant (%)** | **Inpatient (%)** | **Confirmed COVID-19 (%)** |
| --- | --- | --- | --- | --- | --- | --- | --- | --- | --- | --- |
| NCT04384380 | 32.9 (10.7) | 57.60 | NA | Mild (87.9) or Moderate (12.1) | 0 | NA | NA | 0.00 | 100 | 100 |
| NCT04261517 | 48.6 (4.1) | 70.00 | NA | Moderate (100) | 0 | NA | NA | 0.00 | 100 | 100 |
| jRCTs041190120 | 50 [38, 64.5] Median IQR | 61.40 | NA | Asymptomatic (31) or Mildly (69) | 0 | NA | NA | 0.00 | 100 | 100 |
| IRCT20100228003449N28 | 57.7 (15.1) | 54.30 | NA | Severe (100) | 1 | NA | NA | 0.00 | 100 | 100 |
| NCT04383535 | Treatment vs SOC: 62.5 [53, 72.5] 62 [49. 71] Median IQR | 67.60 | 0.00 | Severe (100) | 1 | NA | NA | 0.00 | 100 | 100 |
| ChiCTR2000029853 | 52 [17, 76] median IQR | 60.00 | 0.00 | Mild (15) or Moderate (85) | 0 | 25 | NA | 0.00 | 100 | 100 |
| ChiCTR2000029308 | 58 [49, 68] median IQR | 60.30 | 16.10 | Severe | 1 | NA | NA | 0.00 | 100 | 100 |
| ChiCTR2000030262 | 35.35 (11.2) | 63.80 | NA | Moderate | 0 | NA | NA | 0.00 | 100 | 100 |
| NCT04333420 | 60 (9) | 73.00 | NA | Severe | 1 | NA | NA | 0.00 | 100 | 100 |
| NCT04345614 | 59.3 (12.7) | 46.70 | NA | Severe to critical | 1 | NA | NA | NA | 100 | 100 |
| NCT04434248 | NA | NA | NA | Moderate | 0 | NA | NA | 0.00 | 100 | 100 |
| NCT04321278 | Treatment vs SOC:59.4 [49.3, 70.0] vs 60.2 [52.0, 70.1] Median IQR | 66.00 | 49.40 | Severe | 1 | 9.1 | NA | NA | 100 | 100 |
| ChiCTR2000029544 | 52.5 (12.5) | 72.40 | NA | NA | 0.5 | NA | NA | NA | 100 | 100 |
| CTRI202004024775 | Treatment vs soc: 52 [42, 60] vs 52 [41, 60] median IQR | 76.30 | NA | Moderate | 0 | 8 (ever smoker) | NA | 0.00 | 100 | 100 |
| NCT04381936_Dex | 66.2 (15.7) | 63.60 | 15.68 | NR | 0.5 | NA | NA | 0.00 | 100 | 100 |
| RBR8969zg | Treatment vs soc: 59 [47, 70] vs 58 [48, 70] | 59.30 | NA | Severe | 1 | NA | NA | 0.00 | 100 | 100 |
| NCT04366908 | 53.0 (10.2) | 59.20 | NA | NR | 0.5 | NA | NA | 0.00 | 100 | 100 |
| NCT04326790 | Treatment vs soc: 65 [54, 80] vs 63 [55, 70] | 58.10 | 0.00 | Mild to Moderate | 0 | 4.4 | 22.2 | 0.00 | 100 | 100 |
| ChiCTR2000029757 | Treatment vs soc: 70 [62, 80] vs 69 [63, 76] | 58.30 | 96.10 | Severe | 1 | NA | NA | 0.00 | 100 | 100 |
| NCT04327401 | 61.4 (14.6) | 62.50 | 100.00 | Severe | 1 | 4.3 | NA | 0.00 | 100 | 100 |
| NCT042323527 | 51.1 (13.9) | 75.30 | NA | Severe | 1 | 8.3 | 22.9 | 2.50 | 100 | 100 |
| NCT02517489 | Treatment vs soc: 63.1 [ 51.5, 70.8] vs 66.3 [53.5, 72.7] | 69.80 | 81.20 | Severe | 1 | 23.9 (ever smoker) | NA | NA | 100 | 100 |
| NCT02735707_hyd | 59.9 (12.8) | 71.10 | 85.20 | Severe | 1 | NA | NA | NA | 100 | 100 |
| NCT04381936_Hydro | 65.3 (15.3) | 62.20 | 16.82 | NR | 0.5 | NA | NA | 6/4716 | 100 | 91 |
| NCT04332991 | Treatment vs soc: 58 [45, 69] vs 57 [43, 68] | 55.70 | 64.90 | 87 Severe among 479 | 0.1816 | NA | NA | NA | 100 | 100 |
| ChiCTR2000030007 | 45 [40, 55] | 56.00 | 87.00 | Mild to Moderate | 0 | NA | NA | 0.00 | 100 | 100 |
| NCT04292730 | 10-day treat vs 5-day treat vs soc: 56 [45, 66] vs 58 [48, 66] vs 57 [45, 66] | 60.80 | 15.90 | Moderate | 0 | NA | NA | NA | 100 | 100 |
| NCT04346355 | 60.0 [53.0, 72.0] Median IQR | 61.10 | 0.00 | Mild or Moderate | 0 | NA | NA | NA | 100 | 100 |
| NCT04331808 | Treatment vs soc: 64.0 [57.1, 74.3] vs 63.3 [57.1, 72.3] | 67.70 | NA | Moderate to Severe | 0.5 | 2.3 | 6.3 | NA | 100 | 100 |
| NCT04449718 | Mean (SD) 56.5 (13.8); 56.0 (15.0) | 56.12 | 13.08 | Moderate (10.55%); Severe (89.45%) | 0.8945 | NA | NA | 0.00 | 100 | 100 |
| NCT04252885 | LPV/r vs Aribdo vs SOC: 50.7 (17-19) vs 50.5 (20-74) vs 44.3 (27-62) mean range | 46.50 | 15.40 | Mild or Moderate | 0 | NA | NA | 0.00 | 100 | 100 |
| ChiCTR20000300001 | 58 [48, 65] | 50.00 | NA | NR | 0.5 | 11.5 | NA | 0.00 | 100 | 100 |
| NCT04356937 | Median (IQR) 59.8 (45.3, 69.4) | 58.00 | 0.00 | Moderate | 0 | 3 | 30 | NA | 100 | 100 |
| IRCT20151227025726N20 | 53.6 (13.5) | 77.40 | NA | Severe | 1 | 1(ever smoking) | NA | 0.00 | 100 | 100 |
| IRCT20200328046886N1 | Treatment vs soc: 45 [38, 69] vs 60 [47.5 vs 68.5] | 37.50 | NA | Moderate | 0 | NA | NA | 0.00 | 100 | 100 |
| IRCT2019072704434N1 | 57.7 (9.3) | 59.30 | NA | Mild or Moderate | 0 | 1.9 | NA | NA | 100 | 100 |
| NCT04342663 | Treatment vs soc: 46 [35, 58] vs 45 [36, 54] | 36.80 | NA | Mild | 0 | NA | NA | NA | 0 | 100 |
| EudraCT202000193437 | Mean (SD) 66 (12); 73 (11) | 58.00 | NA | Moderate to Severe | 0.5 | NA | NA | 0.00 | 100 | 100 |
| NCT04304053 | 42.0 (12.8) | 60.94 | 0.00 | Mild or Moderate | 0 | NA | NA | 0.00 | 0 | 100 |
| NCT04308668 | Treatment vs soc: 41[33, 49] vs 39 [31, 50] | 43.70 | NA | Mild | 0 | 4 | NA | 0.00 | 0 | 100 |
| ChiCTR2000029868 | 46.1 (14.7) | 55.00 | NA | Mild (15) to Moderate (84) severe (1) | 0 | NA | NA | 0.00 | 100 | 100 |
| NCT04353336 | 40.72(19.32) | 58.80 | NA | NR | 0.5 | 31.4 | NA | 0.00 | 100 | 100 |
| NCT04329832 | 55 [42, 65] | 61.00 | 16.00 | 31% NIV + MV and MV+other | 0.31 | NA | NA | NA | 100 | 100 |
| NCT04322123 | 50.3 (14.6) | 58.30 | 0.00 | Mild to Moderate | 0 | 6.6 (ever smoking) | NA | NA | 100 | 76 |
| IRCT20100228003449N27 | Treatment vs soc: 60 [47, 73] vs 61 [50, 71] | 59.10 | 1.50 | Severe | 1 | NA | NA | 0.00 | 100 | 100 |
| NCT04381936_Lopi | 66.3 (15.9) | 61.10 | 4.05 | NR | 0.5 | NA | NA | 0.10 | 100 | 100 |
| NCT04343729 | 55 (15) | 64.60 | 81.60 | NR | 0.5 | NA | NA | 0.00 | 100 | suspected |
| ChiCTR2000029387 | 42.5 (11.5) | 46.00 | 0.00 | Mild to Moderate | 0 | NA | NA | 0.00 | 100 | 100 |
| NCT04331899 | Median (Range) 36 (18- 71) | 58.30 | NA | Mild to Moderate | 0 | NA | NA | 0.00 | 0 | 100 |
| NCT04354259 | Median (IQR) 48 (30-53); 39 (33-55) | 41.67 | NA | Moderate | 0 | NA | NA | 0.00 | 0 | 100 |
| Oman | 55 (14) | 58.00 | NA | Moderate to Severe | 0.5 | NA | NA | 0.00 | 100 | 100 |
| NCT04356534 | 51.7 (13.6) | 80.00 | NA | Severe | 1 | 0 | NA | NA | 100 | 100 |
| NCT04349592 | H+A vs H vs soc: 42 [38, 48] vs 40 [31, 47] vs 41 [31, 47] | 98.50 | NA | Mild to Moderate | 0 | NA | NA | 0.00 | 0 | 100 |
| NCT04280705 | 58.9 (15.0) | 64.40 | 45.00 | Mild/moderate (15%);severe (85%) | 0.85 | NA | NA | NA | 100 | 100 |
| NCT04257656 | Treatment vs soc: 66 [57, 73] vs 64 [53, 70] | 59.30 | 16.10 | Severe | 1 | NA | NA | 0.00 | 100 | 100 |
| NCT04315948 | 35 (<50 years old) | 62.00 | 8.00 | NR | 0.5 | 7 | NA | NA | 100 | 100 |
| JAK | 63 [58, 68] | 58.50 | 12.20 | Severe | 1 | 9.8 (erver smoking) | NA | 0.00 | 100 | 100 |
| ChiCTR2000029496 | N vs L+N vs LPV: 46.5 [40.0, 63.8] vs 50.0 [37.8, 62.8] vs 37.0 [26.0, 54.0] | 47.20 | NA | Moderate (84), Severe (5) | 0.0538 | NA | NA | NA | 100 | 100 |
| IRCT20200128046294N2 | Treatment vs soc: 58 [38, 65] vs 62 [49, 70] | 51.50 | NA | Moderate or Severe | 0.5 | NA | NA | NA | 100 | 100 |
| RBR949z6v | 56.5 (13.1) | 80 | 100 | Severe | 1 | NA | NA | 0 | 100 | 100 |
| ChiCTR2000030058 | Treatment vs soc: 56.0 [43.0, 67.3] vs 55.5 [47.8, 66.5] | 45.80 | NA | Mild/moderate (39); severe (9) | 0.1875 | NA | NA | 0.00 | 100 | 100 |
| NCT04276688 | Treatment vs soc: 51.0 [31.0, 61.3] vs 52.0 [33.5, 62.5] | 53.50 | NA | Mild to Moderate | 0 | 5.5 | NA | 0.00 | 100 | 100 |
| NCT04447534 | 43.56 (13.88) | 60.70 | NA | Severe 57/191 | 0.298 | 42.4 | NA | 0.00 | 100 | 100 |
| NCT04385095 | 57.1 (13.2) | 59.20 | 2.00 | NA | 0.5 | 2 | 27.6 | 0.00 | 100 | 100 |
| NCT04372186 | 55.9 (14.4) | 59.20 | 26.50 | Moderate | 0 | 5.8 | 17 | NA | 100 | 100 |
| IRCT20200501047259N1 | 56 [46, 62] | 69.50 | NA | Severe | 1 | NA | NA | NA | 100 | 100 |
| NCT04552483 | 5.6% (>=60 years old) | 46.90 | NA | Mild | 0 | NA | NA | NA | NA | 100 |
| NCT04325893 | 77 [58, 86] | 48.40 | 60.40 | Mild | 0 | 2.4 | NA | 0.00 | 100 | 100 |
| NCT04288102 | 60.45 (8.66) | 56.00 | 1.00 | Severe | 1 | NA | NA | 0.00 | 100 | 100 |
| Umi | 36.5 (12.1) | 60.00 | NA | Mild | 0 | NA | NA | 0.00 | 100 | 100 |
| IRCT202003117046797N4 | 59.8 (14.9) | 56.40 | NA | NA | 0.5 | NA | NA | 0.00 | 100 | 100 |
| NCT04369742 | 66.2 (16.2) | 59.40 | 0.80 | (21+62)/128 severe | 0.6484 | 6.2 | 28.1 | 0.00 | 100 | 100 |
| NCT04375098 | Mean (Range) Early 64.3 (33–92); Deferred 67.1 (27–91) | 50.00 | NA | NR | 0.5 | NA | NA | 0.00 | 100 | 100 |
| RPCEC00000317-En | 45.35 (12.0) | 70.00 | NA | 2/20 severe | 0.1 | NA | NA | NA | 100 | 100 |
| NCT04273763 | Treatment vs soc: 53 [50, 62] vs 48 [32, 51] | 77.80 | NA | NA | 0.5 | NA | NA | 0.00 | 100 | 100 |
| NCT04320615 | 60.8 (14.3) | 69.90 | 37.70 | Severe | 1 | NA | NA | NA | 100 | 100 |
| Linazi | 58.75 (15.55) | 62.50 | 0.00 | NR | 0.5 | NA | NA | 0.00 | 100 | 100 |
| NCT04292899 | Median (IQR) 61 (50-69); 62 (50-71) | 63.73 | 30.73 | Severe | 1 | NA | NA | NA | 100 | 100 |
| Iver_Ahmed | Mean 42 | NA | 0.00 | Mild to Moderate | 0 | NA | NA | NA | 100 | 100 |
| NCT04381936_Azi | Mean (SD) 65.4 (15.6); 65.2 (15.7) | 62.08 | 5.82 | NA | 0.5 | NA | NA | 0.32 | 100 | 91 |
| NCT04401579 | Mean (SD) 55.4 (15.7) | 63.12 | 31.66 | Moderate (68.3%); Severe (31.7%) | 0.317 | NA | NA | NA | 100 | 100 |
| IRCT20180725040596N2 | Mean (SD) 56.4 (16.3) | 60.00 | 5.00 | Mild (19%); Moderate (58%); Severe (23%) | 0.23 | 15 | NA | 0.00 | 100 | 100 |
| ISRCTN59048638 | Mean (SD) 55.3 (10.3); 54.0 (10.9) | 47.33 | 0.00 | Mild | 0 | NA | NA | NA | 0 | 100 |
| NCT04420247 | Mean (SD) 54.7 (12.1); 52.8 (12.6) | 66.67 | 18.10 | Severe (18.1%) | 0.181 | NA | NA | 0.00 | 100 | 100 |
| IRCT20200403046926N1 | Median (IQR) 43 (37–52); 47.5 (37–53) | 47.27 | 0.00 | Mild | 0 | NA | NA | 0.00 | 0 | 100 |
| NCT04355728 | Mean (SD) 58.58 (15.93); 58.83 (11.61) | 54.17 | 100.00 | Severe | 1 | NA | 8.333333 | NA | 100 | 100 |
| NCT04484493 | Median (IQR) 28.0 (20.5–38.0); 30.0 (22.5–39.0) | 46.00 | NA | Mild (70%); Moderate (24%); Severe (6%) | 0.06 | NA | NA | 0.00 | 31 | 100 |
| NCT04365153 | Median (IQR) 67.0 (60.9-74.0) | 75.00 | 50.00 | Severe (50%) | 0.5 | 35 | | 0.00 | 100 | 100 |
| CTRI-2020-08-027225 | Mean (SD) 50.7 (12.7); 54.2 (16.3) | 72.32 | 0.00 | Mild/Moderate | 0 | NA | NA | 0.00 | 100 | 100 |
| NCT04315948_Discovery | Median (IQR) 63 (54-71) | 71.70 | 36.88 | Moderate (63.8%); Severe (36.2%) | 0.362 | 18 | NA | 0.00 | 100 | 100 |
| NCT04479163 | Mean (SD) 76.4 (8.7); 77.9 (8.4) | 37.50 | NA | Severe | 1 | NA | NA | 0.00 | 100 | 100 |
| ChiCTR2000029765 | Median (IQR) 63.5 (58–71); 63 (54–69) | 50.77 | NA | Moderate (56.92%); Severe (43.08%) | 0.4308 | NA | NA | 0.00 | 100 | 100 |
| NCT04390022 | Median (IQR) 26 (19-36); 26 (21-44) | 50.00 | NA | Mild/moderate | 0 | NA | NA | NA | 100 | 100 |
| NCT04403685 | Mean (SD) 57.4 (15.7); 57.5 (13.5) | 68.22 | 48.06 | Severe | 1 | NA | NA | NA | 100 | 100 |
| CTRI-2020-04-024729 | Mean (SD) 35.6 (11.3); 34.2 (9.4) | 78.333 | 0 | Asymptomatic/Mild | 0 | 15 | 1.666667 | 0.00 | 0 | 100 |
| NCT04273321 | Median (IQR) 57 (49–67); 55 (38–65) | 47.67 | NA | Moderate | 0 | 12.7907 | NA | 0.00 | 100 | 100 |
| NCT04322682 | Mean (SD) 54.4 (9.7); 54.9 (9.9) | 46.06 | 0.00 | Mild | 0 | NA | NA | 0.00 | 0 | 100 |
| NCT04351295 | Mean (SD) 36.15 (17.67); 34.86 (15.95) | 48.91 | 0.00 | Mild/Moderate | 0 | NA | NA | 0.00 | 100 | 100 |
| NCT04341584 | Median (IQR) 67.0 (55.5–74.3); 64.9 (59.5–78.3) | 70.18 | 0.00 | Mild/Moderate | 0 | 0 | 18.34862 | 0 | 100 | 100 |
| NCT04327388 | Median (IQR) SOC 60.0 (53.0–69.5); Sarilumab 200mg 58.0 (51.0–67.0); Sarilumab 400mg 58.0 (48.0–67.0) | 62.74 | 13.22 | Severe/Critical | 1 | NA | NA | 0.00 | 100 | 100 |
| IRCT20100228003449N29 | Median (IQR) 61.5 (46.5-74.25); 63 (53.25-70.75) | NA | 0.00 | Mild/Moderate | 0 | 3.658537 | NA | 0.00 | 100 | 50 |
| CTRI-2020-05-025114 | Mean (SD) 43.6 (12.2); 43.0 (11.2) | 73.47 | 0.00 | Mild/Moderate | 0 | NA | NA | 0.00 | 100 | 100 |
| NCT04381936_Toci | Mean (SD) 63.3 (13.7); 63.9 (13.6) | 67.35 | 13.65 | Severe (54.6%) | 0.5462 | NA | NA | 0.07 | 100 | 100 |
| NCT04321616 | Mean (SD) 59.8 (15.3) | 65.75 | NA | NA | 0.5 | 39.22652 | NA | 0.00 | 100 | 100 |
| NCT04416399 | Median (IQR) 44 (19–71); 46 (19–79) | 42.45 | 0.00 | Mild | 0 | NA | NA | NA | 0 | 100 |
| IRCT20200411047025N1 | Mean (SD) 57.53 (18.27); 61 (15.90) | 50.00 | NA | Severe | 1 | NA | NA | 0.00 | 100 | 100 |
| CTRI-2020-06-026222 | Mean (SD) 48.4(11.6); 49.0 (13.5) | 33.00 | 0.00 | Moderate | 0.5 | NA | NA | 0.00 | 100 | 100 |
| NCT04342728 | Mean (SD) 45.2 (14.6) | 38.32 | 0.00 | Mild | 0 | 31.7757 | | 0.00 | 0 | 100 |
| INA-TX6YYSS | Median (Range) 37 (20-55) | 95.34 | 0.00 | Mild (92.4%); Moderate (7.6%) | 0 | NA | NA | 0.00 | 100 | 100 |
| NCT04364893 | Median (IQR) 55 (46.1-63.1); 56 (46.1-66.1) | 59.636 | 0 | Mild (57.1%); Moderate (42.9%) | 0 | 20.36424 | NA | NA | 100 | 100 |
| ISRCTN40302986 | Mena (SD) 44.1 (14.7) | 69.35 | 0.00 | Mild/Moderate | 0 | NA | NA | NA | 100 | 100 |
| NCT04365127 | Mean (SD) 56.0 (17.3); 54.6 (16.0) | NA | 0.00 | Mild/Moderate | 0 | NA | NA | NA | 100 | 100 |
| ChiCTR2000029638 | Median (IQR) 51.0 (33.5–59.3); 56.0 (49.3–69.0) | 46.81 | 0.00 | Moderate (88.3%); Severe (11.7%) | 0.117 | NA | NA | 0.00 | 100 | 100 |
| NCT04329923 | Median (Range) 53 (40-80) | 26.47 | 0.00 | Mild | 0 | 5.882353 | NA | 0.00 | 0 | 100 |
| NCT04354428 | Median (Range) 37 (18-78) | 43.29 | 0.00 | Mild | 0 | NA | NA | NA | 0 | 100 |
| Brazilian 4.173.074 | Mean (SD) 44.5 (13.1); 46.1 (12.7); | 54.24 | 0.00 | Mild/Moderate | 0 | NA | NA | NA | 0 | 100 |
| EAT-DUTA AndroCoV | Mean (SD) 40 (10.8); 43.8 (14.1) | 100.00 | 0.00 | Mild/Moderate | 0 | 4.597701 | NA | 0.00 | 0 | 100 |
| NCT04405843 | Median (IQR) 37 (29-47.7); 37 (28.7-49.2) | 41.96 | 0.00 | Mild/Moderate | 0 | 2.763819 | NA | 0.00 | 1.00503 | 100 |
| ISRCTN86534580_Azi | Mean (SD) 60.9 (7.9); 60.5 (7.8) | 43.16 | 0.00 | Mild | 0 | 12.89625 | 36.31124 | NA | 0 | 38 |
| NCT04338009 | Mean (SD) 62 (12) | 55.263 | NA | Mild (52.6%); Moderate (34.9%); Severe (12.5%) | 0.125 | 8.552632 | NA | 0 | 100 | 100 |
| CTRI-2020-05-025369 | Median (IQR) 56 (47–63); 54 (43–63) | 84.92 | 5.03 | Moderate(49.2%); Severe (50.8%) | 0.508 | NA | NA | NA | 100 | 100 |
| NCT04381936_CP | Mean (SD) 63.5 (14.7); 63.4 (14.6) | 64.28 | 4.47 | NA | 0.5 | NA | NA | 0.24 | 100 | 100 |
| RBR-8h7q82 | Mean (SD) HCQ 54.8 (15.5); CQ 51.9 (14.0); IV 53.2 (17.3) | 56.55 | NA | Severe | 1 | 9.52381 | 41.07143 | NA | 100 | 100 |
| ACTRN12620000454976 | Mean (SD) 59.8 (16.8); 63.8 (16.9) | 63.64 | NA | NA | 0.5 | NA | NA | NA | 100 | 100 |
| NCT02735707, NCT04505774, NCT04359277, NCT04372589_Critical | Mean (SD) 60.4 (13.1); 61.7 (12.5) | 69.991 | 66.1831369 | Severe | 1 | NA | NA | NA | 100 | 100 |
| NCT04359810 | Median (IQR) 60 (48-71); 63 (49-72) | 65.92 | 94.17 | Severe (94.17%) | 0.942 | NA | NA | NA | 100 | 100 |
| NCT04427501_P2 | Median (IQR) 700mg 39 (31-58); 2800mg 45 (31-56); 7000mg 46 (34-55); combination 44 (30-60); SOC 46 (35-57) | 45.41 | 0.00 | Mild/Moderate | 0 | NA | NA | NA | 0 | 100 |
| CTRI-2020-06-026087 | Mean (SD) 49.35 (14.89); 49.10 (12.44) | 75.00 | 0.00 | Moderate | 0 | NA | NA | 0.00 | 100 | 100 |
| IRCT20200505047318N1 | Median (IQR) 71 (62-81); 72 (65-77) | 100.00 | NA | Moderate/Severe | 0.5 | NA | NA | 0.00 | 100 | 100 |
| IRCT20180802040678N4 | Mean (SD) 67.3 (14.8); 60.1 (17.3) | 51.25 | NA | NA | 0.5 | 13.75 | NA | 0.00 | 100 | 100 |
| NCT04431466 | Mean (SD) 49.4 (14.6) | 45.161 | 0 | Mild | 0 | NA | NA | 0 | 100 | 100 |
| Tolouian_Bromhexine | Mean (SD) 50.7 (16.4); 53.1 (15.2) | 46.00 | NA | NA | 0.5 | 13 | NA | 0.00 | 100 | 100 |
| NCT04486508 | Median (IQR) 62 (51-70.7) 61 (47-71) | 57.83 | 54.45 | Moderate (45.5%); Severe (54.5%) | 0.545 | 9.964413 | NA | 0.00 | 100 | 100 |
| IRCT20200318046812N1 | Mean (SD) 58.6 (17.5); 56.6 (17.1) | 54.96 | NA | Moderate/Severe | 0.5 | NA | NA | 0.00 | 100 | 100 |
| NCT04399980 | Median (IQR) 54.8 (49.7–68.1); 59.0 (41.0–69.3) | 65.00 | 50.00 | Moderate (50%); Severe (50%) | 0.5 | 27.5 | | 0.00 | 100 | 100 |
| IRCT20190810044500N7 | Mean (SD) 30.0 (14); 32.0 (15) | 60.00 | 0.00 | Mild/Moderate | 0 | 12 | NA | 0.00 | 0 | 100 |
| Mahajan_Remdesivir | Mean (SD) 58.08 (12.1) 57.41 (14.1) | 68.57 | 24.30 | Severe (24.3%); Moderate (75.7%) | 0.243 | NA | NA | NA | 100 | 100 |
| IRCT20151226025699N3 | Mean (SD) 66 (14.58); 64 (14.25) | 59.41 | NA | Critically ill | 1 | NA | NA | NA | 100 | 100 |
| NCT04349241 | Mean (SD) 36.3 (12.5); 36.4 (11.5) | 50.00 | 0.00 | Mild/Moderate | 0 | NA | NA | 0.00 | 100 | 100 |
| NCT04569825 | Median (IQR) 29 (23–35); 30 (24–38) | 28.26 | 0.00 | Mild | 0 | NA | NA | 0.00 | 0 | 100 |
| ISRCTN86534580_Budesonide | Mean (SD) 64.7 (7.3); 63.8 (7.8) | 48.19 | 0.00 | Mild | 0 | 5.308831 | 40.93925 | NA | 0 | 100 |
| IRCT20200310046736N1 | <=50 (38.33%); >50 (61.67%) | 55.00 | 100.00 | Severe | 1 | NA | NA | 0.00 | 100 | 100 |
| NCT04494984 | Median (IQR) 54 (43-63); 54 (45-65) | 65.15 | 2.90 | Severe (39%); Moderate (61%) | 0.39 | NA | NA | 0.00 | 100 | 100 |
| NCT04128579 | Mean (SD) 49.55 (12.49); 48.30 (14.62) | 86.67 | NA | Moderate/Severe | 0.5 | NA | NA | NA | 100 | 100 |
| IRCT20200204046369N1 | NA | 56.98 | NA | NA | 0.5 | 15.11628 | 22.09302 | 0.00 | 100 | 100 |
| NCT04343768 | Median (IQR) 69.0 (55.0–82.0) | 51.67 | NA | Severe | 1 | 30 | NA | 0.00 | 100 | 100 |
| Sakoulas_IVIG | Median 57; 54 | NA | 0.00 | Moderate | 0 | NA | NA | NA | 100 | 100 |
| NCT04252664 | Median (Range) 57 (12-95) | 61.00 | 0.00 | Moderate | 0 | NA | NA | NA | 100 | 100 |
| NCT04453384 | Median (IQR) 71 (51-75) | 64.71 | 0.00 | Moderate | 0 | NA | NA | 0.00 | 100 | 100 |
| IRCT20151227025726N17 | Median (IQR) 62 (52–71); 62 (54–68) | 72.00 | 0.00 | Mild/Moderate | 0 | NA | NA | 0.00 | 100 | 100 |
| NCT04491994 | Mean (SD) 34 (11.778); 34 (9.813) | 93.20 | 0.00 | Mild | 0 | NA | NA | NA | 100 | 100 |
| NCT04344535 | Mean (SD) 67 (15.8); 64 (17.4) | 59.46 | 18.92 | NA | 0.5 | NA | NA | 0.00 | 100 | 100 |
| NCT04403100 | Median (IQR) 53 (18-81); 54 (18-94); 53 (18-80) | 44.96 | 0.00 | Mild/Moderate | 0 | 5.109489 | NA | NA | 0 | 100 |
| NCT04381962 | Mean (SD) 45.53 (14.23) 46.30 (15.53) | 51.53 | 0.00 | Mild/Moderate | 0 | 11.18644 | 17.28814 | NA | 0.7 | 66 |
| NCT04321096 | Median (IQR) 62 (51-75); 61 (55-74) | 60.00 | 7.80487805 | Severe (7.8%); Moderate (92.2%) | 0.078 | NA | NA | 0.00 | 100 | 100 |
| NCT04333589 | Mean (SD) 55.8 (14.2); 55.5 (12.6) | 45.45 | NA | Severe (5.5%); Moderate (92.7%); Mild (1.8%) | 0.0545 | NA | NA | 0.00 | 18.1818 | 100 |
| NCT04646109 | Mean (SD) 58.17 (11.52) 66.23 (13.31) | 66.67 | 3.33 | Severe | 1 | NA | NA | 0.00 | 100 | 100 |
| NCT02735707_toci | Mean (SD) 61.5 (12.5); 63.4 (13.4); 61.1 (12.8) | 72.72 | 70.87 | Severe | 1 | NA | NA | 0.00 | 100 | 100 |
| NCT04519385 | Median (IQR) 64 (55-72); 60.5 (49.5-66.5) | 56.88 | 100.00 | Severe | 1 | NA | NA | NA | 100 | 100 |
| NCT04310228 | Median (IQR) 75 (34–81); 70 (45–89); 71 (48–77) | 53.85 | 3.85 | Moderate (46.1%); Severe (53.9%) | 0.539 | 46.15385 | NA | 0.00 | 100 | 100 |
| NCT04501978 | Median (IQR) 63 (50–72); 59 (48–71) | 56.37 | 15.29 | Severe (15.3%); Moderate (84.7%) | 0.153 | NA | NA | 0.96 | 100 | 100 |
| ChiCTR2000031494 | Mean (SD) 61.00 (17.87); 57.86 (15.79) | 58.54 | 24.39 | Severe | 1 | NA | NA | NA | 100 | 100 |
| RBR-6sx3sz | <=35 years 11.4%; 36-59 years 57.1%; >=60 years 31.4% | 37.14 | 0.00 | Mild/Moderate | 0 | NA | NA | NA | 100 | 100 |
| NCT04340557 | Median 53; 53 | 61.29 | 0.00 | Mild/Moderate | 0 | NA | NA | NA | 100 | 100 |
| NCT04523831 | Mean (SD) 41 (14); 38 (12) | 58.75 | 0.00 | Mild/Moderate | 0 | NA | NA | 0.00 | 33 | 100 |
| NCT04323228 | Mean (SD) 45.08 (9.19); 52.80 (10.84) | 63.64 | 0.00 | Mild/Moderate | 0 | NA | NA | 0.00 | 100 | 100 |
| NCT04549376 | Mean (SD) 43.98 (12.67) | 84.13 | 0.00 | Severe (1.9%) | 0.019 | NA | NA | NA | NA | 100 |
| CTRI202012030083 | Mean (SD) 45 (13) | 74.71 | 0.00 | Mild/Moderate | 0 | NA | NA | 0.00 | 100 | 100 |
| NCT04370288 | Mean (SD) 53.7 (13); 55.2 (13.8) | 52.50 | NA | Severe | 1 | NA | NA | 0.00 | 100 | 100 |
| NCT04352959 | Mean (SD) 42.06 (14.97); 44.08 (16.16) | 45.45 | 0.00 | Mild | 0 | NA | NA | 0.00 | 0 | 100 |
| NCT04347174 | Median (IQR) 59 (52–62.5); 51 (45–65) | 69.05 | 30.95 | Severe | 1 | NA | NA | 0.00 | 100 | 100 |
| IRCT20130812014333N145 | Mean (SD) 53.6 (16.3); 54.6 (19.4) | 55.00 | NA | NA | 0.5 | NA | NA | 0.00 | 100 | 100 |
| ChiCTR2000033627 | Mean (SD) 31.78 (7.85); 31.58 (7.68) | 50.00 | 0.00 | Asymptomatic/Mild | 0 | NA | NA | 0.00 | 0 | 100 |
| IRCT20111224008507N3 | Mean (SD) 47.63 (22.20); 45.18 (23.11) | 52.17 | 4.35 | Severe (44.9%) | 0.4493 | NA | NA | 0.00 | 0 | 100 |
| NCT04342182 | Median (IQR) 61 (56–70); 63 (55–77) | 72.09 | 15.12 | Severe (90.7%); Moderate (9.3%) | 0.907 | NA | NA | NA | 100 | 100 |
| NCT04403555 | Mean (SD) 42.38 (16.02); 39.38 (16.92) | 50.00 | NA | Mild/Moderate | 0 | NA | NA | 0.00 | 100 | 100 |
| NCT04394377 | Mean (SD) 56.7 (14.1); 56.5 (14.5) | 59.837 | 6.99186992 | Mild/Moderate(93.5%); Severe (6.5%) | 0.065 | 19.34959 | NA | 0 | 100 | 100 |
| VD_Elamir | NA | NA | NA | NA | 0.5 | NA | NA | NA | 100 | 100 |
| NCT04457609 | <40 (17.5%); 40-60 (37.5%); >60 (45%) | 75.00 | NA | Severe/Critical | 1 | NA | NA | 0.00 | 100 | 100 |
| NCT04521309 | Mean (SD) 55.9 (1.34); 59.1 (12.06) | 70.00 | 56.00 | Severe | 1 | NA | NA | 0.00 | 100 | 100 |
| NCT04330586 | Mean (SD) 44.9 (17.9); 49.0 (16.8) | 32.79 | 0.00 | Mild/Moderate | 0 | NA | NA | 0.00 | 100 | 100 |
| NCT04622891 | Mean (SD) 45.8 (18); 46.1 (19); 41.1 (18) | 70.16 | 0.00 | Mild | 0 | NA | 0 | NA | 100 | 100 |
| IRCT20200504047298N3 | Mean (SD) 51.98 (11.46); 52 (12) | 59.17 | NA | NA | 0.5 | 4.166667 | NA | NA | 82.5 | 100 |
| ISRCTN21085622 | Mean (SD) 40 (6.1); 48 (2.2); 46 (5.8) | 53.33 | NA | Moderate (38.33%); Severe (61.67%) | 0.6167 | NA | NA | 0.00 | 100 | 100 |
| NCT04542941 | Median (IQR) 48 (35–64); 53 (44–61) | 71.32 | 0.74 | Mild/Moderate | 0 | NA | NA | NA | 100 | 100 |
| NCT04446429 | Median (IQR) 45 (19); 46 (15) | 100.00 | 0.00 | Mild | 0 | NA | NA | 0.00 | 0 | 100 |
| NCT04372589, NCT04505774, NCT02735707, NCT04359277_Noncritical | Mean (SD) 59.0 (14.1); 58.8 (13.9) | 58.72 | 0.00 | Moderate | 0 | NA | NA | NA | 100 | 100 |
| NCT04353596 | Median (IQR) 74 (63–80); 75 (69–80) | 63.235 | 2.45098039 | NA | 0.5 | 7.352941 | NA | 0 | 100 | 100 |
| NCT04362813 | Median (IQR) 59 (49-69); 57 (50-68) | 33.04 | NA | Severe | 1 | NA | NA | NA | 100 | 100 |
| CTRI-2020-04-024479 | Mean (SD) 57.8 (12.6); 57.3 (14.1) | 72.73 | NA | Moderate/Severe | 0.5 | 27.27273 | NA | NA | 100 | 100 |
| NCT04324528 | Median (IQR) 62·0 (54.0–71.5); 59.0 (43.5–66.5) | 73.53 | 100.00 | Critically ill | 1 | 8.823529 | NA | NA | 100 | 100 |
| NCT04521400 | Mean (SD) 59.6 (16.3); 60.1 (16.8) | 61.905 | NA | Severe | 1 | NA | NA | 0 | 100 | 100 |
| ISRCTN86534580_Doxy | Mean (SD) 60.9 (7.9); 61.3 (7.7) | 44.08 | 0.00 | Mild | 0 | 11.10491 | 37.72321 | NA | 0 | 44 |
| NCT04392973 | Mean (SD) 53.03 (12.79); 52.27 (13.36) | 59.45 | 17.72 | Moderate (72.3%); Severe (17.7%); | 0.177 | NA | NA | 0.00 | 100 | 100 |
| NCT04498936 | NA | NA | 0.00 | Mild/Moderate | 0 | NA | NA | 0.00 | 100 | 100 |
| NCT04350593 | Mean (SD) 61.0 (13.4); 61.8 (13.5) | 57.36 | 0.00 | NA | 0.5 | 3.92 | NA | NA | 100 | 93 |
| NCT04332107 | Median (IQR) 42 (35-49); 44 (35-51) | 33.08 | 0.00 | Mild | 0 | 17.11027 | NA | 0.00 | 0 | 100 |
| IRCT20151113025025N3 | Mean (SD) 65.0 (9.9); 67.5 (9.8) | 46.88 | 12.50 | Severe (90.6%) | 0.9063 | 6.25 | 25 | 0.00 | 100 | 100 |
| IRCT20200408046987N1 | Median (IQR) 55 (45-70); 58 (45-68); 61 (42-68); 53 (42-65); 54 (47-60); 54 (46-65) | 50.00 | 0.00 | Mild | 0 | NA | NA | 0.00 | 100 | 100 |
| NCT04427501_P3 | Mean (SD) 54.3 (17.1); 53.3 (16.4) | NA | 0.00 | Mild/Moderate | 0 | NA | NA | 0.00 | 0 | 100 |
| NCT04350684 | Mean (SD) 62.1 (15.3); 60.2 (16.5) | 56.44 | 0.00 | Moderate/Severe | 0.5 | NA | NA | 0.00 | 100 | 100 |
| NCT02735707_LPH | Mean (SD) 61 (13); 56.3 (13); 60.3 (8.9); 60.8 (12.9) | 70.42 | 73.02 | Critically ill | 1 | NA | NA | 0.00 | 100 | 100 |
| NCT04547660 | Median (IQR) 59.0 (48.0-68.5); 62.0 (49.5-68.0) | 58.13 | 75.00 | Severe (75%); Moderate (25%) | 0.75 | NA | NA | 0.00 | 100 | 100 |
| IRCT20151227025726N23 | Mean (SD) 53.15 (12.9); 56.12 (11.51) | 58.82 | NA | Severe | 1 | NA | 7.352941 | NA | 100 | 100 |
| NCT04360824 | Median (IQR) 63.5 (30–85); 65 (24–86) | 56.069 | NA | Severe | 1 | 42.19653 | NA | 0 | 100 | 100 |
| NCT04409522 | Mean (SD) 50.75 (14.43); 52.95 (14.07) | 59.09 | 0.00 | Mild/Moderate | 0 | 4.545455 | NA | 0.00 | 100 | 100 |
| NCT04529525 | Mean (SD) 42.58 (15.29); 42.40 (15.75) | 52.69 | NA | Mild | 0 | 10.37924 | 28.54291 | 0.00 | 0 | 100 |
| NCT04341038 | Mean (SD) 61.5 (13.9); 64.8 (12.1) | 80.00 | 29.09 | Severe | 1 | NA | 30.90909 | NA | 100 | 100 |
| NCT04861298 | Mean (SD) 42.5 (3.3); 56.2 (3.3) | 47.62 | 0.00 | Mild | 0 | NA | NA | NA | 0 | 100 |
| SCTR20061006 | Mean (SD) 46.3 (15.2); 53.5 (12.3) | 49.275 | 0 | Mild/Moderate | 0 | NA | NA | 0 | 74.026 | 100 |
| NCT04685512 | Mean (SD) 39.9 (14.8); 42.6 (16.7) | 43.33 | 0.00 | Mild/Moderate | 0 | 30 | NA | 0.00 | 0 | 100 |
| NCT04348409 | Median (IQR) 64 (17); 64 (21) | 30.00 | 0.00 | Moderate | 0 | NA | NA | 0.00 | 100 | 100 |
| NCT04578158 | NA | 57.89 | 0.00 | Mild/Moderate | 0 | NA | NA | NA | 0 | 100 |
| NCT04355936 | Mean (SD) 63.7 (17.0); 66.9 (17.2) | 53.16 | NA | NA | 0.5 | NA | NA | 0.00 | 100 | 100 |
| NCT04381884 | Mean (SD) 42.3 (12.8); 38.1 (11.7) | 55.56 | 0.00 | Moderate | 0 | NA | NA | 0.00 | 100 | 100 |
| EudraCT2020-001236-10 | Median (IQR) 64 (57–73); 64 (55–74) | 68.57 | NA | Severe | 1 | 39.74026 | NA | 0.00 | 100 | 100 |
| NCT04329611 | Mean (SD) 46.7 (11.5); 46.9 (11.0) | 55.41 | 0.00 | Mild | 0 | 14.18919 | NA | 0.00 | 0 | 100 |
| NCT04716569 | Mean (SD) 44.8 (19.2); 45.5 (18.8) | 71.93 | 0.00 | Mild | 0 | 38.59649 | NA | 0.00 | 0 | 100 |
| NCT04311177 | Median (IQR) 37 (27-46); 38 (29-51) | 50.43 | 0.00 | Mild | 0 | NA | NA | 0.00 | 0 | 100 |
| NCT04348305 | Median (IQR) 59 (52-74); 62 (55-71) | 80.00 | 50.00 | Severe | 1 | NA | NA | NA | 100 | 100 |
| NCT04469114 | Mean (SD) 55 (14); 57 (14) | 65.05 | 0.00 | NA | 0.5 | 28.3737 | NA | NA | 100 | 100 |
| NCT04322396 | Median (IQR) 68 (52–80); 63 (52–74) | 55.56 | 3.42 | Moderate | 0 | 5.128205 | 41.02564 | 0.00 | 100 | 100 |

## **Table S5. Evaluation of risk of bias (mortality) (1: low; 2: probably low; 3: probably high, 4: high).**

| **Registration Number** | **Randomization** | **Deviations from the intended intervention** | **Missing outcome data** | **Measurement of outcome** | **Selection of the reported results** | **Final** |
| --- | --- | --- | --- | --- | --- | --- |
| ChiCTR2000029308 | 1 | 3 | 1 | 1 | 1 | 4 |
| ChiCTR2000029544 | 1 | 3 | 1 | 1 | 1 | 4 |
| ChiCTR2000029757 | 2 | 3 | 1 | 1 | 1 | 4 |
| ChiCTR2000029868 | 1 | 3 | 1 | 1 | 1 | 4 |
| ChiCTR2000030007 | 1 | 3 | 1 | 1 | 2 | 4 |
| ChiCTR2000031494 | 4 | 3 | 1 | 1 | 2 | 4 |
| CTRI-2020-04-024479 | 4 | 3 | 1 | 1 | 1 | 4 |
| CTRI202004024775 | 1 | 3 | 1 | 1 | 1 | 4 |
| CTRI-2020-05-025114 | 3 | 3 | 1 | 1 | 2 | 4 |
| CTRI-2020-05-025369 | 1 | 3 | 1 | 1 | 1 | 4 |
| CTRI-2020-06-026222 | 2 | 3 | 1 | 1 | 2 | 4 |
| CTRI-2020-08-027225 | 1 | 1 | 1 | 1 | 1 | 1 |
| CTRI202012030083 | 4 | 3 | 1 | 1 | 1 | 4 |
| EudraCT2020-001236-10 | 1 | 1 | 1 | 1 | 1 | 1 |
| EudraCT202000193437 | 1 | 3 | 1 | 1 | 1 | 4 |
| IRCT20100228003449N27 | 1 | 3 | 4 | 1 | 2 | 4 |
| IRCT20100228003449N28 | 1 | 4 | 3 | 1 | 2 | 4 |
| IRCT20111224008507N3 | 1 | 1 | 2 | 1 | 2 | 1 |
| IRCT20151113025025N3 | 2 | 1 | 2 | 1 | 1 | 1 |
| IRCT20151227025726N17 | 4 | 3 | 1 | 1 | 3 | 4 |
| IRCT20151227025726N20 | 4 | 3 | 1 | 1 | 3 | 4 |
| IRCT20200128046294N2 | 3 | 4 | 1 | 1 | 3 | 4 |
| IRCT20200204046369N1 | 1 | 1 | 1 | 1 | 1 | 1 |
| IRCT20200310046736N1 | 1 | 3 | 1 | 1 | 3 | 4 |
| IRCT20200318046812N1 | 1 | 3 | 2 | 1 | 1 | 4 |
| IRCT20200408046987N1 | 1 | 3 | 1 | 1 | 2 | 4 |
| IRCT20200411047025N1 | 3 | 3 | 1 | 1 | 2 | 4 |
| IRCT20200501047259N1 | 2 | 1 | 1 | 1 | 1 | 1 |
| IRCT20200504047298N3 | 2 | 1 | 1 | 1 | 1 | 1 |
| ISRCTN21085622 | 1 | 3 | 1 | 1 | 2 | 4 |
| ISRCTN40302986 | 1 | 1 | 1 | 1 | 2 | 1 |
| ISRCTN59048638 | 2 | 1 | 3 | 1 | 1 | 4 |
| ISRCTN86534580_Azi | 1 | 3 | 1 | 1 | 1 | 4 |
| ISRCTN86534580_Budesonide | 1 | 3 | 1 | 1 | 1 | 4 |
| ISRCTN86534580_Doxy | 1 | 3 | 1 | 1 | 1 | 4 |
| ISRCTN86534580 | 1 | 3 | 1 | 1 | 1 | 4 |
| Mahajan_Remdesivir | 2 | 3 | 1 | 1 | 3 | 4 |
| NCT02517489 | 1 | 1 | 1 | 1 | 1 | 1 |
| NCT02735707_hyd | 3 | 3 | 1 | 1 | 1 | 4 |
| NCT02735707_toci | 1 | 3 | 1 | 1 | 1 | 4 |
| NCT02735707_LPH | 1 | 3 | 1 | 1 | 1 | 4 |
| NCT02735707 | 1 | 3 | 1 | 1 | 1 | 4 |
| NCT04252664 | 4 | 3 | 1 | 1 | 1 | 4 |
| NCT04252885 | 1 | 3 | 1 | 1 | 2 | 4 |
| NCT04257656 | 1 | 1 | 1 | 1 | 1 | 1 |
| NCT04261517 | 3 | 3 | 1 | 1 | 1 | 4 |
| NCT04273321 | 2 | 3 | 1 | 1 | 1 | 4 |
| NCT04280705 | 1 | 1 | 1 | 1 | 1 | 1 |
| NCT04288102 | 1 | 1 | 1 | 1 | 1 | 1 |
| NCT04292730 | 1 | 4 | 2 | 1 | 1 | 4 |
| NCT04304053 | 1 | 3 | 4 | 1 | 1 | 4 |
| NCT04308668 | 1 | 1 | 1 | 1 | 1 | 1 |
| NCT04315948 | 1 | 3 | 1 | 1 | 1 | 4 |
| NCT04320615 | 1 | 3 | 1 | 1 | 1 | 4 |
| NCT04321096 | 1 | 1 | 1 | 1 | 1 | 1 |
| NCT04321278 | 1 | 3 | 1 | 1 | 1 | 4 |
| NCT04321616 | 1 | 3 | 1 | 1 | 1 | 4 |
| NCT04322123 | 1 | 3 | 1 | 1 | 1 | 4 |
| NCT04322396 | 1 | 1 | 1 | 1 | 1 | 1 |
| NCT04322682 | 2 | 1 | 1 | 1 | 1 | 1 |
| NCT04325893 | 2 | 1 | 1 | 1 | 1 | 1 |
| NCT04326790 | 1 | 3 | 1 | 1 | 1 | 4 |
| NCT04327388 | 1 | 1 | 1 | 1 | 2 | 1 |
| NCT04327401 | 1 | 3 | 1 | 1 | 1 | 4 |
| NCT04329611 | 1 | 1 | 1 | 1 | 1 | 1 |
| NCT04329832 | 1 | 4 | 1 | 1 | 1 | 4 |
| NCT04331808 | 1 | 3 | 1 | 1 | 1 | 4 |
| NCT04332107 | 1 | 1 | 4 | 1 | 1 | 4 |
| NCT04332991 | 1 | 1 | 1 | 1 | 1 | 1 |
| NCT04333589 | 1 | 3 | 1 | 1 | 2 | 4 |
| NCT04338009 | 1 | 3 | 1 | 1 | 2 | 4 |
| NCT04342182 | 1 | 3 | 1 | 1 | 1 | 4 |
| NCT04342728 | 1 | 3 | 1 | 1 | 2 | 4 |
| NCT04343729 | 1 | 1 | 1 | 1 | 1 | 1 |
| NCT04343768 | 1 | 3 | 1 | 1 | 3 | 4 |
| NCT04344535 | 1 | 1 | 1 | 1 | 2 | 1 |
| NCT04346355 | 1 | 3 | 1 | 1 | 1 | 4 |
| NCT04348305 | 1 | 1 | 1 | 1 | 1 | 1 |
| NCT04349241 | 3 | 3 | 1 | 1 | 2 | 4 |
| NCT04349592 | 1 | 1 | 1 | 1 | 2 | 1 |
| NCT04350593 | 1 | 1 | 1 | 1 | 1 | 1 |
| NCT04351295 | 3 | 3 | 1 | 1 | 1 | 4 |
| NCT04353336 | 3 | 3 | 1 | 1 | 1 | 4 |
| NCT04353596 | 2 | 3 | 1 | 1 | 1 | 4 |
| NCT04354428 | 2 | 1 | 1 | 1 | 2 | 1 |
| NCT04355728 | 1 | 1 | 1 | 1 | 1 | 1 |
| NCT04356534 | 2 | 3 | 1 | 1 | 1 | 4 |
| NCT04356937 | 2 | 1 | 1 | 1 | 1 | 1 |
| NCT04359810 | 1 | 1 | 1 | 1 | 1 | 1 |
| NCT04362813 | 1 | 1 | 1 | 1 | 1 | 1 |
| NCT04364893 | 1 | 3 | 1 | 1 | 1 | 4 |
| NCT04366908 | 3 | 3 | 1 | 1 | 1 | 4 |
| NCT04369742 | 2 | 1 | 4 | 1 | 1 | 4 |
| NCT04372186 | 2 | 1 | 1 | 1 | 2 | 1 |
| NCT02735707, NCT04505774, NCT04359277, NCT04372589_Critical | 1 | 3 | 1 | 1 | 2 | 4 |
| NCT04372589, NCT04505774, NCT02735707, NCT04359277_Noncritical | 1 | 3 | 1 | 1 | 2 | 4 |
| NCT02735707, NCT04505774, NCT04359277, NCT04372589 | 1 | 3 | 1 | 1 | 2 | 4 |
| NCT04381884 | 1 | 3 | 1 | 1 | 1 | 4 |
| NCT04381936_Dex | 1 | 3 | 1 | 1 | 1 | 4 |
| NCT04381936_Hydro | 1 | 3 | 1 | 1 | 1 | 4 |
| NCT04381936_Lopi | 1 | 3 | 1 | 1 | 1 | 4 |
| NCT04381936_Azi | 1 | 3 | 1 | 1 | 1 | 4 |
| NCT04381936 | 1 | 3 | 1 | 1 | 1 | 4 |
| NCT04381936_CP | 1 | 3 | 1 | 1 | 1 | 4 |
| NCT04381936_Toci | 1 | 3 | 1 | 1 | 1 | 4 |
| NCT04381962 | 1 | 3 | 1 | 1 | 1 | 4 |
| NCT04383535 | 1 | 1 | 1 | 1 | 1 | 1 |
| NCT04384380 | 1 | 3 | 1 | 1 | 2 | 4 |
| NCT04385095 | 1 | 1 | 4 | 1 | 1 | 4 |
| NCT04392973 | 1 | 3 | 2 | 1 | 2 | 4 |
| NCT04394377 | 1 | 3 | 1 | 1 | 1 | 4 |
| NCT04401579 | 1 | 1 | 1 | 1 | 1 | 1 |
| NCT04403100 | 1 | 1 | 3 | 1 | 1 | 4 |
| NCT04403555 | 1 | 3 | 1 | 1 | 2 | 4 |
| NCT04403685 | 1 | 3 | 1 | 1 | 1 | 4 |
| NCT04405843 | 1 | 1 | 1 | 1 | 1 | 1 |
| NCT04420247 | 1 | 3 | 4 | 1 | 2 | 4 |
| NCT04427501_P2 | 1 | 1 | 1 | 1 | 1 | 1 |
| NCT04427501_P3 | 1 | 1 | 1 | 1 | 1 | 1 |
| NCT04427501 | 1 | 1 | 1 | 1 | 1 | 1 |
| NCT04449718 | 2 | 1 | 1 | 1 | 1 | 1 |
| NCT04457609 | 1 | 1 | 1 | 1 | 1 | 1 |
| NCT04469114 | 1 | 1 | 1 | 1 | 1 | 1 |
| NCT04479163 | 1 | 1 | 1 | 1 | 1 | 1 |
| NCT04494984 | 1 | 1 | 1 | 1 | 1 | 1 |
| NCT04501978 | 1 | 1 | 1 | 1 | 1 | 1 |
| NCT04519385 | 1 | 3 | 1 | 1 | 2 | 4 |
| NCT04521309 | 1 | 3 | 1 | 1 | 1 | 4 |
| NCT04529525 | 1 | 1 | 1 | 1 | 1 | 1 |
| NCT04542941 | 1 | 3 | 1 | 1 | 1 | 4 |
| NCT04547660 | 1 | 3 | 1 | 1 | 1 | 4 |
| NCT04622891 | 1 | 3 | 1 | 1 | 2 | 4 |
| NCT04646109 | 4 | 3 | 1 | 1 | 1 | 4 |
| RBR-8h7q82 | 2 | 1 | 1 | 1 | 1 | 1 |
| RBR949z6v | 1 | 3 | 1 | 1 | 1 | 4 |
| Sakoulas_IVIG | 1 | 3 | 1 | 1 | 2 | 4 |
| VD_Elamir | 4 | 3 | 1 | 1 | 2 | 4 |

## **Table S6. Evaluation of risk of bias (mechanical ventilation) (1: low; 2: probably low; 3: probably high, 4: high).**

| **Registration Number** | **Randomization** | **Deviations from the intended intervention** | **Missing outcome data** | **Measurement of outcome** | **Selection of the reported results** | **Final** |
| --- | --- | --- | --- | --- | --- | --- |
| ChiCTR2000029308 | 1 | 3 | 1 | 1 | 1 | 4 |
| ChiCTR2000029544 | 1 | 3 | 1 | 1 | 1 | 4 |
| ChiCTR2000030007 | 1 | 3 | 1 | 1 | 2 | 4 |
| CTRI-2020-04-024479 | 4 | 3 | 1 | 1 | 1 | 4 |
| CTRI202004024775 | 1 | 3 | 1 | 1 | 1 | 4 |
| CTRI-2020-05-025114 | 3 | 3 | 1 | 1 | 2 | 4 |
| CTRI-2020-05-025369 | 1 | 3 | 1 | 1 | 2 | 4 |
| CTRI-2020-06-026222 | 2 | 3 | 1 | 1 | 2 | 4 |
| CTRI-2020-08-027225 | 1 | 1 | 1 | 1 | 1 | 1 |
| EudraCT2020-001236-10 | 1 | 1 | 1 | 1 | 1 | 1 |
| EudraCT202000193437 | 1 | 3 | 1 | 1 | 1 | 4 |
| IRCT20100228003449N27 | 1 | 3 | 4 | 1 | 2 | 4 |
| IRCT20100228003449N28 | 1 | 4 | 3 | 1 | 2 | 4 |
| IRCT20111224008507N3 | 1 | 1 | 2 | 1 | 2 | 1 |
| IRCT20151227025726N17 | 4 | 3 | 1 | 1 | 3 | 4 |
| IRCT20151227025726N20 | 4 | 3 | 1 | 1 | 3 | 4 |
| IRCT20200128046294N2 | 3 | 4 | 1 | 1 | 3 | 4 |
| IRCT20200204046369N1 | 1 | 1 | 1 | 1 | 1 | 1 |
| IRCT202003117046797N4 | 3 | 3 | 1 | 1 | 1 | 4 |
| IRCT20200318046812N1 | 1 | 3 | 2 | 1 | 1 | 4 |
| IRCT20200504047298N3 | 2 | 1 | 1 | 1 | 2 | 1 |
| ISRCTN21085622 | 1 | 3 | 1 | 1 | 2 | 4 |
| ISRCTN59048638 | 2 | 1 | 3 | 1 | 1 | 4 |
| ISRCTN86534580_Azi | 1 | 3 | 1 | 1 | 1 | 4 |
| ISRCTN86534580_Budesonide | 1 | 3 | 1 | 1 | 1 | 4 |
| ISRCTN86534580_Doxy | 1 | 3 | 1 | 1 | 1 | 4 |
| ISRCTN86534580 | 1 | 3 | 1 | 1 | 1 | 4 |
| Mahajan_Remdesivir | 2 | 3 | 1 | 1 | 3 | 4 |
| NCT02735707_toci | 1 | 3 | 1 | 1 | 1 | 4 |
| NCT04257656 | 1 | 1 | 1 | 1 | 2 | 1 |
| NCT04280705 | 1 | 1 | 1 | 1 | 1 | 1 |
| NCT04292730 | 1 | 4 | 2 | 1 | 1 | 4 |
| NCT04304053 | 1 | 3 | 4 | 1 | 2 | 4 |
| NCT04310228 | 3 | 3 | 1 | 1 | 2 | 4 |
| NCT04315948 | 1 | 3 | 1 | 1 | 1 | 4 |
| NCT04320615 | 1 | 3 | 1 | 1 | 1 | 4 |
| NCT04321096 | 1 | 1 | 1 | 1 | 1 | 1 |
| NCT04321278 | 1 | 3 | 1 | 1 | 1 | 4 |
| NCT04322123 | 1 | 3 | 1 | 1 | 1 | 4 |
| NCT04322396 | 1 | 1 | 1 | 1 | 1 | 1 |
| NCT04322682 | 2 | 1 | 1 | 1 | 1 | 1 |
| NCT04325893 | 2 | 1 | 1 | 1 | 1 | 1 |
| NCT04326790 | 1 | 3 | 1 | 1 | 1 | 4 |
| NCT04327388 | 1 | 1 | 1 | 1 | 1 | 1 |
| NCT04327401 | 1 | 3 | 1 | 1 | 1 | 4 |
| NCT04329611 | 1 | 1 | 1 | 1 | 1 | 1 |
| NCT04329832 | 1 | 4 | 1 | 1 | 1 | 4 |
| NCT04331808 | 1 | 3 | 1 | 1 | 2 | 4 |
| NCT04332991 | 1 | 1 | 1 | 1 | 1 | 1 |
| NCT04338009 | 1 | 3 | 1 | 1 | 2 | 4 |
| NCT04343729 | 1 | 1 | 4 | 1 | 1 | 4 |
| NCT04343768 | 1 | 3 | 1 | 1 | 1 | 4 |
| NCT04351295 | 3 | 3 | 1 | 1 | 1 | 4 |
| NCT04353336 | 3 | 3 | 1 | 1 | 2 | 4 |
| NCT04353596 | 3 | 3 | 1 | 1 | 2 | 4 |
| NCT04356534 | 2 | 3 | 1 | 1 | 2 | 4 |
| NCT04356937 | 2 | 1 | 1 | 1 | 1 | 1 |
| NCT04362813 | 1 | 1 | 1 | 1 | 1 | 1 |
| NCT04364893 | 1 | 3 | 1 | 1 | 1 | 4 |
| NCT04369742 | 2 | 1 | 1 | 1 | 1 | 1 |
| NCT04381884 | 1 | 3 | 1 | 1 | 2 | 4 |
| NCT04381936_Dex | 1 | 3 | 1 | 1 | 1 | 4 |
| NCT04381936_Hydro | 1 | 3 | 1 | 1 | 1 | 4 |
| NCT04381936_Lopi | 1 | 3 | 1 | 1 | 1 | 4 |
| NCT04381936_Azi | 1 | 3 | 1 | 1 | 1 | 4 |
| NCT04381936 | 1 | 3 | 1 | 1 | 1 | 4 |
| NCT04381936_CP | 1 | 3 | 1 | 1 | 1 | 4 |
| NCT04381936_Toci | 1 | 3 | 1 | 1 | 1 | 4 |
| NCT04381962 | 1 | 3 | 1 | 1 | 1 | 4 |
| NCT04383535 | 1 | 1 | 1 | 1 | 1 | 1 |
| NCT04385095 | 1 | 1 | 4 | 1 | 1 | 4 |
| NCT04392973 | 1 | 3 | 2 | 1 | 2 | 4 |
| NCT04401579 | 1 | 1 | 1 | 1 | 1 | 1 |
| NCT04403555 | 1 | 3 | 1 | 1 | 3 | 4 |
| NCT04403685 | 1 | 3 | 1 | 1 | 1 | 4 |
| NCT04405843 | 1 | 1 | 1 | 1 | 1 | 1 |
| NCT04420247 | 1 | 3 | 4 | 1 | 2 | 4 |
| NCT04449718 | 2 | 1 | 1 | 1 | 2 | 1 |
| NCT04469114 | 1 | 1 | 1 | 1 | 1 | 1 |
| NCT04479163 | 1 | 1 | 1 | 1 | 1 | 1 |
| NCT04494984 | 1 | 1 | 1 | 1 | 2 | 1 |
| NCT04501978 | 1 | 1 | 1 | 1 | 1 | 1 |
| NCT04521309 | 1 | 3 | 1 | 1 | 2 | 4 |
| RBR-8h7q82 | 2 | 1 | 1 | 1 | 1 | 1 |
| Sakoulas_IVIG | 1 | 3 | 1 | 1 | 2 | 4 |
| Tolouian_Bromhexine | 2 | 3 | 3 | 1 | 2 | 4 |

## **Table S7. Evaluation of risk of bias (discharge) (1: low; 2: probably low; 3: probably high, 4: high).**

| **Registration Number** | **Randomization** | **Deviations from the intended intervention** | **Missing outcome data** | **Measurement of outcome** | **Selection of the reported results** | **Final** |
| --- | --- | --- | --- | --- | --- | --- |
| ChiCTR2000029308 | 1 | 3 | 1 | 1 | 1 | 4 |
| ChiCTR2000029544 | 1 | 3 | 1 | 1 | 2 | 4 |
| ChiCTR2000029757 | 2 | 3 | 1 | 1 | 1 | 4 |
| ChiCTR2000031494 | 4 | 3 | 1 | 1 | 2 | 4 |
| CTRI-2020-05-025114 | 3 | 3 | 1 | 1 | 1 | 4 |
| CTRI-2020-08-027225 | 1 | 1 | 1 | 1 | 1 | 1 |
| IRCT20100228003449N27 | 1 | 3 | 4 | 1 | 2 | 4 |
| IRCT20100228003449N28 | 1 | 4 | 3 | 1 | 2 | 4 |
| NCT02735707_toci | 1 | 3 | 1 | 1 | 1 | 4 |
| NCT04257656 | 1 | 1 | 1 | 1 | 1 | 1 |
| NCT04280705 | 1 | 1 | 1 | 1 | 1 | 1 |
| NCT04288102 | 1 | 1 | 1 | 1 | 1 | 1 |
| NCT04292730 | 1 | 4 | 2 | 1 | 1 | 4 |
| NCT04315948_Discovery | 1 | 3 | 1 | 1 | 1 | 4 |
| NCT04320615 | 1 | 3 | 1 | 1 | 1 | 4 |
| NCT04321096 | 1 | 1 | 1 | 1 | 1 | 1 |
| NCT04321278 | 1 | 3 | 1 | 1 | 1 | 4 |
| NCT04322123 | 1 | 3 | 1 | 1 | 1 | 4 |
| NCT04322396 | 1 | 1 | 1 | 1 | 1 | 1 |
| NCT04325893 | 2 | 1 | 1 | 1 | 1 | 1 |
| NCT04327388 | 1 | 1 | 1 | 1 | 1 | 1 |
| NCT04327401 | 1 | 3 | 1 | 1 | 1 | 4 |
| NCT04329832 | 1 | 4 | 1 | 1 | 1 | 4 |
| NCT04331808 | 1 | 3 | 1 | 1 | 1 | 4 |
| NCT04332991 | 1 | 1 | 1 | 1 | 1 | 1 |
| NCT04346355 | 1 | 3 | 1 | 1 | 2 | 4 |
| NCT04350593 | 1 | 1 | 1 | 1 | 1 | 1 |
| NCT04356937 | 2 | 1 | 1 | 1 | 1 | 1 |
| NCT04359810 | 1 | 1 | 1 | 1 | 1 | 1 |
| NCT04362813 | 1 | 1 | 1 | 1 | 1 | 1 |
| NCT04369742 | 2 | 1 | 1 | 1 | 1 | 1 |
| NCT04381936_Dex | 1 | 3 | 1 | 1 | 2 | 4 |
| NCT04381936_Hydro | 1 | 3 | 1 | 1 | 2 | 4 |
| NCT04381936_Lopi | 1 | 3 | 1 | 1 | 2 | 4 |
| NCT04381936_Azi | 1 | 3 | 1 | 1 | 2 | 4 |
| NCT04381936 | 1 | 3 | 1 | 1 | 2 | 4 |
| NCT04381936_CP | 1 | 3 | 1 | 1 | 1 | 4 |
| NCT04381936_Toci | 1 | 3 | 1 | 1 | 2 | 4 |
| NCT04383535 | 1 | 1 | 1 | 1 | 1 | 1 |
| NCT04385095 | 1 | 1 | 4 | 1 | 1 | 4 |
| NCT04392973 | 1 | 3 | 2 | 1 | 2 | 4 |
| NCT04401579 | 1 | 1 | 1 | 1 | 1 | 1 |
| NCT04403685 | 1 | 3 | 1 | 1 | 1 | 4 |
| NCT04405843 | 1 | 1 | 1 | 1 | 1 | 1 |
| NCT04420247 | 1 | 3 | 4 | 1 | 2 | 4 |
| NCT04434248 | 3 | 4 | 1 | 1 | 2 | 4 |
| NCT04469114 | 1 | 1 | 1 | 1 | 1 | 1 |
| NCT04501978 | 1 | 1 | 1 | 1 | 1 | 1 |
| NCT04547660 | 1 | 3 | 1 | 1 | 1 | 4 |

## **Table S8. Evaluation of risk of bias (viral clearance) (1: low; 2: probably low; 3: probably high, 4: high).**

| **Registration Number** | **Randomization** | **Deviations from the intended intervention** | **Missing outcome data** | **Measurement of outcome** | **Selection of the reported results** | **Final** |
| --- | --- | --- | --- | --- | --- | --- |
| Brazilian 4.173.074 | 4 | 1 | 1 | 1 | 2 | 4 |
| ChiCTR2000029544 | 1 | 3 | 1 | 1 | 1 | 4 |
| ChiCTR2000029757 | 1 | 3 | 2 | 1 | 1 | 4 |
| ChiCTR2000029868 | 1 | 3 | 1 | 1 | 1 | 4 |
| CTRI202004024775 | 1 | 3 | 4 | 1 | 2 | 4 |
| CTRI-2020-05-025114 | 3 | 3 | 1 | 1 | 1 | 4 |
| CTRI-2020-08-027225 | 1 | 1 | 1 | 1 | 1 | 1 |
| ISRCTN40302986 | 1 | 1 | 1 | 1 | 2 | 1 |
| Iver_Ahmed | 2 | 1 | 1 | 1 | 2 | 1 |
| NCT04252885 | 1 | 3 | 1 | 1 | 1 | 4 |
| NCT04257656 | 1 | 1 | 1 | 1 | 1 | 1 |
| NCT04261517 | 3 | 3 | 1 | 1 | 1 | 4 |
| NCT04315948_Discovery | 1 | 3 | 4 | 1 | 1 | 4 |
| NCT04325893 | 2 | 1 | 4 | 1 | 1 | 4 |
| NCT04333589 | 1 | 3 | 1 | 1 | 1 | 4 |
| NCT04343729 | 1 | 1 | 4 | 1 | 1 | 4 |
| NCT04348409 | 2 | 1 | 1 | 1 | 2 | 1 |
| NCT04349241 | 3 | 3 | 1 | 1 | 1 | 4 |
| NCT04349592 | 1 | 1 | 1 | 1 | 1 | 1 |
| NCT04354428 | 2 | 1 | 1 | 1 | 1 | 1 |
| NCT04369742 | 2 | 1 | 4 | 1 | 1 | 4 |
| NCT04384380 | 2 | 3 | 1 | 1 | 1 | 4 |
| NCT04390022 | 1 | 1 | 1 | 1 | 1 | 1 |
| NCT04392973 | 1 | 3 | 4 | 1 | 1 | 4 |
| NCT04427501_P2 | 1 | 1 | 1 | 1 | 1 | 1 |
| NCT04434248 | 3 | 4 | 1 | 1 | 1 | 4 |
| NCT04491994 | 4 | 3 | 1 | 1 | 4 | 4 |
| NCT04523831 | 1 | 1 | 1 | 1 | 2 | 1 |
| NCT04542941 | 1 | 3 | 1 | 1 | 1 | 4 |
| NCT04547660 | 1 | 3 | 4 | 1 | 1 | 4 |
| NCT04552483 | 1 | 1 | 4 | 1 | 2 | 4 |
| NCT04716569 | 4 | 3 | 1 | 1 | 2 | 4 |

## **Table S9. Network meta-analysis results of the primary analysis (log odds ratio and corresponding 95% equal-tailed Bayesian credible intervals) (mortality).**

|  |  | **Direct** | | | | | **Indirect** | | | | | **Network** | | | | |
| --- | --- | --- | --- | --- | --- | --- | --- | --- | --- | --- | --- | --- | --- | --- | --- | --- |
| **Treatment 1** | **Treatment 2** | **EST^*^** | **LCrI^*^** | **UCrI^*^** | **Evidence**^†^ | **Reason** | **EST** | **LCrI** | **UCrI** | **Evidence** | **Reason** | **EST** | **LCrI** | **UCrI** | **Evidence** | **Reason** |
| aceis/arbs | soc | 0.22 | -0.29 | 0.73 | 3 | RoB; | NA | NA | NA | NA | NA | 0.22 | -0.29 | 0.73 | 2 | Imprecision |
| ammonium chloride | soc | 0.04 | -0.39 | 0.73 | 4 | NA | NA | NA | NA | NA | NA | 0.04 | -0.39 | 0.73 | 3 | Imprecision |
| azithromycin | soc | -0.05 | -0.16 | 0.06 | 3 | RoB; | -0.21 | -1.24 | 0.27 | 3 | NA | -0.08 | -0.18 | 0.03 | 2 | Imprecision |
| bamlanivimab | soc | -0.51 | -1.07 | 0.08 | 4 | NA | NA | NA | NA | NA | NA | -0.51 | -1.07 | 0.08 | 3 | Imprecision |
| baricitinib + remdesivir | soc | NA | NA | NA | NA | NA | -0.50 | -1.06 | 0.03 | 3 | NA | -0.50 | -1.06 | 0.03 | 2 | Imprecision |
| budesonide | soc | -0.05 | -0.46 | 0.53 | 3 | RoB; | NA | NA | NA | NA | NA | -0.05 | -0.46 | 0.53 | 2 | Imprecision |
| camostat mesilate | soc | 0.03 | -1.19 | 1.39 | 4 | NA | NA | NA | NA | NA | NA | 0.03 | -1.19 | 1.39 | 3 | Imprecision |
| canakinumab | soc | -0.26 | -0.78 | 0.13 | 4 | NA | NA | NA | NA | NA | NA | -0.26 | -0.78 | 0.13 | 3 | Imprecision |
| chloroquine | soc | 0.17 | -0.31 | 0.74 | 3 | RoB; | 0.09 | -0.45 | 0.54 | 3 | NA | 0.12 | -0.32 | 0.52 | 2 | Imprecision |
| colchicine | soc | -0.73 | -1.82 | 0.24 | 3 | RoB; | NA | NA | NA | NA | NA | -0.73 | -1.82 | 0.24 | 1 | Severe Imprecision |
| convalescent plasma | soc | -0.03 | -0.11 | 0.05 | 3 | RoB; | NA | NA | NA | NA | NA | -0.03 | -0.11 | 0.05 | 2 | Imprecision |
| dapagliflozin | soc | -0.30 | -0.73 | 0.12 | 4 | NA | NA | NA | NA | NA | NA | -0.30 | -0.73 | 0.12 | 3 | Imprecision |
| dexamethasone | soc | 0.00 | -0.12 | 0.11 | 3 | RoB; | -0.17 | -0.59 | 0.21 | 3 | NA | -0.03 | -0.14 | 0.08 | 2 | Imprecision |
| doxycycline | soc | -0.04 | -0.52 | 0.56 | 3 | RoB; | NA | NA | NA | NA | NA | -0.04 | -0.52 | 0.56 | 2 | Imprecision |
| favipiravir | soc | -0.08 | -0.73 | 0.39 | 3 | RoB; | -0.01 | -0.41 | 0.42 | 3 | NA | -0.03 | -0.40 | 0.35 | 2 | Imprecision |
| hydrocortisone | soc | -0.22 | -0.48 | 0.01 | 4 | NA | NA | NA | NA | NA | NA | -0.22 | -0.48 | 0.01 | 3 | Imprecision |
| hydroxychloroquine | soc | 0.17 | 0.07 | 0.27 | 3 | RoB; | 0.30 | -0.10 | 0.89 | 3 | NA | 0.16 | 0.05 | 0.26 | 3 | NA |
| hydroxychloroquine + azithromycin | soc | -0.61 | -1.96 | 0.50 | 4 | NA | NA | NA | NA | NA | NA | -0.61 | -1.96 | 0.50 | 2 | Severe Imprecision |
| hydroxychloroquine + favipiravir | soc | 0.24 | -0.40 | 0.85 | 3 | RoB; | NA | NA | NA | NA | NA | 0.24 | -0.40 | 0.85 | 2 | Imprecision |
| hydroxychloroquine + lopinavir/ritonavir | soc | 0.39 | -0.23 | 1.02 | 3 | RoB; | NA | NA | NA | NA | NA | 0.39 | -0.23 | 1.02 | 2 | Imprecision |
| imatinib | soc | -0.59 | -1.12 | -0.09 | 4 | NA | NA | NA | NA | NA | NA | -0.59 | -1.12 | -0.09 | 4 | NA |
| inm005 | soc | -0.56 | -1.14 | 0.01 | 4 | NA | NA | NA | NA | NA | NA | -0.56 | -1.14 | 0.01 | 3 | Imprecision |
| interferon beta | soc | 0.00 | -0.16 | 0.15 | 2 | RoB;Inconsistency; | NA | NA | NA | NA | NA | 0.00 | -0.16 | 0.15 | 1 | Imprecision |
| intravenous immunoglobulin | soc | -0.73 | -1.36 | -0.12 | 3 | RoB; | NA | NA | NA | NA | NA | -0.73 | -1.36 | -0.12 | 3 | NA |
| ivermectin | soc | -0.48 | -1.06 | 0.07 | 4 | NA | -0.50 | -1.08 | 0.06 | 2 | NA | -0.49 | -0.99 | 0.00 | 3 | Imprecision |
| lopinavir/ritonavir | soc | -0.01 | -0.11 | 0.09 | 3 | RoB; | -0.24 | -1.18 | 0.27 | 3 | NA | -0.02 | -0.11 | 0.08 | 2 | Imprecision |
| mesenchymal stem cells | soc | -0.26 | -1.18 | 0.14 | 4 | NA | NA | NA | NA | NA | NA | -0.26 | -1.18 | 0.14 | 3 | Imprecision |
| methylprednisolone | soc | -0.07 | -0.36 | 0.24 | 3 | RoB; | -0.16 | -0.82 | 0.19 | 3 | NA | -0.14 | -0.43 | 0.14 | 2 | Imprecision |
| recombinant human gcsf | soc | -0.25 | -1.19 | 0.18 | 3 | RoB; | NA | NA | NA | NA | NA | -0.25 | -1.19 | 0.18 | 2 | Imprecision |
| remdesivir | soc | -0.05 | -0.17 | 0.08 | 3 | RoB; | NA | NA | NA | NA | NA | -0.05 | -0.17 | 0.08 | 2 | Imprecision |
| sarilumab | soc | -0.26 | -0.66 | 0.06 | 3 | RoB; | NA | NA | NA | NA | NA | -0.26 | -0.66 | 0.06 | 2 | Imprecision |
| sofosbuvir + daclatasvir | soc | -0.14 | -0.80 | 0.24 | 3 | RoB; | NA | NA | NA | NA | NA | -0.14 | -0.80 | 0.24 | 2 | Imprecision |
| sulodexide | soc | -0.16 | -0.93 | 0.31 | 3 | RoB; | NA | NA | NA | NA | NA | -0.16 | -0.93 | 0.31 | 2 | Imprecision |
| therapeutic anticoagulation | soc | -0.05 | -0.22 | 0.12 | 3 | RoB; | NA | NA | NA | NA | NA | -0.05 | -0.22 | 0.12 | 2 | Imprecision |
| tocilizumab | soc | -0.17 | -0.28 | -0.06 | 3 | RoB; | 0.11 | -0.31 | 0.78 | 2 | Intransitivity | -0.16 | -0.27 | -0.05 | 3 | NA |
| tofacitinib | soc | -0.27 | -0.90 | 0.15 | 4 | NA | NA | NA | NA | NA | NA | -0.27 | -0.90 | 0.15 | 3 | Imprecision |
| vitamin c | soc | -0.27 | -1.12 | 0.60 | 3 | RoB; | NA | NA | NA | NA | NA | -0.27 | -1.12 | 0.60 | 2 | Imprecision |
| vitamin d3 | soc | -0.34 | -1.08 | 0.37 | 3 | RoB; | NA | NA | NA | NA | NA | -0.34 | -1.08 | 0.37 | 2 | Imprecision |
| ammonium chloride | aceis/arbs | NA | NA | NA | NA | NA | -0.16 | -0.84 | 0.65 | 3 | NA | -0.16 | -0.84 | 0.65 | 2 | Imprecision |
| azithromycin | aceis/arbs | NA | NA | NA | NA | NA | -0.30 | -0.82 | 0.22 | 3 | NA | -0.30 | -0.82 | 0.22 | 2 | Imprecision |
| bamlanivimab | aceis/arbs | NA | NA | NA | NA | NA | -0.72 | -1.49 | 0.05 | 3 | NA | -0.72 | -1.49 | 0.05 | 2 | Imprecision |
| baricitinib + remdesivir | aceis/arbs | NA | NA | NA | NA | NA | -0.72 | -1.47 | 0.02 | 3 | NA | -0.72 | -1.47 | 0.02 | 2 | Imprecision |
| budesonide | aceis/arbs | NA | NA | NA | NA | NA | -0.26 | -0.93 | 0.49 | 3 | NA | -0.26 | -0.93 | 0.49 | 2 | Imprecision |
| camostat mesilate | aceis/arbs | NA | NA | NA | NA | NA | -0.19 | -1.53 | 1.27 | 3 | NA | -0.19 | -1.53 | 1.27 | 1 | Severe Imprecision |
| canakinumab | aceis/arbs | NA | NA | NA | NA | NA | -0.49 | -1.20 | 0.16 | 3 | NA | -0.49 | -1.20 | 0.16 | 2 | Imprecision |
| chloroquine | aceis/arbs | NA | NA | NA | NA | NA | -0.11 | -0.77 | 0.54 | 3 | NA | -0.11 | -0.77 | 0.54 | 2 | Imprecision |
| colchicine | aceis/arbs | NA | NA | NA | NA | NA | -0.94 | -2.14 | 0.16 | 3 | NA | -0.94 | -2.14 | 0.16 | 1 | Severe Imprecision |
| convalescent plasma | aceis/arbs | NA | NA | NA | NA | NA | -0.25 | -0.77 | 0.26 | 3 | NA | -0.25 | -0.77 | 0.26 | 2 | Imprecision |
| dapagliflozin | aceis/arbs | NA | NA | NA | NA | NA | -0.52 | -1.19 | 0.15 | 3 | NA | -0.52 | -1.19 | 0.15 | 2 | Imprecision |
| dexamethasone | aceis/arbs | NA | NA | NA | NA | NA | -0.25 | -0.77 | 0.27 | 3 | NA | -0.25 | -0.77 | 0.27 | 2 | Imprecision |
| doxycycline | aceis/arbs | NA | NA | NA | NA | NA | -0.25 | -0.96 | 0.51 | 3 | NA | -0.25 | -0.96 | 0.51 | 2 | Imprecision |
| favipiravir | aceis/arbs | NA | NA | NA | NA | NA | -0.25 | -0.88 | 0.38 | 3 | NA | -0.25 | -0.88 | 0.38 | 2 | Imprecision |
| hydrocortisone | aceis/arbs | NA | NA | NA | NA | NA | -0.44 | -1.01 | 0.12 | 3 | NA | -0.44 | -1.01 | 0.12 | 2 | Imprecision |
| hydroxychloroquine | aceis/arbs | NA | NA | NA | NA | NA | -0.06 | -0.59 | 0.45 | 3 | NA | -0.06 | -0.59 | 0.45 | 2 | Imprecision |
| hydroxychloroquine + azithromycin | aceis/arbs | NA | NA | NA | NA | NA | -0.83 | -2.29 | 0.40 | 3 | NA | -0.83 | -2.29 | 0.40 | 1 | Severe Imprecision |
| hydroxychloroquine + favipiravir | aceis/arbs | NA | NA | NA | NA | NA | 0.02 | -0.79 | 0.82 | 3 | NA | 0.02 | -0.79 | 0.82 | 2 | Imprecision |
| hydroxychloroquine + lopinavir/ritonavir | aceis/arbs | NA | NA | NA | NA | NA | 0.17 | -0.64 | 0.98 | 3 | NA | 0.17 | -0.64 | 0.98 | 2 | Imprecision |
| imatinib | aceis/arbs | NA | NA | NA | NA | NA | -0.81 | -1.56 | -0.10 | 3 | NA | -0.81 | -1.56 | -0.10 | 3 | NA |
| inm005 | aceis/arbs | NA | NA | NA | NA | NA | -0.78 | -1.55 | -0.01 | 3 | NA | -0.78 | -1.55 | -0.01 | 1 | Severe Imprecision |
| interferon beta | aceis/arbs | NA | NA | NA | NA | NA | -0.22 | -0.75 | 0.31 | 2 | NA | -0.22 | -0.75 | 0.31 | 1 | Imprecision |
| intravenous immunoglobulin | aceis/arbs | NA | NA | NA | NA | NA | -0.95 | -1.76 | -0.16 | 3 | NA | -0.95 | -1.76 | -0.16 | 3 | NA |
| ivermectin | aceis/arbs | NA | NA | NA | NA | NA | -0.71 | -1.43 | 0.00 | 3 | NA | -0.71 | -1.43 | 0.00 | 2 | Imprecision |
| lopinavir/ritonavir | aceis/arbs | NA | NA | NA | NA | NA | -0.24 | -0.76 | 0.28 | 3 | NA | -0.24 | -0.76 | 0.28 | 2 | Imprecision |
| mesenchymal stem cells | aceis/arbs | NA | NA | NA | NA | NA | -0.52 | -1.52 | 0.18 | 3 | NA | -0.52 | -1.52 | 0.18 | 1 | Severe Imprecision |
| methylprednisolone | aceis/arbs | NA | NA | NA | NA | NA | -0.36 | -0.94 | 0.22 | 3 | NA | -0.36 | -0.94 | 0.22 | 2 | Imprecision |
| recombinant human gcsf | aceis/arbs | NA | NA | NA | NA | NA | -0.50 | -1.53 | 0.21 | 3 | NA | -0.50 | -1.53 | 0.21 | 1 | Severe Imprecision |
| remdesivir | aceis/arbs | NA | NA | NA | NA | NA | -0.27 | -0.79 | 0.26 | 3 | NA | -0.27 | -0.79 | 0.26 | 2 | Imprecision |
| sarilumab | aceis/arbs | NA | NA | NA | NA | NA | -0.49 | -1.13 | 0.12 | 3 | NA | -0.49 | -1.13 | 0.12 | 2 | Imprecision |
| sofosbuvir + daclatasvir | aceis/arbs | NA | NA | NA | NA | NA | -0.38 | -1.17 | 0.28 | 3 | NA | -0.38 | -1.17 | 0.28 | 2 | Imprecision |
| sulodexide | aceis/arbs | NA | NA | NA | NA | NA | -0.40 | -1.28 | 0.31 | 3 | NA | -0.40 | -1.28 | 0.31 | 2 | Imprecision |
| therapeutic anticoagulation | aceis/arbs | NA | NA | NA | NA | NA | -0.27 | -0.81 | 0.26 | 3 | NA | -0.27 | -0.81 | 0.26 | 2 | Imprecision |
| tocilizumab | aceis/arbs | NA | NA | NA | NA | NA | -0.38 | -0.90 | 0.14 | 3 | NA | -0.38 | -0.90 | 0.14 | 2 | Imprecision |
| tofacitinib | aceis/arbs | NA | NA | NA | NA | NA | -0.51 | -1.29 | 0.17 | 3 | NA | -0.51 | -1.29 | 0.17 | 2 | Imprecision |
| vitamin c | aceis/arbs | NA | NA | NA | NA | NA | -0.49 | -1.48 | 0.52 | 3 | NA | -0.49 | -1.48 | 0.52 | 2 | Imprecision |
| vitamin d3 | aceis/arbs | NA | NA | NA | NA | NA | -0.56 | -1.46 | 0.31 | 3 | NA | -0.56 | -1.46 | 0.31 | 2 | Imprecision |
| azithromycin | ammonium chloride | NA | NA | NA | NA | NA | -0.12 | -0.81 | 0.32 | 3 | NA | -0.12 | -0.81 | 0.32 | 2 | Imprecision |
| bamlanivimab | ammonium chloride | NA | NA | NA | NA | NA | -0.56 | -1.40 | 0.15 | 4 | NA | -0.56 | -1.40 | 0.15 | 3 | Imprecision |
| baricitinib + remdesivir | ammonium chloride | NA | NA | NA | NA | NA | -0.56 | -1.40 | 0.14 | 3 | NA | -0.56 | -1.40 | 0.14 | 2 | Imprecision |
| budesonide | ammonium chloride | NA | NA | NA | NA | NA | -0.09 | -0.84 | 0.56 | 3 | NA | -0.09 | -0.84 | 0.56 | 2 | Imprecision |
| camostat mesilate | ammonium chloride | NA | NA | NA | NA | NA | -0.03 | -1.40 | 1.41 | 4 | NA | -0.03 | -1.40 | 1.41 | 3 | Imprecision |
| canakinumab | ammonium chloride | NA | NA | NA | NA | NA | -0.30 | -1.23 | 0.25 | 4 | NA | -0.30 | -1.23 | 0.25 | 3 | Imprecision |
| chloroquine | ammonium chloride | NA | NA | NA | NA | NA | 0.08 | -0.77 | 0.64 | 3 | NA | 0.08 | -0.77 | 0.64 | 2 | Imprecision |
| colchicine | ammonium chloride | NA | NA | NA | NA | NA | -0.79 | -2.03 | 0.29 | 3 | NA | -0.79 | -2.03 | 0.29 | 1 | Severe Imprecision |
| convalescent plasma | ammonium chloride | NA | NA | NA | NA | NA | -0.07 | -0.76 | 0.35 | 3 | NA | -0.07 | -0.76 | 0.35 | 2 | Imprecision |
| dapagliflozin | ammonium chloride | NA | NA | NA | NA | NA | -0.35 | -1.13 | 0.26 | 4 | NA | -0.35 | -1.13 | 0.26 | 3 | Imprecision |
| dexamethasone | ammonium chloride | NA | NA | NA | NA | NA | -0.07 | -0.75 | 0.38 | 3 | NA | -0.07 | -0.75 | 0.38 | 2 | Imprecision |
| doxycycline | ammonium chloride | NA | NA | NA | NA | NA | -0.08 | -0.90 | 0.60 | 3 | NA | -0.08 | -0.90 | 0.60 | 2 | Imprecision |
| favipiravir | ammonium chloride | NA | NA | NA | NA | NA | -0.07 | -0.85 | 0.48 | 3 | NA | -0.07 | -0.85 | 0.48 | 2 | Imprecision |
| hydrocortisone | ammonium chloride | NA | NA | NA | NA | NA | -0.25 | -1.03 | 0.22 | 4 | NA | -0.25 | -1.03 | 0.22 | 3 | Imprecision |
| hydroxychloroquine | ammonium chloride | NA | NA | NA | NA | NA | 0.11 | -0.57 | 0.56 | 3 | NA | 0.11 | -0.57 | 0.56 | 2 | Imprecision |
| hydroxychloroquine + azithromycin | ammonium chloride | NA | NA | NA | NA | NA | -0.68 | -2.15 | 0.54 | 4 | NA | -0.68 | -2.15 | 0.54 | 2 | Severe Imprecision |
| hydroxychloroquine + favipiravir | ammonium chloride | NA | NA | NA | NA | NA | 0.19 | -0.79 | 0.95 | 3 | NA | 0.19 | -0.79 | 0.95 | 2 | Imprecision |
| hydroxychloroquine + lopinavir/ritonavir | ammonium chloride | NA | NA | NA | NA | NA | 0.32 | -0.52 | 1.09 | 3 | NA | 0.32 | -0.52 | 1.09 | 2 | Imprecision |
| imatinib | ammonium chloride | NA | NA | NA | NA | NA | -0.64 | -1.52 | 0.02 | 4 | NA | -0.64 | -1.52 | 0.02 | 2 | Severe Imprecision |
| inm005 | ammonium chloride | NA | NA | NA | NA | NA | -0.61 | -1.50 | 0.10 | 4 | NA | -0.61 | -1.50 | 0.10 | 2 | Severe Imprecision |
| interferon beta | ammonium chloride | NA | NA | NA | NA | NA | -0.04 | -0.73 | 0.41 | 2 | NA | -0.04 | -0.73 | 0.41 | 1 | Imprecision |
| intravenous immunoglobulin | ammonium chloride | NA | NA | NA | NA | NA | -0.79 | -1.68 | -0.03 | 3 | NA | -0.79 | -1.68 | -0.03 | 3 | NA |
| ivermectin | ammonium chloride | NA | NA | NA | NA | NA | -0.54 | -1.36 | 0.12 | 4 | NA | -0.54 | -1.36 | 0.12 | 3 | Imprecision |
| lopinavir/ritonavir | ammonium chloride | NA | NA | NA | NA | NA | -0.06 | -0.74 | 0.38 | 3 | NA | -0.06 | -0.74 | 0.38 | 2 | Imprecision |
| mesenchymal stem cells | ammonium chloride | NA | NA | NA | NA | NA | -0.29 | -1.58 | 0.14 | 4 | NA | -0.29 | -1.58 | 0.14 | 2 | Severe Imprecision |
| methylprednisolone | ammonium chloride | NA | NA | NA | NA | NA | -0.18 | -0.94 | 0.32 | 3 | NA | -0.18 | -0.94 | 0.32 | 2 | Imprecision |
| recombinant human gcsf | ammonium chloride | NA | NA | NA | NA | NA | -0.29 | -1.66 | 0.31 | 3 | NA | -0.29 | -1.66 | 0.31 | 1 | Severe Imprecision |
| remdesivir | ammonium chloride | NA | NA | NA | NA | NA | -0.09 | -0.78 | 0.36 | 3 | NA | -0.09 | -0.78 | 0.36 | 2 | Imprecision |
| sarilumab | ammonium chloride | NA | NA | NA | NA | NA | -0.30 | -1.14 | 0.22 | 3 | NA | -0.30 | -1.14 | 0.22 | 2 | Imprecision |
| sofosbuvir + daclatasvir | ammonium chloride | NA | NA | NA | NA | NA | -0.17 | -1.27 | 0.38 | 3 | NA | -0.17 | -1.27 | 0.38 | 2 | Imprecision |
| sulodexide | ammonium chloride | NA | NA | NA | NA | NA | -0.20 | -1.37 | 0.42 | 3 | NA | -0.20 | -1.37 | 0.42 | 2 | Imprecision |
| therapeutic anticoagulation | ammonium chloride | NA | NA | NA | NA | NA | -0.10 | -0.79 | 0.36 | 3 | NA | -0.10 | -0.79 | 0.36 | 2 | Imprecision |
| tocilizumab | ammonium chloride | NA | NA | NA | NA | NA | -0.20 | -0.89 | 0.24 | 3 | NA | -0.20 | -0.89 | 0.24 | 2 | Imprecision |
| tofacitinib | ammonium chloride | NA | NA | NA | NA | NA | -0.31 | -1.35 | 0.26 | 4 | NA | -0.31 | -1.35 | 0.26 | 3 | Imprecision |
| vitamin c | ammonium chloride | NA | NA | NA | NA | NA | -0.33 | -1.37 | 0.62 | 3 | NA | -0.33 | -1.37 | 0.62 | 2 | Imprecision |
| vitamin d3 | ammonium chloride | NA | NA | NA | NA | NA | -0.40 | -1.38 | 0.45 | 3 | NA | -0.40 | -1.38 | 0.45 | 2 | Imprecision |
| bamlanivimab | azithromycin | NA | NA | NA | NA | NA | -0.43 | -1.00 | 0.17 | 3 | NA | -0.43 | -1.00 | 0.17 | 2 | Imprecision |
| baricitinib + remdesivir | azithromycin | NA | NA | NA | NA | NA | -0.42 | -0.99 | 0.12 | 3 | NA | -0.42 | -0.99 | 0.12 | 2 | Imprecision |
| budesonide | azithromycin | 0.03 | -0.39 | 0.60 | 3 | RoB; | NA | NA | NA | NA | NA | 0.03 | -0.39 | 0.60 | 2 | Imprecision |
| camostat mesilate | azithromycin | NA | NA | NA | NA | NA | 0.11 | -1.12 | 1.47 | 3 | NA | 0.11 | -1.12 | 1.47 | 2 | Imprecision |
| canakinumab | azithromycin | NA | NA | NA | NA | NA | -0.18 | -0.71 | 0.22 | 2 | Intransitivity | -0.18 | -0.71 | 0.22 | 1 | Imprecision |
| chloroquine | azithromycin | NA | NA | NA | NA | NA | 0.20 | -0.26 | 0.60 | 3 | NA | 0.20 | -0.26 | 0.60 | 2 | Imprecision |
| colchicine | azithromycin | NA | NA | NA | NA | NA | -0.65 | -1.75 | 0.32 | 3 | NA | -0.65 | -1.75 | 0.32 | 1 | Severe Imprecision |
| convalescent plasma | azithromycin | NA | NA | NA | NA | NA | 0.04 | -0.09 | 0.17 | 2 | Intransitivity | 0.04 | -0.09 | 0.17 | 1 | Imprecision |
| dapagliflozin | azithromycin | NA | NA | NA | NA | NA | -0.22 | -0.66 | 0.21 | 3 | NA | -0.22 | -0.66 | 0.21 | 2 | Imprecision |
| dexamethasone | azithromycin | 0.06 | -0.08 | 0.19 | 3 | RoB; | -0.01 | -0.42 | 0.34 | 3 | NA | 0.05 | -0.09 | 0.18 | 2 | Imprecision |
| doxycycline | azithromycin | 0.04 | -0.44 | 0.63 | 3 | RoB; | NA | NA | NA | NA | NA | 0.04 | -0.44 | 0.63 | 2 | Imprecision |
| favipiravir | azithromycin | NA | NA | NA | NA | NA | 0.05 | -0.34 | 0.44 | 3 | NA | 0.05 | -0.34 | 0.44 | 2 | Imprecision |
| hydrocortisone | azithromycin | NA | NA | NA | NA | NA | -0.14 | -0.42 | 0.10 | 2 | Intransitivity | -0.14 | -0.42 | 0.10 | 1 | Imprecision |
| hydroxychloroquine | azithromycin | 0.28 | 0.14 | 0.42 | 3 | RoB; | 0.14 | -0.21 | 0.45 | 3 | NA | 0.23 | 0.10 | 0.36 | 3 | NA |
| hydroxychloroquine + azithromycin | azithromycin | NA | NA | NA | NA | NA | -0.53 | -1.88 | 0.58 | 3 | NA | -0.53 | -1.88 | 0.58 | 1 | Severe Imprecision |
| hydroxychloroquine + favipiravir | azithromycin | NA | NA | NA | NA | NA | 0.32 | -0.33 | 0.94 | 3 | NA | 0.32 | -0.33 | 0.94 | 2 | Imprecision |
| hydroxychloroquine + lopinavir/ritonavir | azithromycin | NA | NA | NA | NA | NA | 0.46 | -0.17 | 1.11 | 2 | Intransitivity | 0.46 | -0.17 | 1.11 | 1 | Imprecision |
| imatinib | azithromycin | NA | NA | NA | NA | NA | -0.52 | -1.06 | 0.00 | 2 | Intransitivity | -0.52 | -1.06 | 0.00 | 1 | Imprecision |
| inm005 | azithromycin | NA | NA | NA | NA | NA | -0.48 | -1.07 | 0.10 | 3 | NA | -0.48 | -1.07 | 0.10 | 2 | Imprecision |
| interferon beta | azithromycin | NA | NA | NA | NA | NA | 0.08 | -0.11 | 0.26 | 1 | Intransitivity | 0.08 | -0.11 | 0.26 | 1 | Imprecision |
| intravenous immunoglobulin | azithromycin | NA | NA | NA | NA | NA | -0.66 | -1.29 | -0.04 | 2 | Intransitivity | -0.66 | -1.29 | -0.04 | 2 | NA |
| ivermectin | azithromycin | NA | NA | NA | NA | NA | -0.41 | -0.92 | 0.09 | 3 | NA | -0.41 | -0.92 | 0.09 | 2 | Imprecision |
| lopinavir/ritonavir | azithromycin | 0.08 | -0.07 | 0.22 | 3 | RoB; | 0.07 | -0.25 | 0.39 | 3 | NA | 0.06 | -0.07 | 0.19 | 2 | Imprecision |
| mesenchymal stem cells | azithromycin | NA | NA | NA | NA | NA | -0.19 | -1.11 | 0.23 | 2 | Intransitivity | -0.19 | -1.11 | 0.23 | 1 | Imprecision |
| methylprednisolone | azithromycin | NA | NA | NA | NA | NA | -0.06 | -0.37 | 0.23 | 3 | NA | -0.06 | -0.37 | 0.23 | 2 | Imprecision |
| recombinant human gcsf | azithromycin | NA | NA | NA | NA | NA | -0.18 | -1.12 | 0.27 | 3 | NA | -0.18 | -1.12 | 0.27 | 2 | Imprecision |
| remdesivir | azithromycin | NA | NA | NA | NA | NA | 0.03 | -0.13 | 0.19 | 3 | NA | 0.03 | -0.13 | 0.19 | 2 | Imprecision |
| sarilumab | azithromycin | NA | NA | NA | NA | NA | -0.19 | -0.60 | 0.16 | 2 | Intransitivity | -0.19 | -0.60 | 0.16 | 1 | Imprecision |
| sofosbuvir + daclatasvir | azithromycin | NA | NA | NA | NA | NA | -0.06 | -0.73 | 0.33 | 3 | NA | -0.06 | -0.73 | 0.33 | 2 | Imprecision |
| sulodexide | azithromycin | NA | NA | NA | NA | NA | -0.09 | -0.86 | 0.40 | 3 | NA | -0.09 | -0.86 | 0.40 | 2 | Imprecision |
| therapeutic anticoagulation | azithromycin | NA | NA | NA | NA | NA | 0.02 | -0.18 | 0.22 | 3 | NA | 0.02 | -0.18 | 0.22 | 2 | Imprecision |
| tocilizumab | azithromycin | NA | NA | NA | NA | NA | -0.08 | -0.23 | 0.07 | 3 | NA | -0.08 | -0.23 | 0.07 | 2 | Imprecision |
| tofacitinib | azithromycin | NA | NA | NA | NA | NA | -0.20 | -0.84 | 0.24 | 3 | NA | -0.20 | -0.84 | 0.24 | 2 | Imprecision |
| vitamin c | azithromycin | NA | NA | NA | NA | NA | -0.19 | -1.05 | 0.68 | 3 | NA | -0.19 | -1.05 | 0.68 | 2 | Imprecision |
| vitamin d3 | azithromycin | NA | NA | NA | NA | NA | -0.26 | -1.01 | 0.46 | 3 | NA | -0.26 | -1.01 | 0.46 | 2 | Imprecision |
| baricitinib + remdesivir | bamlanivimab | NA | NA | NA | NA | NA | 0.00 | -0.80 | 0.79 | 3 | NA | 0.00 | -0.80 | 0.79 | 2 | Imprecision |
| budesonide | bamlanivimab | NA | NA | NA | NA | NA | 0.47 | -0.25 | 1.24 | 3 | NA | 0.47 | -0.25 | 1.24 | 2 | Imprecision |
| camostat mesilate | bamlanivimab | NA | NA | NA | NA | NA | 0.54 | -0.82 | 2.01 | 4 | NA | 0.54 | -0.82 | 2.01 | 2 | Severe Imprecision |
| canakinumab | bamlanivimab | NA | NA | NA | NA | NA | 0.24 | -0.57 | 0.92 | 3 | Intransitivity | 0.24 | -0.57 | 0.92 | 2 | Imprecision |
| chloroquine | bamlanivimab | NA | NA | NA | NA | NA | 0.62 | -0.13 | 1.30 | 3 | NA | 0.62 | -0.13 | 1.30 | 2 | Imprecision |
| colchicine | bamlanivimab | NA | NA | NA | NA | NA | -0.22 | -1.44 | 0.90 | 3 | NA | -0.22 | -1.44 | 0.90 | 2 | Imprecision |
| convalescent plasma | bamlanivimab | NA | NA | NA | NA | NA | 0.47 | -0.12 | 1.04 | 2 | Intransitivity | 0.47 | -0.12 | 1.04 | 1 | Imprecision |
| dapagliflozin | bamlanivimab | NA | NA | NA | NA | NA | 0.20 | -0.52 | 0.91 | 4 | NA | 0.20 | -0.52 | 0.91 | 3 | Imprecision |
| dexamethasone | bamlanivimab | NA | NA | NA | NA | NA | 0.48 | -0.12 | 1.05 | 3 | NA | 0.48 | -0.12 | 1.05 | 2 | Imprecision |
| doxycycline | bamlanivimab | NA | NA | NA | NA | NA | 0.48 | -0.28 | 1.27 | 3 | NA | 0.48 | -0.28 | 1.27 | 2 | Imprecision |
| favipiravir | bamlanivimab | NA | NA | NA | NA | NA | 0.48 | -0.22 | 1.14 | 3 | NA | 0.48 | -0.22 | 1.14 | 2 | Imprecision |
| hydrocortisone | bamlanivimab | NA | NA | NA | NA | NA | 0.29 | -0.38 | 0.89 | 3 | Intransitivity | 0.29 | -0.38 | 0.89 | 2 | Imprecision |
| hydroxychloroquine | bamlanivimab | NA | NA | NA | NA | NA | 0.66 | 0.06 | 1.24 | 3 | NA | 0.66 | 0.06 | 1.24 | 3 | NA |
| hydroxychloroquine + azithromycin | bamlanivimab | NA | NA | NA | NA | NA | -0.11 | -1.58 | 1.15 | 4 | NA | -0.11 | -1.58 | 1.15 | 2 | Severe Imprecision |
| hydroxychloroquine + favipiravir | bamlanivimab | NA | NA | NA | NA | NA | 0.75 | -0.15 | 1.58 | 3 | NA | 0.75 | -0.15 | 1.58 | 1 | Severe Imprecision |
| hydroxychloroquine + lopinavir/ritonavir | bamlanivimab | NA | NA | NA | NA | NA | 0.89 | 0.05 | 1.74 | 2 | Intransitivity | 0.89 | 0.05 | 1.74 | 2 | NA |
| imatinib | bamlanivimab | NA | NA | NA | NA | NA | -0.07 | -0.71 | 0.39 | 3 | Intransitivity | -0.07 | -0.71 | 0.39 | 2 | Imprecision |
| inm005 | bamlanivimab | NA | NA | NA | NA | NA | -0.04 | -0.67 | 0.47 | 4 | NA | -0.04 | -0.67 | 0.47 | 3 | Imprecision |
| interferon beta | bamlanivimab | NA | NA | NA | NA | NA | 0.50 | -0.10 | 1.09 | 1 | Intransitivity | 0.50 | -0.10 | 1.09 | 1 | Imprecision |
| intravenous immunoglobulin | bamlanivimab | NA | NA | NA | NA | NA | -0.23 | -1.08 | 0.61 | 2 | Intransitivity | -0.23 | -1.08 | 0.61 | 1 | Imprecision |
| ivermectin | bamlanivimab | NA | NA | NA | NA | NA | 0.01 | -0.75 | 0.76 | 4 | NA | 0.01 | -0.75 | 0.76 | 3 | Imprecision |
| lopinavir/ritonavir | bamlanivimab | NA | NA | NA | NA | NA | 0.49 | -0.11 | 1.06 | 3 | NA | 0.49 | -0.11 | 1.06 | 2 | Imprecision |
| mesenchymal stem cells | bamlanivimab | NA | NA | NA | NA | NA | 0.23 | -0.94 | 0.94 | 3 | Intransitivity | 0.23 | -0.94 | 0.94 | 2 | Imprecision |
| methylprednisolone | bamlanivimab | NA | NA | NA | NA | NA | 0.37 | -0.30 | 1.00 | 3 | NA | 0.37 | -0.30 | 1.00 | 2 | Imprecision |
| recombinant human gcsf | bamlanivimab | NA | NA | NA | NA | NA | 0.24 | -0.94 | 0.97 | 3 | NA | 0.24 | -0.94 | 0.97 | 2 | Imprecision |
| remdesivir | bamlanivimab | NA | NA | NA | NA | NA | 0.46 | -0.15 | 1.04 | 3 | NA | 0.46 | -0.15 | 1.04 | 2 | Imprecision |
| sarilumab | bamlanivimab | NA | NA | NA | NA | NA | 0.24 | -0.50 | 0.89 | 2 | Intransitivity | 0.24 | -0.50 | 0.89 | 1 | Imprecision |
| sofosbuvir + daclatasvir | bamlanivimab | NA | NA | NA | NA | NA | 0.36 | -0.57 | 1.04 | 3 | NA | 0.36 | -0.57 | 1.04 | 2 | Imprecision |
| sulodexide | bamlanivimab | NA | NA | NA | NA | NA | 0.34 | -0.68 | 1.08 | 3 | NA | 0.34 | -0.68 | 1.08 | 2 | Imprecision |
| therapeutic anticoagulation | bamlanivimab | NA | NA | NA | NA | NA | 0.45 | -0.16 | 1.04 | 3 | NA | 0.45 | -0.16 | 1.04 | 2 | Imprecision |
| tocilizumab | bamlanivimab | NA | NA | NA | NA | NA | 0.35 | -0.25 | 0.92 | 3 | NA | 0.35 | -0.25 | 0.92 | 2 | Imprecision |
| tofacitinib | bamlanivimab | NA | NA | NA | NA | NA | 0.23 | -0.67 | 0.93 | 4 | NA | 0.23 | -0.67 | 0.93 | 3 | Imprecision |
| vitamin c | bamlanivimab | NA | NA | NA | NA | NA | 0.24 | -0.79 | 1.27 | 3 | NA | 0.24 | -0.79 | 1.27 | 2 | Imprecision |
| vitamin d3 | bamlanivimab | NA | NA | NA | NA | NA | 0.16 | -0.78 | 1.07 | 3 | NA | 0.16 | -0.78 | 1.07 | 2 | Imprecision |
| budesonide | baricitinib + remdesivir | NA | NA | NA | NA | NA | 0.46 | -0.22 | 1.23 | 3 | NA | 0.46 | -0.22 | 1.23 | 2 | Imprecision |
| camostat mesilate | baricitinib + remdesivir | NA | NA | NA | NA | NA | 0.54 | -0.82 | 2.00 | 3 | NA | 0.54 | -0.82 | 2.00 | 1 | Severe Imprecision |
| canakinumab | baricitinib + remdesivir | NA | NA | NA | NA | NA | 0.23 | -0.49 | 0.92 | 3 | NA | 0.23 | -0.49 | 0.92 | 2 | Imprecision |
| chloroquine | baricitinib + remdesivir | NA | NA | NA | NA | NA | 0.61 | -0.06 | 1.29 | 3 | NA | 0.61 | -0.06 | 1.29 | 2 | Imprecision |
| colchicine | baricitinib + remdesivir | NA | NA | NA | NA | NA | -0.23 | -1.42 | 0.90 | 3 | NA | -0.23 | -1.42 | 0.90 | 2 | Imprecision |
| convalescent plasma | baricitinib + remdesivir | NA | NA | NA | NA | NA | 0.47 | -0.07 | 1.03 | 3 | NA | 0.47 | -0.07 | 1.03 | 2 | Imprecision |
| dapagliflozin | baricitinib + remdesivir | NA | NA | NA | NA | NA | 0.20 | -0.49 | 0.90 | 3 | NA | 0.20 | -0.49 | 0.90 | 2 | Imprecision |
| dexamethasone | baricitinib + remdesivir | NA | NA | NA | NA | NA | 0.47 | -0.07 | 1.04 | 3 | NA | 0.47 | -0.07 | 1.04 | 2 | Imprecision |
| doxycycline | baricitinib + remdesivir | NA | NA | NA | NA | NA | 0.47 | -0.25 | 1.26 | 3 | NA | 0.47 | -0.25 | 1.26 | 2 | Imprecision |
| favipiravir | baricitinib + remdesivir | NA | NA | NA | NA | NA | 0.47 | -0.16 | 1.13 | 3 | NA | 0.47 | -0.16 | 1.13 | 2 | Imprecision |
| hydrocortisone | baricitinib + remdesivir | NA | NA | NA | NA | NA | 0.28 | -0.31 | 0.88 | 3 | NA | 0.28 | -0.31 | 0.88 | 2 | Imprecision |
| hydroxychloroquine | baricitinib + remdesivir | NA | NA | NA | NA | NA | 0.65 | 0.12 | 1.22 | 3 | NA | 0.65 | 0.12 | 1.22 | 3 | NA |
| hydroxychloroquine + azithromycin | baricitinib + remdesivir | NA | NA | NA | NA | NA | -0.11 | -1.57 | 1.14 | 3 | NA | -0.11 | -1.57 | 1.14 | 1 | Severe Imprecision |
| hydroxychloroquine + favipiravir | baricitinib + remdesivir | NA | NA | NA | NA | NA | 0.74 | -0.09 | 1.56 | 3 | NA | 0.74 | -0.09 | 1.56 | 1 | Severe Imprecision |
| hydroxychloroquine + lopinavir/ritonavir | baricitinib + remdesivir | NA | NA | NA | NA | NA | 0.89 | 0.07 | 1.72 | 3 | NA | 0.89 | 0.07 | 1.72 | 3 | NA |
| imatinib | baricitinib + remdesivir | NA | NA | NA | NA | NA | -0.09 | -0.85 | 0.66 | 3 | NA | -0.09 | -0.85 | 0.66 | 2 | Imprecision |
| inm005 | baricitinib + remdesivir | NA | NA | NA | NA | NA | -0.06 | -0.85 | 0.74 | 3 | NA | -0.06 | -0.85 | 0.74 | 2 | Imprecision |
| interferon beta | baricitinib + remdesivir | NA | NA | NA | NA | NA | 0.50 | -0.05 | 1.07 | 3 | NA | 0.50 | -0.05 | 1.07 | 2 | Imprecision |
| intravenous immunoglobulin | baricitinib + remdesivir | NA | NA | NA | NA | NA | -0.23 | -1.06 | 0.59 | 3 | NA | -0.23 | -1.06 | 0.59 | 2 | Imprecision |
| ivermectin | baricitinib + remdesivir | NA | NA | NA | NA | NA | 0.01 | -0.72 | 0.75 | 3 | NA | 0.01 | -0.72 | 0.75 | 2 | Imprecision |
| lopinavir/ritonavir | baricitinib + remdesivir | NA | NA | NA | NA | NA | 0.48 | -0.05 | 1.04 | 3 | NA | 0.48 | -0.05 | 1.04 | 2 | Imprecision |
| mesenchymal stem cells | baricitinib + remdesivir | NA | NA | NA | NA | NA | 0.20 | -0.81 | 0.93 | 3 | NA | 0.20 | -0.81 | 0.93 | 2 | Imprecision |
| methylprednisolone | baricitinib + remdesivir | NA | NA | NA | NA | NA | 0.36 | -0.25 | 0.98 | 3 | NA | 0.36 | -0.25 | 0.98 | 2 | Imprecision |
| recombinant human gcsf | baricitinib + remdesivir | NA | NA | NA | NA | NA | 0.22 | -0.81 | 0.96 | 3 | NA | 0.22 | -0.81 | 0.96 | 2 | Imprecision |
| remdesivir | baricitinib + remdesivir | 0.45 | -0.07 | 0.99 | 4 | NA | NA | NA | NA | NA | NA | 0.45 | -0.07 | 0.99 | 3 | Imprecision |
| sarilumab | baricitinib + remdesivir | NA | NA | NA | NA | NA | 0.23 | -0.42 | 0.88 | 3 | NA | 0.23 | -0.42 | 0.88 | 2 | Imprecision |
| sofosbuvir + daclatasvir | baricitinib + remdesivir | NA | NA | NA | NA | NA | 0.34 | -0.46 | 1.02 | 3 | NA | 0.34 | -0.46 | 1.02 | 2 | Imprecision |
| sulodexide | baricitinib + remdesivir | NA | NA | NA | NA | NA | 0.32 | -0.57 | 1.07 | 3 | NA | 0.32 | -0.57 | 1.07 | 2 | Imprecision |
| therapeutic anticoagulation | baricitinib + remdesivir | NA | NA | NA | NA | NA | 0.45 | -0.11 | 1.03 | 3 | NA | 0.45 | -0.11 | 1.03 | 2 | Imprecision |
| tocilizumab | baricitinib + remdesivir | NA | NA | NA | NA | NA | 0.34 | -0.20 | 0.91 | 3 | NA | 0.34 | -0.20 | 0.91 | 2 | Imprecision |
| tofacitinib | baricitinib + remdesivir | NA | NA | NA | NA | NA | 0.21 | -0.58 | 0.92 | 3 | NA | 0.21 | -0.58 | 0.92 | 2 | Imprecision |
| vitamin c | baricitinib + remdesivir | NA | NA | NA | NA | NA | 0.23 | -0.77 | 1.26 | 3 | NA | 0.23 | -0.77 | 1.26 | 2 | Imprecision |
| vitamin d3 | baricitinib + remdesivir | NA | NA | NA | NA | NA | 0.16 | -0.75 | 1.07 | 3 | NA | 0.16 | -0.75 | 1.07 | 2 | Imprecision |
| camostat mesilate | budesonide | NA | NA | NA | NA | NA | 0.06 | -1.27 | 1.50 | 3 | NA | 0.06 | -1.27 | 1.50 | 2 | Imprecision |
| canakinumab | budesonide | NA | NA | NA | NA | NA | -0.21 | -1.04 | 0.33 | 2 | Intransitivity | -0.21 | -1.04 | 0.33 | 1 | Imprecision |
| chloroquine | budesonide | NA | NA | NA | NA | NA | 0.17 | -0.58 | 0.72 | 3 | NA | 0.17 | -0.58 | 0.72 | 2 | Imprecision |
| colchicine | budesonide | NA | NA | NA | NA | NA | -0.69 | -1.89 | 0.38 | 3 | NA | -0.69 | -1.89 | 0.38 | 1 | Severe Imprecision |
| convalescent plasma | budesonide | NA | NA | NA | NA | NA | 0.01 | -0.56 | 0.43 | 2 | Intransitivity | 0.01 | -0.56 | 0.43 | 1 | Imprecision |
| dapagliflozin | budesonide | NA | NA | NA | NA | NA | -0.26 | -0.95 | 0.33 | 3 | NA | -0.26 | -0.95 | 0.33 | 2 | Imprecision |
| dexamethasone | budesonide | NA | NA | NA | NA | NA | 0.02 | -0.55 | 0.44 | 3 | NA | 0.02 | -0.55 | 0.44 | 2 | Imprecision |
| doxycycline | budesonide | 0.01 | -0.70 | 0.67 | 3 | RoB; | NA | NA | NA | NA | NA | 0.01 | -0.70 | 0.67 | 2 | Imprecision |
| favipiravir | budesonide | NA | NA | NA | NA | NA | 0.02 | -0.66 | 0.56 | 3 | NA | 0.02 | -0.66 | 0.56 | 2 | Imprecision |
| hydrocortisone | budesonide | NA | NA | NA | NA | NA | -0.16 | -0.81 | 0.24 | 2 | Intransitivity | -0.16 | -0.81 | 0.24 | 1 | Imprecision |
| hydroxychloroquine | budesonide | NA | NA | NA | NA | NA | 0.20 | -0.37 | 0.62 | 3 | NA | 0.20 | -0.37 | 0.62 | 2 | Imprecision |
| hydroxychloroquine + azithromycin | budesonide | NA | NA | NA | NA | NA | -0.58 | -2.02 | 0.62 | 3 | NA | -0.58 | -2.02 | 0.62 | 1 | Severe Imprecision |
| hydroxychloroquine + favipiravir | budesonide | NA | NA | NA | NA | NA | 0.29 | -0.61 | 1.02 | 3 | NA | 0.29 | -0.61 | 1.02 | 2 | Imprecision |
| hydroxychloroquine + lopinavir/ritonavir | budesonide | NA | NA | NA | NA | NA | 0.42 | -0.36 | 1.17 | 2 | Intransitivity | 0.42 | -0.36 | 1.17 | 1 | Imprecision |
| imatinib | budesonide | NA | NA | NA | NA | NA | -0.55 | -1.34 | 0.10 | 2 | Intransitivity | -0.55 | -1.34 | 0.10 | 1 | Imprecision |
| inm005 | budesonide | NA | NA | NA | NA | NA | -0.52 | -1.33 | 0.18 | 3 | NA | -0.52 | -1.33 | 0.18 | 2 | Imprecision |
| interferon beta | budesonide | NA | NA | NA | NA | NA | 0.04 | -0.54 | 0.49 | 1 | Intransitivity | 0.04 | -0.54 | 0.49 | 1 | Imprecision |
| intravenous immunoglobulin | budesonide | NA | NA | NA | NA | NA | -0.70 | -1.51 | 0.05 | 2 | Intransitivity | -0.70 | -1.51 | 0.05 | 1 | Severe Imprecision |
| ivermectin | budesonide | NA | NA | NA | NA | NA | -0.45 | -1.18 | 0.20 | 3 | NA | -0.45 | -1.18 | 0.20 | 2 | Imprecision |
| lopinavir/ritonavir | budesonide | NA | NA | NA | NA | NA | 0.03 | -0.54 | 0.45 | 3 | NA | 0.03 | -0.54 | 0.45 | 2 | Imprecision |
| mesenchymal stem cells | budesonide | NA | NA | NA | NA | NA | -0.21 | -1.45 | 0.33 | 2 | Intransitivity | -0.21 | -1.45 | 0.33 | 1 | Imprecision |
| methylprednisolone | budesonide | NA | NA | NA | NA | NA | -0.08 | -0.72 | 0.34 | 3 | NA | -0.08 | -0.72 | 0.34 | 2 | Imprecision |
| recombinant human gcsf | budesonide | NA | NA | NA | NA | NA | -0.20 | -1.45 | 0.37 | 3 | NA | -0.20 | -1.45 | 0.37 | 2 | Imprecision |
| remdesivir | budesonide | NA | NA | NA | NA | NA | 0.00 | -0.58 | 0.43 | 3 | NA | 0.00 | -0.58 | 0.43 | 2 | Imprecision |
| sarilumab | budesonide | NA | NA | NA | NA | NA | -0.21 | -0.95 | 0.29 | 2 | Intransitivity | -0.21 | -0.95 | 0.29 | 1 | Imprecision |
| sofosbuvir + daclatasvir | budesonide | NA | NA | NA | NA | NA | -0.09 | -1.07 | 0.45 | 3 | NA | -0.09 | -1.07 | 0.45 | 2 | Imprecision |
| sulodexide | budesonide | NA | NA | NA | NA | NA | -0.11 | -1.17 | 0.49 | 3 | NA | -0.11 | -1.17 | 0.49 | 2 | Imprecision |
| therapeutic anticoagulation | budesonide | NA | NA | NA | NA | NA | -0.01 | -0.60 | 0.44 | 3 | NA | -0.01 | -0.60 | 0.44 | 2 | Imprecision |
| tocilizumab | budesonide | NA | NA | NA | NA | NA | -0.11 | -0.69 | 0.31 | 3 | NA | -0.11 | -0.69 | 0.31 | 2 | Imprecision |
| tofacitinib | budesonide | NA | NA | NA | NA | NA | -0.23 | -1.16 | 0.34 | 3 | NA | -0.23 | -1.16 | 0.34 | 2 | Imprecision |
| vitamin c | budesonide | NA | NA | NA | NA | NA | -0.24 | -1.23 | 0.72 | 3 | NA | -0.24 | -1.23 | 0.72 | 2 | Imprecision |
| vitamin d3 | budesonide | NA | NA | NA | NA | NA | -0.30 | -1.22 | 0.53 | 3 | NA | -0.30 | -1.22 | 0.53 | 2 | Imprecision |
| canakinumab | camostat mesilate | NA | NA | NA | NA | NA | -0.31 | -1.74 | 0.98 | 3 | Intransitivity | -0.31 | -1.74 | 0.98 | 1 | Severe Imprecision |
| chloroquine | camostat mesilate | NA | NA | NA | NA | NA | 0.08 | -1.34 | 1.37 | 3 | NA | 0.08 | -1.34 | 1.37 | 2 | Imprecision |
| colchicine | camostat mesilate | NA | NA | NA | NA | NA | -0.77 | -2.47 | 0.82 | 3 | NA | -0.77 | -2.47 | 0.82 | 1 | Severe Imprecision |
| convalescent plasma | camostat mesilate | NA | NA | NA | NA | NA | -0.06 | -1.43 | 1.16 | 2 | Intransitivity | -0.06 | -1.43 | 1.16 | 1 | Imprecision |
| dapagliflozin | camostat mesilate | NA | NA | NA | NA | NA | -0.33 | -1.76 | 0.97 | 4 | NA | -0.33 | -1.76 | 0.97 | 2 | Severe Imprecision |
| dexamethasone | camostat mesilate | NA | NA | NA | NA | NA | -0.06 | -1.43 | 1.17 | 3 | NA | -0.06 | -1.43 | 1.17 | 2 | Imprecision |
| doxycycline | camostat mesilate | NA | NA | NA | NA | NA | -0.06 | -1.50 | 1.29 | 3 | NA | -0.06 | -1.50 | 1.29 | 2 | Imprecision |
| favipiravir | camostat mesilate | NA | NA | NA | NA | NA | -0.06 | -1.47 | 1.23 | 3 | NA | -0.06 | -1.47 | 1.23 | 2 | Imprecision |
| hydrocortisone | camostat mesilate | NA | NA | NA | NA | NA | -0.25 | -1.64 | 1.00 | 3 | Intransitivity | -0.25 | -1.64 | 1.00 | 1 | Severe Imprecision |
| hydroxychloroquine | camostat mesilate | NA | NA | NA | NA | NA | 0.13 | -1.24 | 1.35 | 3 | NA | 0.13 | -1.24 | 1.35 | 2 | Imprecision |
| hydroxychloroquine + azithromycin | camostat mesilate | NA | NA | NA | NA | NA | -0.66 | -2.54 | 1.01 | 4 | NA | -0.66 | -2.54 | 1.01 | 2 | Severe Imprecision |
| hydroxychloroquine + favipiravir | camostat mesilate | NA | NA | NA | NA | NA | 0.20 | -1.29 | 1.58 | 3 | NA | 0.20 | -1.29 | 1.58 | 1 | Severe Imprecision |
| hydroxychloroquine + lopinavir/ritonavir | camostat mesilate | NA | NA | NA | NA | NA | 0.35 | -1.14 | 1.74 | 2 | Intransitivity | 0.35 | -1.14 | 1.74 | 1 | Severe Imprecision |
| imatinib | camostat mesilate | NA | NA | NA | NA | NA | -0.63 | -2.08 | 0.70 | 3 | Intransitivity | -0.63 | -2.08 | 0.70 | 1 | Severe Imprecision |
| inm005 | camostat mesilate | NA | NA | NA | NA | NA | -0.59 | -2.07 | 0.76 | 4 | NA | -0.59 | -2.07 | 0.76 | 2 | Severe Imprecision |
| interferon beta | camostat mesilate | NA | NA | NA | NA | NA | -0.03 | -1.40 | 1.20 | 1 | Intransitivity | -0.03 | -1.40 | 1.20 | 1 | Imprecision |
| intravenous immunoglobulin | camostat mesilate | NA | NA | NA | NA | NA | -0.77 | -2.26 | 0.61 | 2 | Intransitivity | -0.77 | -2.26 | 0.61 | 1 | Severe Imprecision |
| ivermectin | camostat mesilate | NA | NA | NA | NA | NA | -0.52 | -1.98 | 0.80 | 4 | NA | -0.52 | -1.98 | 0.80 | 2 | Severe Imprecision |
| lopinavir/ritonavir | camostat mesilate | NA | NA | NA | NA | NA | -0.05 | -1.41 | 1.18 | 3 | NA | -0.05 | -1.41 | 1.18 | 2 | Imprecision |
| mesenchymal stem cells | camostat mesilate | NA | NA | NA | NA | NA | -0.35 | -1.93 | 0.99 | 3 | Intransitivity | -0.35 | -1.93 | 0.99 | 1 | Severe Imprecision |
| methylprednisolone | camostat mesilate | NA | NA | NA | NA | NA | -0.17 | -1.56 | 1.09 | 3 | NA | -0.17 | -1.56 | 1.09 | 1 | Severe Imprecision |
| recombinant human gcsf | camostat mesilate | NA | NA | NA | NA | NA | -0.34 | -1.94 | 1.01 | 3 | NA | -0.34 | -1.94 | 1.01 | 1 | Severe Imprecision |
| remdesivir | camostat mesilate | NA | NA | NA | NA | NA | -0.08 | -1.45 | 1.15 | 3 | NA | -0.08 | -1.45 | 1.15 | 2 | Imprecision |
| sarilumab | camostat mesilate | NA | NA | NA | NA | NA | -0.30 | -1.72 | 0.97 | 2 | Intransitivity | -0.30 | -1.72 | 0.97 | 1 | Severe Imprecision |
| sofosbuvir + daclatasvir | camostat mesilate | NA | NA | NA | NA | NA | -0.20 | -1.67 | 1.11 | 3 | NA | -0.20 | -1.67 | 1.11 | 1 | Severe Imprecision |
| sulodexide | camostat mesilate | NA | NA | NA | NA | NA | -0.23 | -1.75 | 1.11 | 3 | NA | -0.23 | -1.75 | 1.11 | 1 | Severe Imprecision |
| therapeutic anticoagulation | camostat mesilate | NA | NA | NA | NA | NA | -0.08 | -1.46 | 1.15 | 3 | NA | -0.08 | -1.46 | 1.15 | 2 | Imprecision |
| tocilizumab | camostat mesilate | NA | NA | NA | NA | NA | -0.19 | -1.56 | 1.04 | 3 | NA | -0.19 | -1.56 | 1.04 | 1 | Severe Imprecision |
| tofacitinib | camostat mesilate | NA | NA | NA | NA | NA | -0.33 | -1.79 | 0.98 | 4 | NA | -0.33 | -1.79 | 0.98 | 2 | Severe Imprecision |
| vitamin c | camostat mesilate | NA | NA | NA | NA | NA | -0.30 | -1.89 | 1.21 | 3 | NA | -0.30 | -1.89 | 1.21 | 1 | Severe Imprecision |
| vitamin d3 | camostat mesilate | NA | NA | NA | NA | NA | -0.37 | -1.91 | 1.04 | 3 | NA | -0.37 | -1.91 | 1.04 | 1 | Severe Imprecision |
| chloroquine | canakinumab | NA | NA | NA | NA | NA | 0.38 | -0.19 | 1.03 | 2 | Intransitivity | 0.38 | -0.19 | 1.03 | 1 | Imprecision |
| colchicine | canakinumab | NA | NA | NA | NA | NA | -0.45 | -1.60 | 0.63 | 2 | Intransitivity | -0.45 | -1.60 | 0.63 | 1 | Severe Imprecision |
| convalescent plasma | canakinumab | NA | NA | NA | NA | NA | 0.23 | -0.17 | 0.75 | 3 | NA | 0.23 | -0.17 | 0.75 | 2 | Imprecision |
| dapagliflozin | canakinumab | NA | NA | NA | NA | NA | -0.03 | -0.62 | 0.62 | 4 | NA | -0.03 | -0.62 | 0.62 | 3 | Imprecision |
| dexamethasone | canakinumab | NA | NA | NA | NA | NA | 0.23 | -0.17 | 0.77 | 3 | NA | 0.23 | -0.17 | 0.77 | 2 | Imprecision |
| doxycycline | canakinumab | NA | NA | NA | NA | NA | 0.22 | -0.37 | 1.06 | 2 | Intransitivity | 0.22 | -0.37 | 1.06 | 1 | Imprecision |
| favipiravir | canakinumab | NA | NA | NA | NA | NA | 0.23 | -0.29 | 0.89 | 2 | Intransitivity | 0.23 | -0.29 | 0.89 | 1 | Imprecision |
| hydrocortisone | canakinumab | NA | NA | NA | NA | NA | 0.05 | -0.43 | 0.57 | 4 | NA | 0.05 | -0.43 | 0.57 | 3 | Imprecision |
| hydroxychloroquine | canakinumab | NA | NA | NA | NA | NA | 0.42 | 0.02 | 0.94 | 2 | Intransitivity | 0.42 | 0.02 | 0.94 | 1 | Imprecision |
| hydroxychloroquine + azithromycin | canakinumab | NA | NA | NA | NA | NA | -0.34 | -1.74 | 0.87 | 3 | Intransitivity | -0.34 | -1.74 | 0.87 | 1 | Severe Imprecision |
| hydroxychloroquine + favipiravir | canakinumab | NA | NA | NA | NA | NA | 0.52 | -0.23 | 1.27 | 2 | Intransitivity | 0.52 | -0.23 | 1.27 | 1 | Imprecision |
| hydroxychloroquine + lopinavir/ritonavir | canakinumab | NA | NA | NA | NA | NA | 0.65 | -0.08 | 1.49 | 3 | NA | 0.65 | -0.08 | 1.49 | 2 | Imprecision |
| imatinib | canakinumab | NA | NA | NA | NA | NA | -0.32 | -0.98 | 0.37 | 4 | NA | -0.32 | -0.98 | 0.37 | 3 | Imprecision |
| inm005 | canakinumab | NA | NA | NA | NA | NA | -0.29 | -0.98 | 0.47 | 4 | NA | -0.29 | -0.98 | 0.47 | 3 | Imprecision |
| interferon beta | canakinumab | NA | NA | NA | NA | NA | 0.26 | -0.16 | 0.80 | 2 | NA | 0.26 | -0.16 | 0.80 | 1 | Imprecision |
| intravenous immunoglobulin | canakinumab | NA | NA | NA | NA | NA | -0.46 | -1.21 | 0.32 | 3 | NA | -0.46 | -1.21 | 0.32 | 2 | Imprecision |
| ivermectin | canakinumab | NA | NA | NA | NA | NA | -0.22 | -0.86 | 0.48 | 3 | Intransitivity | -0.22 | -0.86 | 0.48 | 2 | Imprecision |
| lopinavir/ritonavir | canakinumab | NA | NA | NA | NA | NA | 0.24 | -0.15 | 0.77 | 3 | NA | 0.24 | -0.15 | 0.77 | 2 | Imprecision |
| mesenchymal stem cells | canakinumab | NA | NA | NA | NA | NA | 0.00 | -0.95 | 0.55 | 4 | NA | 0.00 | -0.95 | 0.55 | 3 | Imprecision |
| methylprednisolone | canakinumab | NA | NA | NA | NA | NA | 0.13 | -0.36 | 0.70 | 3 | NA | 0.13 | -0.36 | 0.70 | 2 | Imprecision |
| recombinant human gcsf | canakinumab | NA | NA | NA | NA | NA | 0.01 | -0.96 | 0.59 | 2 | Intransitivity | 0.01 | -0.96 | 0.59 | 1 | Imprecision |
| remdesivir | canakinumab | NA | NA | NA | NA | NA | 0.22 | -0.19 | 0.74 | 3 | NA | 0.22 | -0.19 | 0.74 | 2 | Imprecision |
| sarilumab | canakinumab | NA | NA | NA | NA | NA | 0.00 | -0.48 | 0.52 | 3 | NA | 0.00 | -0.48 | 0.52 | 2 | Imprecision |
| sofosbuvir + daclatasvir | canakinumab | NA | NA | NA | NA | NA | 0.12 | -0.60 | 0.72 | 3 | NA | 0.12 | -0.60 | 0.72 | 2 | Imprecision |
| sulodexide | canakinumab | NA | NA | NA | NA | NA | 0.09 | -0.72 | 0.77 | 2 | Intransitivity | 0.09 | -0.72 | 0.77 | 1 | Imprecision |
| therapeutic anticoagulation | canakinumab | NA | NA | NA | NA | NA | 0.21 | -0.21 | 0.75 | 3 | NA | 0.21 | -0.21 | 0.75 | 2 | Imprecision |
| tocilizumab | canakinumab | NA | NA | NA | NA | NA | 0.10 | -0.28 | 0.62 | 3 | NA | 0.10 | -0.28 | 0.62 | 2 | Imprecision |
| tofacitinib | canakinumab | NA | NA | NA | NA | NA | -0.01 | -0.63 | 0.53 | 4 | NA | -0.01 | -0.63 | 0.53 | 3 | Imprecision |
| vitamin c | canakinumab | NA | NA | NA | NA | NA | 0.00 | -0.94 | 1.04 | 3 | NA | 0.00 | -0.94 | 1.04 | 2 | Imprecision |
| vitamin d3 | canakinumab | NA | NA | NA | NA | NA | -0.07 | -0.91 | 0.79 | 3 | NA | -0.07 | -0.91 | 0.79 | 2 | Imprecision |
| colchicine | chloroquine | NA | NA | NA | NA | NA | -0.84 | -1.99 | 0.22 | 3 | NA | -0.84 | -1.99 | 0.22 | 1 | Severe Imprecision |
| convalescent plasma | chloroquine | NA | NA | NA | NA | NA | -0.15 | -0.55 | 0.29 | 2 | Intransitivity | -0.15 | -0.55 | 0.29 | 1 | Imprecision |
| dapagliflozin | chloroquine | NA | NA | NA | NA | NA | -0.42 | -1.00 | 0.18 | 3 | NA | -0.42 | -1.00 | 0.18 | 2 | Imprecision |
| dexamethasone | chloroquine | NA | NA | NA | NA | NA | -0.15 | -0.55 | 0.31 | 3 | NA | -0.15 | -0.55 | 0.31 | 2 | Imprecision |
| doxycycline | chloroquine | NA | NA | NA | NA | NA | -0.16 | -0.76 | 0.60 | 3 | NA | -0.16 | -0.76 | 0.60 | 2 | Imprecision |
| favipiravir | chloroquine | -0.20 | -0.92 | 0.39 | 3 | RoB; | -0.13 | -0.67 | 0.48 | 3 | NA | -0.15 | -0.67 | 0.43 | 2 | Imprecision |
| hydrocortisone | chloroquine | NA | NA | NA | NA | NA | -0.33 | -0.82 | 0.13 | 2 | Intransitivity | -0.33 | -0.82 | 0.13 | 1 | Imprecision |
| hydroxychloroquine | chloroquine | 0.00 | -0.38 | 0.38 | 3 | RoB; | -0.05 | -0.94 | 0.61 | 3 | NA | 0.04 | -0.35 | 0.47 | 2 | Imprecision |
| hydroxychloroquine + azithromycin | chloroquine | NA | NA | NA | NA | NA | -0.73 | -2.13 | 0.46 | 3 | NA | -0.73 | -2.13 | 0.46 | 1 | Severe Imprecision |
| hydroxychloroquine + favipiravir | chloroquine | NA | NA | NA | NA | NA | 0.13 | -0.63 | 0.86 | 3 | NA | 0.13 | -0.63 | 0.86 | 2 | Imprecision |
| hydroxychloroquine + lopinavir/ritonavir | chloroquine | NA | NA | NA | NA | NA | 0.27 | -0.46 | 1.06 | 3 | NA | 0.27 | -0.46 | 1.06 | 2 | Imprecision |
| imatinib | chloroquine | NA | NA | NA | NA | NA | -0.71 | -1.37 | -0.06 | 2 | Intransitivity | -0.71 | -1.37 | -0.06 | 2 | NA |
| inm005 | chloroquine | NA | NA | NA | NA | NA | -0.67 | -1.37 | 0.04 | 3 | NA | -0.67 | -1.37 | 0.04 | 2 | Imprecision |
| interferon beta | chloroquine | NA | NA | NA | NA | NA | -0.12 | -0.54 | 0.34 | 1 | Intransitivity | -0.12 | -0.54 | 0.34 | 1 | Imprecision |
| intravenous immunoglobulin | chloroquine | NA | NA | NA | NA | NA | -0.85 | -1.59 | -0.10 | 2 | Intransitivity | -0.85 | -1.59 | -0.10 | 2 | NA |
| ivermectin | chloroquine | -0.47 | -1.12 | 0.31 | 4 | NA | -0.78 | -1.59 | -0.08 | 3 | NA | -0.60 | -1.20 | -0.01 | 3 | Imprecision |
| lopinavir/ritonavir | chloroquine | NA | NA | NA | NA | NA | -0.13 | -0.53 | 0.31 | 3 | NA | -0.13 | -0.53 | 0.31 | 2 | Imprecision |
| mesenchymal stem cells | chloroquine | NA | NA | NA | NA | NA | -0.39 | -1.37 | 0.16 | 2 | Intransitivity | -0.39 | -1.37 | 0.16 | 1 | Imprecision |
| methylprednisolone | chloroquine | NA | NA | NA | NA | NA | -0.25 | -0.75 | 0.25 | 3 | NA | -0.25 | -0.75 | 0.25 | 2 | Imprecision |
| recombinant human gcsf | chloroquine | NA | NA | NA | NA | NA | -0.37 | -1.38 | 0.19 | 3 | NA | -0.37 | -1.38 | 0.19 | 2 | Imprecision |
| remdesivir | chloroquine | NA | NA | NA | NA | NA | -0.16 | -0.58 | 0.29 | 3 | NA | -0.16 | -0.58 | 0.29 | 2 | Imprecision |
| sarilumab | chloroquine | NA | NA | NA | NA | NA | -0.38 | -0.95 | 0.15 | 2 | Intransitivity | -0.38 | -0.95 | 0.15 | 1 | Imprecision |
| sofosbuvir + daclatasvir | chloroquine | NA | NA | NA | NA | NA | -0.26 | -1.01 | 0.29 | 3 | NA | -0.26 | -1.01 | 0.29 | 2 | Imprecision |
| sulodexide | chloroquine | NA | NA | NA | NA | NA | -0.29 | -1.13 | 0.35 | 3 | NA | -0.29 | -1.13 | 0.35 | 2 | Imprecision |
| therapeutic anticoagulation | chloroquine | NA | NA | NA | NA | NA | -0.17 | -0.60 | 0.30 | 3 | NA | -0.17 | -0.60 | 0.30 | 2 | Imprecision |
| tocilizumab | chloroquine | NA | NA | NA | NA | NA | -0.28 | -0.68 | 0.18 | 3 | NA | -0.28 | -0.68 | 0.18 | 2 | Imprecision |
| tofacitinib | chloroquine | NA | NA | NA | NA | NA | -0.40 | -1.13 | 0.20 | 3 | NA | -0.40 | -1.13 | 0.20 | 2 | Imprecision |
| vitamin c | chloroquine | NA | NA | NA | NA | NA | -0.39 | -1.32 | 0.60 | 3 | NA | -0.39 | -1.32 | 0.60 | 2 | Imprecision |
| vitamin d3 | chloroquine | NA | NA | NA | NA | NA | -0.45 | -1.29 | 0.37 | 3 | NA | -0.45 | -1.29 | 0.37 | 2 | Imprecision |
| convalescent plasma | colchicine | NA | NA | NA | NA | NA | 0.69 | -0.28 | 1.79 | 2 | Intransitivity | 0.69 | -0.28 | 1.79 | 1 | Severe Imprecision |
| dapagliflozin | colchicine | NA | NA | NA | NA | NA | 0.42 | -0.63 | 1.59 | 3 | NA | 0.42 | -0.63 | 1.59 | 1 | Severe Imprecision |
| dexamethasone | colchicine | NA | NA | NA | NA | NA | 0.70 | -0.28 | 1.79 | 3 | NA | 0.70 | -0.28 | 1.79 | 1 | Severe Imprecision |
| doxycycline | colchicine | NA | NA | NA | NA | NA | 0.70 | -0.39 | 1.90 | 3 | NA | 0.70 | -0.39 | 1.90 | 1 | Severe Imprecision |
| favipiravir | colchicine | NA | NA | NA | NA | NA | 0.70 | -0.34 | 1.85 | 3 | NA | 0.70 | -0.34 | 1.85 | 1 | Severe Imprecision |
| hydrocortisone | colchicine | NA | NA | NA | NA | NA | 0.50 | -0.50 | 1.62 | 2 | Intransitivity | 0.50 | -0.50 | 1.62 | 1 | Severe Imprecision |
| hydroxychloroquine | colchicine | NA | NA | NA | NA | NA | 0.88 | -0.09 | 1.98 | 3 | NA | 0.88 | -0.09 | 1.98 | 1 | Severe Imprecision |
| hydroxychloroquine + azithromycin | colchicine | NA | NA | NA | NA | NA | 0.10 | -1.54 | 1.69 | 3 | NA | 0.10 | -1.54 | 1.69 | 1 | Severe Imprecision |
| hydroxychloroquine + favipiravir | colchicine | NA | NA | NA | NA | NA | 0.97 | -0.20 | 2.21 | 3 | NA | 0.97 | -0.20 | 2.21 | 1 | Severe Imprecision |
| hydroxychloroquine + lopinavir/ritonavir | colchicine | NA | NA | NA | NA | NA | 1.12 | -0.04 | 2.36 | 2 | Intransitivity | 1.12 | -0.04 | 2.36 | 1 | Severe Imprecision |
| imatinib | colchicine | NA | NA | NA | NA | NA | 0.13 | -0.97 | 1.33 | 2 | Intransitivity | 0.13 | -0.97 | 1.33 | 1 | Imprecision |
| inm005 | colchicine | NA | NA | NA | NA | NA | 0.17 | -0.96 | 1.39 | 3 | NA | 0.17 | -0.96 | 1.39 | 2 | Imprecision |
| interferon beta | colchicine | NA | NA | NA | NA | NA | 0.72 | -0.26 | 1.83 | 1 | Intransitivity | 0.72 | -0.26 | 1.83 | 1 | Severe Imprecision |
| intravenous immunoglobulin | colchicine | NA | NA | NA | NA | NA | -0.01 | -1.16 | 1.23 | 2 | Intransitivity | -0.01 | -1.16 | 1.23 | 1 | Imprecision |
| ivermectin | colchicine | NA | NA | NA | NA | NA | 0.23 | -0.85 | 1.44 | 3 | NA | 0.23 | -0.85 | 1.44 | 2 | Imprecision |
| lopinavir/ritonavir | colchicine | NA | NA | NA | NA | NA | 0.71 | -0.26 | 1.81 | 3 | NA | 0.71 | -0.26 | 1.81 | 1 | Severe Imprecision |
| mesenchymal stem cells | colchicine | NA | NA | NA | NA | NA | 0.41 | -0.85 | 1.61 | 2 | Intransitivity | 0.41 | -0.85 | 1.61 | 1 | Severe Imprecision |
| methylprednisolone | colchicine | NA | NA | NA | NA | NA | 0.58 | -0.43 | 1.71 | 3 | NA | 0.58 | -0.43 | 1.71 | 1 | Severe Imprecision |
| recombinant human gcsf | colchicine | NA | NA | NA | NA | NA | 0.42 | -0.85 | 1.64 | 3 | NA | 0.42 | -0.85 | 1.64 | 1 | Severe Imprecision |
| remdesivir | colchicine | NA | NA | NA | NA | NA | 0.68 | -0.30 | 1.78 | 3 | NA | 0.68 | -0.30 | 1.78 | 1 | Severe Imprecision |
| sarilumab | colchicine | NA | NA | NA | NA | NA | 0.45 | -0.58 | 1.59 | 2 | Intransitivity | 0.45 | -0.58 | 1.59 | 1 | Severe Imprecision |
| sofosbuvir + daclatasvir | colchicine | NA | NA | NA | NA | NA | 0.56 | -0.57 | 1.73 | 3 | NA | 0.56 | -0.57 | 1.73 | 1 | Severe Imprecision |
| sulodexide | colchicine | NA | NA | NA | NA | NA | 0.53 | -0.64 | 1.73 | 3 | NA | 0.53 | -0.64 | 1.73 | 1 | Severe Imprecision |
| therapeutic anticoagulation | colchicine | NA | NA | NA | NA | NA | 0.67 | -0.31 | 1.77 | 3 | NA | 0.67 | -0.31 | 1.77 | 1 | Severe Imprecision |
| tocilizumab | colchicine | NA | NA | NA | NA | NA | 0.57 | -0.41 | 1.67 | 3 | NA | 0.57 | -0.41 | 1.67 | 1 | Severe Imprecision |
| tofacitinib | colchicine | NA | NA | NA | NA | NA | 0.43 | -0.68 | 1.60 | 3 | NA | 0.43 | -0.68 | 1.60 | 1 | Severe Imprecision |
| vitamin c | colchicine | NA | NA | NA | NA | NA | 0.46 | -0.86 | 1.85 | 3 | NA | 0.46 | -0.86 | 1.85 | 1 | Severe Imprecision |
| vitamin d3 | colchicine | NA | NA | NA | NA | NA | 0.39 | -0.86 | 1.69 | 3 | NA | 0.39 | -0.86 | 1.69 | 1 | Severe Imprecision |
| dapagliflozin | convalescent plasma | NA | NA | NA | NA | NA | -0.27 | -0.70 | 0.16 | 3 | NA | -0.27 | -0.70 | 0.16 | 2 | Imprecision |
| dexamethasone | convalescent plasma | NA | NA | NA | NA | NA | 0.00 | -0.13 | 0.14 | 3 | NA | 0.00 | -0.13 | 0.14 | 2 | Imprecision |
| doxycycline | convalescent plasma | NA | NA | NA | NA | NA | 0.00 | -0.49 | 0.60 | 2 | Intransitivity | 0.00 | -0.49 | 0.60 | 1 | Imprecision |
| favipiravir | convalescent plasma | NA | NA | NA | NA | NA | 0.00 | -0.38 | 0.39 | 2 | Intransitivity | 0.00 | -0.38 | 0.39 | 1 | Imprecision |
| hydrocortisone | convalescent plasma | NA | NA | NA | NA | NA | -0.18 | -0.45 | 0.06 | 3 | NA | -0.18 | -0.45 | 0.06 | 2 | Imprecision |
| hydroxychloroquine | convalescent plasma | NA | NA | NA | NA | NA | 0.19 | 0.06 | 0.32 | 2 | Intransitivity | 0.19 | 0.06 | 0.32 | 2 | NA |
| hydroxychloroquine + azithromycin | convalescent plasma | NA | NA | NA | NA | NA | -0.58 | -1.92 | 0.54 | 2 | Intransitivity | -0.58 | -1.92 | 0.54 | 1 | Severe Imprecision |
| hydroxychloroquine + favipiravir | convalescent plasma | NA | NA | NA | NA | NA | 0.28 | -0.37 | 0.89 | 2 | Intransitivity | 0.28 | -0.37 | 0.89 | 1 | Imprecision |
| hydroxychloroquine + lopinavir/ritonavir | convalescent plasma | NA | NA | NA | NA | NA | 0.42 | -0.21 | 1.06 | 3 | NA | 0.42 | -0.21 | 1.06 | 2 | Imprecision |
| imatinib | convalescent plasma | NA | NA | NA | NA | NA | -0.56 | -1.09 | -0.05 | 3 | NA | -0.56 | -1.09 | -0.05 | 3 | NA |
| inm005 | convalescent plasma | NA | NA | NA | NA | NA | -0.52 | -1.11 | 0.05 | 3 | NA | -0.52 | -1.11 | 0.05 | 2 | Imprecision |
| interferon beta | convalescent plasma | NA | NA | NA | NA | NA | 0.03 | -0.14 | 0.20 | 2 | NA | 0.03 | -0.14 | 0.20 | 1 | Imprecision |
| intravenous immunoglobulin | convalescent plasma | NA | NA | NA | NA | NA | -0.70 | -1.33 | -0.08 | 3 | NA | -0.70 | -1.33 | -0.08 | 3 | NA |
| ivermectin | convalescent plasma | NA | NA | NA | NA | NA | -0.46 | -0.96 | 0.04 | 2 | Intransitivity | -0.46 | -0.96 | 0.04 | 1 | Imprecision |
| lopinavir/ritonavir | convalescent plasma | NA | NA | NA | NA | NA | 0.02 | -0.11 | 0.15 | 3 | NA | 0.02 | -0.11 | 0.15 | 2 | Imprecision |
| mesenchymal stem cells | convalescent plasma | NA | NA | NA | NA | NA | -0.23 | -1.15 | 0.17 | 3 | NA | -0.23 | -1.15 | 0.17 | 2 | Imprecision |
| methylprednisolone | convalescent plasma | NA | NA | NA | NA | NA | -0.10 | -0.41 | 0.18 | 3 | NA | -0.10 | -0.41 | 0.18 | 2 | Imprecision |
| recombinant human gcsf | convalescent plasma | NA | NA | NA | NA | NA | -0.22 | -1.16 | 0.23 | 2 | Intransitivity | -0.22 | -1.16 | 0.23 | 1 | Imprecision |
| remdesivir | convalescent plasma | NA | NA | NA | NA | NA | -0.01 | -0.16 | 0.14 | 3 | NA | -0.01 | -0.16 | 0.14 | 2 | Imprecision |
| sarilumab | convalescent plasma | NA | NA | NA | NA | NA | -0.23 | -0.63 | 0.11 | 3 | NA | -0.23 | -0.63 | 0.11 | 2 | Imprecision |
| sofosbuvir + daclatasvir | convalescent plasma | NA | NA | NA | NA | NA | -0.10 | -0.77 | 0.28 | 3 | NA | -0.10 | -0.77 | 0.28 | 2 | Imprecision |
| sulodexide | convalescent plasma | NA | NA | NA | NA | NA | -0.13 | -0.90 | 0.35 | 2 | Intransitivity | -0.13 | -0.90 | 0.35 | 1 | Imprecision |
| therapeutic anticoagulation | convalescent plasma | NA | NA | NA | NA | NA | -0.02 | -0.21 | 0.17 | 3 | NA | -0.02 | -0.21 | 0.17 | 2 | Imprecision |
| tocilizumab | convalescent plasma | NA | NA | NA | NA | NA | -0.12 | -0.26 | 0.01 | 3 | NA | -0.12 | -0.26 | 0.01 | 2 | Imprecision |
| tofacitinib | convalescent plasma | NA | NA | NA | NA | NA | -0.24 | -0.87 | 0.19 | 3 | NA | -0.24 | -0.87 | 0.19 | 2 | Imprecision |
| vitamin c | convalescent plasma | NA | NA | NA | NA | NA | -0.24 | -1.09 | 0.64 | 3 | NA | -0.24 | -1.09 | 0.64 | 2 | Imprecision |
| vitamin d3 | convalescent plasma | NA | NA | NA | NA | NA | -0.31 | -1.05 | 0.41 | 3 | NA | -0.31 | -1.05 | 0.41 | 2 | Imprecision |
| dexamethasone | dapagliflozin | NA | NA | NA | NA | NA | 0.27 | -0.16 | 0.71 | 3 | NA | 0.27 | -0.16 | 0.71 | 2 | Imprecision |
| doxycycline | dapagliflozin | NA | NA | NA | NA | NA | 0.27 | -0.38 | 0.98 | 3 | NA | 0.27 | -0.38 | 0.98 | 2 | Imprecision |
| favipiravir | dapagliflozin | NA | NA | NA | NA | NA | 0.27 | -0.29 | 0.83 | 3 | NA | 0.27 | -0.29 | 0.83 | 2 | Imprecision |
| hydrocortisone | dapagliflozin | NA | NA | NA | NA | NA | 0.08 | -0.41 | 0.57 | 4 | NA | 0.08 | -0.41 | 0.57 | 3 | Imprecision |
| hydroxychloroquine | dapagliflozin | NA | NA | NA | NA | NA | 0.46 | 0.02 | 0.90 | 3 | NA | 0.46 | 0.02 | 0.90 | 3 | NA |
| hydroxychloroquine + azithromycin | dapagliflozin | NA | NA | NA | NA | NA | -0.31 | -1.71 | 0.88 | 4 | NA | -0.31 | -1.71 | 0.88 | 2 | Severe Imprecision |
| hydroxychloroquine + favipiravir | dapagliflozin | NA | NA | NA | NA | NA | 0.54 | -0.22 | 1.29 | 3 | NA | 0.54 | -0.22 | 1.29 | 2 | Imprecision |
| hydroxychloroquine + lopinavir/ritonavir | dapagliflozin | NA | NA | NA | NA | NA | 0.69 | -0.07 | 1.46 | 3 | NA | 0.69 | -0.07 | 1.46 | 2 | Imprecision |
| imatinib | dapagliflozin | NA | NA | NA | NA | NA | -0.29 | -0.97 | 0.37 | 4 | NA | -0.29 | -0.97 | 0.37 | 3 | Imprecision |
| inm005 | dapagliflozin | NA | NA | NA | NA | NA | -0.25 | -0.97 | 0.45 | 4 | NA | -0.25 | -0.97 | 0.45 | 3 | Imprecision |
| interferon beta | dapagliflozin | NA | NA | NA | NA | NA | 0.30 | -0.15 | 0.75 | 2 | NA | 0.30 | -0.15 | 0.75 | 1 | Imprecision |
| intravenous immunoglobulin | dapagliflozin | NA | NA | NA | NA | NA | -0.43 | -1.19 | 0.32 | 3 | NA | -0.43 | -1.19 | 0.32 | 2 | Imprecision |
| ivermectin | dapagliflozin | NA | NA | NA | NA | NA | -0.19 | -0.85 | 0.47 | 4 | NA | -0.19 | -0.85 | 0.47 | 3 | Imprecision |
| lopinavir/ritonavir | dapagliflozin | NA | NA | NA | NA | NA | 0.28 | -0.15 | 0.72 | 3 | NA | 0.28 | -0.15 | 0.72 | 2 | Imprecision |
| mesenchymal stem cells | dapagliflozin | NA | NA | NA | NA | NA | 0.01 | -0.96 | 0.63 | 4 | NA | 0.01 | -0.96 | 0.63 | 3 | Imprecision |
| methylprednisolone | dapagliflozin | NA | NA | NA | NA | NA | 0.16 | -0.35 | 0.67 | 3 | NA | 0.16 | -0.35 | 0.67 | 2 | Imprecision |
| recombinant human gcsf | dapagliflozin | NA | NA | NA | NA | NA | 0.02 | -0.97 | 0.67 | 3 | NA | 0.02 | -0.97 | 0.67 | 2 | Imprecision |
| remdesivir | dapagliflozin | NA | NA | NA | NA | NA | 0.25 | -0.19 | 0.70 | 3 | NA | 0.25 | -0.19 | 0.70 | 2 | Imprecision |
| sarilumab | dapagliflozin | NA | NA | NA | NA | NA | 0.03 | -0.54 | 0.57 | 3 | NA | 0.03 | -0.54 | 0.57 | 2 | Imprecision |
| sofosbuvir + daclatasvir | dapagliflozin | NA | NA | NA | NA | NA | 0.15 | -0.61 | 0.73 | 3 | NA | 0.15 | -0.61 | 0.73 | 2 | Imprecision |
| sulodexide | dapagliflozin | NA | NA | NA | NA | NA | 0.12 | -0.72 | 0.78 | 3 | NA | 0.12 | -0.72 | 0.78 | 2 | Imprecision |
| therapeutic anticoagulation | dapagliflozin | NA | NA | NA | NA | NA | 0.25 | -0.21 | 0.70 | 3 | NA | 0.25 | -0.21 | 0.70 | 2 | Imprecision |
| tocilizumab | dapagliflozin | NA | NA | NA | NA | NA | 0.14 | -0.29 | 0.58 | 3 | NA | 0.14 | -0.29 | 0.58 | 2 | Imprecision |
| tofacitinib | dapagliflozin | NA | NA | NA | NA | NA | 0.01 | -0.72 | 0.62 | 4 | NA | 0.01 | -0.72 | 0.62 | 3 | Imprecision |
| vitamin c | dapagliflozin | NA | NA | NA | NA | NA | 0.03 | -0.92 | 1.01 | 3 | NA | 0.03 | -0.92 | 1.01 | 2 | Imprecision |
| vitamin d3 | dapagliflozin | NA | NA | NA | NA | NA | -0.04 | -0.90 | 0.80 | 3 | NA | -0.04 | -0.90 | 0.80 | 2 | Imprecision |
| doxycycline | dexamethasone | NA | NA | NA | NA | NA | -0.01 | -0.50 | 0.58 | 3 | NA | -0.01 | -0.50 | 0.58 | 2 | Imprecision |
| favipiravir | dexamethasone | NA | NA | NA | NA | NA | 0.00 | -0.39 | 0.39 | 3 | NA | 0.00 | -0.39 | 0.39 | 2 | Imprecision |
| hydrocortisone | dexamethasone | NA | NA | NA | NA | NA | -0.19 | -0.47 | 0.05 | 3 | NA | -0.19 | -0.47 | 0.05 | 2 | Imprecision |
| hydroxychloroquine | dexamethasone | 0.22 | 0.07 | 0.36 | 3 | RoB; | 0.23 | -0.07 | 0.51 | 3 | NA | 0.18 | 0.05 | 0.32 | 3 | NA |
| hydroxychloroquine + azithromycin | dexamethasone | NA | NA | NA | NA | NA | -0.58 | -1.93 | 0.54 | 3 | NA | -0.58 | -1.93 | 0.54 | 1 | Severe Imprecision |
| hydroxychloroquine + favipiravir | dexamethasone | NA | NA | NA | NA | NA | 0.27 | -0.38 | 0.90 | 3 | NA | 0.27 | -0.38 | 0.90 | 2 | Imprecision |
| hydroxychloroquine + lopinavir/ritonavir | dexamethasone | NA | NA | NA | NA | NA | 0.42 | -0.21 | 1.06 | 3 | NA | 0.42 | -0.21 | 1.06 | 2 | Imprecision |
| imatinib | dexamethasone | NA | NA | NA | NA | NA | -0.56 | -1.11 | -0.05 | 3 | NA | -0.56 | -1.11 | -0.05 | 3 | NA |
| inm005 | dexamethasone | NA | NA | NA | NA | NA | -0.53 | -1.12 | 0.05 | 3 | NA | -0.53 | -1.12 | 0.05 | 2 | Imprecision |
| interferon beta | dexamethasone | NA | NA | NA | NA | NA | 0.03 | -0.16 | 0.21 | 2 | NA | 0.03 | -0.16 | 0.21 | 1 | Imprecision |
| intravenous immunoglobulin | dexamethasone | NA | NA | NA | NA | NA | -0.70 | -1.34 | -0.08 | 3 | NA | -0.70 | -1.34 | -0.08 | 3 | NA |
| ivermectin | dexamethasone | NA | NA | NA | NA | NA | -0.46 | -0.97 | 0.04 | 3 | NA | -0.46 | -0.97 | 0.04 | 2 | Imprecision |
| lopinavir/ritonavir | dexamethasone | 0.03 | -0.12 | 0.17 | 3 | RoB; | 0.11 | -0.17 | 0.41 | 3 | NA | 0.01 | -0.12 | 0.15 | 2 | Imprecision |
| mesenchymal stem cells | dexamethasone | NA | NA | NA | NA | NA | -0.24 | -1.17 | 0.19 | 3 | NA | -0.24 | -1.17 | 0.19 | 2 | Imprecision |
| methylprednisolone | dexamethasone | -0.11 | -0.77 | 0.21 | 4 | NA | -0.03 | -0.34 | 0.28 | 3 | NA | -0.10 | -0.41 | 0.17 | 3 | Imprecision |
| recombinant human gcsf | dexamethasone | NA | NA | NA | NA | NA | -0.22 | -1.18 | 0.23 | 3 | NA | -0.22 | -1.18 | 0.23 | 2 | Imprecision |
| remdesivir | dexamethasone | NA | NA | NA | NA | NA | -0.02 | -0.18 | 0.14 | 3 | NA | -0.02 | -0.18 | 0.14 | 2 | Imprecision |
| sarilumab | dexamethasone | NA | NA | NA | NA | NA | -0.23 | -0.65 | 0.11 | 3 | NA | -0.23 | -0.65 | 0.11 | 2 | Imprecision |
| sofosbuvir + daclatasvir | dexamethasone | NA | NA | NA | NA | NA | -0.11 | -0.78 | 0.28 | 3 | NA | -0.11 | -0.78 | 0.28 | 2 | Imprecision |
| sulodexide | dexamethasone | NA | NA | NA | NA | NA | -0.13 | -0.91 | 0.35 | 3 | NA | -0.13 | -0.91 | 0.35 | 2 | Imprecision |
| therapeutic anticoagulation | dexamethasone | NA | NA | NA | NA | NA | -0.02 | -0.23 | 0.18 | 3 | NA | -0.02 | -0.23 | 0.18 | 2 | Imprecision |
| tocilizumab | dexamethasone | 0.12 | -0.30 | 0.78 | 3 | RoB; | -0.15 | -0.31 | 0.00 | 3 | NA | -0.13 | -0.28 | 0.02 | 2 | Imprecision |
| tofacitinib | dexamethasone | NA | NA | NA | NA | NA | -0.24 | -0.89 | 0.19 | 3 | NA | -0.24 | -0.89 | 0.19 | 2 | Imprecision |
| vitamin c | dexamethasone | NA | NA | NA | NA | NA | -0.24 | -1.10 | 0.64 | 3 | NA | -0.24 | -1.10 | 0.64 | 2 | Imprecision |
| vitamin d3 | dexamethasone | NA | NA | NA | NA | NA | -0.31 | -1.06 | 0.41 | 3 | NA | -0.31 | -1.06 | 0.41 | 2 | Imprecision |
| favipiravir | doxycycline | NA | NA | NA | NA | NA | 0.01 | -0.69 | 0.62 | 3 | NA | 0.01 | -0.69 | 0.62 | 2 | Imprecision |
| hydrocortisone | doxycycline | NA | NA | NA | NA | NA | -0.17 | -0.86 | 0.33 | 2 | Intransitivity | -0.17 | -0.86 | 0.33 | 1 | Imprecision |
| hydroxychloroquine | doxycycline | NA | NA | NA | NA | NA | 0.19 | -0.41 | 0.68 | 3 | NA | 0.19 | -0.41 | 0.68 | 2 | Imprecision |
| hydroxychloroquine + azithromycin | doxycycline | NA | NA | NA | NA | NA | -0.58 | -2.04 | 0.65 | 3 | NA | -0.58 | -2.04 | 0.65 | 1 | Severe Imprecision |
| hydroxychloroquine + favipiravir | doxycycline | NA | NA | NA | NA | NA | 0.28 | -0.62 | 1.04 | 3 | NA | 0.28 | -0.62 | 1.04 | 2 | Imprecision |
| hydroxychloroquine + lopinavir/ritonavir | doxycycline | NA | NA | NA | NA | NA | 0.41 | -0.40 | 1.22 | 2 | Intransitivity | 0.41 | -0.40 | 1.22 | 1 | Imprecision |
| imatinib | doxycycline | NA | NA | NA | NA | NA | -0.56 | -1.36 | 0.13 | 2 | Intransitivity | -0.56 | -1.36 | 0.13 | 1 | Imprecision |
| inm005 | doxycycline | NA | NA | NA | NA | NA | -0.52 | -1.36 | 0.21 | 3 | NA | -0.52 | -1.36 | 0.21 | 2 | Imprecision |
| interferon beta | doxycycline | NA | NA | NA | NA | NA | 0.03 | -0.57 | 0.54 | 1 | Intransitivity | 0.03 | -0.57 | 0.54 | 1 | Imprecision |
| intravenous immunoglobulin | doxycycline | NA | NA | NA | NA | NA | -0.71 | -1.54 | 0.07 | 2 | Intransitivity | -0.71 | -1.54 | 0.07 | 1 | Severe Imprecision |
| ivermectin | doxycycline | NA | NA | NA | NA | NA | -0.46 | -1.20 | 0.22 | 3 | NA | -0.46 | -1.20 | 0.22 | 2 | Imprecision |
| lopinavir/ritonavir | doxycycline | NA | NA | NA | NA | NA | 0.02 | -0.57 | 0.51 | 3 | NA | 0.02 | -0.57 | 0.51 | 2 | Imprecision |
| mesenchymal stem cells | doxycycline | NA | NA | NA | NA | NA | -0.22 | -1.44 | 0.36 | 2 | Intransitivity | -0.22 | -1.44 | 0.36 | 1 | Imprecision |
| methylprednisolone | doxycycline | NA | NA | NA | NA | NA | -0.10 | -0.77 | 0.44 | 3 | NA | -0.10 | -0.77 | 0.44 | 2 | Imprecision |
| recombinant human gcsf | doxycycline | NA | NA | NA | NA | NA | -0.21 | -1.44 | 0.38 | 3 | NA | -0.21 | -1.44 | 0.38 | 2 | Imprecision |
| remdesivir | doxycycline | NA | NA | NA | NA | NA | -0.01 | -0.62 | 0.49 | 3 | NA | -0.01 | -0.62 | 0.49 | 2 | Imprecision |
| sarilumab | doxycycline | NA | NA | NA | NA | NA | -0.22 | -0.97 | 0.34 | 2 | Intransitivity | -0.22 | -0.97 | 0.34 | 1 | Imprecision |
| sofosbuvir + daclatasvir | doxycycline | NA | NA | NA | NA | NA | -0.10 | -1.07 | 0.48 | 3 | NA | -0.10 | -1.07 | 0.48 | 2 | Imprecision |
| sulodexide | doxycycline | NA | NA | NA | NA | NA | -0.13 | -1.16 | 0.52 | 3 | NA | -0.13 | -1.16 | 0.52 | 2 | Imprecision |
| therapeutic anticoagulation | doxycycline | NA | NA | NA | NA | NA | -0.02 | -0.63 | 0.49 | 3 | NA | -0.02 | -0.63 | 0.49 | 2 | Imprecision |
| tocilizumab | doxycycline | NA | NA | NA | NA | NA | -0.12 | -0.72 | 0.37 | 3 | NA | -0.12 | -0.72 | 0.37 | 2 | Imprecision |
| tofacitinib | doxycycline | NA | NA | NA | NA | NA | -0.24 | -1.16 | 0.37 | 3 | NA | -0.24 | -1.16 | 0.37 | 2 | Imprecision |
| vitamin c | doxycycline | NA | NA | NA | NA | NA | -0.24 | -1.25 | 0.76 | 3 | NA | -0.24 | -1.25 | 0.76 | 2 | Imprecision |
| vitamin d3 | doxycycline | NA | NA | NA | NA | NA | -0.31 | -1.25 | 0.55 | 3 | NA | -0.31 | -1.25 | 0.55 | 2 | Imprecision |
| hydrocortisone | favipiravir | NA | NA | NA | NA | NA | -0.18 | -0.66 | 0.22 | 2 | Intransitivity | -0.18 | -0.66 | 0.22 | 1 | Imprecision |
| hydroxychloroquine | favipiravir | 0.22 | -0.41 | 1.01 | 3 | RoB; | 0.17 | -0.23 | 0.55 | 3 | NA | 0.19 | -0.20 | 0.57 | 2 | Imprecision |
| hydroxychloroquine + azithromycin | favipiravir | NA | NA | NA | NA | NA | -0.58 | -1.97 | 0.59 | 3 | NA | -0.58 | -1.97 | 0.59 | 1 | Severe Imprecision |
| hydroxychloroquine + favipiravir | favipiravir | NA | NA | NA | NA | NA | 0.27 | -0.48 | 0.98 | 3 | NA | 0.27 | -0.48 | 0.98 | 2 | Imprecision |
| hydroxychloroquine + lopinavir/ritonavir | favipiravir | NA | NA | NA | NA | NA | 0.41 | -0.30 | 1.15 | 2 | Intransitivity | 0.41 | -0.30 | 1.15 | 1 | Imprecision |
| imatinib | favipiravir | NA | NA | NA | NA | NA | -0.56 | -1.22 | 0.05 | 2 | Intransitivity | -0.56 | -1.22 | 0.05 | 1 | Imprecision |
| inm005 | favipiravir | NA | NA | NA | NA | NA | -0.53 | -1.22 | 0.14 | 3 | NA | -0.53 | -1.22 | 0.14 | 2 | Imprecision |
| interferon beta | favipiravir | NA | NA | NA | NA | NA | 0.03 | -0.37 | 0.42 | 1 | Intransitivity | 0.03 | -0.37 | 0.42 | 1 | Imprecision |
| intravenous immunoglobulin | favipiravir | NA | NA | NA | NA | NA | -0.70 | -1.43 | 0.00 | 2 | Intransitivity | -0.70 | -1.43 | 0.00 | 1 | Imprecision |
| ivermectin | favipiravir | NA | NA | NA | NA | NA | -0.46 | -1.08 | 0.14 | 3 | NA | -0.46 | -1.08 | 0.14 | 2 | Imprecision |
| lopinavir/ritonavir | favipiravir | -0.05 | -0.45 | 0.27 | 3 | RoB; | 0.12 | -0.30 | 0.91 | 3 | NA | 0.01 | -0.36 | 0.38 | 2 | Imprecision |
| mesenchymal stem cells | favipiravir | NA | NA | NA | NA | NA | -0.24 | -1.26 | 0.28 | 2 | Intransitivity | -0.24 | -1.26 | 0.28 | 1 | Imprecision |
| methylprednisolone | favipiravir | NA | NA | NA | NA | NA | -0.10 | -0.60 | 0.35 | 3 | NA | -0.10 | -0.60 | 0.35 | 2 | Imprecision |
| recombinant human gcsf | favipiravir | NA | NA | NA | NA | NA | -0.22 | -1.27 | 0.31 | 3 | NA | -0.22 | -1.27 | 0.31 | 2 | Imprecision |
| remdesivir | favipiravir | NA | NA | NA | NA | NA | -0.02 | -0.41 | 0.36 | 3 | NA | -0.02 | -0.41 | 0.36 | 2 | Imprecision |
| sarilumab | favipiravir | NA | NA | NA | NA | NA | -0.23 | -0.80 | 0.25 | 2 | Intransitivity | -0.23 | -0.80 | 0.25 | 1 | Imprecision |
| sofosbuvir + daclatasvir | favipiravir | NA | NA | NA | NA | NA | -0.10 | -0.84 | 0.33 | 3 | NA | -0.10 | -0.84 | 0.33 | 2 | Imprecision |
| sulodexide | favipiravir | NA | NA | NA | NA | NA | -0.13 | -1.01 | 0.45 | 3 | NA | -0.13 | -1.01 | 0.45 | 2 | Imprecision |
| therapeutic anticoagulation | favipiravir | NA | NA | NA | NA | NA | -0.02 | -0.44 | 0.38 | 3 | NA | -0.02 | -0.44 | 0.38 | 2 | Imprecision |
| tocilizumab | favipiravir | NA | NA | NA | NA | NA | -0.13 | -0.52 | 0.26 | 3 | NA | -0.13 | -0.52 | 0.26 | 2 | Imprecision |
| tofacitinib | favipiravir | NA | NA | NA | NA | NA | -0.25 | -1.00 | 0.31 | 3 | NA | -0.25 | -1.00 | 0.31 | 2 | Imprecision |
| vitamin c | favipiravir | NA | NA | NA | NA | NA | -0.24 | -1.16 | 0.71 | 3 | NA | -0.24 | -1.16 | 0.71 | 2 | Imprecision |
| vitamin d3 | favipiravir | NA | NA | NA | NA | NA | -0.31 | -1.14 | 0.48 | 3 | NA | -0.31 | -1.14 | 0.48 | 2 | Imprecision |
| hydroxychloroquine | hydrocortisone | 0.24 | -0.16 | 0.68 | 3 | RoB; | 0.29 | -0.06 | 0.77 | 2 | Intransitivity | 0.37 | 0.13 | 0.65 | 3 | NA |
| hydroxychloroquine + azithromycin | hydrocortisone | NA | NA | NA | NA | NA | -0.39 | -1.75 | 0.75 | 3 | Intransitivity | -0.39 | -1.75 | 0.75 | 1 | Severe Imprecision |
| hydroxychloroquine + favipiravir | hydrocortisone | NA | NA | NA | NA | NA | 0.46 | -0.20 | 1.11 | 2 | Intransitivity | 0.46 | -0.20 | 1.11 | 1 | Imprecision |
| hydroxychloroquine + lopinavir/ritonavir | hydrocortisone | 0.61 | -0.05 | 1.30 | 3 | RoB; | NA | NA | NA | NA | NA | 0.61 | -0.05 | 1.30 | 2 | Imprecision |
| imatinib | hydrocortisone | NA | NA | NA | NA | NA | -0.37 | -0.94 | 0.19 | 4 | NA | -0.37 | -0.94 | 0.19 | 3 | Imprecision |
| inm005 | hydrocortisone | NA | NA | NA | NA | NA | -0.34 | -0.95 | 0.29 | 4 | NA | -0.34 | -0.95 | 0.29 | 3 | Imprecision |
| interferon beta | hydrocortisone | NA | NA | NA | NA | NA | 0.22 | -0.06 | 0.52 | 2 | NA | 0.22 | -0.06 | 0.52 | 1 | Imprecision |
| intravenous immunoglobulin | hydrocortisone | NA | NA | NA | NA | NA | -0.51 | -1.18 | 0.15 | 3 | NA | -0.51 | -1.18 | 0.15 | 2 | Imprecision |
| ivermectin | hydrocortisone | NA | NA | NA | NA | NA | -0.27 | -0.82 | 0.28 | 3 | Intransitivity | -0.27 | -0.82 | 0.28 | 2 | Imprecision |
| lopinavir/ritonavir | hydrocortisone | 0.21 | -0.08 | 0.55 | 3 | RoB; | 0.16 | -0.21 | 0.63 | 3 | NA | 0.20 | -0.04 | 0.47 | 2 | Imprecision |
| mesenchymal stem cells | hydrocortisone | NA | NA | NA | NA | NA | -0.06 | -0.94 | 0.41 | 4 | NA | -0.06 | -0.94 | 0.41 | 3 | Imprecision |
| methylprednisolone | hydrocortisone | NA | NA | NA | NA | NA | 0.08 | -0.24 | 0.44 | 3 | NA | 0.08 | -0.24 | 0.44 | 2 | Imprecision |
| recombinant human gcsf | hydrocortisone | NA | NA | NA | NA | NA | -0.05 | -0.95 | 0.45 | 2 | Intransitivity | -0.05 | -0.95 | 0.45 | 1 | Imprecision |
| remdesivir | hydrocortisone | NA | NA | NA | NA | NA | 0.17 | -0.09 | 0.45 | 3 | NA | 0.17 | -0.09 | 0.45 | 2 | Imprecision |
| sarilumab | hydrocortisone | -0.18 | -0.68 | 0.22 | 3 | RoB; | -0.06 | -0.57 | 0.60 | 3 | NA | -0.05 | -0.47 | 0.36 | 2 | Imprecision |
| sofosbuvir + daclatasvir | hydrocortisone | NA | NA | NA | NA | NA | 0.07 | -0.57 | 0.53 | 3 | NA | 0.07 | -0.57 | 0.53 | 2 | Imprecision |
| sulodexide | hydrocortisone | NA | NA | NA | NA | NA | 0.04 | -0.69 | 0.60 | 2 | Intransitivity | 0.04 | -0.69 | 0.60 | 1 | Imprecision |
| therapeutic anticoagulation | hydrocortisone | NA | NA | NA | NA | NA | 0.17 | -0.12 | 0.47 | 3 | NA | 0.17 | -0.12 | 0.47 | 2 | Imprecision |
| tocilizumab | hydrocortisone | -0.06 | -0.33 | 0.23 | 3 | RoB; | 0.01 | -0.33 | 0.49 | 3 | NA | 0.06 | -0.18 | 0.33 | 2 | Imprecision |
| tofacitinib | hydrocortisone | NA | NA | NA | NA | NA | -0.07 | -0.68 | 0.44 | 4 | NA | -0.07 | -0.68 | 0.44 | 3 | Imprecision |
| vitamin c | hydrocortisone | NA | NA | NA | NA | NA | -0.05 | -0.93 | 0.88 | 3 | NA | -0.05 | -0.93 | 0.88 | 2 | Imprecision |
| vitamin d3 | hydrocortisone | NA | NA | NA | NA | NA | -0.12 | -0.90 | 0.63 | 3 | NA | -0.12 | -0.90 | 0.63 | 2 | Imprecision |
| hydroxychloroquine + azithromycin | hydroxychloroquine | -0.77 | -2.07 | 0.49 | 4 | NA | -0.81 | -2.21 | 0.54 | 3 | NA | -0.77 | -2.11 | 0.35 | 2 | Severe Imprecision |
| hydroxychloroquine + favipiravir | hydroxychloroquine | NA | NA | NA | NA | NA | 0.09 | -0.56 | 0.71 | 3 | NA | 0.09 | -0.56 | 0.71 | 2 | Imprecision |
| hydroxychloroquine + lopinavir/ritonavir | hydroxychloroquine | 0.23 | -0.40 | 0.88 | 3 | RoB; | NA | NA | NA | NA | NA | 0.23 | -0.40 | 0.88 | 2 | Imprecision |
| imatinib | hydroxychloroquine | NA | NA | NA | NA | NA | -0.75 | -1.29 | -0.23 | 2 | Intransitivity | -0.75 | -1.29 | -0.23 | 2 | NA |
| inm005 | hydroxychloroquine | NA | NA | NA | NA | NA | -0.71 | -1.30 | -0.14 | 3 | NA | -0.71 | -1.30 | -0.14 | 3 | NA |
| interferon beta | hydroxychloroquine | 0.01 | -0.23 | 0.26 | 3 | RoB; | -1.11 | -1.76 | -0.49 | 1 | Intransitivity | -0.16 | -0.33 | 0.02 | 2 | Imprecision |
| intravenous immunoglobulin | hydroxychloroquine | NA | NA | NA | NA | NA | -0.89 | -1.52 | -0.27 | 2 | Intransitivity | -0.89 | -1.52 | -0.27 | 2 | NA |
| ivermectin | hydroxychloroquine | -0.72 | -1.31 | -0.16 | 2 | RoB;Inconsistency; | -0.66 | -1.25 | -0.08 | 3 | NA | -0.64 | -1.15 | -0.15 | 3 | NA |
| lopinavir/ritonavir | hydroxychloroquine | -0.16 | -0.29 | -0.03 | 3 | RoB; | -0.39 | -0.97 | 0.02 | 3 | NA | -0.17 | -0.30 | -0.04 | 3 | NA |
| mesenchymal stem cells | hydroxychloroquine | NA | NA | NA | NA | NA | -0.42 | -1.34 | 0.00 | 2 | Intransitivity | -0.42 | -1.34 | 0.00 | 1 | Imprecision |
| methylprednisolone | hydroxychloroquine | NA | NA | NA | NA | NA | -0.29 | -0.60 | 0.00 | 3 | NA | -0.29 | -0.60 | 0.00 | 2 | Imprecision |
| recombinant human gcsf | hydroxychloroquine | NA | NA | NA | NA | NA | -0.41 | -1.35 | 0.04 | 3 | NA | -0.41 | -1.35 | 0.04 | 2 | Imprecision |
| remdesivir | hydroxychloroquine | -0.05 | -0.26 | 0.18 | 3 | RoB; | -0.38 | -0.70 | -0.11 | 3 | NA | -0.20 | -0.35 | -0.05 | 3 | NA |
| sarilumab | hydroxychloroquine | -0.41 | -1.01 | 0.06 | 3 | RoB; | -0.34 | -0.76 | 0.10 | 2 | Intransitivity | -0.42 | -0.83 | -0.08 | 3 | NA |
| sofosbuvir + daclatasvir | hydroxychloroquine | NA | NA | NA | NA | NA | -0.29 | -0.96 | 0.10 | 3 | NA | -0.29 | -0.96 | 0.10 | 2 | Imprecision |
| sulodexide | hydroxychloroquine | NA | NA | NA | NA | NA | -0.32 | -1.09 | 0.17 | 3 | NA | -0.32 | -1.09 | 0.17 | 2 | Imprecision |
| therapeutic anticoagulation | hydroxychloroquine | NA | NA | NA | NA | NA | -0.21 | -0.41 | -0.01 | 3 | NA | -0.21 | -0.41 | -0.01 | 2 | Imprecision |
| tocilizumab | hydroxychloroquine | -0.34 | -0.77 | 0.09 | 3 | RoB; | -0.29 | -0.44 | -0.13 | 3 | NA | -0.31 | -0.46 | -0.17 | 3 | NA |
| tofacitinib | hydroxychloroquine | NA | NA | NA | NA | NA | -0.43 | -1.07 | 0.00 | 3 | NA | -0.43 | -1.07 | 0.00 | 2 | Imprecision |
| vitamin c | hydroxychloroquine | NA | NA | NA | NA | NA | -0.43 | -1.28 | 0.45 | 3 | NA | -0.43 | -1.28 | 0.45 | 2 | Imprecision |
| vitamin d3 | hydroxychloroquine | NA | NA | NA | NA | NA | -0.49 | -1.25 | 0.23 | 3 | NA | -0.49 | -1.25 | 0.23 | 2 | Imprecision |
| hydroxychloroquine + favipiravir | hydroxychloroquine + azithromycin | NA | NA | NA | NA | NA | 0.86 | -0.44 | 2.33 | 3 | NA | 0.86 | -0.44 | 2.33 | 1 | Severe Imprecision |
| hydroxychloroquine + lopinavir/ritonavir | hydroxychloroquine + azithromycin | NA | NA | NA | NA | NA | 1.00 | -0.29 | 2.48 | 2 | Intransitivity | 1.00 | -0.29 | 2.48 | 1 | Severe Imprecision |
| imatinib | hydroxychloroquine + azithromycin | NA | NA | NA | NA | NA | 0.02 | -1.23 | 1.46 | 3 | Intransitivity | 0.02 | -1.23 | 1.46 | 2 | Imprecision |
| inm005 | hydroxychloroquine + azithromycin | NA | NA | NA | NA | NA | 0.06 | -1.21 | 1.52 | 4 | NA | 0.06 | -1.21 | 1.52 | 2 | Severe Imprecision |
| interferon beta | hydroxychloroquine + azithromycin | NA | NA | NA | NA | NA | 0.61 | -0.51 | 1.96 | 1 | Intransitivity | 0.61 | -0.51 | 1.96 | 1 | Severe Imprecision |
| intravenous immunoglobulin | hydroxychloroquine + azithromycin | NA | NA | NA | NA | NA | -0.12 | -1.39 | 1.35 | 2 | Intransitivity | -0.12 | -1.39 | 1.35 | 1 | Imprecision |
| ivermectin | hydroxychloroquine + azithromycin | NA | NA | NA | NA | NA | 0.12 | -1.10 | 1.54 | 4 | NA | 0.12 | -1.10 | 1.54 | 2 | Severe Imprecision |
| lopinavir/ritonavir | hydroxychloroquine + azithromycin | NA | NA | NA | NA | NA | 0.59 | -0.52 | 1.94 | 3 | NA | 0.59 | -0.52 | 1.94 | 1 | Severe Imprecision |
| mesenchymal stem cells | hydroxychloroquine + azithromycin | NA | NA | NA | NA | NA | 0.30 | -1.09 | 1.74 | 3 | Intransitivity | 0.30 | -1.09 | 1.74 | 1 | Severe Imprecision |
| methylprednisolone | hydroxychloroquine + azithromycin | NA | NA | NA | NA | NA | 0.47 | -0.68 | 1.85 | 3 | NA | 0.47 | -0.68 | 1.85 | 1 | Severe Imprecision |
| recombinant human gcsf | hydroxychloroquine + azithromycin | NA | NA | NA | NA | NA | 0.31 | -1.09 | 1.77 | 3 | NA | 0.31 | -1.09 | 1.77 | 1 | Severe Imprecision |
| remdesivir | hydroxychloroquine + azithromycin | NA | NA | NA | NA | NA | 0.56 | -0.56 | 1.91 | 3 | NA | 0.56 | -0.56 | 1.91 | 1 | Severe Imprecision |
| sarilumab | hydroxychloroquine + azithromycin | NA | NA | NA | NA | NA | 0.34 | -0.83 | 1.74 | 2 | Intransitivity | 0.34 | -0.83 | 1.74 | 1 | Severe Imprecision |
| sofosbuvir + daclatasvir | hydroxychloroquine + azithromycin | NA | NA | NA | NA | NA | 0.45 | -0.82 | 1.86 | 3 | NA | 0.45 | -0.82 | 1.86 | 1 | Severe Imprecision |
| sulodexide | hydroxychloroquine + azithromycin | NA | NA | NA | NA | NA | 0.42 | -0.89 | 1.87 | 3 | NA | 0.42 | -0.89 | 1.87 | 1 | Severe Imprecision |
| therapeutic anticoagulation | hydroxychloroquine + azithromycin | NA | NA | NA | NA | NA | 0.56 | -0.57 | 1.92 | 3 | NA | 0.56 | -0.57 | 1.92 | 1 | Severe Imprecision |
| tocilizumab | hydroxychloroquine + azithromycin | NA | NA | NA | NA | NA | 0.45 | -0.67 | 1.80 | 3 | NA | 0.45 | -0.67 | 1.80 | 1 | Severe Imprecision |
| tofacitinib | hydroxychloroquine + azithromycin | NA | NA | NA | NA | NA | 0.32 | -0.94 | 1.74 | 4 | NA | 0.32 | -0.94 | 1.74 | 2 | Severe Imprecision |
| vitamin c | hydroxychloroquine + azithromycin | NA | NA | NA | NA | NA | 0.35 | -1.07 | 1.93 | 3 | NA | 0.35 | -1.07 | 1.93 | 1 | Severe Imprecision |
| vitamin d3 | hydroxychloroquine + azithromycin | NA | NA | NA | NA | NA | 0.28 | -1.08 | 1.78 | 3 | NA | 0.28 | -1.08 | 1.78 | 1 | Severe Imprecision |
| hydroxychloroquine + lopinavir/ritonavir | hydroxychloroquine + favipiravir | NA | NA | NA | NA | NA | 0.11 | -0.32 | 0.82 | 2 | Intransitivity | 0.11 | -0.32 | 0.82 | 1 | Imprecision |
| imatinib | hydroxychloroquine + favipiravir | NA | NA | NA | NA | NA | -0.83 | -1.64 | -0.03 | 2 | Intransitivity | -0.83 | -1.64 | -0.03 | 2 | NA |
| inm005 | hydroxychloroquine + favipiravir | NA | NA | NA | NA | NA | -0.80 | -1.64 | 0.06 | 3 | NA | -0.80 | -1.64 | 0.06 | 1 | Severe Imprecision |
| interferon beta | hydroxychloroquine + favipiravir | NA | NA | NA | NA | NA | -0.24 | -0.87 | 0.42 | 1 | Intransitivity | -0.24 | -0.87 | 0.42 | 1 | Imprecision |
| intravenous immunoglobulin | hydroxychloroquine + favipiravir | NA | NA | NA | NA | NA | -0.98 | -1.85 | -0.09 | 2 | Intransitivity | -0.98 | -1.85 | -0.09 | 2 | NA |
| ivermectin | hydroxychloroquine + favipiravir | NA | NA | NA | NA | NA | -0.73 | -1.52 | 0.07 | 3 | NA | -0.73 | -1.52 | 0.07 | 1 | Severe Imprecision |
| lopinavir/ritonavir | hydroxychloroquine + favipiravir | NA | NA | NA | NA | NA | -0.26 | -0.88 | 0.39 | 3 | NA | -0.26 | -0.88 | 0.39 | 2 | Imprecision |
| mesenchymal stem cells | hydroxychloroquine + favipiravir | NA | NA | NA | NA | NA | -0.55 | -1.52 | 0.21 | 2 | Intransitivity | -0.55 | -1.52 | 0.21 | 1 | Severe Imprecision |
| methylprednisolone | hydroxychloroquine + favipiravir | NA | NA | NA | NA | NA | -0.38 | -1.05 | 0.31 | 3 | NA | -0.38 | -1.05 | 0.31 | 2 | Imprecision |
| recombinant human gcsf | hydroxychloroquine + favipiravir | NA | NA | NA | NA | NA | -0.53 | -1.52 | 0.24 | 3 | NA | -0.53 | -1.52 | 0.24 | 1 | Severe Imprecision |
| remdesivir | hydroxychloroquine + favipiravir | NA | NA | NA | NA | NA | -0.29 | -0.91 | 0.36 | 3 | NA | -0.29 | -0.91 | 0.36 | 2 | Imprecision |
| sarilumab | hydroxychloroquine + favipiravir | NA | NA | NA | NA | NA | -0.51 | -1.21 | 0.20 | 2 | Intransitivity | -0.51 | -1.21 | 0.20 | 1 | Imprecision |
| sofosbuvir + daclatasvir | hydroxychloroquine + favipiravir | NA | NA | NA | NA | NA | -0.40 | -1.21 | 0.34 | 3 | NA | -0.40 | -1.21 | 0.34 | 2 | Imprecision |
| sulodexide | hydroxychloroquine + favipiravir | NA | NA | NA | NA | NA | -0.43 | -1.31 | 0.38 | 3 | NA | -0.43 | -1.31 | 0.38 | 2 | Imprecision |
| therapeutic anticoagulation | hydroxychloroquine + favipiravir | NA | NA | NA | NA | NA | -0.29 | -0.93 | 0.37 | 3 | NA | -0.29 | -0.93 | 0.37 | 2 | Imprecision |
| tocilizumab | hydroxychloroquine + favipiravir | NA | NA | NA | NA | NA | -0.40 | -1.02 | 0.25 | 3 | NA | -0.40 | -1.02 | 0.25 | 2 | Imprecision |
| tofacitinib | hydroxychloroquine + favipiravir | NA | NA | NA | NA | NA | -0.54 | -1.34 | 0.23 | 3 | NA | -0.54 | -1.34 | 0.23 | 2 | Imprecision |
| vitamin c | hydroxychloroquine + favipiravir | NA | NA | NA | NA | NA | -0.52 | -1.56 | 0.60 | 3 | NA | -0.52 | -1.56 | 0.60 | 1 | Severe Imprecision |
| vitamin d3 | hydroxychloroquine + favipiravir | NA | NA | NA | NA | NA | -0.58 | -1.54 | 0.38 | 3 | NA | -0.58 | -1.54 | 0.38 | 1 | Severe Imprecision |
| imatinib | hydroxychloroquine + lopinavir/ritonavir | NA | NA | NA | NA | NA | -0.98 | -1.82 | -0.18 | 3 | NA | -0.98 | -1.82 | -0.18 | 3 | NA |
| inm005 | hydroxychloroquine + lopinavir/ritonavir | NA | NA | NA | NA | NA | -0.94 | -1.82 | -0.11 | 3 | NA | -0.94 | -1.82 | -0.11 | 3 | NA |
| interferon beta | hydroxychloroquine + lopinavir/ritonavir | NA | NA | NA | NA | NA | -0.39 | -1.04 | 0.25 | 2 | NA | -0.39 | -1.04 | 0.25 | 1 | Imprecision |
| intravenous immunoglobulin | hydroxychloroquine + lopinavir/ritonavir | NA | NA | NA | NA | NA | -1.12 | -2.01 | -0.25 | 3 | NA | -1.12 | -2.01 | -0.25 | 3 | NA |
| ivermectin | hydroxychloroquine + lopinavir/ritonavir | NA | NA | NA | NA | NA | -0.88 | -1.68 | -0.09 | 2 | Intransitivity | -0.88 | -1.68 | -0.09 | 2 | NA |
| lopinavir/ritonavir | hydroxychloroquine + lopinavir/ritonavir | -0.40 | -1.04 | 0.22 | 3 | RoB; | NA | NA | NA | NA | NA | -0.40 | -1.04 | 0.22 | 2 | Imprecision |
| mesenchymal stem cells | hydroxychloroquine + lopinavir/ritonavir | NA | NA | NA | NA | NA | -0.67 | -1.85 | 0.11 | 3 | NA | -0.67 | -1.85 | 0.11 | 1 | Severe Imprecision |
| methylprednisolone | hydroxychloroquine + lopinavir/ritonavir | NA | NA | NA | NA | NA | -0.52 | -1.23 | 0.15 | 3 | NA | -0.52 | -1.23 | 0.15 | 2 | Imprecision |
| recombinant human gcsf | hydroxychloroquine + lopinavir/ritonavir | NA | NA | NA | NA | NA | -0.66 | -1.85 | 0.14 | 2 | Intransitivity | -0.66 | -1.85 | 0.14 | 1 | Severe Imprecision |
| remdesivir | hydroxychloroquine + lopinavir/ritonavir | NA | NA | NA | NA | NA | -0.43 | -1.08 | 0.20 | 3 | NA | -0.43 | -1.08 | 0.20 | 2 | Imprecision |
| sarilumab | hydroxychloroquine + lopinavir/ritonavir | -0.65 | -1.41 | 0.05 | 3 | RoB; | NA | NA | NA | NA | NA | -0.65 | -1.41 | 0.05 | 2 | Imprecision |
| sofosbuvir + daclatasvir | hydroxychloroquine + lopinavir/ritonavir | NA | NA | NA | NA | NA | -0.54 | -1.48 | 0.20 | 3 | NA | -0.54 | -1.48 | 0.20 | 2 | Imprecision |
| sulodexide | hydroxychloroquine + lopinavir/ritonavir | NA | NA | NA | NA | NA | -0.56 | -1.59 | 0.23 | 2 | Intransitivity | -0.56 | -1.59 | 0.23 | 1 | Severe Imprecision |
| therapeutic anticoagulation | hydroxychloroquine + lopinavir/ritonavir | NA | NA | NA | NA | NA | -0.44 | -1.10 | 0.20 | 3 | NA | -0.44 | -1.10 | 0.20 | 2 | Imprecision |
| tocilizumab | hydroxychloroquine + lopinavir/ritonavir | -0.54 | -1.18 | 0.08 | 3 | RoB; | NA | NA | NA | NA | NA | -0.54 | -1.18 | 0.08 | 2 | Imprecision |
| tofacitinib | hydroxychloroquine + lopinavir/ritonavir | NA | NA | NA | NA | NA | -0.67 | -1.59 | 0.08 | 3 | NA | -0.67 | -1.59 | 0.08 | 1 | Severe Imprecision |
| vitamin c | hydroxychloroquine + lopinavir/ritonavir | NA | NA | NA | NA | NA | -0.65 | -1.71 | 0.40 | 3 | NA | -0.65 | -1.71 | 0.40 | 1 | Severe Imprecision |
| vitamin d3 | hydroxychloroquine + lopinavir/ritonavir | NA | NA | NA | NA | NA | -0.73 | -1.70 | 0.22 | 3 | NA | -0.73 | -1.70 | 0.22 | 1 | Severe Imprecision |
| inm005 | imatinib | NA | NA | NA | NA | NA | 0.03 | -0.48 | 0.60 | 4 | NA | 0.03 | -0.48 | 0.60 | 3 | Imprecision |
| interferon beta | imatinib | NA | NA | NA | NA | NA | 0.59 | 0.06 | 1.15 | 2 | NA | 0.59 | 0.06 | 1.15 | 2 | NA |
| intravenous immunoglobulin | imatinib | NA | NA | NA | NA | NA | -0.14 | -0.94 | 0.67 | 3 | NA | -0.14 | -0.94 | 0.67 | 2 | Imprecision |
| ivermectin | imatinib | NA | NA | NA | NA | NA | 0.10 | -0.61 | 0.83 | 3 | Intransitivity | 0.10 | -0.61 | 0.83 | 2 | Imprecision |
| lopinavir/ritonavir | imatinib | NA | NA | NA | NA | NA | 0.58 | 0.06 | 1.12 | 3 | NA | 0.58 | 0.06 | 1.12 | 3 | NA |
| mesenchymal stem cells | imatinib | NA | NA | NA | NA | NA | 0.30 | -0.68 | 0.97 | 4 | NA | 0.30 | -0.68 | 0.97 | 3 | Imprecision |
| methylprednisolone | imatinib | NA | NA | NA | NA | NA | 0.45 | -0.13 | 1.05 | 3 | NA | 0.45 | -0.13 | 1.05 | 2 | Imprecision |
| recombinant human gcsf | imatinib | NA | NA | NA | NA | NA | 0.31 | -0.69 | 1.00 | 2 | Intransitivity | 0.31 | -0.69 | 1.00 | 1 | Imprecision |
| remdesivir | imatinib | NA | NA | NA | NA | NA | 0.54 | 0.03 | 1.09 | 3 | NA | 0.54 | 0.03 | 1.09 | 3 | NA |
| sarilumab | imatinib | NA | NA | NA | NA | NA | 0.32 | -0.30 | 0.95 | 3 | NA | 0.32 | -0.30 | 0.95 | 2 | Imprecision |
| sofosbuvir + daclatasvir | imatinib | NA | NA | NA | NA | NA | 0.44 | -0.34 | 1.09 | 3 | NA | 0.44 | -0.34 | 1.09 | 2 | Imprecision |
| sulodexide | imatinib | NA | NA | NA | NA | NA | 0.41 | -0.45 | 1.13 | 2 | Intransitivity | 0.41 | -0.45 | 1.13 | 1 | Imprecision |
| therapeutic anticoagulation | imatinib | NA | NA | NA | NA | NA | 0.54 | 0.01 | 1.09 | 3 | NA | 0.54 | 0.01 | 1.09 | 2 | Imprecision |
| tocilizumab | imatinib | NA | NA | NA | NA | NA | 0.43 | -0.08 | 0.97 | 3 | NA | 0.43 | -0.08 | 0.97 | 2 | Imprecision |
| tofacitinib | imatinib | NA | NA | NA | NA | NA | 0.30 | -0.46 | 0.98 | 4 | NA | 0.30 | -0.46 | 0.98 | 3 | Imprecision |
| vitamin c | imatinib | NA | NA | NA | NA | NA | 0.32 | -0.66 | 1.36 | 3 | NA | 0.32 | -0.66 | 1.36 | 2 | Imprecision |
| vitamin d3 | imatinib | NA | NA | NA | NA | NA | 0.25 | -0.64 | 1.14 | 3 | NA | 0.25 | -0.64 | 1.14 | 2 | Imprecision |
| interferon beta | inm005 | NA | NA | NA | NA | NA | 0.55 | -0.03 | 1.16 | 2 | NA | 0.55 | -0.03 | 1.16 | 1 | Imprecision |
| intravenous immunoglobulin | inm005 | NA | NA | NA | NA | NA | -0.18 | -1.02 | 0.67 | 3 | NA | -0.18 | -1.02 | 0.67 | 2 | Imprecision |
| ivermectin | inm005 | NA | NA | NA | NA | NA | 0.07 | -0.69 | 0.83 | 4 | NA | 0.07 | -0.69 | 0.83 | 3 | Imprecision |
| lopinavir/ritonavir | inm005 | NA | NA | NA | NA | NA | 0.54 | -0.03 | 1.13 | 3 | NA | 0.54 | -0.03 | 1.13 | 2 | Imprecision |
| mesenchymal stem cells | inm005 | NA | NA | NA | NA | NA | 0.27 | -0.80 | 0.97 | 4 | NA | 0.27 | -0.80 | 0.97 | 3 | Imprecision |
| methylprednisolone | inm005 | NA | NA | NA | NA | NA | 0.42 | -0.22 | 1.06 | 3 | NA | 0.42 | -0.22 | 1.06 | 2 | Imprecision |
| recombinant human gcsf | inm005 | NA | NA | NA | NA | NA | 0.28 | -0.81 | 1.00 | 3 | NA | 0.28 | -0.81 | 1.00 | 2 | Imprecision |
| remdesivir | inm005 | NA | NA | NA | NA | NA | 0.51 | -0.07 | 1.10 | 3 | NA | 0.51 | -0.07 | 1.10 | 2 | Imprecision |
| sarilumab | inm005 | NA | NA | NA | NA | NA | 0.29 | -0.41 | 0.95 | 3 | NA | 0.29 | -0.41 | 0.95 | 2 | Imprecision |
| sofosbuvir + daclatasvir | inm005 | NA | NA | NA | NA | NA | 0.40 | -0.45 | 1.09 | 3 | NA | 0.40 | -0.45 | 1.09 | 2 | Imprecision |
| sulodexide | inm005 | NA | NA | NA | NA | NA | 0.38 | -0.56 | 1.13 | 3 | NA | 0.38 | -0.56 | 1.13 | 2 | Imprecision |
| therapeutic anticoagulation | inm005 | NA | NA | NA | NA | NA | 0.50 | -0.09 | 1.11 | 3 | NA | 0.50 | -0.09 | 1.11 | 2 | Imprecision |
| tocilizumab | inm005 | NA | NA | NA | NA | NA | 0.40 | -0.18 | 0.99 | 3 | NA | 0.40 | -0.18 | 0.99 | 2 | Imprecision |
| tofacitinib | inm005 | NA | NA | NA | NA | NA | 0.27 | -0.57 | 0.98 | 4 | NA | 0.27 | -0.57 | 0.98 | 3 | Imprecision |
| vitamin c | inm005 | NA | NA | NA | NA | NA | 0.29 | -0.73 | 1.35 | 3 | NA | 0.29 | -0.73 | 1.35 | 2 | Imprecision |
| vitamin d3 | inm005 | NA | NA | NA | NA | NA | 0.22 | -0.72 | 1.14 | 3 | NA | 0.22 | -0.72 | 1.14 | 2 | Imprecision |
| intravenous immunoglobulin | interferon beta | NA | NA | NA | NA | NA | -0.73 | -1.37 | -0.10 | 2 | NA | -0.73 | -1.37 | -0.10 | 2 | NA |
| ivermectin | interferon beta | NA | NA | NA | NA | NA | -0.49 | -1.01 | 0.02 | 1 | Intransitivity | -0.49 | -1.01 | 0.02 | 1 | Imprecision |
| lopinavir/ritonavir | interferon beta | -0.07 | -0.29 | 0.14 | 3 | RoB; | 0.87 | 0.25 | 1.55 | 2 | NA | -0.02 | -0.18 | 0.16 | 2 | Imprecision |
| mesenchymal stem cells | interferon beta | NA | NA | NA | NA | NA | -0.27 | -1.20 | 0.18 | 2 | NA | -0.27 | -1.20 | 0.18 | 1 | Imprecision |
| methylprednisolone | interferon beta | NA | NA | NA | NA | NA | -0.14 | -0.47 | 0.18 | 2 | NA | -0.14 | -0.47 | 0.18 | 1 | Imprecision |
| recombinant human gcsf | interferon beta | NA | NA | NA | NA | NA | -0.24 | -1.20 | 0.16 | 1 | Intransitivity | -0.24 | -1.20 | 0.16 | 1 | Imprecision |
| remdesivir | interferon beta | -0.05 | -0.23 | 0.13 | 3 | RoB; | 0.65 | 0.03 | 1.34 | 2 | NA | -0.05 | -0.22 | 0.13 | 2 | Imprecision |
| sarilumab | interferon beta | NA | NA | NA | NA | NA | -0.26 | -0.69 | 0.10 | 2 | NA | -0.26 | -0.69 | 0.10 | 1 | Imprecision |
| sofosbuvir + daclatasvir | interferon beta | NA | NA | NA | NA | NA | -0.14 | -0.81 | 0.27 | 2 | NA | -0.14 | -0.81 | 0.27 | 1 | Imprecision |
| sulodexide | interferon beta | NA | NA | NA | NA | NA | -0.16 | -0.94 | 0.34 | 1 | Intransitivity | -0.16 | -0.94 | 0.34 | 1 | Imprecision |
| therapeutic anticoagulation | interferon beta | NA | NA | NA | NA | NA | -0.05 | -0.28 | 0.18 | 2 | NA | -0.05 | -0.28 | 0.18 | 1 | Imprecision |
| tocilizumab | interferon beta | NA | NA | NA | NA | NA | -0.16 | -0.34 | 0.03 | 2 | NA | -0.16 | -0.34 | 0.03 | 1 | Imprecision |
| tofacitinib | interferon beta | NA | NA | NA | NA | NA | -0.28 | -0.92 | 0.17 | 2 | NA | -0.28 | -0.92 | 0.17 | 1 | Imprecision |
| vitamin c | interferon beta | NA | NA | NA | NA | NA | -0.27 | -1.13 | 0.62 | 2 | NA | -0.27 | -1.13 | 0.62 | 1 | Imprecision |
| vitamin d3 | interferon beta | NA | NA | NA | NA | NA | -0.34 | -1.10 | 0.39 | 2 | NA | -0.34 | -1.10 | 0.39 | 1 | Imprecision |
| ivermectin | intravenous immunoglobulin | NA | NA | NA | NA | NA | 0.24 | -0.55 | 1.04 | 2 | Intransitivity | 0.24 | -0.55 | 1.04 | 1 | Imprecision |
| lopinavir/ritonavir | intravenous immunoglobulin | NA | NA | NA | NA | NA | 0.72 | 0.10 | 1.35 | 3 | NA | 0.72 | 0.10 | 1.35 | 3 | NA |
| mesenchymal stem cells | intravenous immunoglobulin | NA | NA | NA | NA | NA | 0.43 | -0.61 | 1.22 | 3 | NA | 0.43 | -0.61 | 1.22 | 2 | Imprecision |
| methylprednisolone | intravenous immunoglobulin | NA | NA | NA | NA | NA | 0.59 | -0.08 | 1.27 | 3 | NA | 0.59 | -0.08 | 1.27 | 2 | Imprecision |
| recombinant human gcsf | intravenous immunoglobulin | NA | NA | NA | NA | NA | 0.44 | -0.62 | 1.25 | 2 | Intransitivity | 0.44 | -0.62 | 1.25 | 1 | Imprecision |
| remdesivir | intravenous immunoglobulin | NA | NA | NA | NA | NA | 0.69 | 0.06 | 1.32 | 3 | NA | 0.69 | 0.06 | 1.32 | 3 | NA |
| sarilumab | intravenous immunoglobulin | NA | NA | NA | NA | NA | 0.46 | -0.25 | 1.18 | 3 | NA | 0.46 | -0.25 | 1.18 | 2 | Imprecision |
| sofosbuvir + daclatasvir | intravenous immunoglobulin | NA | NA | NA | NA | NA | 0.57 | -0.28 | 1.32 | 3 | NA | 0.57 | -0.28 | 1.32 | 2 | Imprecision |
| sulodexide | intravenous immunoglobulin | NA | NA | NA | NA | NA | 0.55 | -0.38 | 1.36 | 2 | Intransitivity | 0.55 | -0.38 | 1.36 | 1 | Imprecision |
| therapeutic anticoagulation | intravenous immunoglobulin | NA | NA | NA | NA | NA | 0.68 | 0.04 | 1.33 | 3 | NA | 0.68 | 0.04 | 1.33 | 3 | NA |
| tocilizumab | intravenous immunoglobulin | NA | NA | NA | NA | NA | 0.57 | -0.05 | 1.21 | 3 | NA | 0.57 | -0.05 | 1.21 | 2 | Imprecision |
| tofacitinib | intravenous immunoglobulin | NA | NA | NA | NA | NA | 0.44 | -0.40 | 1.21 | 3 | NA | 0.44 | -0.40 | 1.21 | 2 | Imprecision |
| vitamin c | intravenous immunoglobulin | NA | NA | NA | NA | NA | 0.47 | -0.59 | 1.53 | 3 | NA | 0.47 | -0.59 | 1.53 | 1 | Severe Imprecision |
| vitamin d3 | intravenous immunoglobulin | NA | NA | NA | NA | NA | 0.39 | -0.57 | 1.34 | 3 | NA | 0.39 | -0.57 | 1.34 | 2 | Imprecision |
| lopinavir/ritonavir | ivermectin | 0.42 | -0.46 | 1.27 | 4 | NA | 0.47 | -0.03 | 1.00 | 3 | NA | 0.47 | -0.03 | 0.98 | 3 | Imprecision |
| mesenchymal stem cells | ivermectin | NA | NA | NA | NA | NA | 0.19 | -0.80 | 0.88 | 3 | Intransitivity | 0.19 | -0.80 | 0.88 | 2 | Imprecision |
| methylprednisolone | ivermectin | NA | NA | NA | NA | NA | 0.35 | -0.22 | 0.92 | 3 | NA | 0.35 | -0.22 | 0.92 | 2 | Imprecision |
| recombinant human gcsf | ivermectin | NA | NA | NA | NA | NA | 0.21 | -0.81 | 0.91 | 3 | NA | 0.21 | -0.81 | 0.91 | 2 | Imprecision |
| remdesivir | ivermectin | NA | NA | NA | NA | NA | 0.44 | -0.06 | 0.96 | 3 | NA | 0.44 | -0.06 | 0.96 | 2 | Imprecision |
| sarilumab | ivermectin | NA | NA | NA | NA | NA | 0.22 | -0.40 | 0.83 | 2 | Intransitivity | 0.22 | -0.40 | 0.83 | 1 | Imprecision |
| sofosbuvir + daclatasvir | ivermectin | NA | NA | NA | NA | NA | 0.33 | -0.45 | 0.98 | 3 | NA | 0.33 | -0.45 | 0.98 | 2 | Imprecision |
| sulodexide | ivermectin | NA | NA | NA | NA | NA | 0.31 | -0.56 | 1.02 | 3 | NA | 0.31 | -0.56 | 1.02 | 2 | Imprecision |
| therapeutic anticoagulation | ivermectin | NA | NA | NA | NA | NA | 0.44 | -0.08 | 0.97 | 3 | NA | 0.44 | -0.08 | 0.97 | 2 | Imprecision |
| tocilizumab | ivermectin | NA | NA | NA | NA | NA | 0.33 | -0.17 | 0.85 | 3 | NA | 0.33 | -0.17 | 0.85 | 2 | Imprecision |
| tofacitinib | ivermectin | NA | NA | NA | NA | NA | 0.20 | -0.57 | 0.87 | 4 | NA | 0.20 | -0.57 | 0.87 | 3 | Imprecision |
| vitamin c | ivermectin | NA | NA | NA | NA | NA | 0.22 | -0.76 | 1.22 | 3 | NA | 0.22 | -0.76 | 1.22 | 2 | Imprecision |
| vitamin d3 | ivermectin | NA | NA | NA | NA | NA | 0.15 | -0.73 | 1.02 | 3 | NA | 0.15 | -0.73 | 1.02 | 2 | Imprecision |
| mesenchymal stem cells | lopinavir/ritonavir | NA | NA | NA | NA | NA | -0.25 | -1.17 | 0.17 | 3 | NA | -0.25 | -1.17 | 0.17 | 2 | Imprecision |
| methylprednisolone | lopinavir/ritonavir | NA | NA | NA | NA | NA | -0.12 | -0.43 | 0.17 | 3 | NA | -0.12 | -0.43 | 0.17 | 2 | Imprecision |
| recombinant human gcsf | lopinavir/ritonavir | NA | NA | NA | NA | NA | -0.24 | -1.18 | 0.21 | 3 | NA | -0.24 | -1.18 | 0.21 | 2 | Imprecision |
| remdesivir | lopinavir/ritonavir | 0.03 | -0.14 | 0.21 | 3 | RoB; | -0.17 | -0.46 | 0.08 | 3 | NA | -0.03 | -0.18 | 0.11 | 2 | Imprecision |
| sarilumab | lopinavir/ritonavir | -0.30 | -0.87 | 0.10 | 3 | RoB; | -0.18 | -0.61 | 0.28 | 3 | NA | -0.25 | -0.66 | 0.09 | 2 | Imprecision |
| sofosbuvir + daclatasvir | lopinavir/ritonavir | NA | NA | NA | NA | NA | -0.12 | -0.78 | 0.26 | 3 | NA | -0.12 | -0.78 | 0.26 | 2 | Imprecision |
| sulodexide | lopinavir/ritonavir | NA | NA | NA | NA | NA | -0.15 | -0.92 | 0.34 | 3 | NA | -0.15 | -0.92 | 0.34 | 2 | Imprecision |
| therapeutic anticoagulation | lopinavir/ritonavir | NA | NA | NA | NA | NA | -0.04 | -0.23 | 0.16 | 3 | NA | -0.04 | -0.23 | 0.16 | 2 | Imprecision |
| tocilizumab | lopinavir/ritonavir | -0.27 | -0.58 | 0.00 | 3 | RoB; | -0.11 | -0.26 | 0.04 | 3 | NA | -0.14 | -0.28 | 0.00 | 2 | Imprecision |
| tofacitinib | lopinavir/ritonavir | NA | NA | NA | NA | NA | -0.26 | -0.90 | 0.17 | 3 | NA | -0.26 | -0.90 | 0.17 | 2 | Imprecision |
| vitamin c | lopinavir/ritonavir | NA | NA | NA | NA | NA | -0.26 | -1.11 | 0.62 | 3 | NA | -0.26 | -1.11 | 0.62 | 2 | Imprecision |
| vitamin d3 | lopinavir/ritonavir | NA | NA | NA | NA | NA | -0.32 | -1.07 | 0.40 | 3 | NA | -0.32 | -1.07 | 0.40 | 2 | Imprecision |
| methylprednisolone | mesenchymal stem cells | NA | NA | NA | NA | NA | 0.13 | -0.35 | 1.07 | 3 | NA | 0.13 | -0.35 | 1.07 | 2 | Imprecision |
| recombinant human gcsf | mesenchymal stem cells | NA | NA | NA | NA | NA | 0.02 | -0.73 | 0.75 | 2 | Intransitivity | 0.02 | -0.73 | 0.75 | 1 | Imprecision |
| remdesivir | mesenchymal stem cells | NA | NA | NA | NA | NA | 0.22 | -0.21 | 1.14 | 3 | NA | 0.22 | -0.21 | 1.14 | 2 | Imprecision |
| sarilumab | mesenchymal stem cells | NA | NA | NA | NA | NA | 0.01 | -0.51 | 0.93 | 3 | NA | 0.01 | -0.51 | 0.93 | 2 | Imprecision |
| sofosbuvir + daclatasvir | mesenchymal stem cells | NA | NA | NA | NA | NA | 0.12 | -0.46 | 0.99 | 3 | NA | 0.12 | -0.46 | 0.99 | 2 | Imprecision |
| sulodexide | mesenchymal stem cells | NA | NA | NA | NA | NA | 0.10 | -0.56 | 1.03 | 2 | Intransitivity | 0.10 | -0.56 | 1.03 | 1 | Imprecision |
| therapeutic anticoagulation | mesenchymal stem cells | NA | NA | NA | NA | NA | 0.22 | -0.23 | 1.15 | 3 | NA | 0.22 | -0.23 | 1.15 | 2 | Imprecision |
| tocilizumab | mesenchymal stem cells | NA | NA | NA | NA | NA | 0.11 | -0.32 | 1.03 | 3 | NA | 0.11 | -0.32 | 1.03 | 2 | Imprecision |
| tofacitinib | mesenchymal stem cells | NA | NA | NA | NA | NA | -0.01 | -0.61 | 0.93 | 4 | NA | -0.01 | -0.61 | 0.93 | 3 | Imprecision |
| vitamin c | mesenchymal stem cells | NA | NA | NA | NA | NA | 0.02 | -0.94 | 1.36 | 3 | NA | 0.02 | -0.94 | 1.36 | 2 | Imprecision |
| vitamin d3 | mesenchymal stem cells | NA | NA | NA | NA | NA | -0.03 | -0.92 | 1.06 | 3 | NA | -0.03 | -0.92 | 1.06 | 2 | Imprecision |
| recombinant human gcsf | methylprednisolone | NA | NA | NA | NA | NA | -0.12 | -1.08 | 0.39 | 3 | NA | -0.12 | -1.08 | 0.39 | 2 | Imprecision |
| remdesivir | methylprednisolone | NA | NA | NA | NA | NA | 0.09 | -0.21 | 0.40 | 3 | NA | 0.09 | -0.21 | 0.40 | 2 | Imprecision |
| sarilumab | methylprednisolone | NA | NA | NA | NA | NA | -0.13 | -0.60 | 0.30 | 3 | NA | -0.13 | -0.60 | 0.30 | 2 | Imprecision |
| sofosbuvir + daclatasvir | methylprednisolone | NA | NA | NA | NA | NA | -0.01 | -0.70 | 0.47 | 3 | NA | -0.01 | -0.70 | 0.47 | 2 | Imprecision |
| sulodexide | methylprednisolone | NA | NA | NA | NA | NA | -0.03 | -0.82 | 0.53 | 3 | NA | -0.03 | -0.82 | 0.53 | 2 | Imprecision |
| therapeutic anticoagulation | methylprednisolone | NA | NA | NA | NA | NA | 0.08 | -0.24 | 0.42 | 3 | NA | 0.08 | -0.24 | 0.42 | 2 | Imprecision |
| tocilizumab | methylprednisolone | NA | NA | NA | NA | NA | -0.02 | -0.31 | 0.29 | 3 | NA | -0.02 | -0.31 | 0.29 | 2 | Imprecision |
| tofacitinib | methylprednisolone | NA | NA | NA | NA | NA | -0.14 | -0.81 | 0.37 | 3 | NA | -0.14 | -0.81 | 0.37 | 2 | Imprecision |
| vitamin c | methylprednisolone | NA | NA | NA | NA | NA | -0.13 | -1.03 | 0.80 | 3 | NA | -0.13 | -1.03 | 0.80 | 2 | Imprecision |
| vitamin d3 | methylprednisolone | NA | NA | NA | NA | NA | -0.20 | -0.99 | 0.57 | 3 | NA | -0.20 | -0.99 | 0.57 | 2 | Imprecision |
| remdesivir | recombinant human gcsf | NA | NA | NA | NA | NA | 0.21 | -0.24 | 1.15 | 3 | NA | 0.21 | -0.24 | 1.15 | 2 | Imprecision |
| sarilumab | recombinant human gcsf | NA | NA | NA | NA | NA | 0.00 | -0.55 | 0.94 | 2 | Intransitivity | 0.00 | -0.55 | 0.94 | 1 | Imprecision |
| sofosbuvir + daclatasvir | recombinant human gcsf | NA | NA | NA | NA | NA | 0.11 | -0.49 | 1.00 | 3 | NA | 0.11 | -0.49 | 1.00 | 2 | Imprecision |
| sulodexide | recombinant human gcsf | NA | NA | NA | NA | NA | 0.08 | -0.59 | 1.04 | 3 | NA | 0.08 | -0.59 | 1.04 | 2 | Imprecision |
| therapeutic anticoagulation | recombinant human gcsf | NA | NA | NA | NA | NA | 0.20 | -0.27 | 1.16 | 3 | NA | 0.20 | -0.27 | 1.16 | 2 | Imprecision |
| tocilizumab | recombinant human gcsf | NA | NA | NA | NA | NA | 0.10 | -0.36 | 1.04 | 3 | NA | 0.10 | -0.36 | 1.04 | 2 | Imprecision |
| tofacitinib | recombinant human gcsf | NA | NA | NA | NA | NA | -0.03 | -0.64 | 0.94 | 3 | NA | -0.03 | -0.64 | 0.94 | 2 | Imprecision |
| vitamin c | recombinant human gcsf | NA | NA | NA | NA | NA | 0.01 | -0.97 | 1.36 | 3 | NA | 0.01 | -0.97 | 1.36 | 2 | Imprecision |
| vitamin d3 | recombinant human gcsf | NA | NA | NA | NA | NA | -0.05 | -0.95 | 1.06 | 3 | NA | -0.05 | -0.95 | 1.06 | 2 | Imprecision |
| sarilumab | remdesivir | NA | NA | NA | NA | NA | -0.22 | -0.63 | 0.14 | 3 | NA | -0.22 | -0.63 | 0.14 | 2 | Imprecision |
| sofosbuvir + daclatasvir | remdesivir | NA | NA | NA | NA | NA | -0.09 | -0.75 | 0.29 | 3 | NA | -0.09 | -0.75 | 0.29 | 2 | Imprecision |
| sulodexide | remdesivir | NA | NA | NA | NA | NA | -0.12 | -0.89 | 0.38 | 3 | NA | -0.12 | -0.89 | 0.38 | 2 | Imprecision |
| therapeutic anticoagulation | remdesivir | NA | NA | NA | NA | NA | -0.01 | -0.22 | 0.21 | 3 | NA | -0.01 | -0.22 | 0.21 | 2 | Imprecision |
| tocilizumab | remdesivir | NA | NA | NA | NA | NA | -0.11 | -0.27 | 0.06 | 3 | NA | -0.11 | -0.27 | 0.06 | 2 | Imprecision |
| tofacitinib | remdesivir | NA | NA | NA | NA | NA | -0.23 | -0.87 | 0.21 | 3 | NA | -0.23 | -0.87 | 0.21 | 2 | Imprecision |
| vitamin c | remdesivir | NA | NA | NA | NA | NA | -0.22 | -1.08 | 0.66 | 3 | NA | -0.22 | -1.08 | 0.66 | 2 | Imprecision |
| vitamin d3 | remdesivir | NA | NA | NA | NA | NA | -0.29 | -1.04 | 0.43 | 3 | NA | -0.29 | -1.04 | 0.43 | 2 | Imprecision |
| sofosbuvir + daclatasvir | sarilumab | NA | NA | NA | NA | NA | 0.12 | -0.57 | 0.65 | 3 | NA | 0.12 | -0.57 | 0.65 | 2 | Imprecision |
| sulodexide | sarilumab | NA | NA | NA | NA | NA | 0.09 | -0.69 | 0.71 | 2 | Intransitivity | 0.09 | -0.69 | 0.71 | 1 | Imprecision |
| therapeutic anticoagulation | sarilumab | NA | NA | NA | NA | NA | 0.21 | -0.16 | 0.64 | 3 | NA | 0.21 | -0.16 | 0.64 | 2 | Imprecision |
| tocilizumab | sarilumab | 0.06 | -0.26 | 0.51 | 3 | RoB; | 0.02 | -0.44 | 0.47 | 3 | NA | 0.10 | -0.22 | 0.51 | 2 | Imprecision |
| tofacitinib | sarilumab | NA | NA | NA | NA | NA | -0.02 | -0.62 | 0.47 | 3 | NA | -0.02 | -0.62 | 0.47 | 2 | Imprecision |
| vitamin c | sarilumab | NA | NA | NA | NA | NA | 0.00 | -0.91 | 0.98 | 3 | NA | 0.00 | -0.91 | 0.98 | 2 | Imprecision |
| vitamin d3 | sarilumab | NA | NA | NA | NA | NA | -0.07 | -0.89 | 0.73 | 3 | NA | -0.07 | -0.89 | 0.73 | 2 | Imprecision |
| sulodexide | sofosbuvir + daclatasvir | NA | NA | NA | NA | NA | -0.03 | -0.78 | 0.72 | 3 | NA | -0.03 | -0.78 | 0.72 | 2 | Imprecision |
| therapeutic anticoagulation | sofosbuvir + daclatasvir | NA | NA | NA | NA | NA | 0.09 | -0.33 | 0.76 | 3 | NA | 0.09 | -0.33 | 0.76 | 2 | Imprecision |
| tocilizumab | sofosbuvir + daclatasvir | NA | NA | NA | NA | NA | -0.02 | -0.41 | 0.65 | 3 | NA | -0.02 | -0.41 | 0.65 | 2 | Imprecision |
| tofacitinib | sofosbuvir + daclatasvir | NA | NA | NA | NA | NA | -0.14 | -0.80 | 0.60 | 3 | NA | -0.14 | -0.80 | 0.60 | 2 | Imprecision |
| vitamin c | sofosbuvir + daclatasvir | NA | NA | NA | NA | NA | -0.12 | -1.04 | 1.01 | 3 | NA | -0.12 | -1.04 | 1.01 | 2 | Imprecision |
| vitamin d3 | sofosbuvir + daclatasvir | NA | NA | NA | NA | NA | -0.18 | -1.03 | 0.74 | 3 | NA | -0.18 | -1.03 | 0.74 | 2 | Imprecision |
| therapeutic anticoagulation | sulodexide | NA | NA | NA | NA | NA | 0.10 | -0.35 | 0.87 | 3 | NA | 0.10 | -0.35 | 0.87 | 2 | Imprecision |
| tocilizumab | sulodexide | NA | NA | NA | NA | NA | 0.01 | -0.48 | 0.78 | 3 | NA | 0.01 | -0.48 | 0.78 | 2 | Imprecision |
| tofacitinib | sulodexide | NA | NA | NA | NA | NA | -0.11 | -0.84 | 0.71 | 3 | NA | -0.11 | -0.84 | 0.71 | 2 | Imprecision |
| vitamin c | sulodexide | NA | NA | NA | NA | NA | -0.09 | -1.07 | 1.11 | 3 | NA | -0.09 | -1.07 | 1.11 | 2 | Imprecision |
| vitamin d3 | sulodexide | NA | NA | NA | NA | NA | -0.15 | -1.06 | 0.84 | 3 | NA | -0.15 | -1.06 | 0.84 | 2 | Imprecision |
| tocilizumab | therapeutic anticoagulation | NA | NA | NA | NA | NA | -0.10 | -0.31 | 0.10 | 3 | NA | -0.10 | -0.31 | 0.10 | 2 | Imprecision |
| tofacitinib | therapeutic anticoagulation | NA | NA | NA | NA | NA | -0.22 | -0.87 | 0.23 | 3 | NA | -0.22 | -0.87 | 0.23 | 2 | Imprecision |
| vitamin c | therapeutic anticoagulation | NA | NA | NA | NA | NA | -0.22 | -1.08 | 0.67 | 3 | NA | -0.22 | -1.08 | 0.67 | 2 | Imprecision |
| vitamin d3 | therapeutic anticoagulation | NA | NA | NA | NA | NA | -0.29 | -1.05 | 0.45 | 3 | NA | -0.29 | -1.05 | 0.45 | 2 | Imprecision |
| tofacitinib | tocilizumab | NA | NA | NA | NA | NA | -0.11 | -0.75 | 0.30 | 3 | NA | -0.11 | -0.75 | 0.30 | 2 | Imprecision |
| vitamin c | tocilizumab | NA | NA | NA | NA | NA | -0.11 | -0.97 | 0.77 | 3 | NA | -0.11 | -0.97 | 0.77 | 2 | Imprecision |
| vitamin d3 | tocilizumab | NA | NA | NA | NA | NA | -0.18 | -0.93 | 0.54 | 3 | NA | -0.18 | -0.93 | 0.54 | 2 | Imprecision |
| vitamin c | tofacitinib | NA | NA | NA | NA | NA | 0.01 | -0.94 | 1.13 | 3 | NA | 0.01 | -0.94 | 1.13 | 2 | Imprecision |
| vitamin d3 | tofacitinib | NA | NA | NA | NA | NA | -0.05 | -0.92 | 0.87 | 3 | NA | -0.05 | -0.92 | 0.87 | 2 | Imprecision |
| vitamin d3 | vitamin c | NA | NA | NA | NA | NA | -0.05 | -0.76 | 0.48 | 3 | NA | -0.05 | -0.76 | 0.48 | 2 | Imprecision |

*EST: estimate; LCrI: lower credible interval; UCrI: upper credible interval. ^†^Evidence: 4 (High), 3 (Moderate), 2 (Low), 1 (Very low).

## **Table S10. Network meta-analysis results of the primary analysis (log odds ratio, log OR and corresponding 95% equal-tailed Bayesian credible intervals) (mechanical ventilation).**

|  |  | **Direct** | | | | | **Indirect** | | | | | **Network** | | | | |
| --- | --- | --- | --- | --- | --- | --- | --- | --- | --- | --- | --- | --- | --- | --- | --- | --- |
| **Treatment 1** | **Treatment 2** | **EST^*^** | **LCrI^*^** | **UCrI^*^** | **Evidence**^†^ | **Reason** | **EST** | **LCrI** | **UCrI** | **Evidence** | **Reason** | **EST** | **LCrI** | **UCrI** | **Evidence** | **Reason** |
| aceis/arbs | soc | -0.13 | -0.54 | 0.28 | 3 | RoB; | NA | NA | NA | NA | NA | -0.13 | -0.54 | 0.28 | 2 | Imprecision |
| ammonium chloride | soc | -0.25 | -0.94 | 0.40 | 4 | NA | NA | NA | NA | NA | NA | -0.25 | -0.94 | 0.40 | 3 | Imprecision |
| azithromycin | soc | -0.06 | -0.21 | 0.10 | 3 | RoB; | -0.40 | -1.17 | 0.36 | 3 | NA | -0.07 | -0.22 | 0.07 | 2 | Imprecision |
| bamlanivimab | soc | 0.05 | -0.72 | 0.87 | 4 | NA | NA | NA | NA | NA | NA | 0.05 | -0.72 | 0.87 | 3 | Imprecision |
| baricitinib + remdesivir | soc | NA | NA | NA | NA | NA | -0.44 | -0.87 | -0.02 | 2 | NA | -0.44 | -0.87 | -0.02 | 2 | NA |
| bromhexine | soc | -0.96 | -2.09 | 0.02 | 2 | RoB;Inconsistency; | NA | NA | NA | NA | NA | -0.96 | -2.09 | 0.02 | 1 | Severe Imprecision |
| budesonide | soc | 0.19 | -0.41 | 0.80 | 3 | RoB; | NA | NA | NA | NA | NA | 0.19 | -0.41 | 0.80 | 2 | Imprecision |
| camostat mesilate | soc | 0.05 | -1.35 | 1.69 | 4 | NA | NA | NA | NA | NA | NA | 0.05 | -1.35 | 1.69 | 2 | Severe Imprecision |
| canakinumab | soc | -0.48 | -1.11 | 0.11 | 4 | NA | NA | NA | NA | NA | NA | -0.48 | -1.11 | 0.11 | 3 | Imprecision |
| chloroquine | soc | 0.44 | -0.33 | 1.34 | 3 | RoB; | 0.25 | -0.41 | 0.92 | 3 | NA | 0.23 | -0.31 | 0.79 | 2 | Imprecision |
| colchicine | soc | -0.86 | -1.61 | -0.18 | 3 | RoB; | NA | NA | NA | NA | NA | -0.86 | -1.61 | -0.18 | 3 | NA |
| convalescent plasma | soc | -0.03 | -0.14 | 0.08 | 3 | RoB; | NA | NA | NA | NA | NA | -0.03 | -0.14 | 0.08 | 2 | Imprecision |
| dexamethasone | soc | -0.42 | -0.61 | -0.23 | 3 | RoB; | 0.56 | -0.28 | 1.53 | 3 | NA | -0.41 | -0.59 | -0.23 | 3 | NA |
| doxycycline | soc | -0.64 | -1.56 | 0.07 | 3 | RoB; | NA | NA | NA | NA | NA | -0.64 | -1.56 | 0.07 | 1 | Severe Imprecision |
| favipiravir | soc | -0.03 | -0.76 | 0.68 | 3 | RoB; | 0.23 | -0.31 | 0.79 | 3 | NA | 0.14 | -0.33 | 0.62 | 2 | Imprecision |
| hydroxychloroquine | soc | -0.02 | -0.17 | 0.11 | 3 | RoB; | 0.20 | -0.40 | 0.83 | 3 | NA | -0.03 | -0.16 | 0.11 | 2 | Imprecision |
| hydroxychloroquine + azithromycin | soc | 0.50 | -0.12 | 1.11 | 3 | RoB; | NA | NA | NA | NA | NA | 0.50 | -0.12 | 1.11 | 2 | Imprecision |
| hydroxychloroquine + favipiravir | soc | 0.10 | -0.60 | 0.79 | 3 | RoB; | NA | NA | NA | NA | NA | 0.10 | -0.60 | 0.79 | 2 | Imprecision |
| imatinib | soc | 0.06 | -0.45 | 0.58 | 4 | NA | NA | NA | NA | NA | NA | 0.06 | -0.45 | 0.58 | 3 | Imprecision |
| inm005 | soc | -0.25 | -0.93 | 0.40 | 4 | NA | NA | NA | NA | NA | NA | -0.25 | -0.93 | 0.40 | 3 | Imprecision |
| interferon beta | soc | -0.03 | -0.19 | 0.13 | 3 | RoB; | NA | NA | NA | NA | NA | -0.03 | -0.19 | 0.13 | 2 | Imprecision |
| intravenous immunoglobulin | soc | -0.35 | -0.89 | 0.19 | 3 | RoB; | NA | NA | NA | NA | NA | -0.35 | -0.89 | 0.19 | 2 | Imprecision |
| ivermectin | soc | -0.22 | -1.00 | 0.54 | 4 | NA | 0.02 | -0.77 | 0.80 | 2 | Intransitivity | -0.09 | -0.72 | 0.54 | 3 | Imprecision |
| lopinavir/ritonavir | soc | -0.04 | -0.18 | 0.10 | 3 | RoB; | -0.46 | -1.38 | 0.34 | 3 | NA | -0.05 | -0.19 | 0.08 | 2 | Imprecision |
| methylprednisolone | soc | 0.11 | -0.43 | 0.67 | 3 | RoB; | -0.90 | -1.70 | -0.23 | 3 | NA | -0.25 | -0.73 | 0.21 | 2 | Imprecision |
| recombinant human gcsf | soc | -1.37 | -2.03 | -0.73 | 3 | RoB; | NA | NA | NA | NA | NA | -1.37 | -2.03 | -0.73 | 3 | NA |
| remdesivir | soc | 0.04 | -0.09 | 0.17 | 2 | RoB;Inconsistency; | NA | NA | NA | NA | NA | 0.04 | -0.09 | 0.17 | 1 | Imprecision |
| sarilumab | soc | -0.40 | -0.82 | 0.01 | 2 | RoB;Inconsistency; | NA | NA | NA | NA | NA | -0.40 | -0.82 | 0.01 | 1 | Imprecision |
| sofosbuvir + daclatasvir | soc | -0.34 | -1.28 | 0.40 | 3 | RoB; | NA | NA | NA | NA | NA | -0.34 | -1.28 | 0.40 | 2 | Imprecision |
| sulodexide | soc | -0.83 | -2.51 | 0.57 | 3 | RoB; | NA | NA | NA | NA | NA | -0.83 | -2.51 | 0.57 | 1 | Severe Imprecision |
| tocilizumab | soc | -0.30 | -0.44 | -0.16 | 3 | RoB; | -0.31 | -1.56 | 0.81 | 3 | NA | -0.29 | -0.44 | -0.15 | 3 | NA |
| tofacitinib | soc | -0.44 | -1.11 | 0.20 | 4 | NA | NA | NA | NA | NA | NA | -0.44 | -1.11 | 0.20 | 3 | Imprecision |
| vitamin d3 | soc | -0.74 | -1.65 | 0.10 | 4 | NA | NA | NA | NA | NA | NA | -0.74 | -1.65 | 0.10 | 2 | Severe Imprecision |
| ammonium chloride | aceis/arbs | NA | NA | NA | NA | NA | -0.12 | -0.93 | 0.65 | 3 | NA | -0.12 | -0.93 | 0.65 | 2 | Imprecision |
| azithromycin | aceis/arbs | NA | NA | NA | NA | NA | 0.05 | -0.38 | 0.49 | 3 | NA | 0.05 | -0.38 | 0.49 | 2 | Imprecision |
| bamlanivimab | aceis/arbs | NA | NA | NA | NA | NA | 0.18 | -0.70 | 1.09 | 3 | NA | 0.18 | -0.70 | 1.09 | 2 | Imprecision |
| baricitinib + remdesivir | aceis/arbs | NA | NA | NA | NA | NA | -0.31 | -0.90 | 0.28 | 2 | NA | -0.31 | -0.90 | 0.28 | 1 | Imprecision |
| bromhexine | aceis/arbs | NA | NA | NA | NA | NA | -0.84 | -2.02 | 0.23 | 2 | NA | -0.84 | -2.02 | 0.23 | 1 | Severe Imprecision |
| budesonide | aceis/arbs | NA | NA | NA | NA | NA | 0.31 | -0.41 | 1.06 | 3 | NA | 0.31 | -0.41 | 1.06 | 2 | Imprecision |
| camostat mesilate | aceis/arbs | NA | NA | NA | NA | NA | 0.18 | -1.28 | 1.87 | 3 | NA | 0.18 | -1.28 | 1.87 | 1 | Severe Imprecision |
| canakinumab | aceis/arbs | NA | NA | NA | NA | NA | -0.35 | -1.10 | 0.37 | 2 | Intransitivity | -0.35 | -1.10 | 0.37 | 1 | Imprecision |
| chloroquine | aceis/arbs | NA | NA | NA | NA | NA | 0.36 | -0.32 | 1.05 | 3 | NA | 0.36 | -0.32 | 1.05 | 2 | Imprecision |
| colchicine | aceis/arbs | NA | NA | NA | NA | NA | -0.73 | -1.58 | 0.06 | 3 | NA | -0.73 | -1.58 | 0.06 | 1 | Severe Imprecision |
| convalescent plasma | aceis/arbs | NA | NA | NA | NA | NA | 0.10 | -0.32 | 0.52 | 2 | Intransitivity | 0.10 | -0.32 | 0.52 | 1 | Imprecision |
| dexamethasone | aceis/arbs | NA | NA | NA | NA | NA | -0.28 | -0.73 | 0.17 | 3 | NA | -0.28 | -0.73 | 0.17 | 2 | Imprecision |
| doxycycline | aceis/arbs | NA | NA | NA | NA | NA | -0.52 | -1.52 | 0.32 | 3 | NA | -0.52 | -1.52 | 0.32 | 1 | Severe Imprecision |
| favipiravir | aceis/arbs | NA | NA | NA | NA | NA | 0.27 | -0.35 | 0.90 | 3 | NA | 0.27 | -0.35 | 0.90 | 2 | Imprecision |
| hydroxychloroquine | aceis/arbs | NA | NA | NA | NA | NA | 0.10 | -0.33 | 0.54 | 3 | NA | 0.10 | -0.33 | 0.54 | 2 | Imprecision |
| hydroxychloroquine + azithromycin | aceis/arbs | NA | NA | NA | NA | NA | 0.63 | -0.11 | 1.36 | 3 | NA | 0.63 | -0.11 | 1.36 | 2 | Imprecision |
| hydroxychloroquine + favipiravir | aceis/arbs | NA | NA | NA | NA | NA | 0.22 | -0.58 | 1.03 | 3 | NA | 0.22 | -0.58 | 1.03 | 2 | Imprecision |
| imatinib | aceis/arbs | NA | NA | NA | NA | NA | 0.19 | -0.47 | 0.85 | 2 | Intransitivity | 0.19 | -0.47 | 0.85 | 1 | Imprecision |
| inm005 | aceis/arbs | NA | NA | NA | NA | NA | -0.12 | -0.91 | 0.65 | 3 | NA | -0.12 | -0.91 | 0.65 | 2 | Imprecision |
| interferon beta | aceis/arbs | NA | NA | NA | NA | NA | 0.10 | -0.34 | 0.54 | 2 | Intransitivity | 0.10 | -0.34 | 0.54 | 1 | Imprecision |
| intravenous immunoglobulin | aceis/arbs | NA | NA | NA | NA | NA | -0.22 | -0.89 | 0.46 | 3 | NA | -0.22 | -0.89 | 0.46 | 2 | Imprecision |
| ivermectin | aceis/arbs | NA | NA | NA | NA | NA | 0.04 | -0.71 | 0.79 | 3 | NA | 0.04 | -0.71 | 0.79 | 2 | Imprecision |
| lopinavir/ritonavir | aceis/arbs | NA | NA | NA | NA | NA | 0.07 | -0.36 | 0.51 | 3 | NA | 0.07 | -0.36 | 0.51 | 2 | Imprecision |
| methylprednisolone | aceis/arbs | NA | NA | NA | NA | NA | -0.13 | -0.75 | 0.50 | 3 | NA | -0.13 | -0.75 | 0.50 | 2 | Imprecision |
| recombinant human gcsf | aceis/arbs | NA | NA | NA | NA | NA | -1.24 | -2.02 | -0.48 | 3 | NA | -1.24 | -2.02 | -0.48 | 3 | NA |
| remdesivir | aceis/arbs | NA | NA | NA | NA | NA | 0.17 | -0.25 | 0.60 | 2 | NA | 0.17 | -0.25 | 0.60 | 1 | Imprecision |
| sarilumab | aceis/arbs | NA | NA | NA | NA | NA | -0.28 | -0.85 | 0.31 | 1 | Intransitivity | -0.28 | -0.85 | 0.31 | 1 | Imprecision |
| sofosbuvir + daclatasvir | aceis/arbs | NA | NA | NA | NA | NA | -0.22 | -1.23 | 0.64 | 3 | NA | -0.22 | -1.23 | 0.64 | 2 | Imprecision |
| sulodexide | aceis/arbs | NA | NA | NA | NA | NA | -0.71 | -2.43 | 0.76 | 3 | NA | -0.71 | -2.43 | 0.76 | 1 | Severe Imprecision |
| tocilizumab | aceis/arbs | NA | NA | NA | NA | NA | -0.17 | -0.60 | 0.27 | 3 | NA | -0.17 | -0.60 | 0.27 | 2 | Imprecision |
| tofacitinib | aceis/arbs | NA | NA | NA | NA | NA | -0.31 | -1.09 | 0.45 | 3 | NA | -0.31 | -1.09 | 0.45 | 2 | Imprecision |
| vitamin d3 | aceis/arbs | NA | NA | NA | NA | NA | -0.62 | -1.61 | 0.32 | 2 | Intransitivity | -0.62 | -1.61 | 0.32 | 1 | Severe Imprecision |
| azithromycin | ammonium chloride | NA | NA | NA | NA | NA | 0.18 | -0.49 | 0.89 | 3 | NA | 0.18 | -0.49 | 0.89 | 2 | Imprecision |
| bamlanivimab | ammonium chloride | NA | NA | NA | NA | NA | 0.30 | -0.71 | 1.39 | 4 | NA | 0.30 | -0.71 | 1.39 | 3 | Imprecision |
| baricitinib + remdesivir | ammonium chloride | NA | NA | NA | NA | NA | -0.19 | -0.97 | 0.62 | 2 | NA | -0.19 | -0.97 | 0.62 | 1 | Imprecision |
| bromhexine | ammonium chloride | NA | NA | NA | NA | NA | -0.71 | -2.01 | 0.49 | 2 | NA | -0.71 | -2.01 | 0.49 | 1 | Severe Imprecision |
| budesonide | ammonium chloride | NA | NA | NA | NA | NA | 0.43 | -0.44 | 1.38 | 3 | NA | 0.43 | -0.44 | 1.38 | 2 | Imprecision |
| camostat mesilate | ammonium chloride | NA | NA | NA | NA | NA | 0.31 | -1.24 | 2.07 | 4 | NA | 0.31 | -1.24 | 2.07 | 2 | Severe Imprecision |
| canakinumab | ammonium chloride | NA | NA | NA | NA | NA | -0.23 | -1.13 | 0.68 | 4 | NA | -0.23 | -1.13 | 0.68 | 3 | Imprecision |
| chloroquine | ammonium chloride | NA | NA | NA | NA | NA | 0.48 | -0.37 | 1.39 | 3 | NA | 0.48 | -0.37 | 1.39 | 2 | Imprecision |
| colchicine | ammonium chloride | NA | NA | NA | NA | NA | -0.61 | -1.60 | 0.36 | 3 | NA | -0.61 | -1.60 | 0.36 | 1 | Severe Imprecision |
| convalescent plasma | ammonium chloride | NA | NA | NA | NA | NA | 0.22 | -0.44 | 0.92 | 3 | NA | 0.22 | -0.44 | 0.92 | 2 | Imprecision |
| dexamethasone | ammonium chloride | NA | NA | NA | NA | NA | -0.16 | -0.84 | 0.56 | 3 | NA | -0.16 | -0.84 | 0.56 | 2 | Imprecision |
| doxycycline | ammonium chloride | NA | NA | NA | NA | NA | -0.39 | -1.49 | 0.57 | 3 | NA | -0.39 | -1.49 | 0.57 | 2 | Imprecision |
| favipiravir | ammonium chloride | NA | NA | NA | NA | NA | 0.39 | -0.41 | 1.25 | 3 | NA | 0.39 | -0.41 | 1.25 | 2 | Imprecision |
| hydroxychloroquine | ammonium chloride | NA | NA | NA | NA | NA | 0.22 | -0.44 | 0.93 | 3 | NA | 0.22 | -0.44 | 0.93 | 2 | Imprecision |
| hydroxychloroquine + azithromycin | ammonium chloride | NA | NA | NA | NA | NA | 0.75 | -0.15 | 1.67 | 3 | NA | 0.75 | -0.15 | 1.67 | 1 | Severe Imprecision |
| hydroxychloroquine + favipiravir | ammonium chloride | NA | NA | NA | NA | NA | 0.35 | -0.61 | 1.32 | 3 | NA | 0.35 | -0.61 | 1.32 | 2 | Imprecision |
| imatinib | ammonium chloride | NA | NA | NA | NA | NA | 0.31 | -0.52 | 1.18 | 4 | NA | 0.31 | -0.52 | 1.18 | 3 | Imprecision |
| inm005 | ammonium chloride | NA | NA | NA | NA | NA | 0.01 | -0.94 | 0.94 | 4 | NA | 0.01 | -0.94 | 0.94 | 3 | Imprecision |
| interferon beta | ammonium chloride | NA | NA | NA | NA | NA | 0.22 | -0.46 | 0.94 | 3 | NA | 0.22 | -0.46 | 0.94 | 2 | Imprecision |
| intravenous immunoglobulin | ammonium chloride | NA | NA | NA | NA | NA | -0.09 | -0.94 | 0.78 | 3 | NA | -0.09 | -0.94 | 0.78 | 2 | Imprecision |
| ivermectin | ammonium chloride | NA | NA | NA | NA | NA | 0.17 | -0.75 | 1.10 | 4 | NA | 0.17 | -0.75 | 1.10 | 3 | Imprecision |
| lopinavir/ritonavir | ammonium chloride | NA | NA | NA | NA | NA | 0.20 | -0.47 | 0.90 | 3 | NA | 0.20 | -0.47 | 0.90 | 2 | Imprecision |
| methylprednisolone | ammonium chloride | NA | NA | NA | NA | NA | 0.00 | -0.81 | 0.83 | 3 | NA | 0.00 | -0.81 | 0.83 | 2 | Imprecision |
| recombinant human gcsf | ammonium chloride | NA | NA | NA | NA | NA | -1.11 | -2.05 | -0.24 | 3 | NA | -1.11 | -2.05 | -0.24 | 3 | NA |
| remdesivir | ammonium chloride | NA | NA | NA | NA | NA | 0.29 | -0.37 | 1.00 | 2 | NA | 0.29 | -0.37 | 1.00 | 1 | Imprecision |
| sarilumab | ammonium chloride | NA | NA | NA | NA | NA | -0.15 | -0.93 | 0.66 | 2 | NA | -0.15 | -0.93 | 0.66 | 1 | Imprecision |
| sofosbuvir + daclatasvir | ammonium chloride | NA | NA | NA | NA | NA | -0.10 | -1.21 | 0.90 | 3 | NA | -0.10 | -1.21 | 0.90 | 2 | Imprecision |
| sulodexide | ammonium chloride | NA | NA | NA | NA | NA | -0.58 | -2.37 | 0.99 | 3 | NA | -0.58 | -2.37 | 0.99 | 1 | Severe Imprecision |
| tocilizumab | ammonium chloride | NA | NA | NA | NA | NA | -0.04 | -0.71 | 0.66 | 3 | NA | -0.04 | -0.71 | 0.66 | 2 | Imprecision |
| tofacitinib | ammonium chloride | NA | NA | NA | NA | NA | -0.19 | -1.12 | 0.76 | 4 | NA | -0.19 | -1.12 | 0.76 | 3 | Imprecision |
| vitamin d3 | ammonium chloride | NA | NA | NA | NA | NA | -0.49 | -1.61 | 0.59 | 4 | NA | -0.49 | -1.61 | 0.59 | 2 | Severe Imprecision |
| bamlanivimab | azithromycin | NA | NA | NA | NA | NA | 0.13 | -0.66 | 0.95 | 3 | NA | 0.13 | -0.66 | 0.95 | 2 | Imprecision |
| baricitinib + remdesivir | azithromycin | NA | NA | NA | NA | NA | -0.36 | -0.82 | 0.08 | 2 | NA | -0.36 | -0.82 | 0.08 | 1 | Imprecision |
| bromhexine | azithromycin | NA | NA | NA | NA | NA | -0.89 | -2.02 | 0.11 | 2 | NA | -0.89 | -2.02 | 0.11 | 1 | Severe Imprecision |
| budesonide | azithromycin | 0.26 | -0.35 | 0.88 | 3 | RoB; | NA | NA | NA | NA | NA | 0.26 | -0.35 | 0.88 | 2 | Imprecision |
| camostat mesilate | azithromycin | NA | NA | NA | NA | NA | 0.13 | -1.28 | 1.77 | 3 | NA | 0.13 | -1.28 | 1.77 | 1 | Severe Imprecision |
| canakinumab | azithromycin | NA | NA | NA | NA | NA | -0.40 | -1.06 | 0.21 | 3 | NA | -0.40 | -1.06 | 0.21 | 2 | Imprecision |
| chloroquine | azithromycin | NA | NA | NA | NA | NA | 0.31 | -0.25 | 0.88 | 3 | NA | 0.31 | -0.25 | 0.88 | 2 | Imprecision |
| colchicine | azithromycin | NA | NA | NA | NA | NA | -0.78 | -1.55 | -0.10 | 3 | NA | -0.78 | -1.55 | -0.10 | 3 | NA |
| convalescent plasma | azithromycin | NA | NA | NA | NA | NA | 0.04 | -0.14 | 0.23 | 3 | NA | 0.04 | -0.14 | 0.23 | 2 | Imprecision |
| dexamethasone | azithromycin | -0.41 | -0.65 | -0.18 | 3 | RoB; | -0.02 | -0.50 | 0.46 | 3 | NA | -0.33 | -0.55 | -0.12 | 3 | NA |
| doxycycline | azithromycin | -0.56 | -1.49 | 0.14 | 3 | RoB; | NA | NA | NA | NA | NA | -0.56 | -1.49 | 0.14 | 2 | Imprecision |
| favipiravir | azithromycin | NA | NA | NA | NA | NA | 0.21 | -0.27 | 0.71 | 3 | NA | 0.21 | -0.27 | 0.71 | 2 | Imprecision |
| hydroxychloroquine | azithromycin | 0.15 | -0.07 | 0.37 | 3 | RoB; | -0.21 | -0.60 | 0.18 | 3 | NA | 0.05 | -0.14 | 0.23 | 2 | Imprecision |
| hydroxychloroquine + azithromycin | azithromycin | NA | NA | NA | NA | NA | 0.57 | -0.06 | 1.20 | 3 | NA | 0.57 | -0.06 | 1.20 | 2 | Imprecision |
| hydroxychloroquine + favipiravir | azithromycin | NA | NA | NA | NA | NA | 0.17 | -0.54 | 0.87 | 3 | NA | 0.17 | -0.54 | 0.87 | 2 | Imprecision |
| imatinib | azithromycin | NA | NA | NA | NA | NA | 0.13 | -0.40 | 0.67 | 3 | NA | 0.13 | -0.40 | 0.67 | 2 | Imprecision |
| inm005 | azithromycin | NA | NA | NA | NA | NA | -0.17 | -0.88 | 0.49 | 3 | NA | -0.17 | -0.88 | 0.49 | 2 | Imprecision |
| interferon beta | azithromycin | NA | NA | NA | NA | NA | 0.05 | -0.17 | 0.26 | 3 | NA | 0.05 | -0.17 | 0.26 | 2 | Imprecision |
| intravenous immunoglobulin | azithromycin | NA | NA | NA | NA | NA | -0.27 | -0.83 | 0.29 | 3 | NA | -0.27 | -0.83 | 0.29 | 2 | Imprecision |
| ivermectin | azithromycin | NA | NA | NA | NA | NA | -0.01 | -0.66 | 0.63 | 3 | NA | -0.01 | -0.66 | 0.63 | 2 | Imprecision |
| lopinavir/ritonavir | azithromycin | 0.13 | -0.09 | 0.34 | 3 | RoB; | -0.21 | -0.59 | 0.16 | 3 | NA | 0.02 | -0.16 | 0.20 | 2 | Imprecision |
| methylprednisolone | azithromycin | NA | NA | NA | NA | NA | -0.18 | -0.67 | 0.30 | 3 | NA | -0.18 | -0.67 | 0.30 | 2 | Imprecision |
| recombinant human gcsf | azithromycin | NA | NA | NA | NA | NA | -1.30 | -1.97 | -0.64 | 3 | NA | -1.30 | -1.97 | -0.64 | 3 | NA |
| remdesivir | azithromycin | NA | NA | NA | NA | NA | 0.12 | -0.07 | 0.31 | 2 | NA | 0.12 | -0.07 | 0.31 | 1 | Imprecision |
| sarilumab | azithromycin | NA | NA | NA | NA | NA | -0.33 | -0.77 | 0.11 | 2 | NA | -0.33 | -0.77 | 0.11 | 1 | Imprecision |
| sofosbuvir + daclatasvir | azithromycin | NA | NA | NA | NA | NA | -0.27 | -1.21 | 0.49 | 3 | NA | -0.27 | -1.21 | 0.49 | 2 | Imprecision |
| sulodexide | azithromycin | NA | NA | NA | NA | NA | -0.76 | -2.44 | 0.65 | 3 | NA | -0.76 | -2.44 | 0.65 | 1 | Severe Imprecision |
| tocilizumab | azithromycin | NA | NA | NA | NA | NA | -0.22 | -0.42 | -0.01 | 3 | NA | -0.22 | -0.42 | -0.01 | 2 | Imprecision |
| tofacitinib | azithromycin | NA | NA | NA | NA | NA | -0.36 | -1.06 | 0.29 | 3 | NA | -0.36 | -1.06 | 0.29 | 2 | Imprecision |
| vitamin d3 | azithromycin | NA | NA | NA | NA | NA | -0.67 | -1.59 | 0.19 | 3 | NA | -0.67 | -1.59 | 0.19 | 1 | Severe Imprecision |
| baricitinib + remdesivir | bamlanivimab | NA | NA | NA | NA | NA | -0.49 | -1.40 | 0.39 | 2 | NA | -0.49 | -1.40 | 0.39 | 1 | Imprecision |
| bromhexine | bamlanivimab | NA | NA | NA | NA | NA | -1.02 | -2.39 | 0.23 | 2 | NA | -1.02 | -2.39 | 0.23 | 1 | Severe Imprecision |
| budesonide | bamlanivimab | NA | NA | NA | NA | NA | 0.13 | -0.85 | 1.12 | 3 | NA | 0.13 | -0.85 | 1.12 | 2 | Imprecision |
| camostat mesilate | bamlanivimab | NA | NA | NA | NA | NA | 0.00 | -1.63 | 1.80 | 4 | NA | 0.00 | -1.63 | 1.80 | 2 | Severe Imprecision |
| canakinumab | bamlanivimab | NA | NA | NA | NA | NA | -0.53 | -1.57 | 0.44 | 3 | Intransitivity | -0.53 | -1.57 | 0.44 | 1 | Severe Imprecision |
| chloroquine | bamlanivimab | NA | NA | NA | NA | NA | 0.18 | -0.78 | 1.14 | 3 | NA | 0.18 | -0.78 | 1.14 | 2 | Imprecision |
| colchicine | bamlanivimab | NA | NA | NA | NA | NA | -0.92 | -2.01 | 0.12 | 3 | NA | -0.92 | -2.01 | 0.12 | 1 | Severe Imprecision |
| convalescent plasma | bamlanivimab | NA | NA | NA | NA | NA | -0.08 | -0.90 | 0.70 | 2 | Intransitivity | -0.08 | -0.90 | 0.70 | 1 | Imprecision |
| dexamethasone | bamlanivimab | NA | NA | NA | NA | NA | -0.46 | -1.30 | 0.34 | 3 | NA | -0.46 | -1.30 | 0.34 | 2 | Imprecision |
| doxycycline | bamlanivimab | NA | NA | NA | NA | NA | -0.70 | -1.95 | 0.35 | 3 | NA | -0.70 | -1.95 | 0.35 | 1 | Severe Imprecision |
| favipiravir | bamlanivimab | NA | NA | NA | NA | NA | 0.08 | -0.84 | 1.00 | 3 | NA | 0.08 | -0.84 | 1.00 | 2 | Imprecision |
| hydroxychloroquine | bamlanivimab | NA | NA | NA | NA | NA | -0.08 | -0.90 | 0.71 | 3 | NA | -0.08 | -0.90 | 0.71 | 2 | Imprecision |
| hydroxychloroquine + azithromycin | bamlanivimab | NA | NA | NA | NA | NA | 0.44 | -0.57 | 1.43 | 3 | NA | 0.44 | -0.57 | 1.43 | 2 | Imprecision |
| hydroxychloroquine + favipiravir | bamlanivimab | NA | NA | NA | NA | NA | 0.04 | -1.02 | 1.08 | 3 | NA | 0.04 | -1.02 | 1.08 | 2 | Imprecision |
| imatinib | bamlanivimab | NA | NA | NA | NA | NA | 0.01 | -0.85 | 0.84 | 3 | Intransitivity | 0.01 | -0.85 | 0.84 | 2 | Imprecision |
| inm005 | bamlanivimab | NA | NA | NA | NA | NA | -0.29 | -1.27 | 0.56 | 4 | NA | -0.29 | -1.27 | 0.56 | 3 | Imprecision |
| interferon beta | bamlanivimab | NA | NA | NA | NA | NA | -0.08 | -0.90 | 0.72 | 2 | Intransitivity | -0.08 | -0.90 | 0.72 | 1 | Imprecision |
| intravenous immunoglobulin | bamlanivimab | NA | NA | NA | NA | NA | -0.40 | -1.37 | 0.54 | 3 | NA | -0.40 | -1.37 | 0.54 | 2 | Imprecision |
| ivermectin | bamlanivimab | NA | NA | NA | NA | NA | -0.14 | -1.17 | 0.86 | 4 | NA | -0.14 | -1.17 | 0.86 | 3 | Imprecision |
| lopinavir/ritonavir | bamlanivimab | NA | NA | NA | NA | NA | -0.11 | -0.93 | 0.68 | 3 | NA | -0.11 | -0.93 | 0.68 | 2 | Imprecision |
| methylprednisolone | bamlanivimab | NA | NA | NA | NA | NA | -0.31 | -1.25 | 0.60 | 3 | NA | -0.31 | -1.25 | 0.60 | 2 | Imprecision |
| recombinant human gcsf | bamlanivimab | NA | NA | NA | NA | NA | -1.41 | -2.52 | -0.43 | 3 | NA | -1.41 | -2.52 | -0.43 | 3 | NA |
| remdesivir | bamlanivimab | NA | NA | NA | NA | NA | -0.01 | -0.83 | 0.78 | 2 | NA | -0.01 | -0.83 | 0.78 | 1 | Imprecision |
| sarilumab | bamlanivimab | NA | NA | NA | NA | NA | -0.46 | -1.37 | 0.43 | 1 | Intransitivity | -0.46 | -1.37 | 0.43 | 1 | Imprecision |
| sofosbuvir + daclatasvir | bamlanivimab | NA | NA | NA | NA | NA | -0.40 | -1.67 | 0.67 | 3 | NA | -0.40 | -1.67 | 0.67 | 1 | Severe Imprecision |
| sulodexide | bamlanivimab | NA | NA | NA | NA | NA | -0.90 | -2.75 | 0.73 | 3 | NA | -0.90 | -2.75 | 0.73 | 1 | Severe Imprecision |
| tocilizumab | bamlanivimab | NA | NA | NA | NA | NA | -0.35 | -1.17 | 0.44 | 3 | NA | -0.35 | -1.17 | 0.44 | 2 | Imprecision |
| tofacitinib | bamlanivimab | NA | NA | NA | NA | NA | -0.49 | -1.55 | 0.50 | 4 | NA | -0.49 | -1.55 | 0.50 | 2 | Severe Imprecision |
| vitamin d3 | bamlanivimab | NA | NA | NA | NA | NA | -0.80 | -2.00 | 0.35 | 3 | Intransitivity | -0.80 | -2.00 | 0.35 | 1 | Severe Imprecision |
| bromhexine | baricitinib + remdesivir | NA | NA | NA | NA | NA | -0.53 | -1.73 | 0.55 | 2 | NA | -0.53 | -1.73 | 0.55 | 1 | Severe Imprecision |
| budesonide | baricitinib + remdesivir | NA | NA | NA | NA | NA | 0.63 | -0.11 | 1.37 | 2 | NA | 0.63 | -0.11 | 1.37 | 1 | Imprecision |
| camostat mesilate | baricitinib + remdesivir | NA | NA | NA | NA | NA | 0.50 | -0.97 | 2.18 | 2 | NA | 0.50 | -0.97 | 2.18 | 1 | Severe Imprecision |
| canakinumab | baricitinib + remdesivir | NA | NA | NA | NA | NA | -0.04 | -0.80 | 0.69 | 2 | NA | -0.04 | -0.80 | 0.69 | 1 | Imprecision |
| chloroquine | baricitinib + remdesivir | NA | NA | NA | NA | NA | 0.67 | -0.02 | 1.37 | 2 | NA | 0.67 | -0.02 | 1.37 | 1 | Imprecision |
| colchicine | baricitinib + remdesivir | NA | NA | NA | NA | NA | -0.42 | -1.28 | 0.38 | 2 | NA | -0.42 | -1.28 | 0.38 | 1 | Imprecision |
| convalescent plasma | baricitinib + remdesivir | NA | NA | NA | NA | NA | 0.41 | -0.02 | 0.85 | 2 | NA | 0.41 | -0.02 | 0.85 | 1 | Imprecision |
| dexamethasone | baricitinib + remdesivir | NA | NA | NA | NA | NA | 0.03 | -0.43 | 0.49 | 2 | NA | 0.03 | -0.43 | 0.49 | 1 | Imprecision |
| doxycycline | baricitinib + remdesivir | NA | NA | NA | NA | NA | -0.21 | -1.21 | 0.64 | 2 | NA | -0.21 | -1.21 | 0.64 | 1 | Imprecision |
| favipiravir | baricitinib + remdesivir | NA | NA | NA | NA | NA | 0.58 | -0.04 | 1.22 | 2 | NA | 0.58 | -0.04 | 1.22 | 1 | Imprecision |
| hydroxychloroquine | baricitinib + remdesivir | NA | NA | NA | NA | NA | 0.41 | -0.02 | 0.86 | 3 | NA | 0.41 | -0.02 | 0.86 | 2 | Imprecision |
| hydroxychloroquine + azithromycin | baricitinib + remdesivir | NA | NA | NA | NA | NA | 0.94 | 0.19 | 1.68 | 2 | NA | 0.94 | 0.19 | 1.68 | 2 | NA |
| hydroxychloroquine + favipiravir | baricitinib + remdesivir | NA | NA | NA | NA | NA | 0.54 | -0.27 | 1.35 | 2 | NA | 0.54 | -0.27 | 1.35 | 1 | Imprecision |
| imatinib | baricitinib + remdesivir | NA | NA | NA | NA | NA | 0.50 | -0.17 | 1.17 | 2 | NA | 0.50 | -0.17 | 1.17 | 1 | Imprecision |
| inm005 | baricitinib + remdesivir | NA | NA | NA | NA | NA | 0.19 | -0.61 | 0.96 | 2 | NA | 0.19 | -0.61 | 0.96 | 1 | Imprecision |
| interferon beta | baricitinib + remdesivir | NA | NA | NA | NA | NA | 0.41 | -0.03 | 0.85 | 3 | NA | 0.41 | -0.03 | 0.85 | 2 | Imprecision |
| intravenous immunoglobulin | baricitinib + remdesivir | NA | NA | NA | NA | NA | 0.09 | -0.59 | 0.78 | 2 | NA | 0.09 | -0.59 | 0.78 | 1 | Imprecision |
| ivermectin | baricitinib + remdesivir | NA | NA | NA | NA | NA | 0.35 | -0.41 | 1.10 | 2 | NA | 0.35 | -0.41 | 1.10 | 1 | Imprecision |
| lopinavir/ritonavir | baricitinib + remdesivir | NA | NA | NA | NA | NA | 0.38 | -0.05 | 0.83 | 3 | NA | 0.38 | -0.05 | 0.83 | 2 | Imprecision |
| methylprednisolone | baricitinib + remdesivir | NA | NA | NA | NA | NA | 0.18 | -0.45 | 0.82 | 2 | NA | 0.18 | -0.45 | 0.82 | 1 | Imprecision |
| recombinant human gcsf | baricitinib + remdesivir | NA | NA | NA | NA | NA | -0.93 | -1.71 | -0.16 | 2 | NA | -0.93 | -1.71 | -0.16 | 2 | NA |
| remdesivir | baricitinib + remdesivir | 0.48 | 0.09 | 0.89 | 4 | NA | NA | NA | NA | NA | NA | 0.48 | 0.09 | 0.89 | 4 | NA |
| sarilumab | baricitinib + remdesivir | NA | NA | NA | NA | NA | 0.03 | -0.56 | 0.63 | 2 | NA | 0.03 | -0.56 | 0.63 | 1 | Imprecision |
| sofosbuvir + daclatasvir | baricitinib + remdesivir | NA | NA | NA | NA | NA | 0.09 | -0.93 | 0.95 | 2 | NA | 0.09 | -0.93 | 0.95 | 1 | Imprecision |
| sulodexide | baricitinib + remdesivir | NA | NA | NA | NA | NA | -0.39 | -2.11 | 1.08 | 2 | NA | -0.39 | -2.11 | 1.08 | 1 | Severe Imprecision |
| tocilizumab | baricitinib + remdesivir | NA | NA | NA | NA | NA | 0.14 | -0.30 | 0.59 | 2 | NA | 0.14 | -0.30 | 0.59 | 1 | Imprecision |
| tofacitinib | baricitinib + remdesivir | NA | NA | NA | NA | NA | 0.00 | -0.79 | 0.77 | 2 | NA | 0.00 | -0.79 | 0.77 | 1 | Imprecision |
| vitamin d3 | baricitinib + remdesivir | NA | NA | NA | NA | NA | -0.31 | -1.30 | 0.64 | 2 | NA | -0.31 | -1.30 | 0.64 | 1 | Imprecision |
| budesonide | bromhexine | NA | NA | NA | NA | NA | 1.15 | -0.01 | 2.42 | 2 | NA | 1.15 | -0.01 | 2.42 | 1 | Severe Imprecision |
| camostat mesilate | bromhexine | NA | NA | NA | NA | NA | 1.04 | -0.71 | 2.97 | 2 | NA | 1.04 | -0.71 | 2.97 | 1 | Severe Imprecision |
| canakinumab | bromhexine | NA | NA | NA | NA | NA | 0.49 | -0.69 | 1.75 | 2 | NA | 0.49 | -0.69 | 1.75 | 1 | Severe Imprecision |
| chloroquine | bromhexine | NA | NA | NA | NA | NA | 1.20 | 0.06 | 2.44 | 2 | NA | 1.20 | 0.06 | 2.44 | 2 | NA |
| colchicine | bromhexine | NA | NA | NA | NA | NA | 0.11 | -1.14 | 1.41 | 2 | NA | 0.11 | -1.14 | 1.41 | 1 | Imprecision |
| convalescent plasma | bromhexine | NA | NA | NA | NA | NA | 0.93 | -0.06 | 2.06 | 2 | NA | 0.93 | -0.06 | 2.06 | 1 | Severe Imprecision |
| dexamethasone | bromhexine | NA | NA | NA | NA | NA | 0.55 | -0.45 | 1.69 | 2 | NA | 0.55 | -0.45 | 1.69 | 1 | Severe Imprecision |
| doxycycline | bromhexine | NA | NA | NA | NA | NA | 0.31 | -1.00 | 1.64 | 2 | NA | 0.31 | -1.00 | 1.64 | 1 | Severe Imprecision |
| favipiravir | bromhexine | NA | NA | NA | NA | NA | 1.11 | 0.01 | 2.32 | 2 | NA | 1.11 | 0.01 | 2.32 | 1 | Severe Imprecision |
| hydroxychloroquine | bromhexine | NA | NA | NA | NA | NA | 0.94 | -0.06 | 2.07 | 2 | NA | 0.94 | -0.06 | 2.07 | 1 | Severe Imprecision |
| hydroxychloroquine + azithromycin | bromhexine | NA | NA | NA | NA | NA | 1.46 | 0.30 | 2.73 | 2 | NA | 1.46 | 0.30 | 2.73 | 2 | NA |
| hydroxychloroquine + favipiravir | bromhexine | NA | NA | NA | NA | NA | 1.07 | -0.15 | 2.37 | 2 | NA | 1.07 | -0.15 | 2.37 | 1 | Severe Imprecision |
| imatinib | bromhexine | NA | NA | NA | NA | NA | 1.02 | -0.09 | 2.26 | 2 | NA | 1.02 | -0.09 | 2.26 | 1 | Severe Imprecision |
| inm005 | bromhexine | NA | NA | NA | NA | NA | 0.71 | -0.48 | 2.02 | 2 | NA | 0.71 | -0.48 | 2.02 | 1 | Severe Imprecision |
| interferon beta | bromhexine | NA | NA | NA | NA | NA | 0.94 | -0.07 | 2.07 | 2 | NA | 0.94 | -0.07 | 2.07 | 1 | Severe Imprecision |
| intravenous immunoglobulin | bromhexine | NA | NA | NA | NA | NA | 0.62 | -0.51 | 1.86 | 2 | NA | 0.62 | -0.51 | 1.86 | 1 | Severe Imprecision |
| ivermectin | bromhexine | NA | NA | NA | NA | NA | 0.88 | -0.30 | 2.15 | 2 | NA | 0.88 | -0.30 | 2.15 | 1 | Severe Imprecision |
| lopinavir/ritonavir | bromhexine | NA | NA | NA | NA | NA | 0.91 | -0.09 | 2.04 | 2 | NA | 0.91 | -0.09 | 2.04 | 1 | Severe Imprecision |
| methylprednisolone | bromhexine | NA | NA | NA | NA | NA | 0.71 | -0.38 | 1.92 | 2 | NA | 0.71 | -0.38 | 1.92 | 1 | Severe Imprecision |
| recombinant human gcsf | bromhexine | NA | NA | NA | NA | NA | -0.41 | -1.59 | 0.89 | 2 | NA | -0.41 | -1.59 | 0.89 | 1 | Severe Imprecision |
| remdesivir | bromhexine | NA | NA | NA | NA | NA | 1.01 | 0.01 | 2.14 | 2 | NA | 1.01 | 0.01 | 2.14 | 1 | Severe Imprecision |
| sarilumab | bromhexine | NA | NA | NA | NA | NA | 0.56 | -0.51 | 1.75 | 2 | NA | 0.56 | -0.51 | 1.75 | 1 | Severe Imprecision |
| sofosbuvir + daclatasvir | bromhexine | NA | NA | NA | NA | NA | 0.61 | -0.73 | 1.97 | 2 | NA | 0.61 | -0.73 | 1.97 | 1 | Severe Imprecision |
| sulodexide | bromhexine | NA | NA | NA | NA | NA | 0.13 | -1.82 | 1.93 | 2 | NA | 0.13 | -1.82 | 1.93 | 1 | Severe Imprecision |
| tocilizumab | bromhexine | NA | NA | NA | NA | NA | 0.67 | -0.33 | 1.80 | 2 | NA | 0.67 | -0.33 | 1.80 | 1 | Severe Imprecision |
| tofacitinib | bromhexine | NA | NA | NA | NA | NA | 0.53 | -0.67 | 1.81 | 2 | NA | 0.53 | -0.67 | 1.81 | 1 | Severe Imprecision |
| vitamin d3 | bromhexine | NA | NA | NA | NA | NA | 0.22 | -1.12 | 1.62 | 2 | NA | 0.22 | -1.12 | 1.62 | 1 | Severe Imprecision |
| camostat mesilate | budesonide | NA | NA | NA | NA | NA | -0.13 | -1.67 | 1.60 | 3 | NA | -0.13 | -1.67 | 1.60 | 1 | Severe Imprecision |
| canakinumab | budesonide | NA | NA | NA | NA | NA | -0.66 | -1.57 | 0.18 | 2 | Intransitivity | -0.66 | -1.57 | 0.18 | 1 | Severe Imprecision |
| chloroquine | budesonide | NA | NA | NA | NA | NA | 0.05 | -0.77 | 0.85 | 3 | NA | 0.05 | -0.77 | 0.85 | 2 | Imprecision |
| colchicine | budesonide | NA | NA | NA | NA | NA | -1.05 | -2.01 | -0.15 | 3 | NA | -1.05 | -2.01 | -0.15 | 3 | NA |
| convalescent plasma | budesonide | NA | NA | NA | NA | NA | -0.22 | -0.84 | 0.39 | 2 | Intransitivity | -0.22 | -0.84 | 0.39 | 1 | Imprecision |
| dexamethasone | budesonide | NA | NA | NA | NA | NA | -0.59 | -1.23 | 0.01 | 3 | NA | -0.59 | -1.23 | 0.01 | 2 | Imprecision |
| doxycycline | budesonide | -0.83 | -1.92 | 0.06 | 3 | RoB; | NA | NA | NA | NA | NA | -0.83 | -1.92 | 0.06 | 1 | Severe Imprecision |
| favipiravir | budesonide | NA | NA | NA | NA | NA | -0.04 | -0.81 | 0.70 | 3 | NA | -0.04 | -0.81 | 0.70 | 2 | Imprecision |
| hydroxychloroquine | budesonide | NA | NA | NA | NA | NA | -0.21 | -0.84 | 0.40 | 3 | NA | -0.21 | -0.84 | 0.40 | 2 | Imprecision |
| hydroxychloroquine + azithromycin | budesonide | NA | NA | NA | NA | NA | 0.31 | -0.56 | 1.17 | 3 | NA | 0.31 | -0.56 | 1.17 | 2 | Imprecision |
| hydroxychloroquine + favipiravir | budesonide | NA | NA | NA | NA | NA | -0.09 | -1.01 | 0.82 | 3 | NA | -0.09 | -1.01 | 0.82 | 2 | Imprecision |
| imatinib | budesonide | NA | NA | NA | NA | NA | -0.12 | -0.92 | 0.66 | 2 | Intransitivity | -0.12 | -0.92 | 0.66 | 1 | Imprecision |
| inm005 | budesonide | NA | NA | NA | NA | NA | -0.43 | -1.38 | 0.46 | 3 | NA | -0.43 | -1.38 | 0.46 | 2 | Imprecision |
| interferon beta | budesonide | NA | NA | NA | NA | NA | -0.21 | -0.84 | 0.40 | 2 | Intransitivity | -0.21 | -0.84 | 0.40 | 1 | Imprecision |
| intravenous immunoglobulin | budesonide | NA | NA | NA | NA | NA | -0.53 | -1.35 | 0.28 | 3 | NA | -0.53 | -1.35 | 0.28 | 2 | Imprecision |
| ivermectin | budesonide | NA | NA | NA | NA | NA | -0.27 | -1.15 | 0.60 | 3 | NA | -0.27 | -1.15 | 0.60 | 2 | Imprecision |
| lopinavir/ritonavir | budesonide | NA | NA | NA | NA | NA | -0.24 | -0.87 | 0.37 | 3 | NA | -0.24 | -0.87 | 0.37 | 2 | Imprecision |
| methylprednisolone | budesonide | NA | NA | NA | NA | NA | -0.43 | -1.18 | 0.25 | 3 | NA | -0.43 | -1.18 | 0.25 | 2 | Imprecision |
| recombinant human gcsf | budesonide | NA | NA | NA | NA | NA | -1.56 | -2.52 | -0.61 | 3 | NA | -1.56 | -2.52 | -0.61 | 3 | NA |
| remdesivir | budesonide | NA | NA | NA | NA | NA | -0.14 | -0.77 | 0.47 | 2 | NA | -0.14 | -0.77 | 0.47 | 1 | Imprecision |
| sarilumab | budesonide | NA | NA | NA | NA | NA | -0.59 | -1.34 | 0.14 | 1 | Intransitivity | -0.59 | -1.34 | 0.14 | 1 | Imprecision |
| sofosbuvir + daclatasvir | budesonide | NA | NA | NA | NA | NA | -0.53 | -1.71 | 0.44 | 3 | NA | -0.53 | -1.71 | 0.44 | 1 | Severe Imprecision |
| sulodexide | budesonide | NA | NA | NA | NA | NA | -1.02 | -2.80 | 0.53 | 3 | NA | -1.02 | -2.80 | 0.53 | 1 | Severe Imprecision |
| tocilizumab | budesonide | NA | NA | NA | NA | NA | -0.48 | -1.11 | 0.14 | 3 | NA | -0.48 | -1.11 | 0.14 | 2 | Imprecision |
| tofacitinib | budesonide | NA | NA | NA | NA | NA | -0.62 | -1.56 | 0.24 | 3 | NA | -0.62 | -1.56 | 0.24 | 1 | Severe Imprecision |
| vitamin d3 | budesonide | NA | NA | NA | NA | NA | -0.93 | -2.01 | 0.10 | 2 | Intransitivity | -0.93 | -2.01 | 0.10 | 1 | Severe Imprecision |
| canakinumab | camostat mesilate | NA | NA | NA | NA | NA | -0.54 | -2.28 | 1.00 | 3 | Intransitivity | -0.54 | -2.28 | 1.00 | 1 | Severe Imprecision |
| chloroquine | camostat mesilate | NA | NA | NA | NA | NA | 0.18 | -1.52 | 1.68 | 3 | NA | 0.18 | -1.52 | 1.68 | 1 | Severe Imprecision |
| colchicine | camostat mesilate | NA | NA | NA | NA | NA | -0.92 | -2.70 | 0.65 | 3 | NA | -0.92 | -2.70 | 0.65 | 1 | Severe Imprecision |
| convalescent plasma | camostat mesilate | NA | NA | NA | NA | NA | -0.08 | -1.72 | 1.32 | 2 | Intransitivity | -0.08 | -1.72 | 1.32 | 1 | Severe Imprecision |
| dexamethasone | camostat mesilate | NA | NA | NA | NA | NA | -0.46 | -2.11 | 0.95 | 3 | NA | -0.46 | -2.11 | 0.95 | 1 | Severe Imprecision |
| doxycycline | camostat mesilate | NA | NA | NA | NA | NA | -0.71 | -2.55 | 0.88 | 3 | NA | -0.71 | -2.55 | 0.88 | 1 | Severe Imprecision |
| favipiravir | camostat mesilate | NA | NA | NA | NA | NA | 0.09 | -1.60 | 1.56 | 3 | NA | 0.09 | -1.60 | 1.56 | 1 | Severe Imprecision |
| hydroxychloroquine | camostat mesilate | NA | NA | NA | NA | NA | -0.08 | -1.72 | 1.33 | 3 | NA | -0.08 | -1.72 | 1.33 | 1 | Severe Imprecision |
| hydroxychloroquine + azithromycin | camostat mesilate | NA | NA | NA | NA | NA | 0.44 | -1.30 | 1.97 | 3 | NA | 0.44 | -1.30 | 1.97 | 1 | Severe Imprecision |
| hydroxychloroquine + favipiravir | camostat mesilate | NA | NA | NA | NA | NA | 0.04 | -1.72 | 1.60 | 3 | NA | 0.04 | -1.72 | 1.60 | 1 | Severe Imprecision |
| imatinib | camostat mesilate | NA | NA | NA | NA | NA | 0.01 | -1.70 | 1.51 | 3 | Intransitivity | 0.01 | -1.70 | 1.51 | 1 | Severe Imprecision |
| inm005 | camostat mesilate | NA | NA | NA | NA | NA | -0.31 | -2.06 | 1.25 | 4 | NA | -0.31 | -2.06 | 1.25 | 2 | Severe Imprecision |
| interferon beta | camostat mesilate | NA | NA | NA | NA | NA | -0.08 | -1.72 | 1.33 | 2 | Intransitivity | -0.08 | -1.72 | 1.33 | 1 | Severe Imprecision |
| intravenous immunoglobulin | camostat mesilate | NA | NA | NA | NA | NA | -0.40 | -2.11 | 1.10 | 3 | NA | -0.40 | -2.11 | 1.10 | 1 | Severe Imprecision |
| ivermectin | camostat mesilate | NA | NA | NA | NA | NA | -0.14 | -1.90 | 1.40 | 4 | NA | -0.14 | -1.90 | 1.40 | 2 | Severe Imprecision |
| lopinavir/ritonavir | camostat mesilate | NA | NA | NA | NA | NA | -0.11 | -1.75 | 1.30 | 3 | NA | -0.11 | -1.75 | 1.30 | 1 | Severe Imprecision |
| methylprednisolone | camostat mesilate | NA | NA | NA | NA | NA | -0.31 | -2.00 | 1.17 | 3 | NA | -0.31 | -2.00 | 1.17 | 1 | Severe Imprecision |
| recombinant human gcsf | camostat mesilate | NA | NA | NA | NA | NA | -1.43 | -3.18 | 0.12 | 3 | NA | -1.43 | -3.18 | 0.12 | 1 | Severe Imprecision |
| remdesivir | camostat mesilate | NA | NA | NA | NA | NA | -0.01 | -1.65 | 1.40 | 2 | NA | -0.01 | -1.65 | 1.40 | 1 | Severe Imprecision |
| sarilumab | camostat mesilate | NA | NA | NA | NA | NA | -0.46 | -2.14 | 1.01 | 1 | Intransitivity | -0.46 | -2.14 | 1.01 | 1 | Severe Imprecision |
| sofosbuvir + daclatasvir | camostat mesilate | NA | NA | NA | NA | NA | -0.42 | -2.28 | 1.20 | 3 | NA | -0.42 | -2.28 | 1.20 | 1 | Severe Imprecision |
| sulodexide | camostat mesilate | NA | NA | NA | NA | NA | -0.91 | -3.22 | 1.10 | 3 | NA | -0.91 | -3.22 | 1.10 | 1 | Severe Imprecision |
| tocilizumab | camostat mesilate | NA | NA | NA | NA | NA | -0.35 | -1.99 | 1.06 | 3 | NA | -0.35 | -1.99 | 1.06 | 1 | Severe Imprecision |
| tofacitinib | camostat mesilate | NA | NA | NA | NA | NA | -0.50 | -2.25 | 1.05 | 4 | NA | -0.50 | -2.25 | 1.05 | 2 | Severe Imprecision |
| vitamin d3 | camostat mesilate | NA | NA | NA | NA | NA | -0.81 | -2.65 | 0.85 | 3 | Intransitivity | -0.81 | -2.65 | 0.85 | 1 | Severe Imprecision |
| chloroquine | canakinumab | NA | NA | NA | NA | NA | 0.71 | -0.10 | 1.57 | 2 | Intransitivity | 0.71 | -0.10 | 1.57 | 1 | Severe Imprecision |
| colchicine | canakinumab | NA | NA | NA | NA | NA | -0.38 | -1.33 | 0.54 | 2 | Intransitivity | -0.38 | -1.33 | 0.54 | 1 | Imprecision |
| convalescent plasma | canakinumab | NA | NA | NA | NA | NA | 0.45 | -0.15 | 1.09 | 3 | NA | 0.45 | -0.15 | 1.09 | 2 | Imprecision |
| dexamethasone | canakinumab | NA | NA | NA | NA | NA | 0.07 | -0.55 | 0.73 | 3 | NA | 0.07 | -0.55 | 0.73 | 2 | Imprecision |
| doxycycline | canakinumab | NA | NA | NA | NA | NA | -0.17 | -1.24 | 0.77 | 2 | Intransitivity | -0.17 | -1.24 | 0.77 | 1 | Imprecision |
| favipiravir | canakinumab | NA | NA | NA | NA | NA | 0.62 | -0.13 | 1.43 | 2 | Intransitivity | 0.62 | -0.13 | 1.43 | 1 | Imprecision |
| hydroxychloroquine | canakinumab | NA | NA | NA | NA | NA | 0.45 | -0.15 | 1.10 | 2 | Intransitivity | 0.45 | -0.15 | 1.10 | 1 | Imprecision |
| hydroxychloroquine + azithromycin | canakinumab | NA | NA | NA | NA | NA | 0.98 | 0.12 | 1.86 | 2 | Intransitivity | 0.98 | 0.12 | 1.86 | 2 | NA |
| hydroxychloroquine + favipiravir | canakinumab | NA | NA | NA | NA | NA | 0.58 | -0.33 | 1.51 | 2 | Intransitivity | 0.58 | -0.33 | 1.51 | 1 | Severe Imprecision |
| imatinib | canakinumab | NA | NA | NA | NA | NA | 0.54 | -0.24 | 1.36 | 4 | NA | 0.54 | -0.24 | 1.36 | 3 | Imprecision |
| inm005 | canakinumab | NA | NA | NA | NA | NA | 0.23 | -0.68 | 1.13 | 4 | NA | 0.23 | -0.68 | 1.13 | 3 | Imprecision |
| interferon beta | canakinumab | NA | NA | NA | NA | NA | 0.45 | -0.16 | 1.11 | 3 | NA | 0.45 | -0.16 | 1.11 | 2 | Imprecision |
| intravenous immunoglobulin | canakinumab | NA | NA | NA | NA | NA | 0.13 | -0.67 | 0.96 | 3 | NA | 0.13 | -0.67 | 0.96 | 2 | Imprecision |
| ivermectin | canakinumab | NA | NA | NA | NA | NA | 0.39 | -0.49 | 1.28 | 3 | Intransitivity | 0.39 | -0.49 | 1.28 | 2 | Imprecision |
| lopinavir/ritonavir | canakinumab | NA | NA | NA | NA | NA | 0.42 | -0.18 | 1.07 | 3 | NA | 0.42 | -0.18 | 1.07 | 2 | Imprecision |
| methylprednisolone | canakinumab | NA | NA | NA | NA | NA | 0.22 | -0.53 | 1.01 | 3 | NA | 0.22 | -0.53 | 1.01 | 2 | Imprecision |
| recombinant human gcsf | canakinumab | NA | NA | NA | NA | NA | -0.88 | -1.78 | -0.04 | 2 | Intransitivity | -0.88 | -1.78 | -0.04 | 2 | NA |
| remdesivir | canakinumab | NA | NA | NA | NA | NA | 0.52 | -0.08 | 1.17 | 2 | NA | 0.52 | -0.08 | 1.17 | 1 | Imprecision |
| sarilumab | canakinumab | NA | NA | NA | NA | NA | 0.07 | -0.61 | 0.80 | 2 | NA | 0.07 | -0.61 | 0.80 | 1 | Imprecision |
| sofosbuvir + daclatasvir | canakinumab | NA | NA | NA | NA | NA | 0.13 | -0.95 | 1.09 | 3 | NA | 0.13 | -0.95 | 1.09 | 2 | Imprecision |
| sulodexide | canakinumab | NA | NA | NA | NA | NA | -0.35 | -2.13 | 1.18 | 2 | Intransitivity | -0.35 | -2.13 | 1.18 | 1 | Severe Imprecision |
| tocilizumab | canakinumab | NA | NA | NA | NA | NA | 0.18 | -0.42 | 0.83 | 3 | NA | 0.18 | -0.42 | 0.83 | 2 | Imprecision |
| tofacitinib | canakinumab | NA | NA | NA | NA | NA | 0.04 | -0.78 | 0.88 | 4 | NA | 0.04 | -0.78 | 0.88 | 3 | Imprecision |
| vitamin d3 | canakinumab | NA | NA | NA | NA | NA | -0.27 | -1.34 | 0.79 | 4 | NA | -0.27 | -1.34 | 0.79 | 3 | Imprecision |
| colchicine | chloroquine | NA | NA | NA | NA | NA | -1.10 | -2.03 | -0.21 | 3 | NA | -1.10 | -2.03 | -0.21 | 3 | NA |
| convalescent plasma | chloroquine | NA | NA | NA | NA | NA | -0.26 | -0.83 | 0.29 | 2 | Intransitivity | -0.26 | -0.83 | 0.29 | 1 | Imprecision |
| dexamethasone | chloroquine | NA | NA | NA | NA | NA | -0.64 | -1.23 | -0.07 | 3 | NA | -0.64 | -1.23 | -0.07 | 3 | NA |
| doxycycline | chloroquine | NA | NA | NA | NA | NA | -0.88 | -1.98 | 0.04 | 3 | NA | -0.88 | -1.98 | 0.04 | 1 | Severe Imprecision |
| favipiravir | chloroquine | -0.59 | -2.01 | 0.43 | 3 | RoB; | 0.09 | -0.65 | 0.84 | 3 | NA | -0.09 | -0.79 | 0.60 | 2 | Imprecision |
| hydroxychloroquine | chloroquine | 0.06 | -0.48 | 0.63 | 3 | RoB; | -0.88 | -2.52 | 0.28 | 3 | NA | -0.26 | -0.81 | 0.28 | 2 | Imprecision |
| hydroxychloroquine + azithromycin | chloroquine | NA | NA | NA | NA | NA | 0.26 | -0.56 | 1.08 | 3 | NA | 0.26 | -0.56 | 1.08 | 2 | Imprecision |
| hydroxychloroquine + favipiravir | chloroquine | NA | NA | NA | NA | NA | -0.14 | -1.02 | 0.74 | 3 | NA | -0.14 | -1.02 | 0.74 | 2 | Imprecision |
| imatinib | chloroquine | NA | NA | NA | NA | NA | -0.17 | -0.93 | 0.58 | 2 | Intransitivity | -0.17 | -0.93 | 0.58 | 1 | Imprecision |
| inm005 | chloroquine | NA | NA | NA | NA | NA | -0.48 | -1.38 | 0.37 | 3 | NA | -0.48 | -1.38 | 0.37 | 2 | Imprecision |
| interferon beta | chloroquine | NA | NA | NA | NA | NA | -0.26 | -0.84 | 0.31 | 2 | Intransitivity | -0.26 | -0.84 | 0.31 | 1 | Imprecision |
| intravenous immunoglobulin | chloroquine | NA | NA | NA | NA | NA | -0.58 | -1.35 | 0.19 | 3 | NA | -0.58 | -1.35 | 0.19 | 2 | Imprecision |
| ivermectin | chloroquine | -0.14 | -0.95 | 0.66 | 4 | NA | -0.61 | -1.71 | 0.40 | 3 | NA | -0.32 | -1.04 | 0.38 | 3 | Imprecision |
| lopinavir/ritonavir | chloroquine | NA | NA | NA | NA | NA | -0.29 | -0.86 | 0.27 | 3 | NA | -0.29 | -0.86 | 0.27 | 2 | Imprecision |
| methylprednisolone | chloroquine | NA | NA | NA | NA | NA | -0.49 | -1.23 | 0.23 | 3 | NA | -0.49 | -1.23 | 0.23 | 2 | Imprecision |
| recombinant human gcsf | chloroquine | NA | NA | NA | NA | NA | -1.60 | -2.50 | -0.73 | 3 | NA | -1.60 | -2.50 | -0.73 | 3 | NA |
| remdesivir | chloroquine | NA | NA | NA | NA | NA | -0.19 | -0.76 | 0.37 | 2 | NA | -0.19 | -0.76 | 0.37 | 1 | Imprecision |
| sarilumab | chloroquine | NA | NA | NA | NA | NA | -0.64 | -1.34 | 0.05 | 1 | Intransitivity | -0.64 | -1.34 | 0.05 | 1 | Imprecision |
| sofosbuvir + daclatasvir | chloroquine | NA | NA | NA | NA | NA | -0.58 | -1.68 | 0.34 | 3 | NA | -0.58 | -1.68 | 0.34 | 1 | Severe Imprecision |
| sulodexide | chloroquine | NA | NA | NA | NA | NA | -1.07 | -2.83 | 0.44 | 3 | NA | -1.07 | -2.83 | 0.44 | 1 | Severe Imprecision |
| tocilizumab | chloroquine | NA | NA | NA | NA | NA | -0.53 | -1.10 | 0.04 | 3 | NA | -0.53 | -1.10 | 0.04 | 2 | Imprecision |
| tofacitinib | chloroquine | NA | NA | NA | NA | NA | -0.67 | -1.56 | 0.17 | 3 | NA | -0.67 | -1.56 | 0.17 | 1 | Severe Imprecision |
| vitamin d3 | chloroquine | NA | NA | NA | NA | NA | -0.98 | -2.03 | 0.03 | 2 | Intransitivity | -0.98 | -2.03 | 0.03 | 1 | Severe Imprecision |
| convalescent plasma | colchicine | NA | NA | NA | NA | NA | 0.83 | 0.14 | 1.59 | 2 | Intransitivity | 0.83 | 0.14 | 1.59 | 2 | NA |
| dexamethasone | colchicine | NA | NA | NA | NA | NA | 0.45 | -0.25 | 1.22 | 3 | NA | 0.45 | -0.25 | 1.22 | 2 | Imprecision |
| doxycycline | colchicine | NA | NA | NA | NA | NA | 0.21 | -0.93 | 1.28 | 3 | NA | 0.21 | -0.93 | 1.28 | 2 | Imprecision |
| favipiravir | colchicine | NA | NA | NA | NA | NA | 1.00 | 0.17 | 1.88 | 3 | NA | 1.00 | 0.17 | 1.88 | 3 | NA |
| hydroxychloroquine | colchicine | NA | NA | NA | NA | NA | 0.83 | 0.14 | 1.59 | 3 | NA | 0.83 | 0.14 | 1.59 | 3 | NA |
| hydroxychloroquine + azithromycin | colchicine | NA | NA | NA | NA | NA | 1.36 | 0.44 | 2.32 | 3 | NA | 1.36 | 0.44 | 2.32 | 3 | NA |
| hydroxychloroquine + favipiravir | colchicine | NA | NA | NA | NA | NA | 0.96 | -0.02 | 1.97 | 3 | NA | 0.96 | -0.02 | 1.97 | 1 | Severe Imprecision |
| imatinib | colchicine | NA | NA | NA | NA | NA | 0.92 | 0.06 | 1.83 | 2 | Intransitivity | 0.92 | 0.06 | 1.83 | 2 | NA |
| inm005 | colchicine | NA | NA | NA | NA | NA | 0.61 | -0.36 | 1.60 | 3 | NA | 0.61 | -0.36 | 1.60 | 1 | Severe Imprecision |
| interferon beta | colchicine | NA | NA | NA | NA | NA | 0.83 | 0.13 | 1.60 | 2 | Intransitivity | 0.83 | 0.13 | 1.60 | 2 | NA |
| intravenous immunoglobulin | colchicine | NA | NA | NA | NA | NA | 0.51 | -0.36 | 1.43 | 3 | NA | 0.51 | -0.36 | 1.43 | 2 | Imprecision |
| ivermectin | colchicine | NA | NA | NA | NA | NA | 0.78 | -0.16 | 1.74 | 3 | NA | 0.78 | -0.16 | 1.74 | 1 | Severe Imprecision |
| lopinavir/ritonavir | colchicine | NA | NA | NA | NA | NA | 0.80 | 0.12 | 1.56 | 3 | NA | 0.80 | 0.12 | 1.56 | 3 | NA |
| methylprednisolone | colchicine | NA | NA | NA | NA | NA | 0.60 | -0.21 | 1.49 | 3 | NA | 0.60 | -0.21 | 1.49 | 2 | Imprecision |
| recombinant human gcsf | colchicine | NA | NA | NA | NA | NA | -0.51 | -1.46 | 0.48 | 3 | NA | -0.51 | -1.46 | 0.48 | 2 | Imprecision |
| remdesivir | colchicine | NA | NA | NA | NA | NA | 0.90 | 0.22 | 1.67 | 2 | NA | 0.90 | 0.22 | 1.67 | 2 | NA |
| sarilumab | colchicine | NA | NA | NA | NA | NA | 0.45 | -0.34 | 1.31 | 1 | Intransitivity | 0.45 | -0.34 | 1.31 | 1 | Imprecision |
| sofosbuvir + daclatasvir | colchicine | NA | NA | NA | NA | NA | 0.51 | -0.64 | 1.57 | 3 | NA | 0.51 | -0.64 | 1.57 | 1 | Severe Imprecision |
| sulodexide | colchicine | NA | NA | NA | NA | NA | 0.03 | -1.78 | 1.62 | 3 | NA | 0.03 | -1.78 | 1.62 | 1 | Severe Imprecision |
| tocilizumab | colchicine | NA | NA | NA | NA | NA | 0.56 | -0.12 | 1.33 | 3 | NA | 0.56 | -0.12 | 1.33 | 2 | Imprecision |
| tofacitinib | colchicine | NA | NA | NA | NA | NA | 0.42 | -0.52 | 1.40 | 3 | NA | 0.42 | -0.52 | 1.40 | 2 | Imprecision |
| vitamin d3 | colchicine | NA | NA | NA | NA | NA | 0.12 | -1.01 | 1.24 | 2 | Intransitivity | 0.12 | -1.01 | 1.24 | 1 | Imprecision |
| dexamethasone | convalescent plasma | NA | NA | NA | NA | NA | -0.38 | -0.59 | -0.17 | 3 | NA | -0.38 | -0.59 | -0.17 | 3 | NA |
| doxycycline | convalescent plasma | NA | NA | NA | NA | NA | -0.61 | -1.53 | 0.11 | 2 | Intransitivity | -0.61 | -1.53 | 0.11 | 1 | Severe Imprecision |
| favipiravir | convalescent plasma | NA | NA | NA | NA | NA | 0.17 | -0.31 | 0.66 | 2 | Intransitivity | 0.17 | -0.31 | 0.66 | 1 | Imprecision |
| hydroxychloroquine | convalescent plasma | NA | NA | NA | NA | NA | 0.01 | -0.17 | 0.18 | 2 | Intransitivity | 0.01 | -0.17 | 0.18 | 1 | Imprecision |
| hydroxychloroquine + azithromycin | convalescent plasma | NA | NA | NA | NA | NA | 0.53 | -0.10 | 1.15 | 2 | Intransitivity | 0.53 | -0.10 | 1.15 | 1 | Imprecision |
| hydroxychloroquine + favipiravir | convalescent plasma | NA | NA | NA | NA | NA | 0.13 | -0.57 | 0.82 | 2 | Intransitivity | 0.13 | -0.57 | 0.82 | 1 | Imprecision |
| imatinib | convalescent plasma | NA | NA | NA | NA | NA | 0.09 | -0.43 | 0.62 | 3 | NA | 0.09 | -0.43 | 0.62 | 2 | Imprecision |
| inm005 | convalescent plasma | NA | NA | NA | NA | NA | -0.22 | -0.91 | 0.44 | 3 | NA | -0.22 | -0.91 | 0.44 | 2 | Imprecision |
| interferon beta | convalescent plasma | NA | NA | NA | NA | NA | 0.00 | -0.19 | 0.20 | 3 | NA | 0.00 | -0.19 | 0.20 | 2 | Imprecision |
| intravenous immunoglobulin | convalescent plasma | NA | NA | NA | NA | NA | -0.32 | -0.86 | 0.23 | 3 | NA | -0.32 | -0.86 | 0.23 | 2 | Imprecision |
| ivermectin | convalescent plasma | NA | NA | NA | NA | NA | -0.05 | -0.70 | 0.58 | 2 | Intransitivity | -0.05 | -0.70 | 0.58 | 1 | Imprecision |
| lopinavir/ritonavir | convalescent plasma | NA | NA | NA | NA | NA | -0.02 | -0.20 | 0.15 | 3 | NA | -0.02 | -0.20 | 0.15 | 2 | Imprecision |
| methylprednisolone | convalescent plasma | NA | NA | NA | NA | NA | -0.22 | -0.71 | 0.26 | 3 | NA | -0.22 | -0.71 | 0.26 | 2 | Imprecision |
| recombinant human gcsf | convalescent plasma | NA | NA | NA | NA | NA | -1.34 | -2.01 | -0.69 | 2 | Intransitivity | -1.34 | -2.01 | -0.69 | 2 | NA |
| remdesivir | convalescent plasma | NA | NA | NA | NA | NA | 0.08 | -0.09 | 0.24 | 2 | NA | 0.08 | -0.09 | 0.24 | 1 | Imprecision |
| sarilumab | convalescent plasma | NA | NA | NA | NA | NA | -0.37 | -0.80 | 0.06 | 2 | NA | -0.37 | -0.80 | 0.06 | 1 | Imprecision |
| sofosbuvir + daclatasvir | convalescent plasma | NA | NA | NA | NA | NA | -0.31 | -1.25 | 0.44 | 3 | NA | -0.31 | -1.25 | 0.44 | 2 | Imprecision |
| sulodexide | convalescent plasma | NA | NA | NA | NA | NA | -0.80 | -2.48 | 0.61 | 2 | Intransitivity | -0.80 | -2.48 | 0.61 | 1 | Severe Imprecision |
| tocilizumab | convalescent plasma | NA | NA | NA | NA | NA | -0.26 | -0.44 | -0.09 | 3 | NA | -0.26 | -0.44 | -0.09 | 3 | NA |
| tofacitinib | convalescent plasma | NA | NA | NA | NA | NA | -0.41 | -1.09 | 0.24 | 3 | NA | -0.41 | -1.09 | 0.24 | 2 | Imprecision |
| vitamin d3 | convalescent plasma | NA | NA | NA | NA | NA | -0.71 | -1.63 | 0.14 | 3 | NA | -0.71 | -1.63 | 0.14 | 1 | Severe Imprecision |
| doxycycline | dexamethasone | NA | NA | NA | NA | NA | -0.23 | -1.15 | 0.50 | 3 | NA | -0.23 | -1.15 | 0.50 | 2 | Imprecision |
| favipiravir | dexamethasone | NA | NA | NA | NA | NA | 0.55 | 0.05 | 1.06 | 3 | NA | 0.55 | 0.05 | 1.06 | 3 | NA |
| hydroxychloroquine | dexamethasone | 0.55 | 0.28 | 0.83 | 3 | RoB; | -0.16 | -0.57 | 0.26 | 3 | NA | 0.38 | 0.17 | 0.60 | 3 | NA |
| hydroxychloroquine + azithromycin | dexamethasone | NA | NA | NA | NA | NA | 0.91 | 0.27 | 1.54 | 3 | NA | 0.91 | 0.27 | 1.54 | 3 | NA |
| hydroxychloroquine + favipiravir | dexamethasone | NA | NA | NA | NA | NA | 0.51 | -0.21 | 1.22 | 3 | NA | 0.51 | -0.21 | 1.22 | 2 | Imprecision |
| imatinib | dexamethasone | NA | NA | NA | NA | NA | 0.47 | -0.07 | 1.02 | 3 | NA | 0.47 | -0.07 | 1.02 | 2 | Imprecision |
| inm005 | dexamethasone | NA | NA | NA | NA | NA | 0.16 | -0.55 | 0.84 | 3 | NA | 0.16 | -0.55 | 0.84 | 2 | Imprecision |
| interferon beta | dexamethasone | NA | NA | NA | NA | NA | 0.38 | 0.14 | 0.62 | 3 | NA | 0.38 | 0.14 | 0.62 | 3 | NA |
| intravenous immunoglobulin | dexamethasone | NA | NA | NA | NA | NA | 0.06 | -0.51 | 0.63 | 3 | NA | 0.06 | -0.51 | 0.63 | 2 | Imprecision |
| ivermectin | dexamethasone | NA | NA | NA | NA | NA | 0.32 | -0.33 | 0.97 | 3 | NA | 0.32 | -0.33 | 0.97 | 2 | Imprecision |
| lopinavir/ritonavir | dexamethasone | 0.53 | 0.28 | 0.80 | 3 | RoB; | -0.21 | -0.64 | 0.20 | 3 | NA | 0.35 | 0.14 | 0.57 | 3 | NA |
| methylprednisolone | dexamethasone | -0.45 | -1.23 | 0.18 | 4 | NA | 0.56 | 0.00 | 1.14 | 3 | NA | 0.15 | -0.32 | 0.63 | 3 | Imprecision |
| recombinant human gcsf | dexamethasone | NA | NA | NA | NA | NA | -0.96 | -1.64 | -0.31 | 3 | NA | -0.96 | -1.64 | -0.31 | 3 | NA |
| remdesivir | dexamethasone | NA | NA | NA | NA | NA | 0.45 | 0.24 | 0.67 | 2 | NA | 0.45 | 0.24 | 0.67 | 2 | NA |
| sarilumab | dexamethasone | NA | NA | NA | NA | NA | 0.00 | -0.45 | 0.46 | 2 | NA | 0.00 | -0.45 | 0.46 | 1 | Imprecision |
| sofosbuvir + daclatasvir | dexamethasone | NA | NA | NA | NA | NA | 0.06 | -0.88 | 0.83 | 3 | NA | 0.06 | -0.88 | 0.83 | 2 | Imprecision |
| sulodexide | dexamethasone | NA | NA | NA | NA | NA | -0.42 | -2.11 | 0.99 | 3 | NA | -0.42 | -2.11 | 0.99 | 1 | Severe Imprecision |
| tocilizumab | dexamethasone | NA | NA | NA | NA | NA | 0.11 | -0.11 | 0.35 | 3 | NA | 0.11 | -0.11 | 0.35 | 2 | Imprecision |
| tofacitinib | dexamethasone | NA | NA | NA | NA | NA | -0.03 | -0.72 | 0.64 | 3 | NA | -0.03 | -0.72 | 0.64 | 2 | Imprecision |
| vitamin d3 | dexamethasone | NA | NA | NA | NA | NA | -0.34 | -1.25 | 0.53 | 3 | NA | -0.34 | -1.25 | 0.53 | 2 | Imprecision |
| favipiravir | doxycycline | NA | NA | NA | NA | NA | 0.78 | -0.08 | 1.83 | 3 | NA | 0.78 | -0.08 | 1.83 | 1 | Severe Imprecision |
| hydroxychloroquine | doxycycline | NA | NA | NA | NA | NA | 0.61 | -0.11 | 1.54 | 3 | NA | 0.61 | -0.11 | 1.54 | 1 | Severe Imprecision |
| hydroxychloroquine + azithromycin | doxycycline | NA | NA | NA | NA | NA | 1.14 | 0.20 | 2.24 | 3 | NA | 1.14 | 0.20 | 2.24 | 3 | NA |
| hydroxychloroquine + favipiravir | doxycycline | NA | NA | NA | NA | NA | 0.74 | -0.27 | 1.89 | 3 | NA | 0.74 | -0.27 | 1.89 | 1 | Severe Imprecision |
| imatinib | doxycycline | NA | NA | NA | NA | NA | 0.70 | -0.18 | 1.76 | 2 | Intransitivity | 0.70 | -0.18 | 1.76 | 1 | Severe Imprecision |
| inm005 | doxycycline | NA | NA | NA | NA | NA | 0.40 | -0.57 | 1.48 | 3 | NA | 0.40 | -0.57 | 1.48 | 2 | Imprecision |
| interferon beta | doxycycline | NA | NA | NA | NA | NA | 0.61 | -0.12 | 1.56 | 2 | Intransitivity | 0.61 | -0.12 | 1.56 | 1 | Severe Imprecision |
| intravenous immunoglobulin | doxycycline | NA | NA | NA | NA | NA | 0.30 | -0.60 | 1.35 | 3 | NA | 0.30 | -0.60 | 1.35 | 2 | Imprecision |
| ivermectin | doxycycline | NA | NA | NA | NA | NA | 0.56 | -0.42 | 1.67 | 3 | NA | 0.56 | -0.42 | 1.67 | 1 | Severe Imprecision |
| lopinavir/ritonavir | doxycycline | NA | NA | NA | NA | NA | 0.58 | -0.14 | 1.52 | 3 | NA | 0.58 | -0.14 | 1.52 | 1 | Severe Imprecision |
| methylprednisolone | doxycycline | NA | NA | NA | NA | NA | 0.39 | -0.45 | 1.40 | 3 | NA | 0.39 | -0.45 | 1.40 | 2 | Imprecision |
| recombinant human gcsf | doxycycline | NA | NA | NA | NA | NA | -0.71 | -1.66 | 0.24 | 3 | NA | -0.71 | -1.66 | 0.24 | 1 | Severe Imprecision |
| remdesivir | doxycycline | NA | NA | NA | NA | NA | 0.68 | -0.04 | 1.62 | 2 | NA | 0.68 | -0.04 | 1.62 | 1 | Severe Imprecision |
| sarilumab | doxycycline | NA | NA | NA | NA | NA | 0.24 | -0.59 | 1.24 | 1 | Intransitivity | 0.24 | -0.59 | 1.24 | 1 | Imprecision |
| sofosbuvir + daclatasvir | doxycycline | NA | NA | NA | NA | NA | 0.30 | -0.82 | 1.42 | 3 | NA | 0.30 | -0.82 | 1.42 | 2 | Imprecision |
| sulodexide | doxycycline | NA | NA | NA | NA | NA | -0.18 | -2.03 | 1.49 | 3 | NA | -0.18 | -2.03 | 1.49 | 1 | Severe Imprecision |
| tocilizumab | doxycycline | NA | NA | NA | NA | NA | 0.34 | -0.38 | 1.28 | 3 | NA | 0.34 | -0.38 | 1.28 | 2 | Imprecision |
| tofacitinib | doxycycline | NA | NA | NA | NA | NA | 0.20 | -0.76 | 1.32 | 3 | NA | 0.20 | -0.76 | 1.32 | 2 | Imprecision |
| vitamin d3 | doxycycline | NA | NA | NA | NA | NA | -0.10 | -1.25 | 1.12 | 2 | Intransitivity | -0.10 | -1.25 | 1.12 | 1 | Imprecision |
| hydroxychloroquine | favipiravir | NA | NA | NA | NA | NA | -0.16 | -0.66 | 0.32 | 3 | NA | -0.16 | -0.66 | 0.32 | 2 | Imprecision |
| hydroxychloroquine + azithromycin | favipiravir | NA | NA | NA | NA | NA | 0.36 | -0.42 | 1.12 | 3 | NA | 0.36 | -0.42 | 1.12 | 2 | Imprecision |
| hydroxychloroquine + favipiravir | favipiravir | NA | NA | NA | NA | NA | -0.04 | -0.88 | 0.79 | 3 | NA | -0.04 | -0.88 | 0.79 | 2 | Imprecision |
| imatinib | favipiravir | NA | NA | NA | NA | NA | -0.08 | -0.78 | 0.62 | 2 | Intransitivity | -0.08 | -0.78 | 0.62 | 1 | Imprecision |
| inm005 | favipiravir | NA | NA | NA | NA | NA | -0.39 | -1.23 | 0.42 | 3 | NA | -0.39 | -1.23 | 0.42 | 2 | Imprecision |
| interferon beta | favipiravir | NA | NA | NA | NA | NA | -0.17 | -0.66 | 0.32 | 2 | Intransitivity | -0.17 | -0.66 | 0.32 | 1 | Imprecision |
| intravenous immunoglobulin | favipiravir | NA | NA | NA | NA | NA | -0.49 | -1.21 | 0.23 | 3 | NA | -0.49 | -1.21 | 0.23 | 2 | Imprecision |
| ivermectin | favipiravir | NA | NA | NA | NA | NA | -0.23 | -1.01 | 0.55 | 3 | NA | -0.23 | -1.01 | 0.55 | 2 | Imprecision |
| lopinavir/ritonavir | favipiravir | -0.29 | -0.83 | 0.21 | 3 | RoB; | 0.12 | -0.56 | 0.83 | 3 | NA | -0.19 | -0.67 | 0.27 | 2 | Imprecision |
| methylprednisolone | favipiravir | NA | NA | NA | NA | NA | -0.39 | -1.07 | 0.27 | 3 | NA | -0.39 | -1.07 | 0.27 | 2 | Imprecision |
| recombinant human gcsf | favipiravir | NA | NA | NA | NA | NA | -1.51 | -2.35 | -0.70 | 3 | NA | -1.51 | -2.35 | -0.70 | 3 | NA |
| remdesivir | favipiravir | NA | NA | NA | NA | NA | -0.09 | -0.58 | 0.38 | 2 | NA | -0.09 | -0.58 | 0.38 | 1 | Imprecision |
| sarilumab | favipiravir | NA | NA | NA | NA | NA | -0.54 | -1.18 | 0.08 | 1 | Intransitivity | -0.54 | -1.18 | 0.08 | 1 | Imprecision |
| sofosbuvir + daclatasvir | favipiravir | NA | NA | NA | NA | NA | -0.48 | -1.51 | 0.34 | 3 | NA | -0.48 | -1.51 | 0.34 | 1 | Severe Imprecision |
| sulodexide | favipiravir | NA | NA | NA | NA | NA | -0.98 | -2.72 | 0.52 | 3 | NA | -0.98 | -2.72 | 0.52 | 1 | Severe Imprecision |
| tocilizumab | favipiravir | -0.53 | -1.71 | 0.49 | 3 | RoB; | -0.41 | -0.91 | 0.07 | 3 | NA | -0.43 | -0.93 | 0.06 | 2 | Imprecision |
| tofacitinib | favipiravir | NA | NA | NA | NA | NA | -0.58 | -1.42 | 0.21 | 3 | NA | -0.58 | -1.42 | 0.21 | 2 | Imprecision |
| vitamin d3 | favipiravir | NA | NA | NA | NA | NA | -0.89 | -1.90 | 0.08 | 2 | Intransitivity | -0.89 | -1.90 | 0.08 | 1 | Severe Imprecision |
| hydroxychloroquine + azithromycin | hydroxychloroquine | 0.47 | -0.24 | 1.23 | 3 | RoB; | 0.72 | -0.49 | 2.04 | 3 | NA | 0.52 | -0.09 | 1.13 | 2 | Imprecision |
| hydroxychloroquine + favipiravir | hydroxychloroquine | NA | NA | NA | NA | NA | 0.12 | -0.58 | 0.83 | 3 | NA | 0.12 | -0.58 | 0.83 | 2 | Imprecision |
| imatinib | hydroxychloroquine | NA | NA | NA | NA | NA | 0.08 | -0.44 | 0.62 | 2 | Intransitivity | 0.08 | -0.44 | 0.62 | 1 | Imprecision |
| inm005 | hydroxychloroquine | NA | NA | NA | NA | NA | -0.22 | -0.92 | 0.44 | 3 | NA | -0.22 | -0.92 | 0.44 | 2 | Imprecision |
| interferon beta | hydroxychloroquine | 0.18 | -0.09 | 0.46 | 3 | RoB; | -0.57 | -1.09 | -0.06 | 2 | Intransitivity | 0.00 | -0.20 | 0.20 | 2 | Imprecision |
| intravenous immunoglobulin | hydroxychloroquine | NA | NA | NA | NA | NA | -0.32 | -0.88 | 0.24 | 3 | NA | -0.32 | -0.88 | 0.24 | 2 | Imprecision |
| ivermectin | hydroxychloroquine | -0.19 | -1.01 | 0.63 | 4 | NA | -0.14 | -0.94 | 0.67 | 3 | NA | -0.06 | -0.70 | 0.56 | 3 | Imprecision |
| lopinavir/ritonavir | hydroxychloroquine | 0.03 | -0.16 | 0.22 | 3 | RoB; | -0.41 | -0.98 | 0.14 | 3 | NA | -0.03 | -0.21 | 0.15 | 2 | Imprecision |
| methylprednisolone | hydroxychloroquine | NA | NA | NA | NA | NA | -0.23 | -0.72 | 0.26 | 3 | NA | -0.23 | -0.72 | 0.26 | 2 | Imprecision |
| recombinant human gcsf | hydroxychloroquine | NA | NA | NA | NA | NA | -1.34 | -2.02 | -0.69 | 3 | NA | -1.34 | -2.02 | -0.69 | 3 | NA |
| remdesivir | hydroxychloroquine | 0.31 | 0.05 | 0.57 | 3 | RoB; | -0.29 | -0.58 | 0.00 | 2 | NA | 0.07 | -0.11 | 0.25 | 2 | Imprecision |
| sarilumab | hydroxychloroquine | NA | NA | NA | NA | NA | -0.38 | -0.82 | 0.06 | 1 | Intransitivity | -0.38 | -0.82 | 0.06 | 1 | Imprecision |
| sofosbuvir + daclatasvir | hydroxychloroquine | NA | NA | NA | NA | NA | -0.32 | -1.26 | 0.44 | 3 | NA | -0.32 | -1.26 | 0.44 | 2 | Imprecision |
| sulodexide | hydroxychloroquine | NA | NA | NA | NA | NA | -0.81 | -2.49 | 0.60 | 3 | NA | -0.81 | -2.49 | 0.60 | 1 | Severe Imprecision |
| tocilizumab | hydroxychloroquine | NA | NA | NA | NA | NA | -0.27 | -0.47 | -0.07 | 3 | NA | -0.27 | -0.47 | -0.07 | 3 | NA |
| tofacitinib | hydroxychloroquine | NA | NA | NA | NA | NA | -0.41 | -1.10 | 0.24 | 3 | NA | -0.41 | -1.10 | 0.24 | 2 | Imprecision |
| vitamin d3 | hydroxychloroquine | NA | NA | NA | NA | NA | -0.72 | -1.63 | 0.14 | 2 | Intransitivity | -0.72 | -1.63 | 0.14 | 1 | Severe Imprecision |
| hydroxychloroquine + favipiravir | hydroxychloroquine + azithromycin | NA | NA | NA | NA | NA | -0.40 | -1.33 | 0.52 | 3 | NA | -0.40 | -1.33 | 0.52 | 2 | Imprecision |
| imatinib | hydroxychloroquine + azithromycin | NA | NA | NA | NA | NA | -0.44 | -1.23 | 0.37 | 2 | Intransitivity | -0.44 | -1.23 | 0.37 | 1 | Imprecision |
| inm005 | hydroxychloroquine + azithromycin | NA | NA | NA | NA | NA | -0.75 | -1.66 | 0.15 | 3 | NA | -0.75 | -1.66 | 0.15 | 1 | Severe Imprecision |
| interferon beta | hydroxychloroquine + azithromycin | NA | NA | NA | NA | NA | -0.53 | -1.15 | 0.11 | 2 | Intransitivity | -0.53 | -1.15 | 0.11 | 1 | Imprecision |
| intravenous immunoglobulin | hydroxychloroquine + azithromycin | NA | NA | NA | NA | NA | -0.84 | -1.66 | -0.03 | 3 | NA | -0.84 | -1.66 | -0.03 | 3 | NA |
| ivermectin | hydroxychloroquine + azithromycin | NA | NA | NA | NA | NA | -0.58 | -1.47 | 0.30 | 3 | NA | -0.58 | -1.47 | 0.30 | 2 | Imprecision |
| lopinavir/ritonavir | hydroxychloroquine + azithromycin | NA | NA | NA | NA | NA | -0.55 | -1.18 | 0.08 | 3 | NA | -0.55 | -1.18 | 0.08 | 2 | Imprecision |
| methylprednisolone | hydroxychloroquine + azithromycin | NA | NA | NA | NA | NA | -0.75 | -1.52 | 0.02 | 3 | NA | -0.75 | -1.52 | 0.02 | 1 | Severe Imprecision |
| recombinant human gcsf | hydroxychloroquine + azithromycin | NA | NA | NA | NA | NA | -1.87 | -2.77 | -0.98 | 3 | NA | -1.87 | -2.77 | -0.98 | 3 | NA |
| remdesivir | hydroxychloroquine + azithromycin | NA | NA | NA | NA | NA | -0.45 | -1.08 | 0.18 | 2 | NA | -0.45 | -1.08 | 0.18 | 1 | Imprecision |
| sarilumab | hydroxychloroquine + azithromycin | NA | NA | NA | NA | NA | -0.90 | -1.64 | -0.16 | 1 | Intransitivity | -0.90 | -1.64 | -0.16 | 1 | NA |
| sofosbuvir + daclatasvir | hydroxychloroquine + azithromycin | NA | NA | NA | NA | NA | -0.85 | -1.95 | 0.13 | 3 | NA | -0.85 | -1.95 | 0.13 | 1 | Severe Imprecision |
| sulodexide | hydroxychloroquine + azithromycin | NA | NA | NA | NA | NA | -1.33 | -3.11 | 0.19 | 3 | NA | -1.33 | -3.11 | 0.19 | 1 | Severe Imprecision |
| tocilizumab | hydroxychloroquine + azithromycin | NA | NA | NA | NA | NA | -0.79 | -1.42 | -0.16 | 3 | NA | -0.79 | -1.42 | -0.16 | 3 | NA |
| tofacitinib | hydroxychloroquine + azithromycin | NA | NA | NA | NA | NA | -0.94 | -1.85 | -0.05 | 3 | NA | -0.94 | -1.85 | -0.05 | 3 | NA |
| vitamin d3 | hydroxychloroquine + azithromycin | NA | NA | NA | NA | NA | -1.24 | -2.33 | -0.20 | 2 | Intransitivity | -1.24 | -2.33 | -0.20 | 2 | NA |
| imatinib | hydroxychloroquine + favipiravir | NA | NA | NA | NA | NA | -0.04 | -0.90 | 0.83 | 2 | Intransitivity | -0.04 | -0.90 | 0.83 | 1 | Imprecision |
| inm005 | hydroxychloroquine + favipiravir | NA | NA | NA | NA | NA | -0.35 | -1.31 | 0.60 | 3 | NA | -0.35 | -1.31 | 0.60 | 2 | Imprecision |
| interferon beta | hydroxychloroquine + favipiravir | NA | NA | NA | NA | NA | -0.12 | -0.83 | 0.58 | 2 | Intransitivity | -0.12 | -0.83 | 0.58 | 1 | Imprecision |
| intravenous immunoglobulin | hydroxychloroquine + favipiravir | NA | NA | NA | NA | NA | -0.44 | -1.32 | 0.43 | 3 | NA | -0.44 | -1.32 | 0.43 | 2 | Imprecision |
| ivermectin | hydroxychloroquine + favipiravir | NA | NA | NA | NA | NA | -0.18 | -1.11 | 0.75 | 3 | NA | -0.18 | -1.11 | 0.75 | 2 | Imprecision |
| lopinavir/ritonavir | hydroxychloroquine + favipiravir | NA | NA | NA | NA | NA | -0.15 | -0.86 | 0.55 | 3 | NA | -0.15 | -0.86 | 0.55 | 2 | Imprecision |
| methylprednisolone | hydroxychloroquine + favipiravir | NA | NA | NA | NA | NA | -0.35 | -1.19 | 0.48 | 3 | NA | -0.35 | -1.19 | 0.48 | 2 | Imprecision |
| recombinant human gcsf | hydroxychloroquine + favipiravir | NA | NA | NA | NA | NA | -1.47 | -2.42 | -0.52 | 3 | NA | -1.47 | -2.42 | -0.52 | 3 | NA |
| remdesivir | hydroxychloroquine + favipiravir | NA | NA | NA | NA | NA | -0.05 | -0.75 | 0.65 | 2 | NA | -0.05 | -0.75 | 0.65 | 1 | Imprecision |
| sarilumab | hydroxychloroquine + favipiravir | NA | NA | NA | NA | NA | -0.50 | -1.31 | 0.30 | 1 | Intransitivity | -0.50 | -1.31 | 0.30 | 1 | Imprecision |
| sofosbuvir + daclatasvir | hydroxychloroquine + favipiravir | NA | NA | NA | NA | NA | -0.45 | -1.60 | 0.58 | 3 | NA | -0.45 | -1.60 | 0.58 | 1 | Severe Imprecision |
| sulodexide | hydroxychloroquine + favipiravir | NA | NA | NA | NA | NA | -0.94 | -2.72 | 0.65 | 3 | NA | -0.94 | -2.72 | 0.65 | 1 | Severe Imprecision |
| tocilizumab | hydroxychloroquine + favipiravir | NA | NA | NA | NA | NA | -0.39 | -1.09 | 0.32 | 3 | NA | -0.39 | -1.09 | 0.32 | 2 | Imprecision |
| tofacitinib | hydroxychloroquine + favipiravir | NA | NA | NA | NA | NA | -0.54 | -1.49 | 0.40 | 3 | NA | -0.54 | -1.49 | 0.40 | 2 | Imprecision |
| vitamin d3 | hydroxychloroquine + favipiravir | NA | NA | NA | NA | NA | -0.84 | -1.97 | 0.25 | 2 | Intransitivity | -0.84 | -1.97 | 0.25 | 1 | Severe Imprecision |
| inm005 | imatinib | NA | NA | NA | NA | NA | -0.30 | -1.10 | 0.42 | 4 | NA | -0.30 | -1.10 | 0.42 | 3 | Imprecision |
| interferon beta | imatinib | NA | NA | NA | NA | NA | -0.09 | -0.63 | 0.45 | 3 | NA | -0.09 | -0.63 | 0.45 | 2 | Imprecision |
| intravenous immunoglobulin | imatinib | NA | NA | NA | NA | NA | -0.41 | -1.16 | 0.34 | 3 | NA | -0.41 | -1.16 | 0.34 | 2 | Imprecision |
| ivermectin | imatinib | NA | NA | NA | NA | NA | -0.15 | -0.97 | 0.67 | 3 | Intransitivity | -0.15 | -0.97 | 0.67 | 2 | Imprecision |
| lopinavir/ritonavir | imatinib | NA | NA | NA | NA | NA | -0.11 | -0.65 | 0.42 | 3 | NA | -0.11 | -0.65 | 0.42 | 2 | Imprecision |
| methylprednisolone | imatinib | NA | NA | NA | NA | NA | -0.31 | -1.02 | 0.38 | 3 | NA | -0.31 | -1.02 | 0.38 | 2 | Imprecision |
| recombinant human gcsf | imatinib | NA | NA | NA | NA | NA | -1.43 | -2.29 | -0.60 | 2 | Intransitivity | -1.43 | -2.29 | -0.60 | 2 | NA |
| remdesivir | imatinib | NA | NA | NA | NA | NA | -0.02 | -0.55 | 0.52 | 2 | NA | -0.02 | -0.55 | 0.52 | 1 | Imprecision |
| sarilumab | imatinib | NA | NA | NA | NA | NA | -0.46 | -1.13 | 0.19 | 2 | NA | -0.46 | -1.13 | 0.19 | 1 | Imprecision |
| sofosbuvir + daclatasvir | imatinib | NA | NA | NA | NA | NA | -0.41 | -1.48 | 0.51 | 3 | NA | -0.41 | -1.48 | 0.51 | 2 | Imprecision |
| sulodexide | imatinib | NA | NA | NA | NA | NA | -0.90 | -2.65 | 0.61 | 2 | Intransitivity | -0.90 | -2.65 | 0.61 | 1 | Severe Imprecision |
| tocilizumab | imatinib | NA | NA | NA | NA | NA | -0.35 | -0.89 | 0.18 | 3 | NA | -0.35 | -0.89 | 0.18 | 2 | Imprecision |
| tofacitinib | imatinib | NA | NA | NA | NA | NA | -0.50 | -1.36 | 0.32 | 4 | NA | -0.50 | -1.36 | 0.32 | 3 | Imprecision |
| vitamin d3 | imatinib | NA | NA | NA | NA | NA | -0.81 | -1.84 | 0.18 | 4 | NA | -0.81 | -1.84 | 0.18 | 2 | Severe Imprecision |
| interferon beta | inm005 | NA | NA | NA | NA | NA | 0.22 | -0.45 | 0.93 | 3 | NA | 0.22 | -0.45 | 0.93 | 2 | Imprecision |
| intravenous immunoglobulin | inm005 | NA | NA | NA | NA | NA | -0.10 | -0.94 | 0.77 | 3 | NA | -0.10 | -0.94 | 0.77 | 2 | Imprecision |
| ivermectin | inm005 | NA | NA | NA | NA | NA | 0.16 | -0.75 | 1.09 | 4 | NA | 0.16 | -0.75 | 1.09 | 3 | Imprecision |
| lopinavir/ritonavir | inm005 | NA | NA | NA | NA | NA | 0.19 | -0.47 | 0.89 | 3 | NA | 0.19 | -0.47 | 0.89 | 2 | Imprecision |
| methylprednisolone | inm005 | NA | NA | NA | NA | NA | -0.01 | -0.81 | 0.82 | 3 | NA | -0.01 | -0.81 | 0.82 | 2 | Imprecision |
| recombinant human gcsf | inm005 | NA | NA | NA | NA | NA | -1.11 | -2.03 | -0.25 | 3 | NA | -1.11 | -2.03 | -0.25 | 3 | NA |
| remdesivir | inm005 | NA | NA | NA | NA | NA | 0.29 | -0.37 | 0.99 | 2 | NA | 0.29 | -0.37 | 0.99 | 1 | Imprecision |
| sarilumab | inm005 | NA | NA | NA | NA | NA | -0.16 | -0.92 | 0.64 | 2 | NA | -0.16 | -0.92 | 0.64 | 1 | Imprecision |
| sofosbuvir + daclatasvir | inm005 | NA | NA | NA | NA | NA | -0.11 | -1.20 | 0.90 | 3 | NA | -0.11 | -1.20 | 0.90 | 2 | Imprecision |
| sulodexide | inm005 | NA | NA | NA | NA | NA | -0.59 | -2.38 | 0.98 | 3 | NA | -0.59 | -2.38 | 0.98 | 1 | Severe Imprecision |
| tocilizumab | inm005 | NA | NA | NA | NA | NA | -0.05 | -0.71 | 0.65 | 3 | NA | -0.05 | -0.71 | 0.65 | 2 | Imprecision |
| tofacitinib | inm005 | NA | NA | NA | NA | NA | -0.19 | -1.12 | 0.75 | 4 | NA | -0.19 | -1.12 | 0.75 | 3 | Imprecision |
| vitamin d3 | inm005 | NA | NA | NA | NA | NA | -0.50 | -1.61 | 0.59 | 4 | NA | -0.50 | -1.61 | 0.59 | 2 | Severe Imprecision |
| intravenous immunoglobulin | interferon beta | NA | NA | NA | NA | NA | -0.32 | -0.88 | 0.25 | 3 | NA | -0.32 | -0.88 | 0.25 | 2 | Imprecision |
| ivermectin | interferon beta | NA | NA | NA | NA | NA | -0.06 | -0.71 | 0.58 | 2 | Intransitivity | -0.06 | -0.71 | 0.58 | 1 | Imprecision |
| lopinavir/ritonavir | interferon beta | -0.09 | -0.33 | 0.14 | 3 | RoB; | 0.51 | 0.00 | 1.03 | 3 | NA | -0.03 | -0.22 | 0.17 | 2 | Imprecision |
| methylprednisolone | interferon beta | NA | NA | NA | NA | NA | -0.23 | -0.73 | 0.27 | 3 | NA | -0.23 | -0.73 | 0.27 | 2 | Imprecision |
| recombinant human gcsf | interferon beta | NA | NA | NA | NA | NA | -1.34 | -2.03 | -0.68 | 2 | Intransitivity | -1.34 | -2.03 | -0.68 | 2 | NA |
| remdesivir | interferon beta | 0.13 | -0.06 | 0.32 | 3 | RoB; | 0.34 | -0.19 | 0.85 | 2 | NA | 0.07 | -0.11 | 0.25 | 2 | Imprecision |
| sarilumab | interferon beta | NA | NA | NA | NA | NA | -0.38 | -0.82 | 0.07 | 2 | NA | -0.38 | -0.82 | 0.07 | 1 | Imprecision |
| sofosbuvir + daclatasvir | interferon beta | NA | NA | NA | NA | NA | -0.31 | -1.27 | 0.44 | 3 | NA | -0.31 | -1.27 | 0.44 | 2 | Imprecision |
| sulodexide | interferon beta | NA | NA | NA | NA | NA | -0.80 | -2.49 | 0.61 | 2 | Intransitivity | -0.80 | -2.49 | 0.61 | 1 | Severe Imprecision |
| tocilizumab | interferon beta | NA | NA | NA | NA | NA | -0.27 | -0.48 | -0.05 | 3 | NA | -0.27 | -0.48 | -0.05 | 3 | NA |
| tofacitinib | interferon beta | NA | NA | NA | NA | NA | -0.41 | -1.11 | 0.25 | 3 | NA | -0.41 | -1.11 | 0.25 | 2 | Imprecision |
| vitamin d3 | interferon beta | NA | NA | NA | NA | NA | -0.72 | -1.63 | 0.15 | 3 | NA | -0.72 | -1.63 | 0.15 | 1 | Severe Imprecision |
| ivermectin | intravenous immunoglobulin | NA | NA | NA | NA | NA | 0.26 | -0.57 | 1.09 | 3 | NA | 0.26 | -0.57 | 1.09 | 2 | Imprecision |
| lopinavir/ritonavir | intravenous immunoglobulin | NA | NA | NA | NA | NA | 0.29 | -0.26 | 0.85 | 3 | NA | 0.29 | -0.26 | 0.85 | 2 | Imprecision |
| methylprednisolone | intravenous immunoglobulin | NA | NA | NA | NA | NA | 0.09 | -0.62 | 0.81 | 3 | NA | 0.09 | -0.62 | 0.81 | 2 | Imprecision |
| recombinant human gcsf | intravenous immunoglobulin | NA | NA | NA | NA | NA | -1.02 | -1.87 | -0.19 | 3 | NA | -1.02 | -1.87 | -0.19 | 3 | NA |
| remdesivir | intravenous immunoglobulin | NA | NA | NA | NA | NA | 0.39 | -0.16 | 0.95 | 2 | NA | 0.39 | -0.16 | 0.95 | 1 | Imprecision |
| sarilumab | intravenous immunoglobulin | NA | NA | NA | NA | NA | -0.06 | -0.74 | 0.63 | 2 | NA | -0.06 | -0.74 | 0.63 | 1 | Imprecision |
| sofosbuvir + daclatasvir | intravenous immunoglobulin | NA | NA | NA | NA | NA | -0.01 | -1.06 | 0.92 | 3 | NA | -0.01 | -1.06 | 0.92 | 2 | Imprecision |
| sulodexide | intravenous immunoglobulin | NA | NA | NA | NA | NA | -0.49 | -2.24 | 1.03 | 3 | NA | -0.49 | -2.24 | 1.03 | 1 | Severe Imprecision |
| tocilizumab | intravenous immunoglobulin | NA | NA | NA | NA | NA | 0.05 | -0.51 | 0.61 | 3 | NA | 0.05 | -0.51 | 0.61 | 2 | Imprecision |
| tofacitinib | intravenous immunoglobulin | NA | NA | NA | NA | NA | -0.09 | -0.95 | 0.74 | 3 | NA | -0.09 | -0.95 | 0.74 | 2 | Imprecision |
| vitamin d3 | intravenous immunoglobulin | NA | NA | NA | NA | NA | -0.40 | -1.45 | 0.60 | 3 | NA | -0.40 | -1.45 | 0.60 | 2 | Imprecision |
| lopinavir/ritonavir | ivermectin | NA | NA | NA | NA | NA | 0.03 | -0.60 | 0.68 | 3 | NA | 0.03 | -0.60 | 0.68 | 2 | Imprecision |
| methylprednisolone | ivermectin | NA | NA | NA | NA | NA | -0.17 | -0.95 | 0.62 | 3 | NA | -0.17 | -0.95 | 0.62 | 2 | Imprecision |
| recombinant human gcsf | ivermectin | NA | NA | NA | NA | NA | -1.29 | -2.20 | -0.37 | 3 | NA | -1.29 | -2.20 | -0.37 | 3 | NA |
| remdesivir | ivermectin | NA | NA | NA | NA | NA | 0.13 | -0.50 | 0.78 | 2 | NA | 0.13 | -0.50 | 0.78 | 1 | Imprecision |
| sarilumab | ivermectin | NA | NA | NA | NA | NA | -0.32 | -1.07 | 0.44 | 1 | Intransitivity | -0.32 | -1.07 | 0.44 | 1 | Imprecision |
| sofosbuvir + daclatasvir | ivermectin | NA | NA | NA | NA | NA | -0.27 | -1.38 | 0.73 | 3 | NA | -0.27 | -1.38 | 0.73 | 2 | Imprecision |
| sulodexide | ivermectin | NA | NA | NA | NA | NA | -0.75 | -2.53 | 0.79 | 3 | NA | -0.75 | -2.53 | 0.79 | 1 | Severe Imprecision |
| tocilizumab | ivermectin | NA | NA | NA | NA | NA | -0.21 | -0.85 | 0.44 | 3 | NA | -0.21 | -0.85 | 0.44 | 2 | Imprecision |
| tofacitinib | ivermectin | NA | NA | NA | NA | NA | -0.35 | -1.27 | 0.55 | 4 | NA | -0.35 | -1.27 | 0.55 | 3 | Imprecision |
| vitamin d3 | ivermectin | NA | NA | NA | NA | NA | -0.66 | -1.75 | 0.40 | 3 | Intransitivity | -0.66 | -1.75 | 0.40 | 1 | Severe Imprecision |
| methylprednisolone | lopinavir/ritonavir | NA | NA | NA | NA | NA | -0.20 | -0.69 | 0.29 | 3 | NA | -0.20 | -0.69 | 0.29 | 2 | Imprecision |
| recombinant human gcsf | lopinavir/ritonavir | NA | NA | NA | NA | NA | -1.31 | -1.99 | -0.66 | 3 | NA | -1.31 | -1.99 | -0.66 | 3 | NA |
| remdesivir | lopinavir/ritonavir | 0.21 | 0.00 | 0.43 | 3 | RoB; | -0.22 | -0.51 | 0.07 | 2 | NA | 0.10 | -0.07 | 0.27 | 2 | Imprecision |
| sarilumab | lopinavir/ritonavir | NA | NA | NA | NA | NA | -0.35 | -0.79 | 0.09 | 2 | NA | -0.35 | -0.79 | 0.09 | 1 | Imprecision |
| sofosbuvir + daclatasvir | lopinavir/ritonavir | NA | NA | NA | NA | NA | -0.29 | -1.23 | 0.46 | 3 | NA | -0.29 | -1.23 | 0.46 | 2 | Imprecision |
| sulodexide | lopinavir/ritonavir | NA | NA | NA | NA | NA | -0.78 | -2.46 | 0.63 | 3 | NA | -0.78 | -2.46 | 0.63 | 1 | Severe Imprecision |
| tocilizumab | lopinavir/ritonavir | NA | NA | NA | NA | NA | -0.24 | -0.44 | -0.04 | 3 | NA | -0.24 | -0.44 | -0.04 | 3 | NA |
| tofacitinib | lopinavir/ritonavir | NA | NA | NA | NA | NA | -0.38 | -1.07 | 0.27 | 3 | NA | -0.38 | -1.07 | 0.27 | 2 | Imprecision |
| vitamin d3 | lopinavir/ritonavir | NA | NA | NA | NA | NA | -0.69 | -1.61 | 0.17 | 3 | NA | -0.69 | -1.61 | 0.17 | 1 | Severe Imprecision |
| recombinant human gcsf | methylprednisolone | NA | NA | NA | NA | NA | -1.11 | -1.93 | -0.33 | 3 | NA | -1.11 | -1.93 | -0.33 | 3 | NA |
| remdesivir | methylprednisolone | NA | NA | NA | NA | NA | 0.30 | -0.18 | 0.79 | 2 | NA | 0.30 | -0.18 | 0.79 | 1 | Imprecision |
| sarilumab | methylprednisolone | NA | NA | NA | NA | NA | -0.15 | -0.77 | 0.48 | 2 | NA | -0.15 | -0.77 | 0.48 | 1 | Imprecision |
| sofosbuvir + daclatasvir | methylprednisolone | NA | NA | NA | NA | NA | -0.10 | -1.13 | 0.79 | 3 | NA | -0.10 | -1.13 | 0.79 | 2 | Imprecision |
| sulodexide | methylprednisolone | NA | NA | NA | NA | NA | -0.58 | -2.32 | 0.91 | 3 | NA | -0.58 | -2.32 | 0.91 | 1 | Severe Imprecision |
| tocilizumab | methylprednisolone | NA | NA | NA | NA | NA | -0.04 | -0.53 | 0.45 | 3 | NA | -0.04 | -0.53 | 0.45 | 2 | Imprecision |
| tofacitinib | methylprednisolone | NA | NA | NA | NA | NA | -0.18 | -1.01 | 0.62 | 3 | NA | -0.18 | -1.01 | 0.62 | 2 | Imprecision |
| vitamin d3 | methylprednisolone | NA | NA | NA | NA | NA | -0.49 | -1.50 | 0.48 | 3 | NA | -0.49 | -1.50 | 0.48 | 1 | Severe Imprecision |
| remdesivir | recombinant human gcsf | NA | NA | NA | NA | NA | 1.41 | 0.76 | 2.09 | 2 | NA | 1.41 | 0.76 | 2.09 | 2 | NA |
| sarilumab | recombinant human gcsf | NA | NA | NA | NA | NA | 0.96 | 0.22 | 1.75 | 1 | Intransitivity | 0.96 | 0.22 | 1.75 | 1 | NA |
| sofosbuvir + daclatasvir | recombinant human gcsf | NA | NA | NA | NA | NA | 1.00 | 0.05 | 1.98 | 3 | NA | 1.00 | 0.05 | 1.98 | 3 | NA |
| sulodexide | recombinant human gcsf | NA | NA | NA | NA | NA | 0.54 | -1.26 | 2.09 | 3 | NA | 0.54 | -1.26 | 2.09 | 1 | Severe Imprecision |
| tocilizumab | recombinant human gcsf | NA | NA | NA | NA | NA | 1.08 | 0.42 | 1.75 | 3 | NA | 1.08 | 0.42 | 1.75 | 3 | NA |
| tofacitinib | recombinant human gcsf | NA | NA | NA | NA | NA | 0.92 | 0.05 | 1.87 | 3 | NA | 0.92 | 0.05 | 1.87 | 3 | NA |
| vitamin d3 | recombinant human gcsf | NA | NA | NA | NA | NA | 0.62 | -0.48 | 1.69 | 2 | Intransitivity | 0.62 | -0.48 | 1.69 | 1 | Severe Imprecision |
| sarilumab | remdesivir | NA | NA | NA | NA | NA | -0.45 | -0.88 | -0.01 | 2 | NA | -0.45 | -0.88 | -0.01 | 1 | Imprecision |
| sofosbuvir + daclatasvir | remdesivir | NA | NA | NA | NA | NA | -0.39 | -1.33 | 0.36 | 2 | NA | -0.39 | -1.33 | 0.36 | 1 | Imprecision |
| sulodexide | remdesivir | NA | NA | NA | NA | NA | -0.88 | -2.56 | 0.53 | 2 | NA | -0.88 | -2.56 | 0.53 | 1 | Severe Imprecision |
| tocilizumab | remdesivir | NA | NA | NA | NA | NA | -0.34 | -0.53 | -0.15 | 2 | NA | -0.34 | -0.53 | -0.15 | 2 | NA |
| tofacitinib | remdesivir | NA | NA | NA | NA | NA | -0.48 | -1.17 | 0.17 | 2 | NA | -0.48 | -1.17 | 0.17 | 1 | Imprecision |
| vitamin d3 | remdesivir | NA | NA | NA | NA | NA | -0.79 | -1.70 | 0.06 | 2 | NA | -0.79 | -1.70 | 0.06 | 1 | Severe Imprecision |
| sofosbuvir + daclatasvir | sarilumab | NA | NA | NA | NA | NA | 0.06 | -0.96 | 0.91 | 2 | NA | 0.06 | -0.96 | 0.91 | 1 | Imprecision |
| sulodexide | sarilumab | NA | NA | NA | NA | NA | -0.43 | -2.15 | 1.04 | 1 | Intransitivity | -0.43 | -2.15 | 1.04 | 1 | Severe Imprecision |
| tocilizumab | sarilumab | 0.53 | -0.07 | 1.29 | 3 | RoB; | -0.32 | -0.93 | 0.24 | 2 | NA | 0.11 | -0.32 | 0.54 | 2 | Imprecision |
| tofacitinib | sarilumab | NA | NA | NA | NA | NA | -0.03 | -0.79 | 0.69 | 2 | NA | -0.03 | -0.79 | 0.69 | 1 | Imprecision |
| vitamin d3 | sarilumab | NA | NA | NA | NA | NA | -0.34 | -1.33 | 0.60 | 2 | NA | -0.34 | -1.33 | 0.60 | 1 | Imprecision |
| sulodexide | sofosbuvir + daclatasvir | NA | NA | NA | NA | NA | -0.47 | -2.31 | 1.20 | 3 | NA | -0.47 | -2.31 | 1.20 | 1 | Severe Imprecision |
| tocilizumab | sofosbuvir + daclatasvir | NA | NA | NA | NA | NA | 0.05 | -0.71 | 0.99 | 3 | NA | 0.05 | -0.71 | 0.99 | 2 | Imprecision |
| tofacitinib | sofosbuvir + daclatasvir | NA | NA | NA | NA | NA | -0.09 | -1.08 | 1.04 | 3 | NA | -0.09 | -1.08 | 1.04 | 2 | Imprecision |
| vitamin d3 | sofosbuvir + daclatasvir | NA | NA | NA | NA | NA | -0.39 | -1.57 | 0.84 | 3 | NA | -0.39 | -1.57 | 0.84 | 1 | Severe Imprecision |
| tocilizumab | sulodexide | NA | NA | NA | NA | NA | 0.54 | -0.87 | 2.22 | 3 | NA | 0.54 | -0.87 | 2.22 | 1 | Severe Imprecision |
| tofacitinib | sulodexide | NA | NA | NA | NA | NA | 0.40 | -1.17 | 2.19 | 3 | NA | 0.40 | -1.17 | 2.19 | 1 | Severe Imprecision |
| vitamin d3 | sulodexide | NA | NA | NA | NA | NA | 0.09 | -1.58 | 1.96 | 2 | Intransitivity | 0.09 | -1.58 | 1.96 | 1 | Severe Imprecision |
| tofacitinib | tocilizumab | NA | NA | NA | NA | NA | -0.14 | -0.83 | 0.50 | 3 | NA | -0.14 | -0.83 | 0.50 | 2 | Imprecision |
| vitamin d3 | tocilizumab | NA | NA | NA | NA | NA | -0.45 | -1.37 | 0.41 | 3 | NA | -0.45 | -1.37 | 0.41 | 2 | Imprecision |
| vitamin d3 | tofacitinib | NA | NA | NA | NA | NA | -0.31 | -1.42 | 0.77 | 4 | NA | -0.31 | -1.42 | 0.77 | 3 | Imprecision |

EST: estimate; LCrI: lower credible interval; UCrI: upper credible interval. ^†^Evidence: 4 (High), 3 (Moderate), 2 (Low), 1 (Very low).

## **Table S11. Network meta-analysis results of the primary analysis (log odds ratio, log OR and corresponding 95% equal-tailed Bayesian credible intervals) (discharge).**

|  |  | **Direct** | | | | | **Indirect** | | | | | **Network** | | | | |
| --- | --- | --- | --- | --- | --- | --- | --- | --- | --- | --- | --- | --- | --- | --- | --- | --- |
| **Treatment 1** | **Treatment 2** | **EST^*^** | **LCrI^*^** | **UCrI^*^** | **Evidence**^†^ | **Reason** | **EST** | **LCrI** | **UCrI** | **Evidence** | **Reason** | **EST** | **LCrI** | **UCrI** | **Evidence** | **Reason** |
| azithromycin | soc | 0.04 | -0.06 | 0.14 | 3 | RoB; | 0.03 | -0.44 | 0.47 | 3 | NA | 0.06 | -0.04 | 0.16 | 2 | Imprecision |
| bamlanivimab | soc | -0.24 | -0.97 | 0.47 | 4 | NA | NA | NA | NA | NA | NA | -0.24 | -0.97 | 0.47 | 3 | Imprecision |
| baricitinib + remdesivir | soc | NA | NA | NA | NA | NA | 0.53 | 0.22 | 0.85 | 4 | NA | 0.53 | 0.22 | 0.85 | 4 | NA |
| camostat mesilate | soc | 0.04 | -0.70 | 0.75 | 4 | NA | NA | NA | NA | NA | NA | 0.04 | -0.70 | 0.75 | 3 | Imprecision |
| canakinumab | soc | 0.29 | -0.03 | 0.57 | 4 | NA | NA | NA | NA | NA | NA | 0.29 | -0.03 | 0.57 | 3 | Imprecision |
| convalescent plasma | soc | 0.00 | -0.07 | 0.08 | 3 | RoB; | NA | NA | NA | NA | NA | 0.00 | -0.07 | 0.08 | 2 | Imprecision |
| dapagliflozin | soc | 0.19 | -0.17 | 0.57 | 4 | NA | NA | NA | NA | NA | NA | 0.19 | -0.17 | 0.57 | 3 | Imprecision |
| dexamethasone | soc | -0.01 | -0.12 | 0.10 | 3 | RoB; | NA | NA | NA | NA | NA | -0.01 | -0.12 | 0.10 | 2 | Imprecision |
| favipiravir | soc | 0.19 | -0.21 | 0.61 | 3 | RoB; | NA | NA | NA | NA | NA | 0.19 | -0.21 | 0.61 | 2 | Imprecision |
| hydroxychloroquine | soc | -0.31 | -0.42 | -0.21 | 3 | RoB; | -0.21 | -0.62 | 0.40 | 2 | NA | -0.29 | -0.39 | -0.19 | 3 | NA |
| hydroxychloroquine + azithromycin | soc | -0.23 | -0.67 | 0.21 | 3 | RoB; | NA | NA | NA | NA | NA | -0.23 | -0.67 | 0.21 | 2 | Imprecision |
| hydroxychloroquine + favipiravir | soc | -0.12 | -0.64 | 0.40 | 3 | RoB; | NA | NA | NA | NA | NA | -0.12 | -0.64 | 0.40 | 2 | Imprecision |
| interferon beta | soc | 0.05 | -0.27 | 0.36 | 2 | RoB;Inconsistency; | NA | NA | NA | NA | NA | 0.05 | -0.27 | 0.36 | 1 | Imprecision |
| ivermectin | soc | 0.31 | -0.49 | 1.14 | 4 | NA | NA | NA | NA | NA | NA | 0.31 | -0.49 | 1.14 | 3 | Imprecision |
| lopinavir/ritonavir | soc | 0.08 | -0.04 | 0.19 | 2 | RoB;Inconsistency; | NA | NA | NA | NA | NA | 0.08 | -0.04 | 0.19 | 1 | Imprecision |
| mesenchymal stem cells | soc | 0.08 | -0.32 | 0.63 | 3 | RoB; | NA | NA | NA | NA | NA | 0.08 | -0.32 | 0.63 | 2 | Imprecision |
| remdesivir | soc | 0.28 | 0.11 | 0.47 | 4 | NA | NA | NA | NA | NA | NA | 0.28 | 0.11 | 0.47 | 4 | NA |
| sarilumab | soc | 0.40 | 0.14 | 0.72 | 3 | RoB; | NA | NA | NA | NA | NA | 0.40 | 0.14 | 0.72 | 3 | NA |
| tocilizumab | soc | 0.30 | 0.19 | 0.40 | 3 | RoB; | NA | NA | NA | NA | NA | 0.30 | 0.19 | 0.40 | 3 | NA |
| tofacitinib | soc | 0.37 | 0.04 | 0.75 | 4 | NA | NA | NA | NA | NA | NA | 0.37 | 0.04 | 0.75 | 4 | NA |
| bamlanivimab | azithromycin | NA | NA | NA | NA | NA | -0.30 | -1.04 | 0.41 | 2 | Intransitivity | -0.30 | -1.04 | 0.41 | 1 | Imprecision |
| baricitinib + remdesivir | azithromycin | NA | NA | NA | NA | NA | 0.47 | 0.14 | 0.80 | 3 | NA | 0.47 | 0.14 | 0.80 | 3 | NA |
| camostat mesilate | azithromycin | NA | NA | NA | NA | NA | -0.02 | -0.77 | 0.70 | 2 | Intransitivity | -0.02 | -0.77 | 0.70 | 1 | Imprecision |
| canakinumab | azithromycin | NA | NA | NA | NA | NA | 0.22 | -0.10 | 0.53 | 3 | NA | 0.22 | -0.10 | 0.53 | 2 | Imprecision |
| convalescent plasma | azithromycin | NA | NA | NA | NA | NA | -0.06 | -0.18 | 0.06 | 3 | NA | -0.06 | -0.18 | 0.06 | 2 | Imprecision |
| dapagliflozin | azithromycin | NA | NA | NA | NA | NA | 0.13 | -0.25 | 0.52 | 3 | NA | 0.13 | -0.25 | 0.52 | 2 | Imprecision |
| dexamethasone | azithromycin | -0.08 | -0.21 | 0.04 | 3 | RoB; | 0.27 | -0.24 | 1.17 | 3 | NA | -0.07 | -0.19 | 0.05 | 2 | Imprecision |
| favipiravir | azithromycin | NA | NA | NA | NA | NA | 0.12 | -0.28 | 0.56 | 2 | Intransitivity | 0.12 | -0.28 | 0.56 | 1 | Imprecision |
| hydroxychloroquine | azithromycin | -0.40 | -0.53 | -0.27 | 3 | RoB; | -0.06 | -0.45 | 0.43 | 2 | Intransitivity | -0.35 | -0.48 | -0.23 | 3 | NA |
| hydroxychloroquine + azithromycin | azithromycin | NA | NA | NA | NA | NA | -0.30 | -0.74 | 0.16 | 2 | Intransitivity | -0.30 | -0.74 | 0.16 | 1 | Imprecision |
| hydroxychloroquine + favipiravir | azithromycin | NA | NA | NA | NA | NA | -0.18 | -0.71 | 0.35 | 2 | Intransitivity | -0.18 | -0.71 | 0.35 | 1 | Imprecision |
| interferon beta | azithromycin | NA | NA | NA | NA | NA | -0.01 | -0.34 | 0.31 | 2 | NA | -0.01 | -0.34 | 0.31 | 1 | Imprecision |
| ivermectin | azithromycin | NA | NA | NA | NA | NA | 0.25 | -0.56 | 1.09 | 2 | Intransitivity | 0.25 | -0.56 | 1.09 | 1 | Imprecision |
| lopinavir/ritonavir | azithromycin | 0.00 | -0.13 | 0.14 | 3 | RoB; | 0.21 | -0.17 | 0.66 | 2 | NA | 0.01 | -0.12 | 0.14 | 2 | Imprecision |
| mesenchymal stem cells | azithromycin | NA | NA | NA | NA | NA | 0.02 | -0.39 | 0.58 | 3 | NA | 0.02 | -0.39 | 0.58 | 2 | Imprecision |
| remdesivir | azithromycin | NA | NA | NA | NA | NA | 0.22 | 0.02 | 0.43 | 3 | NA | 0.22 | 0.02 | 0.43 | 3 | NA |
| sarilumab | azithromycin | NA | NA | NA | NA | NA | 0.34 | 0.06 | 0.67 | 3 | NA | 0.34 | 0.06 | 0.67 | 3 | NA |
| tocilizumab | azithromycin | NA | NA | NA | NA | NA | 0.23 | 0.09 | 0.38 | 3 | NA | 0.23 | 0.09 | 0.38 | 3 | NA |
| tofacitinib | azithromycin | NA | NA | NA | NA | NA | 0.31 | -0.04 | 0.70 | 3 | NA | 0.31 | -0.04 | 0.70 | 2 | Imprecision |
| baricitinib + remdesivir | bamlanivimab | NA | NA | NA | NA | NA | 0.77 | 0.00 | 1.57 | 4 | NA | 0.77 | 0.00 | 1.57 | 2 | Severe Imprecision |
| camostat mesilate | bamlanivimab | NA | NA | NA | NA | NA | 0.29 | -0.75 | 1.31 | 4 | NA | 0.29 | -0.75 | 1.31 | 3 | Imprecision |
| canakinumab | bamlanivimab | NA | NA | NA | NA | NA | 0.52 | -0.25 | 1.31 | 3 | Intransitivity | 0.52 | -0.25 | 1.31 | 2 | Imprecision |
| convalescent plasma | bamlanivimab | NA | NA | NA | NA | NA | 0.24 | -0.47 | 0.98 | 2 | Intransitivity | 0.24 | -0.47 | 0.98 | 1 | Imprecision |
| dapagliflozin | bamlanivimab | NA | NA | NA | NA | NA | 0.44 | -0.36 | 1.26 | 4 | NA | 0.44 | -0.36 | 1.26 | 3 | Imprecision |
| dexamethasone | bamlanivimab | NA | NA | NA | NA | NA | 0.23 | -0.49 | 0.97 | 2 | Intransitivity | 0.23 | -0.49 | 0.97 | 1 | Imprecision |
| favipiravir | bamlanivimab | NA | NA | NA | NA | NA | 0.43 | -0.38 | 1.27 | 3 | NA | 0.43 | -0.38 | 1.27 | 2 | Imprecision |
| hydroxychloroquine | bamlanivimab | NA | NA | NA | NA | NA | -0.05 | -0.77 | 0.69 | 3 | NA | -0.05 | -0.77 | 0.69 | 2 | Imprecision |
| hydroxychloroquine + azithromycin | bamlanivimab | NA | NA | NA | NA | NA | 0.01 | -0.82 | 0.86 | 3 | NA | 0.01 | -0.82 | 0.86 | 2 | Imprecision |
| hydroxychloroquine + favipiravir | bamlanivimab | NA | NA | NA | NA | NA | 0.12 | -0.76 | 1.02 | 3 | NA | 0.12 | -0.76 | 1.02 | 2 | Imprecision |
| interferon beta | bamlanivimab | NA | NA | NA | NA | NA | 0.29 | -0.49 | 1.09 | 1 | Intransitivity | 0.29 | -0.49 | 1.09 | 1 | Imprecision |
| ivermectin | bamlanivimab | NA | NA | NA | NA | NA | 0.56 | -0.52 | 1.65 | 4 | NA | 0.56 | -0.52 | 1.65 | 2 | Severe Imprecision |
| lopinavir/ritonavir | bamlanivimab | NA | NA | NA | NA | NA | 0.32 | -0.40 | 1.06 | 2 | NA | 0.32 | -0.40 | 1.06 | 1 | Imprecision |
| mesenchymal stem cells | bamlanivimab | NA | NA | NA | NA | NA | 0.34 | -0.49 | 1.24 | 2 | Intransitivity | 0.34 | -0.49 | 1.24 | 1 | Imprecision |
| remdesivir | bamlanivimab | NA | NA | NA | NA | NA | 0.53 | -0.20 | 1.28 | 4 | NA | 0.53 | -0.20 | 1.28 | 3 | Imprecision |
| sarilumab | bamlanivimab | NA | NA | NA | NA | NA | 0.65 | -0.11 | 1.44 | 2 | Intransitivity | 0.65 | -0.11 | 1.44 | 1 | Imprecision |
| tocilizumab | bamlanivimab | NA | NA | NA | NA | NA | 0.54 | -0.18 | 1.28 | 3 | NA | 0.54 | -0.18 | 1.28 | 2 | Imprecision |
| tofacitinib | bamlanivimab | NA | NA | NA | NA | NA | 0.61 | -0.17 | 1.43 | 4 | NA | 0.61 | -0.17 | 1.43 | 3 | Imprecision |
| camostat mesilate | baricitinib + remdesivir | NA | NA | NA | NA | NA | -0.49 | -1.30 | 0.29 | 4 | NA | -0.49 | -1.30 | 0.29 | 3 | Imprecision |
| canakinumab | baricitinib + remdesivir | NA | NA | NA | NA | NA | -0.24 | -0.70 | 0.18 | 4 | NA | -0.24 | -0.70 | 0.18 | 3 | Imprecision |
| convalescent plasma | baricitinib + remdesivir | NA | NA | NA | NA | NA | -0.53 | -0.85 | -0.21 | 3 | NA | -0.53 | -0.85 | -0.21 | 3 | NA |
| dapagliflozin | baricitinib + remdesivir | NA | NA | NA | NA | NA | -0.33 | -0.82 | 0.15 | 4 | NA | -0.33 | -0.82 | 0.15 | 3 | Imprecision |
| dexamethasone | baricitinib + remdesivir | NA | NA | NA | NA | NA | -0.54 | -0.87 | -0.21 | 3 | NA | -0.54 | -0.87 | -0.21 | 3 | NA |
| favipiravir | baricitinib + remdesivir | NA | NA | NA | NA | NA | -0.33 | -0.83 | 0.14 | 3 | NA | -0.33 | -0.83 | 0.14 | 2 | Imprecision |
| hydroxychloroquine | baricitinib + remdesivir | NA | NA | NA | NA | NA | -0.82 | -1.16 | -0.49 | 3 | NA | -0.82 | -1.16 | -0.49 | 3 | NA |
| hydroxychloroquine + azithromycin | baricitinib + remdesivir | NA | NA | NA | NA | NA | -0.76 | -1.30 | -0.22 | 3 | NA | -0.76 | -1.30 | -0.22 | 3 | NA |
| hydroxychloroquine + favipiravir | baricitinib + remdesivir | NA | NA | NA | NA | NA | -0.65 | -1.26 | -0.04 | 3 | NA | -0.65 | -1.26 | -0.04 | 3 | NA |
| interferon beta | baricitinib + remdesivir | NA | NA | NA | NA | NA | -0.48 | -0.93 | -0.04 | 2 | NA | -0.48 | -0.93 | -0.04 | 2 | NA |
| ivermectin | baricitinib + remdesivir | NA | NA | NA | NA | NA | -0.22 | -1.08 | 0.67 | 4 | NA | -0.22 | -1.08 | 0.67 | 3 | Imprecision |
| lopinavir/ritonavir | baricitinib + remdesivir | NA | NA | NA | NA | NA | -0.45 | -0.79 | -0.13 | 2 | NA | -0.45 | -0.79 | -0.13 | 2 | NA |
| mesenchymal stem cells | baricitinib + remdesivir | NA | NA | NA | NA | NA | -0.43 | -0.96 | 0.16 | 3 | NA | -0.43 | -0.96 | 0.16 | 2 | Imprecision |
| remdesivir | baricitinib + remdesivir | -0.24 | -0.50 | 0.01 | 4 | NA | NA | NA | NA | NA | NA | -0.24 | -0.50 | 0.01 | 3 | Imprecision |
| sarilumab | baricitinib + remdesivir | NA | NA | NA | NA | NA | -0.12 | -0.54 | 0.30 | 3 | NA | -0.12 | -0.54 | 0.30 | 2 | Imprecision |
| tocilizumab | baricitinib + remdesivir | NA | NA | NA | NA | NA | -0.23 | -0.57 | 0.10 | 3 | NA | -0.23 | -0.57 | 0.10 | 2 | Imprecision |
| tofacitinib | baricitinib + remdesivir | NA | NA | NA | NA | NA | -0.15 | -0.62 | 0.31 | 4 | NA | -0.15 | -0.62 | 0.31 | 3 | Imprecision |
| canakinumab | camostat mesilate | NA | NA | NA | NA | NA | 0.24 | -0.53 | 1.04 | 3 | Intransitivity | 0.24 | -0.53 | 1.04 | 2 | Imprecision |
| convalescent plasma | camostat mesilate | NA | NA | NA | NA | NA | -0.04 | -0.76 | 0.71 | 2 | Intransitivity | -0.04 | -0.76 | 0.71 | 1 | Imprecision |
| dapagliflozin | camostat mesilate | NA | NA | NA | NA | NA | 0.15 | -0.65 | 0.99 | 4 | NA | 0.15 | -0.65 | 0.99 | 3 | Imprecision |
| dexamethasone | camostat mesilate | NA | NA | NA | NA | NA | -0.05 | -0.77 | 0.70 | 2 | Intransitivity | -0.05 | -0.77 | 0.70 | 1 | Imprecision |
| favipiravir | camostat mesilate | NA | NA | NA | NA | NA | 0.15 | -0.66 | 0.99 | 3 | NA | 0.15 | -0.66 | 0.99 | 2 | Imprecision |
| hydroxychloroquine | camostat mesilate | NA | NA | NA | NA | NA | -0.34 | -1.06 | 0.42 | 3 | NA | -0.34 | -1.06 | 0.42 | 2 | Imprecision |
| hydroxychloroquine + azithromycin | camostat mesilate | NA | NA | NA | NA | NA | -0.28 | -1.11 | 0.58 | 3 | NA | -0.28 | -1.11 | 0.58 | 2 | Imprecision |
| hydroxychloroquine + favipiravir | camostat mesilate | NA | NA | NA | NA | NA | -0.16 | -1.05 | 0.75 | 3 | NA | -0.16 | -1.05 | 0.75 | 2 | Imprecision |
| interferon beta | camostat mesilate | NA | NA | NA | NA | NA | 0.00 | -0.77 | 0.81 | 1 | Intransitivity | 0.00 | -0.77 | 0.81 | 1 | Imprecision |
| ivermectin | camostat mesilate | NA | NA | NA | NA | NA | 0.27 | -0.81 | 1.39 | 4 | NA | 0.27 | -0.81 | 1.39 | 3 | Imprecision |
| lopinavir/ritonavir | camostat mesilate | NA | NA | NA | NA | NA | 0.03 | -0.69 | 0.79 | 2 | NA | 0.03 | -0.69 | 0.79 | 1 | Imprecision |
| mesenchymal stem cells | camostat mesilate | NA | NA | NA | NA | NA | 0.05 | -0.77 | 0.96 | 2 | Intransitivity | 0.05 | -0.77 | 0.96 | 1 | Imprecision |
| remdesivir | camostat mesilate | NA | NA | NA | NA | NA | 0.24 | -0.49 | 1.01 | 4 | NA | 0.24 | -0.49 | 1.01 | 3 | Imprecision |
| sarilumab | camostat mesilate | NA | NA | NA | NA | NA | 0.37 | -0.40 | 1.17 | 2 | Intransitivity | 0.37 | -0.40 | 1.17 | 1 | Imprecision |
| tocilizumab | camostat mesilate | NA | NA | NA | NA | NA | 0.25 | -0.46 | 1.01 | 3 | NA | 0.25 | -0.46 | 1.01 | 2 | Imprecision |
| tofacitinib | camostat mesilate | NA | NA | NA | NA | NA | 0.33 | -0.46 | 1.16 | 4 | NA | 0.33 | -0.46 | 1.16 | 3 | Imprecision |
| convalescent plasma | canakinumab | NA | NA | NA | NA | NA | -0.28 | -0.58 | 0.04 | 3 | NA | -0.28 | -0.58 | 0.04 | 2 | Imprecision |
| dapagliflozin | canakinumab | NA | NA | NA | NA | NA | -0.09 | -0.56 | 0.39 | 4 | NA | -0.09 | -0.56 | 0.39 | 3 | Imprecision |
| dexamethasone | canakinumab | NA | NA | NA | NA | NA | -0.30 | -0.60 | 0.03 | 3 | NA | -0.30 | -0.60 | 0.03 | 2 | Imprecision |
| favipiravir | canakinumab | NA | NA | NA | NA | NA | -0.10 | -0.57 | 0.44 | 2 | Intransitivity | -0.10 | -0.57 | 0.44 | 1 | Imprecision |
| hydroxychloroquine | canakinumab | NA | NA | NA | NA | NA | -0.58 | -0.88 | -0.25 | 2 | Intransitivity | -0.58 | -0.88 | -0.25 | 2 | NA |
| hydroxychloroquine + azithromycin | canakinumab | NA | NA | NA | NA | NA | -0.52 | -1.04 | 0.02 | 2 | Intransitivity | -0.52 | -1.04 | 0.02 | 1 | Imprecision |
| hydroxychloroquine + favipiravir | canakinumab | NA | NA | NA | NA | NA | -0.40 | -1.00 | 0.20 | 2 | Intransitivity | -0.40 | -1.00 | 0.20 | 1 | Imprecision |
| interferon beta | canakinumab | NA | NA | NA | NA | NA | -0.24 | -0.66 | 0.20 | 2 | NA | -0.24 | -0.66 | 0.20 | 1 | Imprecision |
| ivermectin | canakinumab | NA | NA | NA | NA | NA | 0.03 | -0.83 | 0.91 | 3 | Intransitivity | 0.03 | -0.83 | 0.91 | 2 | Imprecision |
| lopinavir/ritonavir | canakinumab | NA | NA | NA | NA | NA | -0.21 | -0.52 | 0.11 | 2 | NA | -0.21 | -0.52 | 0.11 | 1 | Imprecision |
| mesenchymal stem cells | canakinumab | NA | NA | NA | NA | NA | -0.20 | -0.69 | 0.46 | 3 | NA | -0.20 | -0.69 | 0.46 | 2 | Imprecision |
| remdesivir | canakinumab | NA | NA | NA | NA | NA | 0.00 | -0.33 | 0.38 | 4 | NA | 0.00 | -0.33 | 0.38 | 3 | Imprecision |
| sarilumab | canakinumab | NA | NA | NA | NA | NA | 0.11 | -0.22 | 0.55 | 3 | NA | 0.11 | -0.22 | 0.55 | 2 | Imprecision |
| tocilizumab | canakinumab | NA | NA | NA | NA | NA | 0.01 | -0.28 | 0.33 | 3 | NA | 0.01 | -0.28 | 0.33 | 2 | Imprecision |
| tofacitinib | canakinumab | NA | NA | NA | NA | NA | 0.08 | -0.29 | 0.55 | 4 | NA | 0.08 | -0.29 | 0.55 | 3 | Imprecision |
| dapagliflozin | convalescent plasma | NA | NA | NA | NA | NA | 0.19 | -0.18 | 0.57 | 3 | NA | 0.19 | -0.18 | 0.57 | 2 | Imprecision |
| dexamethasone | convalescent plasma | NA | NA | NA | NA | NA | -0.01 | -0.14 | 0.12 | 3 | NA | -0.01 | -0.14 | 0.12 | 2 | Imprecision |
| favipiravir | convalescent plasma | NA | NA | NA | NA | NA | 0.19 | -0.21 | 0.62 | 2 | Intransitivity | 0.19 | -0.21 | 0.62 | 1 | Imprecision |
| hydroxychloroquine | convalescent plasma | NA | NA | NA | NA | NA | -0.29 | -0.42 | -0.17 | 2 | Intransitivity | -0.29 | -0.42 | -0.17 | 2 | NA |
| hydroxychloroquine + azithromycin | convalescent plasma | NA | NA | NA | NA | NA | -0.23 | -0.68 | 0.22 | 2 | Intransitivity | -0.23 | -0.68 | 0.22 | 1 | Imprecision |
| hydroxychloroquine + favipiravir | convalescent plasma | NA | NA | NA | NA | NA | -0.12 | -0.65 | 0.41 | 2 | Intransitivity | -0.12 | -0.65 | 0.41 | 1 | Imprecision |
| interferon beta | convalescent plasma | NA | NA | NA | NA | NA | 0.05 | -0.28 | 0.37 | 2 | NA | 0.05 | -0.28 | 0.37 | 1 | Imprecision |
| ivermectin | convalescent plasma | NA | NA | NA | NA | NA | 0.31 | -0.50 | 1.14 | 2 | Intransitivity | 0.31 | -0.50 | 1.14 | 1 | Imprecision |
| lopinavir/ritonavir | convalescent plasma | NA | NA | NA | NA | NA | 0.07 | -0.06 | 0.21 | 2 | NA | 0.07 | -0.06 | 0.21 | 1 | Imprecision |
| mesenchymal stem cells | convalescent plasma | NA | NA | NA | NA | NA | 0.08 | -0.32 | 0.63 | 3 | NA | 0.08 | -0.32 | 0.63 | 2 | Imprecision |
| remdesivir | convalescent plasma | NA | NA | NA | NA | NA | 0.28 | 0.09 | 0.48 | 3 | NA | 0.28 | 0.09 | 0.48 | 3 | NA |
| sarilumab | convalescent plasma | NA | NA | NA | NA | NA | 0.40 | 0.12 | 0.72 | 3 | NA | 0.40 | 0.12 | 0.72 | 3 | NA |
| tocilizumab | convalescent plasma | NA | NA | NA | NA | NA | 0.30 | 0.17 | 0.42 | 3 | NA | 0.30 | 0.17 | 0.42 | 3 | NA |
| tofacitinib | convalescent plasma | NA | NA | NA | NA | NA | 0.37 | 0.03 | 0.76 | 3 | NA | 0.37 | 0.03 | 0.76 | 3 | NA |
| dexamethasone | dapagliflozin | NA | NA | NA | NA | NA | -0.20 | -0.59 | 0.18 | 3 | NA | -0.20 | -0.59 | 0.18 | 2 | Imprecision |
| favipiravir | dapagliflozin | NA | NA | NA | NA | NA | -0.01 | -0.55 | 0.55 | 3 | NA | -0.01 | -0.55 | 0.55 | 2 | Imprecision |
| hydroxychloroquine | dapagliflozin | NA | NA | NA | NA | NA | -0.49 | -0.87 | -0.10 | 3 | NA | -0.49 | -0.87 | -0.10 | 3 | NA |
| hydroxychloroquine + azithromycin | dapagliflozin | NA | NA | NA | NA | NA | -0.43 | -1.00 | 0.14 | 3 | NA | -0.43 | -1.00 | 0.14 | 2 | Imprecision |
| hydroxychloroquine + favipiravir | dapagliflozin | NA | NA | NA | NA | NA | -0.31 | -0.95 | 0.32 | 3 | NA | -0.31 | -0.95 | 0.32 | 2 | Imprecision |
| interferon beta | dapagliflozin | NA | NA | NA | NA | NA | -0.15 | -0.63 | 0.34 | 2 | NA | -0.15 | -0.63 | 0.34 | 1 | Imprecision |
| ivermectin | dapagliflozin | NA | NA | NA | NA | NA | 0.12 | -0.78 | 1.02 | 4 | NA | 0.12 | -0.78 | 1.02 | 3 | Imprecision |
| lopinavir/ritonavir | dapagliflozin | NA | NA | NA | NA | NA | -0.12 | -0.51 | 0.27 | 2 | NA | -0.12 | -0.51 | 0.27 | 1 | Imprecision |
| mesenchymal stem cells | dapagliflozin | NA | NA | NA | NA | NA | -0.10 | -0.66 | 0.54 | 3 | NA | -0.10 | -0.66 | 0.54 | 2 | Imprecision |
| remdesivir | dapagliflozin | NA | NA | NA | NA | NA | 0.09 | -0.32 | 0.50 | 4 | NA | 0.09 | -0.32 | 0.50 | 3 | Imprecision |
| sarilumab | dapagliflozin | NA | NA | NA | NA | NA | 0.21 | -0.25 | 0.69 | 3 | NA | 0.21 | -0.25 | 0.69 | 2 | Imprecision |
| tocilizumab | dapagliflozin | NA | NA | NA | NA | NA | 0.10 | -0.28 | 0.49 | 3 | NA | 0.10 | -0.28 | 0.49 | 2 | Imprecision |
| tofacitinib | dapagliflozin | NA | NA | NA | NA | NA | 0.18 | -0.32 | 0.70 | 4 | NA | 0.18 | -0.32 | 0.70 | 3 | Imprecision |
| favipiravir | dexamethasone | NA | NA | NA | NA | NA | 0.20 | -0.21 | 0.63 | 2 | Intransitivity | 0.20 | -0.21 | 0.63 | 1 | Imprecision |
| hydroxychloroquine | dexamethasone | -0.34 | -0.47 | -0.20 | 3 | RoB; | -0.48 | -1.10 | -0.03 | 2 | Intransitivity | -0.28 | -0.41 | -0.15 | 3 | NA |
| hydroxychloroquine + azithromycin | dexamethasone | NA | NA | NA | NA | NA | -0.22 | -0.67 | 0.23 | 2 | Intransitivity | -0.22 | -0.67 | 0.23 | 1 | Imprecision |
| hydroxychloroquine + favipiravir | dexamethasone | NA | NA | NA | NA | NA | -0.11 | -0.64 | 0.42 | 2 | Intransitivity | -0.11 | -0.64 | 0.42 | 1 | Imprecision |
| interferon beta | dexamethasone | NA | NA | NA | NA | NA | 0.06 | -0.27 | 0.38 | 2 | NA | 0.06 | -0.27 | 0.38 | 1 | Imprecision |
| ivermectin | dexamethasone | NA | NA | NA | NA | NA | 0.32 | -0.49 | 1.16 | 2 | Intransitivity | 0.32 | -0.49 | 1.16 | 1 | Imprecision |
| lopinavir/ritonavir | dexamethasone | 0.07 | -0.07 | 0.20 | 3 | RoB; | -0.12 | -0.81 | 0.30 | 2 | NA | 0.09 | -0.05 | 0.22 | 2 | Imprecision |
| mesenchymal stem cells | dexamethasone | NA | NA | NA | NA | NA | 0.09 | -0.32 | 0.66 | 3 | NA | 0.09 | -0.32 | 0.66 | 2 | Imprecision |
| remdesivir | dexamethasone | NA | NA | NA | NA | NA | 0.30 | 0.09 | 0.51 | 3 | NA | 0.30 | 0.09 | 0.51 | 3 | NA |
| sarilumab | dexamethasone | NA | NA | NA | NA | NA | 0.41 | 0.13 | 0.74 | 3 | NA | 0.41 | 0.13 | 0.74 | 3 | NA |
| tocilizumab | dexamethasone | NA | NA | NA | NA | NA | 0.31 | 0.16 | 0.45 | 3 | NA | 0.31 | 0.16 | 0.45 | 3 | NA |
| tofacitinib | dexamethasone | NA | NA | NA | NA | NA | 0.38 | 0.03 | 0.78 | 3 | NA | 0.38 | 0.03 | 0.78 | 3 | NA |
| hydroxychloroquine | favipiravir | NA | NA | NA | NA | NA | -0.48 | -0.92 | -0.08 | 3 | NA | -0.48 | -0.92 | -0.08 | 3 | NA |
| hydroxychloroquine + azithromycin | favipiravir | NA | NA | NA | NA | NA | -0.42 | -1.02 | 0.17 | 3 | NA | -0.42 | -1.02 | 0.17 | 2 | Imprecision |
| hydroxychloroquine + favipiravir | favipiravir | NA | NA | NA | NA | NA | -0.31 | -0.97 | 0.34 | 3 | NA | -0.31 | -0.97 | 0.34 | 2 | Imprecision |
| interferon beta | favipiravir | NA | NA | NA | NA | NA | -0.14 | -0.66 | 0.35 | 1 | Intransitivity | -0.14 | -0.66 | 0.35 | 1 | Imprecision |
| ivermectin | favipiravir | NA | NA | NA | NA | NA | 0.12 | -0.78 | 1.04 | 3 | NA | 0.12 | -0.78 | 1.04 | 2 | Imprecision |
| lopinavir/ritonavir | favipiravir | NA | NA | NA | NA | NA | -0.11 | -0.55 | 0.28 | 2 | NA | -0.11 | -0.55 | 0.28 | 1 | Imprecision |
| mesenchymal stem cells | favipiravir | NA | NA | NA | NA | NA | -0.11 | -0.67 | 0.58 | 2 | Intransitivity | -0.11 | -0.67 | 0.58 | 1 | Imprecision |
| remdesivir | favipiravir | NA | NA | NA | NA | NA | 0.09 | -0.31 | 0.52 | 3 | NA | 0.09 | -0.31 | 0.52 | 2 | Imprecision |
| sarilumab | favipiravir | NA | NA | NA | NA | NA | 0.21 | -0.26 | 0.73 | 2 | Intransitivity | 0.21 | -0.26 | 0.73 | 1 | Imprecision |
| tocilizumab | favipiravir | NA | NA | NA | NA | NA | 0.11 | -0.33 | 0.51 | 3 | NA | 0.11 | -0.33 | 0.51 | 2 | Imprecision |
| tofacitinib | favipiravir | NA | NA | NA | NA | NA | 0.18 | -0.35 | 0.74 | 3 | NA | 0.18 | -0.35 | 0.74 | 2 | Imprecision |
| hydroxychloroquine + azithromycin | hydroxychloroquine | 0.03 | -0.47 | 0.54 | 3 | RoB; | 0.05 | -0.59 | 0.70 | 3 | NA | 0.06 | -0.38 | 0.51 | 2 | Imprecision |
| hydroxychloroquine + favipiravir | hydroxychloroquine | NA | NA | NA | NA | NA | 0.17 | -0.36 | 0.70 | 3 | NA | 0.17 | -0.36 | 0.70 | 2 | Imprecision |
| interferon beta | hydroxychloroquine | 0.06 | -0.42 | 0.51 | 3 | RoB; | 0.73 | 0.21 | 1.30 | 1 | Intransitivity | 0.34 | 0.02 | 0.66 | 2 | Imprecision |
| ivermectin | hydroxychloroquine | NA | NA | NA | NA | NA | 0.60 | -0.21 | 1.44 | 3 | NA | 0.60 | -0.21 | 1.44 | 2 | Imprecision |
| lopinavir/ritonavir | hydroxychloroquine | 0.37 | 0.24 | 0.51 | 3 | RoB; | 0.50 | 0.17 | 0.95 | 2 | NA | 0.37 | 0.23 | 0.50 | 3 | NA |
| mesenchymal stem cells | hydroxychloroquine | NA | NA | NA | NA | NA | 0.38 | -0.04 | 0.94 | 2 | Intransitivity | 0.38 | -0.04 | 0.94 | 1 | Imprecision |
| remdesivir | hydroxychloroquine | NA | NA | NA | NA | NA | 0.58 | 0.37 | 0.79 | 3 | NA | 0.58 | 0.37 | 0.79 | 3 | NA |
| sarilumab | hydroxychloroquine | NA | NA | NA | NA | NA | 0.70 | 0.41 | 1.03 | 2 | Intransitivity | 0.70 | 0.41 | 1.03 | 2 | NA |
| tocilizumab | hydroxychloroquine | NA | NA | NA | NA | NA | 0.59 | 0.44 | 0.73 | 3 | NA | 0.59 | 0.44 | 0.73 | 3 | NA |
| tofacitinib | hydroxychloroquine | NA | NA | NA | NA | NA | 0.66 | 0.32 | 1.06 | 3 | NA | 0.66 | 0.32 | 1.06 | 3 | NA |
| hydroxychloroquine + favipiravir | hydroxychloroquine + azithromycin | NA | NA | NA | NA | NA | 0.11 | -0.57 | 0.80 | 3 | NA | 0.11 | -0.57 | 0.80 | 2 | Imprecision |
| interferon beta | hydroxychloroquine + azithromycin | NA | NA | NA | NA | NA | 0.28 | -0.26 | 0.82 | 1 | Intransitivity | 0.28 | -0.26 | 0.82 | 1 | Imprecision |
| ivermectin | hydroxychloroquine + azithromycin | NA | NA | NA | NA | NA | 0.54 | -0.37 | 1.48 | 3 | NA | 0.54 | -0.37 | 1.48 | 2 | Imprecision |
| lopinavir/ritonavir | hydroxychloroquine + azithromycin | NA | NA | NA | NA | NA | 0.31 | -0.15 | 0.76 | 2 | NA | 0.31 | -0.15 | 0.76 | 1 | Imprecision |
| mesenchymal stem cells | hydroxychloroquine + azithromycin | NA | NA | NA | NA | NA | 0.33 | -0.28 | 1.00 | 2 | Intransitivity | 0.33 | -0.28 | 1.00 | 1 | Imprecision |
| remdesivir | hydroxychloroquine + azithromycin | NA | NA | NA | NA | NA | 0.52 | 0.04 | 0.99 | 3 | NA | 0.52 | 0.04 | 0.99 | 3 | NA |
| sarilumab | hydroxychloroquine + azithromycin | NA | NA | NA | NA | NA | 0.64 | 0.12 | 1.17 | 2 | Intransitivity | 0.64 | 0.12 | 1.17 | 2 | NA |
| tocilizumab | hydroxychloroquine + azithromycin | NA | NA | NA | NA | NA | 0.53 | 0.07 | 0.98 | 3 | NA | 0.53 | 0.07 | 0.98 | 3 | NA |
| tofacitinib | hydroxychloroquine + azithromycin | NA | NA | NA | NA | NA | 0.61 | 0.05 | 1.18 | 3 | NA | 0.61 | 0.05 | 1.18 | 3 | NA |
| interferon beta | hydroxychloroquine + favipiravir | NA | NA | NA | NA | NA | 0.17 | -0.44 | 0.77 | 1 | Intransitivity | 0.17 | -0.44 | 0.77 | 1 | Imprecision |
| ivermectin | hydroxychloroquine + favipiravir | NA | NA | NA | NA | NA | 0.43 | -0.53 | 1.41 | 3 | NA | 0.43 | -0.53 | 1.41 | 2 | Imprecision |
| lopinavir/ritonavir | hydroxychloroquine + favipiravir | NA | NA | NA | NA | NA | 0.19 | -0.34 | 0.73 | 2 | NA | 0.19 | -0.34 | 0.73 | 1 | Imprecision |
| mesenchymal stem cells | hydroxychloroquine + favipiravir | NA | NA | NA | NA | NA | 0.21 | -0.45 | 0.95 | 2 | Intransitivity | 0.21 | -0.45 | 0.95 | 1 | Imprecision |
| remdesivir | hydroxychloroquine + favipiravir | NA | NA | NA | NA | NA | 0.40 | -0.15 | 0.96 | 3 | NA | 0.40 | -0.15 | 0.96 | 2 | Imprecision |
| sarilumab | hydroxychloroquine + favipiravir | NA | NA | NA | NA | NA | 0.53 | -0.06 | 1.13 | 2 | Intransitivity | 0.53 | -0.06 | 1.13 | 1 | Imprecision |
| tocilizumab | hydroxychloroquine + favipiravir | NA | NA | NA | NA | NA | 0.42 | -0.11 | 0.95 | 3 | NA | 0.42 | -0.11 | 0.95 | 2 | Imprecision |
| tofacitinib | hydroxychloroquine + favipiravir | NA | NA | NA | NA | NA | 0.49 | -0.13 | 1.13 | 3 | NA | 0.49 | -0.13 | 1.13 | 2 | Imprecision |
| ivermectin | interferon beta | NA | NA | NA | NA | NA | 0.26 | -0.60 | 1.15 | 1 | Intransitivity | 0.26 | -0.60 | 1.15 | 1 | Imprecision |
| lopinavir/ritonavir | interferon beta | -0.07 | -0.47 | 0.36 | 3 | RoB; | -0.40 | -0.89 | 0.05 | 2 | NA | 0.03 | -0.29 | 0.35 | 2 | Imprecision |
| mesenchymal stem cells | interferon beta | NA | NA | NA | NA | NA | 0.04 | -0.47 | 0.66 | 2 | NA | 0.04 | -0.47 | 0.66 | 1 | Imprecision |
| remdesivir | interferon beta | NA | NA | NA | NA | NA | 0.24 | -0.12 | 0.60 | 2 | NA | 0.24 | -0.12 | 0.60 | 1 | Imprecision |
| sarilumab | interferon beta | NA | NA | NA | NA | NA | 0.36 | -0.06 | 0.80 | 2 | NA | 0.36 | -0.06 | 0.80 | 1 | Imprecision |
| tocilizumab | interferon beta | NA | NA | NA | NA | NA | 0.25 | -0.08 | 0.58 | 2 | NA | 0.25 | -0.08 | 0.58 | 1 | Imprecision |
| tofacitinib | interferon beta | NA | NA | NA | NA | NA | 0.32 | -0.13 | 0.81 | 2 | NA | 0.32 | -0.13 | 0.81 | 1 | Imprecision |
| lopinavir/ritonavir | ivermectin | NA | NA | NA | NA | NA | -0.24 | -1.07 | 0.58 | 2 | NA | -0.24 | -1.07 | 0.58 | 1 | Imprecision |
| mesenchymal stem cells | ivermectin | NA | NA | NA | NA | NA | -0.22 | -1.15 | 0.74 | 2 | Intransitivity | -0.22 | -1.15 | 0.74 | 1 | Imprecision |
| remdesivir | ivermectin | NA | NA | NA | NA | NA | -0.02 | -0.87 | 0.80 | 4 | NA | -0.02 | -0.87 | 0.80 | 3 | Imprecision |
| sarilumab | ivermectin | NA | NA | NA | NA | NA | 0.10 | -0.78 | 0.96 | 2 | Intransitivity | 0.10 | -0.78 | 0.96 | 1 | Imprecision |
| tocilizumab | ivermectin | NA | NA | NA | NA | NA | -0.01 | -0.85 | 0.80 | 3 | NA | -0.01 | -0.85 | 0.80 | 2 | Imprecision |
| tofacitinib | ivermectin | NA | NA | NA | NA | NA | 0.06 | -0.83 | 0.95 | 4 | NA | 0.06 | -0.83 | 0.95 | 3 | Imprecision |
| mesenchymal stem cells | lopinavir/ritonavir | NA | NA | NA | NA | NA | 0.01 | -0.40 | 0.58 | 2 | NA | 0.01 | -0.40 | 0.58 | 1 | Imprecision |
| remdesivir | lopinavir/ritonavir | NA | NA | NA | NA | NA | 0.21 | 0.01 | 0.43 | 2 | NA | 0.21 | 0.01 | 0.43 | 1 | Imprecision |
| sarilumab | lopinavir/ritonavir | NA | NA | NA | NA | NA | 0.33 | 0.04 | 0.67 | 2 | NA | 0.33 | 0.04 | 0.67 | 2 | NA |
| tocilizumab | lopinavir/ritonavir | NA | NA | NA | NA | NA | 0.22 | 0.07 | 0.37 | 2 | NA | 0.22 | 0.07 | 0.37 | 2 | NA |
| tofacitinib | lopinavir/ritonavir | NA | NA | NA | NA | NA | 0.29 | -0.05 | 0.70 | 2 | NA | 0.29 | -0.05 | 0.70 | 1 | Imprecision |
| remdesivir | mesenchymal stem cells | NA | NA | NA | NA | NA | 0.19 | -0.35 | 0.65 | 3 | NA | 0.19 | -0.35 | 0.65 | 2 | Imprecision |
| sarilumab | mesenchymal stem cells | NA | NA | NA | NA | NA | 0.31 | -0.26 | 0.83 | 3 | NA | 0.31 | -0.26 | 0.83 | 2 | Imprecision |
| tocilizumab | mesenchymal stem cells | NA | NA | NA | NA | NA | 0.21 | -0.35 | 0.63 | 3 | NA | 0.21 | -0.35 | 0.63 | 2 | Imprecision |
| tofacitinib | mesenchymal stem cells | NA | NA | NA | NA | NA | 0.28 | -0.34 | 0.83 | 3 | NA | 0.28 | -0.34 | 0.83 | 2 | Imprecision |
| sarilumab | remdesivir | NA | NA | NA | NA | NA | 0.12 | -0.20 | 0.46 | 3 | NA | 0.12 | -0.20 | 0.46 | 2 | Imprecision |
| tocilizumab | remdesivir | NA | NA | NA | NA | NA | 0.01 | -0.20 | 0.22 | 3 | NA | 0.01 | -0.20 | 0.22 | 2 | Imprecision |
| tofacitinib | remdesivir | NA | NA | NA | NA | NA | 0.09 | -0.30 | 0.48 | 4 | NA | 0.09 | -0.30 | 0.48 | 3 | Imprecision |
| tocilizumab | sarilumab | -0.10 | -0.54 | 0.17 | 3 | RoB; | 0.00 | -0.34 | 0.33 | 3 | NA | -0.10 | -0.43 | 0.16 | 2 | Imprecision |
| tofacitinib | sarilumab | NA | NA | NA | NA | NA | -0.03 | -0.44 | 0.36 | 3 | NA | -0.03 | -0.44 | 0.36 | 2 | Imprecision |
| tofacitinib | tocilizumab | NA | NA | NA | NA | NA | 0.07 | -0.26 | 0.46 | 3 | NA | 0.07 | -0.26 | 0.46 | 2 | Imprecision |

EST: estimate; LCrI: lower credible interval; UCrI: upper credible interval. ^†^Evidence: 4 (High), 3 (Moderate), 2 (Low), 1 (Very low).

## **Table S12. Network meta-analysis results of the primary analysis (log odds ratio, log OR and corresponding 95% equal-tailed Bayesian credible intervals) (viral clearance).**

|  |  | **Direct** | | | | | **Indirect** | | | | | **Network** | | | | |
| --- | --- | --- | --- | --- | --- | --- | --- | --- | --- | --- | --- | --- | --- | --- | --- | --- |
| **Treatment 1** | **Treatment 2** | **EST^*^** | **LCrI^*^** | **UCrI^*^** | **Evidence**^†^ | **Reason** | **EST** | **LCrI** | **UCrI** | **Evidence** | **Reason** | **EST** | **LCrI** | **UCrI** | **Evidence** | **Reason** |
| bamlanivimab | soc | -0.03 | -0.63 | 0.62 | 4 | NA | NA | NA | NA | NA | NA | -0.03 | -0.63 | 0.62 | 3 | Imprecision |
| bamlanivimab + etesevimab | soc | 0.10 | -0.57 | 0.83 | 4 | NA | NA | NA | NA | NA | NA | 0.10 | -0.57 | 0.83 | 3 | Imprecision |
| convalescent plasma | soc | 0.48 | 0.16 | 0.80 | 2 | RoB;Inconsistency; | NA | NA | NA | NA | NA | 0.48 | 0.16 | 0.80 | 2 | NA |
| favipiravir | soc | 0.31 | -0.07 | 0.74 | 3 | RoB; | 0.07 | -0.55 | 0.58 | 2 | NA | 0.26 | -0.10 | 0.63 | 2 | Imprecision |
| hydroxychloroquine | soc | 0.26 | 0.03 | 0.48 | 2 | RoB;Inconsistency; | 0.52 | -0.02 | 1.25 | 3 | NA | 0.27 | 0.05 | 0.48 | 3 | NA |
| hydroxychloroquine + azithromycin | soc | 0.03 | -0.45 | 0.51 | 4 | NA | NA | NA | NA | NA | NA | 0.03 | -0.45 | 0.51 | 3 | Imprecision |
| hydroxychloroquine + favipiravir | soc | 0.14 | -0.49 | 0.77 | 3 | RoB; | NA | NA | NA | NA | NA | 0.14 | -0.49 | 0.77 | 2 | Imprecision |
| ivermectin | soc | 0.74 | 0.20 | 1.30 | 3 | Inconsistency; | 0.89 | 0.15 | 1.76 | 4 | NA | 0.84 | 0.32 | 1.37 | 4 | NA |
| ivermectin + doxycycline | soc | 0.93 | 0.39 | 1.50 | 4 | NA | NA | NA | NA | NA | NA | 0.93 | 0.39 | 1.50 | 4 | NA |
| lopinavir/ritonavir | soc | 0.20 | -0.22 | 0.63 | 3 | RoB; | 0.13 | -0.64 | 0.73 | 2 | NA | 0.23 | -0.17 | 0.62 | 2 | Imprecision |
| methylprednisolone | soc | 0.02 | -0.52 | 0.57 | 3 | RoB; | NA | NA | NA | NA | NA | 0.02 | -0.52 | 0.57 | 2 | Imprecision |
| nitazoxanide | soc | 0.54 | 0.18 | 1.00 | 3 | RoB; | NA | NA | NA | NA | NA | 0.54 | 0.18 | 1.00 | 3 | NA |
| proxalutamide | soc | 2.34 | 1.70 | 3.01 | 3 | RoB; | NA | NA | NA | NA | NA | 2.34 | 1.70 | 3.01 | 3 | NA |
| remdesivir | soc | 0.16 | -0.31 | 0.58 | 4 | NA | NA | NA | NA | NA | NA | 0.16 | -0.31 | 0.58 | 3 | Imprecision |
| bamlanivimab + etesevimab | bamlanivimab | 0.11 | -0.31 | 0.66 | 4 | NA | NA | NA | NA | NA | NA | 0.11 | -0.31 | 0.66 | 3 | Imprecision |
| convalescent plasma | bamlanivimab | NA | NA | NA | NA | NA | 0.51 | -0.21 | 1.20 | 2 | NA | 0.51 | -0.21 | 1.20 | 1 | Imprecision |
| favipiravir | bamlanivimab | NA | NA | NA | NA | NA | 0.29 | -0.45 | 1.00 | 3 | NA | 0.29 | -0.45 | 1.00 | 2 | Imprecision |
| hydroxychloroquine | bamlanivimab | NA | NA | NA | NA | NA | 0.30 | -0.38 | 0.94 | 2 | NA | 0.30 | -0.38 | 0.94 | 1 | Imprecision |
| hydroxychloroquine + azithromycin | bamlanivimab | NA | NA | NA | NA | NA | 0.06 | -0.74 | 0.83 | 4 | NA | 0.06 | -0.74 | 0.83 | 3 | Imprecision |
| hydroxychloroquine + favipiravir | bamlanivimab | NA | NA | NA | NA | NA | 0.17 | -0.73 | 1.05 | 3 | NA | 0.17 | -0.73 | 1.05 | 2 | Imprecision |
| ivermectin | bamlanivimab | NA | NA | NA | NA | NA | 0.87 | 0.05 | 1.67 | 3 | NA | 0.87 | 0.05 | 1.67 | 3 | NA |
| ivermectin + doxycycline | bamlanivimab | NA | NA | NA | NA | NA | 0.96 | 0.12 | 1.80 | 4 | NA | 0.96 | 0.12 | 1.80 | 4 | NA |
| lopinavir/ritonavir | bamlanivimab | NA | NA | NA | NA | NA | 0.26 | -0.50 | 0.98 | 3 | NA | 0.26 | -0.50 | 0.98 | 2 | Imprecision |
| methylprednisolone | bamlanivimab | NA | NA | NA | NA | NA | 0.05 | -0.80 | 0.86 | 3 | NA | 0.05 | -0.80 | 0.86 | 2 | Imprecision |
| nitazoxanide | bamlanivimab | NA | NA | NA | NA | NA | 0.58 | -0.18 | 1.35 | 3 | NA | 0.58 | -0.18 | 1.35 | 2 | Imprecision |
| proxalutamide | bamlanivimab | NA | NA | NA | NA | NA | 2.37 | 1.45 | 3.27 | 3 | NA | 2.37 | 1.45 | 3.27 | 3 | NA |
| remdesivir | bamlanivimab | NA | NA | NA | NA | NA | 0.19 | -0.60 | 0.93 | 3 | Intransitivity | 0.19 | -0.60 | 0.93 | 2 | Imprecision |
| convalescent plasma | bamlanivimab + etesevimab | NA | NA | NA | NA | NA | 0.38 | -0.41 | 1.13 | 2 | NA | 0.38 | -0.41 | 1.13 | 1 | Imprecision |
| favipiravir | bamlanivimab + etesevimab | NA | NA | NA | NA | NA | 0.16 | -0.65 | 0.92 | 3 | NA | 0.16 | -0.65 | 0.92 | 2 | Imprecision |
| hydroxychloroquine | bamlanivimab + etesevimab | NA | NA | NA | NA | NA | 0.17 | -0.60 | 0.87 | 2 | NA | 0.17 | -0.60 | 0.87 | 1 | Imprecision |
| hydroxychloroquine + azithromycin | bamlanivimab + etesevimab | NA | NA | NA | NA | NA | -0.07 | -0.94 | 0.76 | 4 | NA | -0.07 | -0.94 | 0.76 | 3 | Imprecision |
| hydroxychloroquine + favipiravir | bamlanivimab + etesevimab | NA | NA | NA | NA | NA | 0.04 | -0.91 | 0.96 | 3 | NA | 0.04 | -0.91 | 0.96 | 2 | Imprecision |
| ivermectin | bamlanivimab + etesevimab | NA | NA | NA | NA | NA | 0.74 | -0.15 | 1.59 | 3 | NA | 0.74 | -0.15 | 1.59 | 1 | Severe Imprecision |
| ivermectin + doxycycline | bamlanivimab + etesevimab | NA | NA | NA | NA | NA | 0.83 | -0.07 | 1.71 | 4 | NA | 0.83 | -0.07 | 1.71 | 2 | Severe Imprecision |
| lopinavir/ritonavir | bamlanivimab + etesevimab | NA | NA | NA | NA | NA | 0.13 | -0.70 | 0.90 | 3 | NA | 0.13 | -0.70 | 0.90 | 2 | Imprecision |
| methylprednisolone | bamlanivimab + etesevimab | NA | NA | NA | NA | NA | -0.08 | -0.99 | 0.79 | 3 | NA | -0.08 | -0.99 | 0.79 | 2 | Imprecision |
| nitazoxanide | bamlanivimab + etesevimab | NA | NA | NA | NA | NA | 0.45 | -0.35 | 1.24 | 3 | NA | 0.45 | -0.35 | 1.24 | 2 | Imprecision |
| proxalutamide | bamlanivimab + etesevimab | NA | NA | NA | NA | NA | 2.23 | 1.27 | 3.19 | 3 | NA | 2.23 | 1.27 | 3.19 | 3 | NA |
| remdesivir | bamlanivimab + etesevimab | NA | NA | NA | NA | NA | 0.06 | -0.82 | 0.86 | 3 | Intransitivity | 0.06 | -0.82 | 0.86 | 2 | Imprecision |
| favipiravir | convalescent plasma | NA | NA | NA | NA | NA | -0.22 | -0.70 | 0.27 | 2 | NA | -0.22 | -0.70 | 0.27 | 1 | Imprecision |
| hydroxychloroquine | convalescent plasma | NA | NA | NA | NA | NA | -0.22 | -0.60 | 0.17 | 2 | NA | -0.22 | -0.60 | 0.17 | 1 | Imprecision |
| hydroxychloroquine + azithromycin | convalescent plasma | NA | NA | NA | NA | NA | -0.45 | -1.03 | 0.12 | 2 | NA | -0.45 | -1.03 | 0.12 | 1 | Imprecision |
| hydroxychloroquine + favipiravir | convalescent plasma | NA | NA | NA | NA | NA | -0.34 | -1.05 | 0.36 | 2 | NA | -0.34 | -1.05 | 0.36 | 1 | Imprecision |
| ivermectin | convalescent plasma | NA | NA | NA | NA | NA | 0.36 | -0.25 | 0.98 | 2 | NA | 0.36 | -0.25 | 0.98 | 1 | Imprecision |
| ivermectin + doxycycline | convalescent plasma | NA | NA | NA | NA | NA | 0.45 | -0.18 | 1.10 | 2 | NA | 0.45 | -0.18 | 1.10 | 1 | Imprecision |
| lopinavir/ritonavir | convalescent plasma | NA | NA | NA | NA | NA | -0.26 | -0.76 | 0.25 | 2 | NA | -0.26 | -0.76 | 0.25 | 1 | Imprecision |
| methylprednisolone | convalescent plasma | NA | NA | NA | NA | NA | -0.46 | -1.09 | 0.17 | 2 | NA | -0.46 | -1.09 | 0.17 | 1 | Imprecision |
| nitazoxanide | convalescent plasma | NA | NA | NA | NA | NA | 0.07 | -0.43 | 0.62 | 2 | NA | 0.07 | -0.43 | 0.62 | 1 | Imprecision |
| proxalutamide | convalescent plasma | NA | NA | NA | NA | NA | 1.85 | 1.14 | 2.60 | 2 | NA | 1.85 | 1.14 | 2.60 | 2 | NA |
| remdesivir | convalescent plasma | NA | NA | NA | NA | NA | -0.32 | -0.89 | 0.21 | 2 | NA | -0.32 | -0.89 | 0.21 | 1 | Imprecision |
| hydroxychloroquine | favipiravir | 0.19 | -0.32 | 0.76 | 3 | RoB; | -0.06 | -0.55 | 0.37 | 2 | NA | 0.01 | -0.40 | 0.40 | 2 | Imprecision |
| hydroxychloroquine + azithromycin | favipiravir | NA | NA | NA | NA | NA | -0.23 | -0.82 | 0.35 | 3 | NA | -0.23 | -0.82 | 0.35 | 2 | Imprecision |
| hydroxychloroquine + favipiravir | favipiravir | NA | NA | NA | NA | NA | -0.12 | -0.85 | 0.60 | 3 | NA | -0.12 | -0.85 | 0.60 | 2 | Imprecision |
| ivermectin | favipiravir | NA | NA | NA | NA | NA | 0.58 | -0.04 | 1.20 | 3 | NA | 0.58 | -0.04 | 1.20 | 2 | Imprecision |
| ivermectin + doxycycline | favipiravir | NA | NA | NA | NA | NA | 0.67 | 0.01 | 1.35 | 3 | NA | 0.67 | 0.01 | 1.35 | 2 | Imprecision |
| lopinavir/ritonavir | favipiravir | NA | NA | NA | NA | NA | -0.03 | -0.50 | 0.41 | 3 | NA | -0.03 | -0.50 | 0.41 | 2 | Imprecision |
| methylprednisolone | favipiravir | NA | NA | NA | NA | NA | -0.24 | -0.90 | 0.42 | 3 | NA | -0.24 | -0.90 | 0.42 | 2 | Imprecision |
| nitazoxanide | favipiravir | NA | NA | NA | NA | NA | 0.28 | -0.21 | 0.86 | 3 | NA | 0.28 | -0.21 | 0.86 | 2 | Imprecision |
| proxalutamide | favipiravir | NA | NA | NA | NA | NA | 2.08 | 1.34 | 2.84 | 3 | NA | 2.08 | 1.34 | 2.84 | 3 | NA |
| remdesivir | favipiravir | NA | NA | NA | NA | NA | -0.09 | -0.64 | 0.35 | 2 | Intransitivity | -0.09 | -0.64 | 0.35 | 1 | Imprecision |
| hydroxychloroquine + azithromycin | hydroxychloroquine | -0.23 | -0.71 | 0.24 | 4 | NA | NA | NA | NA | NA | NA | -0.23 | -0.71 | 0.24 | 3 | Imprecision |
| hydroxychloroquine + favipiravir | hydroxychloroquine | NA | NA | NA | NA | NA | -0.13 | -0.79 | 0.55 | 2 | NA | -0.13 | -0.79 | 0.55 | 1 | Imprecision |
| ivermectin | hydroxychloroquine | NA | NA | NA | NA | NA | 0.57 | 0.02 | 1.14 | 2 | NA | 0.57 | 0.02 | 1.14 | 1 | Imprecision |
| ivermectin + doxycycline | hydroxychloroquine | NA | NA | NA | NA | NA | 0.67 | 0.08 | 1.27 | 2 | NA | 0.67 | 0.08 | 1.27 | 2 | NA |
| lopinavir/ritonavir | hydroxychloroquine | 0.00 | -0.48 | 0.57 | 3 | RoB; | -0.15 | -0.79 | 0.37 | 2 | NA | -0.04 | -0.46 | 0.39 | 2 | Imprecision |
| methylprednisolone | hydroxychloroquine | NA | NA | NA | NA | NA | -0.25 | -0.83 | 0.34 | 2 | NA | -0.25 | -0.83 | 0.34 | 1 | Imprecision |
| nitazoxanide | hydroxychloroquine | NA | NA | NA | NA | NA | 0.27 | -0.09 | 0.78 | 2 | NA | 0.27 | -0.09 | 0.78 | 1 | Imprecision |
| proxalutamide | hydroxychloroquine | NA | NA | NA | NA | NA | 2.07 | 1.39 | 2.78 | 2 | NA | 2.07 | 1.39 | 2.78 | 2 | NA |
| remdesivir | hydroxychloroquine | NA | NA | NA | NA | NA | -0.11 | -0.59 | 0.36 | 1 | Intransitivity | -0.11 | -0.59 | 0.36 | 1 | Imprecision |
| hydroxychloroquine + favipiravir | hydroxychloroquine + azithromycin | NA | NA | NA | NA | NA | 0.11 | -0.68 | 0.90 | 3 | NA | 0.11 | -0.68 | 0.90 | 2 | Imprecision |
| ivermectin | hydroxychloroquine + azithromycin | NA | NA | NA | NA | NA | 0.81 | 0.11 | 1.52 | 3 | NA | 0.81 | 0.11 | 1.52 | 3 | NA |
| ivermectin + doxycycline | hydroxychloroquine + azithromycin | NA | NA | NA | NA | NA | 0.90 | 0.18 | 1.64 | 4 | NA | 0.90 | 0.18 | 1.64 | 4 | NA |
| lopinavir/ritonavir | hydroxychloroquine + azithromycin | NA | NA | NA | NA | NA | 0.19 | -0.41 | 0.80 | 3 | NA | 0.19 | -0.41 | 0.80 | 2 | Imprecision |
| methylprednisolone | hydroxychloroquine + azithromycin | NA | NA | NA | NA | NA | -0.01 | -0.73 | 0.71 | 3 | NA | -0.01 | -0.73 | 0.71 | 2 | Imprecision |
| nitazoxanide | hydroxychloroquine + azithromycin | NA | NA | NA | NA | NA | 0.52 | -0.08 | 1.17 | 3 | NA | 0.52 | -0.08 | 1.17 | 2 | Imprecision |
| proxalutamide | hydroxychloroquine + azithromycin | NA | NA | NA | NA | NA | 2.31 | 1.50 | 3.13 | 3 | NA | 2.31 | 1.50 | 3.13 | 3 | NA |
| remdesivir | hydroxychloroquine + azithromycin | NA | NA | NA | NA | NA | 0.13 | -0.52 | 0.76 | 3 | Intransitivity | 0.13 | -0.52 | 0.76 | 2 | Imprecision |
| ivermectin | hydroxychloroquine + favipiravir | NA | NA | NA | NA | NA | 0.70 | -0.12 | 1.52 | 3 | NA | 0.70 | -0.12 | 1.52 | 1 | Severe Imprecision |
| ivermectin + doxycycline | hydroxychloroquine + favipiravir | NA | NA | NA | NA | NA | 0.79 | -0.04 | 1.64 | 3 | NA | 0.79 | -0.04 | 1.64 | 1 | Severe Imprecision |
| lopinavir/ritonavir | hydroxychloroquine + favipiravir | NA | NA | NA | NA | NA | 0.08 | -0.66 | 0.83 | 3 | NA | 0.08 | -0.66 | 0.83 | 2 | Imprecision |
| methylprednisolone | hydroxychloroquine + favipiravir | NA | NA | NA | NA | NA | -0.12 | -0.95 | 0.71 | 3 | NA | -0.12 | -0.95 | 0.71 | 2 | Imprecision |
| nitazoxanide | hydroxychloroquine + favipiravir | NA | NA | NA | NA | NA | 0.41 | -0.33 | 1.18 | 3 | NA | 0.41 | -0.33 | 1.18 | 2 | Imprecision |
| proxalutamide | hydroxychloroquine + favipiravir | NA | NA | NA | NA | NA | 2.20 | 1.30 | 3.11 | 3 | NA | 2.20 | 1.30 | 3.11 | 3 | NA |
| remdesivir | hydroxychloroquine + favipiravir | NA | NA | NA | NA | NA | 0.02 | -0.76 | 0.78 | 2 | Intransitivity | 0.02 | -0.76 | 0.78 | 1 | Imprecision |
| ivermectin + doxycycline | ivermectin | -0.15 | -1.21 | 0.72 | 4 | NA | 0.20 | -0.57 | 1.00 | 3 | NA | 0.09 | -0.61 | 0.80 | 3 | Imprecision |
| lopinavir/ritonavir | ivermectin | -0.70 | -1.47 | 0.01 | 4 | NA | -0.57 | -1.20 | 0.09 | 3 | NA | -0.62 | -1.22 | -0.01 | 3 | Imprecision |
| methylprednisolone | ivermectin | NA | NA | NA | NA | NA | -0.82 | -1.58 | -0.07 | 3 | NA | -0.82 | -1.58 | -0.07 | 3 | NA |
| nitazoxanide | ivermectin | NA | NA | NA | NA | NA | -0.29 | -0.94 | 0.39 | 3 | NA | -0.29 | -0.94 | 0.39 | 2 | Imprecision |
| proxalutamide | ivermectin | NA | NA | NA | NA | NA | 1.50 | 0.67 | 2.34 | 3 | NA | 1.50 | 0.67 | 2.34 | 3 | NA |
| remdesivir | ivermectin | NA | NA | NA | NA | NA | -0.68 | -1.36 | -0.03 | 2 | Intransitivity | -0.68 | -1.36 | -0.03 | 2 | NA |
| lopinavir/ritonavir | ivermectin + doxycycline | NA | NA | NA | NA | NA | -0.71 | -1.39 | -0.04 | 3 | NA | -0.71 | -1.39 | -0.04 | 3 | NA |
| methylprednisolone | ivermectin + doxycycline | NA | NA | NA | NA | NA | -0.91 | -1.70 | -0.14 | 3 | NA | -0.91 | -1.70 | -0.14 | 3 | NA |
| nitazoxanide | ivermectin + doxycycline | NA | NA | NA | NA | NA | -0.38 | -1.06 | 0.32 | 3 | NA | -0.38 | -1.06 | 0.32 | 2 | Imprecision |
| proxalutamide | ivermectin + doxycycline | NA | NA | NA | NA | NA | 1.40 | 0.54 | 2.27 | 3 | NA | 1.40 | 0.54 | 2.27 | 3 | NA |
| remdesivir | ivermectin + doxycycline | NA | NA | NA | NA | NA | -0.77 | -1.51 | -0.08 | 3 | Intransitivity | -0.77 | -1.51 | -0.08 | 3 | NA |
| methylprednisolone | lopinavir/ritonavir | NA | NA | NA | NA | NA | -0.21 | -0.88 | 0.47 | 3 | NA | -0.21 | -0.88 | 0.47 | 2 | Imprecision |
| nitazoxanide | lopinavir/ritonavir | NA | NA | NA | NA | NA | 0.32 | -0.20 | 0.93 | 3 | NA | 0.32 | -0.20 | 0.93 | 2 | Imprecision |
| proxalutamide | lopinavir/ritonavir | NA | NA | NA | NA | NA | 2.11 | 1.36 | 2.89 | 3 | NA | 2.11 | 1.36 | 2.89 | 3 | NA |
| remdesivir | lopinavir/ritonavir | NA | NA | NA | NA | NA | -0.06 | -0.61 | 0.41 | 2 | Intransitivity | -0.06 | -0.61 | 0.41 | 1 | Imprecision |
| nitazoxanide | methylprednisolone | NA | NA | NA | NA | NA | 0.53 | -0.14 | 1.23 | 3 | NA | 0.53 | -0.14 | 1.23 | 2 | Imprecision |
| proxalutamide | methylprednisolone | NA | NA | NA | NA | NA | 2.32 | 1.47 | 3.18 | 3 | NA | 2.32 | 1.47 | 3.18 | 3 | NA |
| remdesivir | methylprednisolone | NA | NA | NA | NA | NA | 0.14 | -0.58 | 0.83 | 3 | NA | 0.14 | -0.58 | 0.83 | 2 | Imprecision |
| proxalutamide | nitazoxanide | NA | NA | NA | NA | NA | 1.78 | 1.00 | 2.56 | 3 | NA | 1.78 | 1.00 | 2.56 | 3 | NA |
| remdesivir | nitazoxanide | NA | NA | NA | NA | NA | -0.38 | -1.08 | 0.17 | 2 | Intransitivity | -0.38 | -1.08 | 0.17 | 1 | Imprecision |
| remdesivir | proxalutamide | NA | NA | NA | NA | NA | -2.18 | -3.00 | -1.41 | 2 | Intransitivity | -2.18 | -3.00 | -1.41 | 2 | NA |

EST: estimate; LCrI: lower credible interval; UCrI: upper credible interval. ^†^Evidence: 4 (High), 3 (Moderate), 2 (Low), 1 (Very low).

## **Table S13. Sensitivity analysis: fixed-effects model versus fixed-effects model which treated trials with multiple publications as multiple separated trials versus random-effects model (mortality).**

|  | **Fixed-effects** | | **Random-effects** | | **Fixed-effects**  **(seperated)** | |
| --- | --- | --- | --- | --- | --- | --- |
| **Treatment** | **OR [95% CrI]** | **Posterior Probability Favouring Treatment** | **OR [95% CrI]** | **Posterior Probability Favouring Treatment** | **OR [95% CrI]** | **Posterior Probability Favouring Treatment** |
| Agents Acting On The Renin-Angiotensin System | 1.25 [0.63,2.49] | 0.251 | 1.24 [0.62,2.51] | 0.263 | 1.25 [0.63,2.47] | 0.246 |
| Aceis/Arbs | 1.25 [0.75,2.08] | 0.197 | 1.24 [0.71,2.18] | 0.221 | 1.25 [0.76,2.07] | 0.193 |
| Anthelmintics | 0.61 [0.31,1.21] | 0.929 | 0.58 [0.29,1.16] | 0.941 | 0.60 [0.30,1.19] | 0.934 |
| Ivermectin | 0.61 [0.37,1.00] | 0.975 | 0.58 [0.34,1.00] | 0.974 | 0.60 [0.36,0.99] | 0.977 |
| Antibacterials For Systemic Use | 0.94 [0.62,1.52] | 0.636 | 0.86 [0.53,1.41] | 0.759 | 1.02 [0.69,1.79] | 0.461 |
| Azithromycin | 0.93 [0.83,1.03] | 0.922 | 0.84 [0.60,1.11] | 0.892 | 0.97 [0.87,1.08] | 0.731 |
| Doxycycline | 0.96 [0.59,1.75] | 0.571 | 0.88 [0.51,1.61] | 0.697 | 1.07 [0.67,2.42] | 0.381 |
| Antigout Preparations | 0.48 [0.15,1.42] | 0.909 | 0.46 [0.14,1.40] | 0.915 | 0.47 [0.15,1.32] | 0.924 |
| Colchicine | 0.48 [0.16,1.27] | 0.930 | 0.46 [0.15,1.29] | 0.930 | 0.47 [0.17,1.19] | 0.943 |
| Antihemorrhagics | 1.03 [0.28,4.32] | 0.482 | 1.04 [0.26,4.79] | 0.481 | 1.02 [0.27,4.31] | 0.489 |
| Camostat Mesilate | 1.03 [0.30,4.02] | 0.481 | 1.03 [0.28,4.52] | 0.481 | 1.02 [0.30,4.00] | 0.490 |
| Antineoplastic Agents | 0.58 [0.34,0.98] | 0.978 | 0.58 [0.32,1.01] | 0.973 | 0.57 [0.33,0.98] | 0.978 |
| Bamlanivimab | 0.60 [0.34,1.09] | 0.956 | 0.59 [0.32,1.08] | 0.957 | 0.60 [0.34,1.09] | 0.956 |
| Imatinib | 0.55 [0.33,0.91] | 0.990 | 0.56 [0.31,0.98] | 0.979 | 0.55 [0.32,0.91] | 0.990 |
| Inm005 | 0.57 [0.32,1.01] | 0.973 | 0.57 [0.31,1.05] | 0.966 | 0.57 [0.32,1.01] | 0.973 |
| Antiprotozoals | 1.15 [0.75,1.70] | 0.207 | 1.07 [0.71,1.62] | 0.349 | 1.12 [0.73,1.66] | 0.251 |
| Chloroquine | 1.13 [0.73,1.67] | 0.269 | 1.06 [0.67,1.63] | 0.387 | 1.10 [0.71,1.63] | 0.312 |
| Hydroxychloroquine | 1.17 [1.05,1.29] | 0.001 | 1.09 [0.87,1.34] | 0.220 | 1.13 [1.01,1.27] | 0.015 |
| Antiprotozoals + Antivirals For Systemic Use | 1.37 [0.71,2.61] | 0.169 | 1.40 [0.70,2.80] | 0.166 | 1.42 [0.74,2.73] | 0.139 |
| Hydroxychloroquine + Favipiravir | 1.27 [0.67,2.35] | 0.225 | 1.33 [0.66,2.60] | 0.208 | 1.31 [0.69,2.43] | 0.195 |
| Hydroxychloroquine + Lopinavir/Ritonavir | 1.47 [0.79,2.78] | 0.112 | 1.48 [0.76,2.94] | 0.124 | 1.54 [0.83,2.93] | 0.087 |
| Antiprotozoals + Antibacterials For Systemic Use | 0.54 [0.13,1.82] | 0.836 | 0.50 [0.12,1.70] | 0.861 | 0.52 [0.13,1.76] | 0.851 |
| Hydroxychloroquine + Azithromycin | 0.54 [0.14,1.65] | 0.851 | 0.51 [0.13,1.59] | 0.875 | 0.52 [0.14,1.61] | 0.867 |
| Antithrombotic Agents | 0.90 [0.52,1.34] | 0.723 | 0.92 [0.52,1.50] | 0.644 | 0.96 [0.55,1.44] | 0.584 |
| Sulodexide | 0.85 [0.40,1.37] | 0.759 | 0.87 [0.42,1.51] | 0.688 | 0.91 [0.41,1.45] | 0.658 |
| Therapeutic Anticoagulation | 0.95 [0.80,1.12] | 0.732 | 0.96 [0.66,1.39] | 0.585 | 1.02 [0.85,1.22] | 0.424 |
| Antivirals For Systemic Use | 0.94 [0.67,1.24] | 0.689 | 0.86 [0.61,1.17] | 0.846 | 0.96 [0.68,1.25] | 0.652 |
| Favipiravir | 0.97 [0.67,1.42] | 0.573 | 0.88 [0.57,1.33] | 0.758 | 0.99 [0.68,1.45] | 0.535 |
| Lopinavir/Ritonavir | 0.98 [0.89,1.09] | 0.620 | 0.90 [0.71,1.11] | 0.831 | 1.02 [0.92,1.14] | 0.331 |
| Remdesivir | 0.95 [0.84,1.08] | 0.767 | 0.87 [0.67,1.11] | 0.874 | 0.93 [0.81,1.07] | 0.834 |
| Sofosbuvir + Daclatasvir | 0.87 [0.45,1.27] | 0.766 | 0.81 [0.45,1.21] | 0.853 | 0.89 [0.46,1.28] | 0.745 |
| Blood Substitutes And Perfusion Solutions | 0.92 [0.58,1.30] | 0.712 | 0.83 [0.53,1.23] | 0.841 | 0.92 [0.58,1.30] | 0.711 |
| Ammonium Chloride | 1.04 [0.68,2.07] | 0.428 | 0.93 [0.59,1.85] | 0.613 | 1.04 [0.68,2.11] | 0.429 |
| Convalescent Plasma | 0.97 [0.89,1.05] | 0.794 | 0.86 [0.66,1.08] | 0.899 | 0.97 [0.89,1.05] | 0.795 |
| Mesenchymal Stem Cells | 0.77 [0.31,1.15] | 0.885 | 0.72 [0.32,1.11] | 0.929 | 0.77 [0.30,1.15] | 0.884 |
| Corticosteroids, Dermatological Preparations | 0.90 [0.67,1.22] | 0.800 | 0.85 [0.62,1.19] | 0.852 | 0.82 [0.60,1.10] | 0.926 |
| Budesonide | 0.95 [0.63,1.69] | 0.597 | 0.89 [0.58,1.57] | 0.696 | 0.81 [0.50,1.28] | 0.854 |
| Dexamethasone | 0.97 [0.87,1.08] | 0.697 | 0.88 [0.68,1.14] | 0.843 | 0.85 [0.76,0.95] | 0.997 |
| Hydrocortisone | 0.80 [0.62,1.01] | 0.970 | 0.81 [0.59,1.09] | 0.921 | 0.80 [0.58,1.06] | 0.941 |
| Methylprednisolone | 0.87 [0.65,1.15] | 0.842 | 0.83 [0.58,1.16] | 0.874 | 0.83 [0.62,1.09] | 0.916 |
| Drugs Used In Diabetes | 0.74 [0.39,1.39] | 0.845 | 0.74 [0.35,1.56] | 0.800 | 0.74 [0.39,1.39] | 0.845 |
| Dapagliflozin | 0.74 [0.48,1.13] | 0.918 | 0.74 [0.40,1.37] | 0.841 | 0.74 [0.48,1.13] | 0.918 |
| Immune Sera And Immunoglobulins | 0.48 [0.22,1.03] | 0.970 | 0.46 [0.21,1.02] | 0.973 | 0.48 [0.22,1.05] | 0.968 |
| Intravenous Immunoglobulin | 0.48 [0.26,0.89] | 0.991 | 0.46 [0.23,0.90] | 0.989 | 0.48 [0.26,0.89] | 0.990 |
| Immunostimulants | 0.88 [0.47,1.26] | 0.755 | 0.69 [0.36,1.09] | 0.943 | 0.87 [0.46,1.26] | 0.764 |
| Interferon Beta | 1.00 [0.86,1.16] | 0.500 | 0.76 [0.47,1.06] | 0.943 | 0.99 [0.82,1.18] | 0.559 |
| Recombinant Human Gcsf | 0.78 [0.30,1.20] | 0.851 | 0.63 [0.28,1.06] | 0.957 | 0.77 [0.30,1.20] | 0.853 |
| Immunosuppressants | 0.79 [0.53,1.06] | 0.950 | 0.83 [0.56,1.16] | 0.868 | 0.79 [0.53,1.06] | 0.949 |
| Canakinumab | 0.77 [0.46,1.14] | 0.915 | 0.82 [0.48,1.24] | 0.841 | 0.77 [0.46,1.14] | 0.916 |
| Sarilumab | 0.77 [0.52,1.07] | 0.945 | 0.81 [0.52,1.16] | 0.878 | 0.77 [0.52,1.07] | 0.942 |
| Tocilizumab | 0.85 [0.77,0.95] | 0.998 | 0.91 [0.74,1.16] | 0.791 | 0.85 [0.77,0.95] | 0.998 |
| Tofacitinib | 0.76 [0.41,1.16] | 0.913 | 0.81 [0.44,1.25] | 0.842 | 0.76 [0.41,1.16] | 0.912 |
| Immunosuppressants + Antivirals For Systemic Use | 0.60 [0.27,1.25] | 0.922 | 0.55 [0.22,1.28] | 0.924 | 0.60 [0.27,1.26] | 0.918 |
| Baricitinib + Remdesivir | 0.61 [0.35,1.03] | 0.967 | 0.55 [0.26,1.13] | 0.949 | 0.59 [0.34,1.02] | 0.971 |
| Vitamins | 0.74 [0.33,1.65] | 0.776 | 0.71 [0.30,1.60] | 0.801 | 0.74 [0.33,1.66] | 0.776 |
| Vitamin C | 0.76 [0.33,1.83] | 0.736 | 0.73 [0.30,1.75] | 0.769 | 0.76 [0.33,1.84] | 0.738 |
| Vitamin D3 | 0.71 [0.34,1.45] | 0.825 | 0.68 [0.31,1.44] | 0.840 | 0.72 [0.34,1.45] | 0.825 |

OR: odds ratio; CrI: equal-tailed credible interval.

## **Table S14. Sensitivity analysis: fixed-effects model versus fixed-effects model which treated trials with multiple publications as multiple separated trials versus random-effects model (mechanical ventilation).**

|  | **Fixed-effects** | | **Random-effects** | | **Fixed-effects**  **(seperated)** | |
| --- | --- | --- | --- | --- | --- | --- |
| **Treatment** | **OR [95% CrI]** | **Posterior Probability Favouring Treatment** | **OR [95% CrI]** | **Posterior Probability Favouring Treatment** | **OR [95% CrI]** | **Posterior Probability Favouring Treatment** |
| Agents Acting On The Renin-Angiotensin System | 0.88 [0.32,2.36] | 0.609 | 0.89 [0.39,2.04] | 0.615 | 0.88 [0.38,2.04] | 0.630 |
| Aceis/Arbs | 0.88 [0.58,1.32] | 0.733 | 0.89 [0.52,1.55] | 0.660 | 0.88 [0.58,1.32] | 0.734 |
| Anthelmintics | 0.92 [0.31,2.75] | 0.567 | 0.90 [0.36,2.32] | 0.588 | 1.02 [0.40,2.61] | 0.484 |
| Ivermectin | 0.92 [0.49,1.71] | 0.605 | 0.91 [0.45,1.82] | 0.609 | 1.02 [0.57,1.82] | 0.473 |
| Antibacterials For Systemic Use | 0.70 [0.31,1.42] | 0.847 | 0.77 [0.38,1.38] | 0.817 | 0.87 [0.42,1.65] | 0.674 |
| Azithromycin | 0.93 [0.80,1.08] | 0.840 | 0.89 [0.60,1.26] | 0.742 | 0.96 [0.83,1.12] | 0.692 |
| Doxycycline | 0.53 [0.21,1.08] | 0.959 | 0.68 [0.28,1.26] | 0.887 | 0.79 [0.32,1.67] | 0.735 |
| Antigout Preparations | 0.42 [0.13,1.31] | 0.937 | 0.40 [0.14,1.12] | 0.961 | 0.43 [0.15,1.14] | 0.957 |
| Colchicine | 0.42 [0.20,0.83] | 0.994 | 0.40 [0.17,0.91] | 0.986 | 0.43 [0.20,0.82] | 0.995 |
| Antihemorrhagics | 1.06 [0.20,6.72] | 0.473 | 1.03 [0.20,6.40] | 0.485 | 1.05 [0.22,6.21] | 0.478 |
| Camostat Mesilate | 1.05 [0.26,5.40] | 0.472 | 1.03 [0.22,5.79] | 0.484 | 1.04 [0.26,5.38] | 0.479 |
| Antineoplastic Agents | 0.96 [0.48,1.91] | 0.553 | 0.95 [0.49,1.88] | 0.557 | 0.95 [0.51,1.78] | 0.563 |
| Bamlanivimab | 1.05 [0.48,2.38] | 0.447 | 1.00 [0.48,2.18] | 0.502 | 1.02 [0.51,2.17] | 0.474 |
| Imatinib | 1.06 [0.64,1.79] | 0.410 | 1.00 [0.53,1.90] | 0.500 | 1.04 [0.64,1.73] | 0.438 |
| Inm005 | 0.78 [0.39,1.49] | 0.771 | 0.87 [0.42,1.74] | 0.657 | 0.82 [0.42,1.50] | 0.744 |
| Antiprotozoals | 1.11 [0.56,2.27] | 0.378 | 1.12 [0.65,2.03] | 0.329 | 1.25 [0.70,2.31] | 0.206 |
| Chloroquine | 1.26 [0.73,2.21] | 0.202 | 1.21 [0.72,2.19] | 0.236 | 1.37 [0.83,2.33] | 0.110 |
| Hydroxychloroquine | 0.97 [0.85,1.12] | 0.643 | 1.03 [0.79,1.38] | 0.403 | 1.14 [0.98,1.34] | 0.047 |
| Antiprotozoals + Antivirals For Systemic Use | 1.10 [0.35,3.42] | 0.431 | 1.10 [0.37,3.30] | 0.431 | 1.10 [0.40,3.00] | 0.424 |
| Hydroxychloroquine + Favipiravir | 1.10 [0.55,2.19] | 0.392 | 1.10 [0.45,2.74] | 0.418 | 1.10 [0.55,2.19] | 0.395 |
| Antiprotozoals + Antibacterials For Systemic Use | 1.64 [0.55,4.87] | 0.173 | 1.73 [0.64,4.64] | 0.133 | 1.76 [0.67,4.57] | 0.114 |
| Hydroxychloroquine + Azithromycin | 1.64 [0.89,3.03] | 0.057 | 1.73 [0.79,3.76] | 0.082 | 1.77 [0.95,3.25] | 0.035 |
| Antithrombotic Agents | 0.43 [0.07,2.30] | 0.837 | 0.44 [0.07,2.28] | 0.834 | 0.44 [0.07,2.17] | 0.843 |
| Sulodexide | 0.44 [0.08,1.77] | 0.876 | 0.44 [0.08,2.02] | 0.852 | 0.44 [0.08,1.82] | 0.870 |
| Antivirals For Systemic Use | 0.95 [0.55,1.56] | 0.590 | 0.89 [0.57,1.37] | 0.724 | 0.99 [0.62,1.51] | 0.525 |
| Favipiravir | 1.15 [0.72,1.86] | 0.280 | 0.97 [0.60,1.67] | 0.549 | 1.19 [0.77,1.91] | 0.217 |
| Lopinavir/Ritonavir | 0.95 [0.82,1.08] | 0.786 | 0.89 [0.64,1.20] | 0.783 | 1.05 [0.90,1.23] | 0.250 |
| Remdesivir | 1.05 [0.92,1.19] | 0.253 | 0.93 [0.67,1.25] | 0.692 | 0.95 [0.83,1.09] | 0.768 |
| Sofosbuvir + Daclatasvir | 0.71 [0.28,1.49] | 0.813 | 0.79 [0.35,1.40] | 0.798 | 0.80 [0.34,1.51] | 0.753 |
| Blood Substitutes And Perfusion Solutions | 0.87 [0.41,1.77] | 0.661 | 0.89 [0.48,1.60] | 0.658 | 0.89 [0.47,1.61] | 0.670 |
| Ammonium Chloride | 0.78 [0.39,1.50] | 0.775 | 0.84 [0.43,1.52] | 0.718 | 0.81 [0.42,1.46] | 0.761 |
| Convalescent Plasma | 0.97 [0.87,1.08] | 0.718 | 0.95 [0.66,1.36] | 0.615 | 0.97 [0.87,1.08] | 0.720 |
| Corticosteroids, Dermatological Preparations | 0.85 [0.48,1.56] | 0.719 | 0.92 [0.56,1.57] | 0.631 | 0.83 [0.50,1.39] | 0.780 |
| Budesonide | 1.20 [0.66,2.23] | 0.277 | 1.08 [0.62,2.15] | 0.397 | 0.87 [0.50,1.56] | 0.688 |
| Dexamethasone | 0.66 [0.55,0.79] | 1.000 | 0.82 [0.58,1.25] | 0.828 | 0.81 [0.67,0.99] | 0.982 |
| Methylprednisolone | 0.78 [0.48,1.24] | 0.857 | 0.86 [0.52,1.42] | 0.722 | 0.81 [0.52,1.27] | 0.825 |
| Cough And Cold Preparations | 0.38 [0.09,1.45] | 0.924 | 0.38 [0.10,1.32] | 0.937 | 0.39 [0.10,1.33] | 0.935 |
| Bromhexine | 0.38 [0.12,1.02] | 0.972 | 0.38 [0.12,1.11] | 0.961 | 0.39 [0.13,1.04] | 0.969 |
| Immune Sera And Immunoglobulins | 0.71 [0.25,2.03] | 0.756 | 0.69 [0.28,1.67] | 0.808 | 0.71 [0.28,1.76] | 0.786 |
| Intravenous Immunoglobulin | 0.71 [0.41,1.21] | 0.897 | 0.69 [0.36,1.29] | 0.877 | 0.71 [0.41,1.21] | 0.895 |
| Immunostimulants | 0.51 [0.23,0.97] | 0.980 | 0.54 [0.27,0.93] | 0.986 | 0.52 [0.26,0.89] | 0.990 |
| Interferon Beta | 0.97 [0.82,1.14] | 0.632 | 0.74 [0.46,1.08] | 0.936 | 0.90 [0.74,1.09] | 0.867 |
| Recombinant Human Gcsf | 0.25 [0.13,0.48] | 1.000 | 0.40 [0.16,0.78] | 0.998 | 0.29 [0.14,0.57] | 1.000 |
| Immunosuppressants | 0.67 [0.39,1.12] | 0.941 | 0.66 [0.41,1.03] | 0.967 | 0.68 [0.42,1.05] | 0.961 |
| Canakinumab | 0.62 [0.33,1.12] | 0.944 | 0.65 [0.35,1.12] | 0.944 | 0.64 [0.35,1.09] | 0.952 |
| Sarilumab | 0.67 [0.44,1.01] | 0.972 | 0.65 [0.40,1.02] | 0.971 | 0.67 [0.45,1.00] | 0.976 |
| Tocilizumab | 0.75 [0.65,0.86] | 1.000 | 0.72 [0.53,0.95] | 0.988 | 0.74 [0.65,0.86] | 1.000 |
| Tofacitinib | 0.65 [0.33,1.22] | 0.912 | 0.66 [0.35,1.17] | 0.932 | 0.66 [0.35,1.18] | 0.926 |
| Immunosuppressants + Antivirals For Systemic Use | 0.58 [0.19,1.75] | 0.848 | 0.55 [0.19,1.54] | 0.883 | 0.61 [0.23,1.55] | 0.868 |
| Baricitinib + Remdesivir | 0.64 [0.42,0.98] | 0.980 | 0.57 [0.25,1.23] | 0.928 | 0.59 [0.38,0.89] | 0.994 |
| Vitamins | 0.47 [0.13,1.62] | 0.888 | 0.48 [0.14,1.59] | 0.888 | 0.48 [0.15,1.46] | 0.906 |
| Vitamin D3 | 0.47 [0.19,1.10] | 0.958 | 0.48 [0.16,1.34] | 0.920 | 0.48 [0.19,1.11] | 0.957 |

OR: odds ratio; CrI: equal-tailed credible interval.

## **Table S15. Sensitivity analysis: fixed-effects model versus fixed-effects model which treated trials with multiple publications as multiple separated trials versus random-effects model (discharge).**

|  | **Fixed-effects** | | **Random-effects** | | **Fixed-effects**  **(seperated)** | |
| --- | --- | --- | --- | --- | --- | --- |
| **Treatment** | **OR [95% CrI]** | **Posterior Probability Favouring Treatment** | **OR [95% CrI]** | **Posterior Probability Favouring Treatment** | **OR [95% CrI]** | **Posterior Probability Favouring Treatment** |
| Anthelmintics | 1.36 [0.55,3.43] | 0.753 | 1.38 [0.54,3.56] | 0.752 | 1.35 [0.54,3.43] | 0.743 |
| Ivermectin | 1.36 [0.61,3.12] | 0.774 | 1.37 [0.59,3.29] | 0.769 | 1.35 [0.60,3.09] | 0.767 |
| Antibacterials For Systemic Use | 1.07 [0.69,1.62] | 0.652 | 1.08 [0.67,1.69] | 0.643 | 1.04 [0.66,1.64] | 0.589 |
| Azithromycin | 1.06 [0.96,1.17] | 0.892 | 1.07 [0.83,1.38] | 0.729 | 1.04 [0.94,1.15] | 0.779 |
| Antihemorrhagics | 1.04 [0.45,2.36] | 0.542 | 1.05 [0.42,2.49] | 0.541 | 1.05 [0.44,2.42] | 0.542 |
| Camostat Mesilate | 1.05 [0.49,2.12] | 0.548 | 1.05 [0.46,2.28] | 0.546 | 1.05 [0.50,2.15] | 0.550 |
| Antineoplastic Agents | 0.79 [0.34,1.78] | 0.275 | 0.78 [0.32,1.88] | 0.288 | 0.78 [0.34,1.79] | 0.275 |
| Bamlanivimab | 0.79 [0.38,1.60] | 0.252 | 0.78 [0.35,1.72] | 0.270 | 0.78 [0.38,1.59] | 0.248 |
| Antiprotozoals | 0.75 [0.49,1.14] | 0.064 | 0.83 [0.53,1.28] | 0.158 | 0.88 [0.56,1.40] | 0.248 |
| Hydroxychloroquine | 0.75 [0.67,0.83] | 0.000 | 0.82 [0.69,1.02] | 0.037 | 0.88 [0.80,0.98] | 0.012 |
| Antiprotozoals + Antivirals For Systemic Use | 0.89 [0.46,1.71] | 0.356 | 0.89 [0.43,1.83] | 0.367 | 0.89 [0.45,1.75] | 0.356 |
| Hydroxychloroquine + Favipiravir | 0.89 [0.53,1.49] | 0.328 | 0.89 [0.48,1.63] | 0.348 | 0.89 [0.53,1.50] | 0.325 |
| Antiprotozoals + Antibacterials For Systemic Use | 0.79 [0.44,1.44] | 0.209 | 0.83 [0.44,1.56] | 0.268 | 0.85 [0.46,1.59] | 0.296 |
| Hydroxychloroquine + Azithromycin | 0.79 [0.51,1.23] | 0.150 | 0.83 [0.50,1.37] | 0.227 | 0.85 [0.55,1.33] | 0.241 |
| Antivirals For Systemic Use | 1.20 [0.91,1.62] | 0.922 | 1.24 [0.92,1.69] | 0.931 | 1.16 [0.87,1.61] | 0.874 |
| Favipiravir | 1.20 [0.81,1.84] | 0.851 | 1.24 [0.82,1.89] | 0.875 | 1.18 [0.78,1.85] | 0.807 |
| Lopinavir/Ritonavir | 1.08 [0.96,1.20] | 0.908 | 1.17 [0.95,1.47] | 0.929 | 1.01 [0.90,1.14] | 0.583 |
| Remdesivir | 1.33 [1.11,1.60] | 0.999 | 1.31 [1.03,1.67] | 0.986 | 1.33 [1.10,1.60] | 0.999 |
| Blood Substitutes And Perfusion Solutions | 1.04 [0.74,1.59] | 0.608 | 1.10 [0.76,1.71] | 0.706 | 1.05 [0.73,1.64] | 0.614 |
| Convalescent Plasma | 1.00 [0.93,1.08] | 0.509 | 1.06 [0.86,1.36] | 0.711 | 1.00 [0.93,1.08] | 0.506 |
| Mesenchymal Stem Cells | 1.09 [0.73,1.89] | 0.664 | 1.14 [0.74,1.96] | 0.732 | 1.10 [0.72,1.96] | 0.676 |
| Corticosteroids, Dermatological Preparations | 0.99 [0.65,1.51] | 0.477 | 1.09 [0.68,1.78] | 0.664 | 1.19 [0.75,1.89] | 0.825 |
| Dexamethasone | 0.99 [0.89,1.10] | 0.423 | 1.08 [0.84,1.49] | 0.739 | 1.19 [1.07,1.33] | 0.999 |
| Drugs Used In Diabetes | 1.21 [0.71,2.10] | 0.775 | 1.21 [0.65,2.25] | 0.744 | 1.22 [0.69,2.15] | 0.766 |
| Dapagliflozin | 1.21 [0.84,1.76] | 0.848 | 1.21 [0.75,1.98] | 0.790 | 1.22 [0.84,1.77] | 0.848 |
| Immunostimulants | 1.05 [0.63,1.74] | 0.581 | 1.15 [0.68,1.96] | 0.712 | 1.07 [0.63,1.82] | 0.611 |
| Interferon Beta | 1.05 [0.77,1.43] | 0.616 | 1.15 [0.80,1.68] | 0.770 | 1.07 [0.78,1.46] | 0.666 |
| Immunosuppressants | 1.40 [1.09,1.85] | 0.992 | 1.41 [1.08,1.89] | 0.991 | 1.40 [1.07,1.88] | 0.990 |
| Canakinumab | 1.33 [0.97,1.77] | 0.965 | 1.36 [0.96,1.88] | 0.960 | 1.33 [0.96,1.78] | 0.959 |
| Sarilumab | 1.50 [1.15,2.05] | 0.998 | 1.50 [1.12,2.11] | 0.996 | 1.51 [1.14,2.08] | 0.998 |
| Tocilizumab | 1.35 [1.21,1.49] | 1.000 | 1.36 [1.13,1.64] | 0.999 | 1.34 [1.21,1.49] | 1.000 |
| Tofacitinib | 1.44 [1.04,2.12] | 0.984 | 1.44 [1.02,2.15] | 0.979 | 1.45 [1.03,2.16] | 0.982 |
| Immunosuppressants + Antivirals For Systemic Use | 1.53 [0.88,2.71] | 0.945 | 1.58 [0.84,3.00] | 0.932 | 1.48 [0.83,2.72] | 0.925 |
| Baricitinib + Remdesivir | 1.70 [1.24,2.33] | 0.999 | 1.67 [1.04,2.68] | 0.982 | 1.69 [1.23,2.32] | 0.999 |

OR: odds ratio; CrI: equal-tailed credible interval.

## **Table S16. Sensitivity analysis: fixed-effects model versus random-effects model (viral clearance).**

|  | **Fixed-effects** | | **Random-effects** | |
| --- | --- | --- | --- | --- |
| **Treatment** | **OR [95% CrI]** | **Posterior Probability Favouring Treatment** | **OR [95% CrI]** | **Posterior Probability Favouring Treatment** |
| Anthelmintics | 2.32 [1.06,5.08] | 0.981 | 2.71 [0.89,8.24] | 0.965 |
| Ivermectin | 2.32 [1.38,3.94] | 0.999 | 2.70 [1.24,6.12] | 0.993 |
| Anthelmintics + Antibacterials For Systemic Use | 2.54 [1.14,5.76] | 0.986 | 2.41 [0.67,8.55] | 0.922 |
| Ivermectin + Doxycycline | 2.54 [1.47,4.49] | 1.000 | 2.41 [0.87,6.62] | 0.956 |
| Antineoplastic Agents | 1.03 [0.51,2.24] | 0.537 | 1.06 [0.29,3.90] | 0.536 |
| Bamlanivimab | 0.97 [0.53,1.86] | 0.460 | 1.01 [0.30,3.44] | 0.507 |
| Bamlanivimab + Etesevimab | 1.10 [0.56,2.28] | 0.613 | 1.10 [0.32,3.82] | 0.566 |
| Antiprotozoals | 1.49 [0.96,2.54] | 0.968 | 1.40 [0.73,3.25] | 0.866 |
| Hydroxychloroquine | 1.31 [1.05,1.62] | 0.992 | 1.24 [0.80,1.93] | 0.838 |
| Nitazoxanide | 1.72 [1.20,2.73] | 0.999 | 1.59 [0.83,4.13] | 0.921 |
| Antiprotozoals + Antivirals For Systemic Use | 1.15 [0.49,2.73] | 0.636 | 1.15 [0.25,5.33] | 0.580 |
| Hydroxychloroquine + Favipiravir | 1.15 [0.61,2.17] | 0.669 | 1.15 [0.31,4.33] | 0.590 |
| Antiprotozoals + Antibacterials For Systemic Use | 1.04 [0.48,2.17] | 0.542 | 1.00 [0.31,3.20] | 0.502 |
| Hydroxychloroquine + Azithromycin | 1.03 [0.64,1.66] | 0.553 | 1.00 [0.42,2.41] | 0.501 |
| Antivirals For Systemic Use | 1.24 [0.79,1.93] | 0.851 | 1.26 [0.63,2.52] | 0.771 |
| Favipiravir | 1.30 [0.91,1.88] | 0.923 | 1.33 [0.74,2.44] | 0.837 |
| Lopinavir/Ritonavir | 1.25 [0.84,1.86] | 0.872 | 1.24 [0.65,2.36] | 0.757 |
| Remdesivir | 1.18 [0.73,1.79] | 0.767 | 1.23 [0.55,2.59] | 0.720 |
| Blood Substitutes And Perfusion Solutions | 1.62 [0.83,3.18] | 0.940 | 1.71 [0.62,4.89] | 0.879 |
| Convalescent Plasma | 1.62 [1.18,2.24] | 0.999 | 1.71 [0.87,3.46] | 0.945 |
| Corticosteroids, Dermatological Preparations | 1.02 [0.46,2.27] | 0.522 | 1.02 [0.23,4.56] | 0.512 |
| Methylprednisolone | 1.02 [0.59,1.76] | 0.530 | 1.02 [0.28,3.67] | 0.515 |
| Endocrine Therapy | 10.33 [ 4.32,>10] | 1.000 | 10.30 [ 2.20,>10] | 0.996 |
| Proxalutamide | 10.33 [ 5.45,>10] | 1.000 | 10.33 [ 2.72,>10] | 0.999 |

OR: odds ratio; CrI: equal-tailed credible interval.

# **Supplementary Figures**


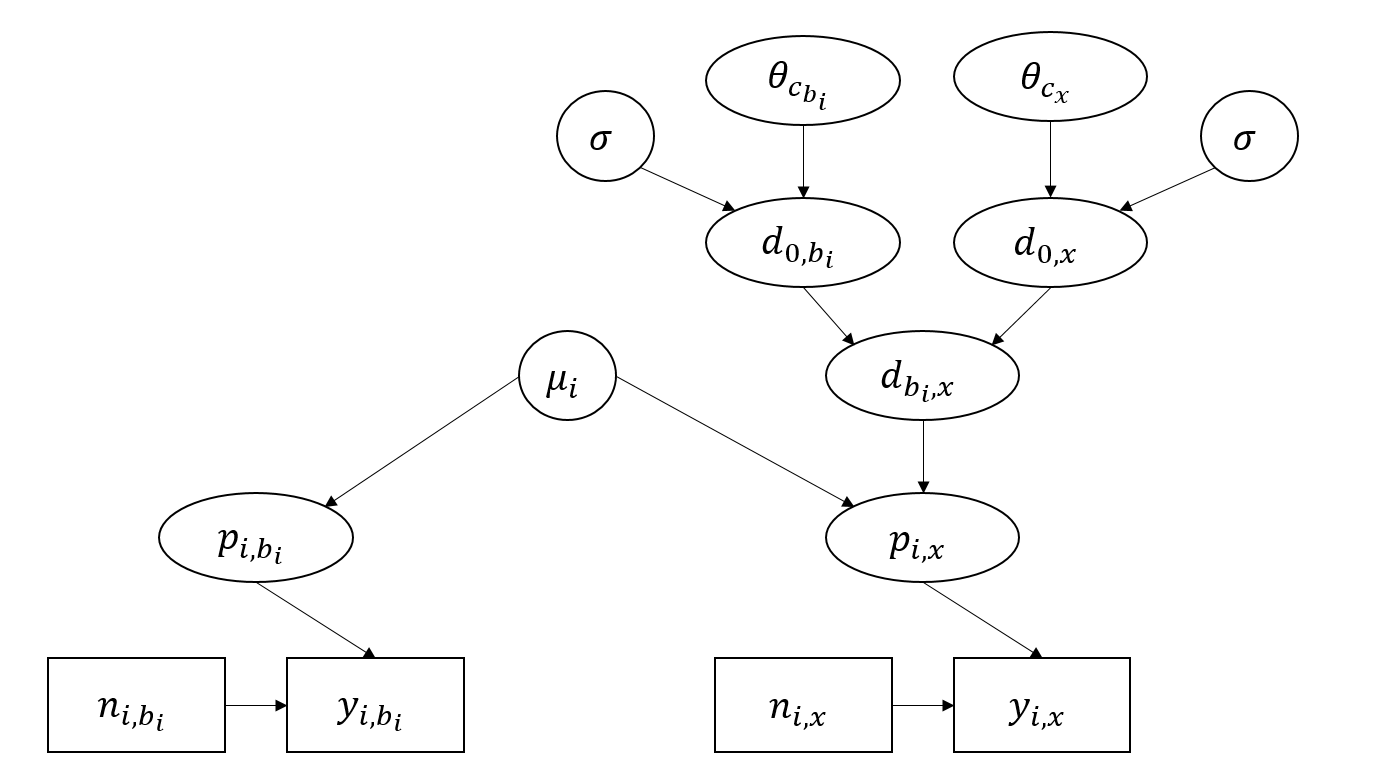


## **Figure S1. Bayesian hierarchical framework for trial in the network meta-analysis,**

where $(\boldsymbol{n}_{\boldsymbol{i},\boldsymbol{x}},\boldsymbol{y}_{\boldsymbol{i},\boldsymbol{x}})$ are the sample size and number of events in for treatment $\boldsymbol{x}$, $\boldsymbol{c}_{\boldsymbol{y}}$ is the class of treatment $\boldsymbol{y}$.


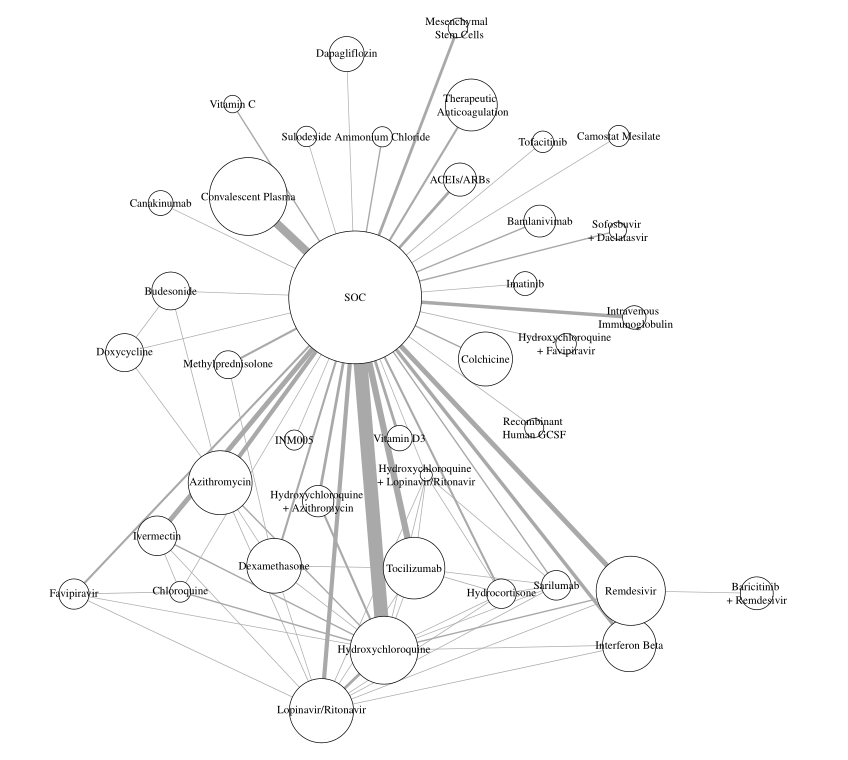


## **Figure S2. Network plot for mortality.**

The width of the lines is proportional to the number of direct comparisons and the size of the node is proportional to the patients included.

**
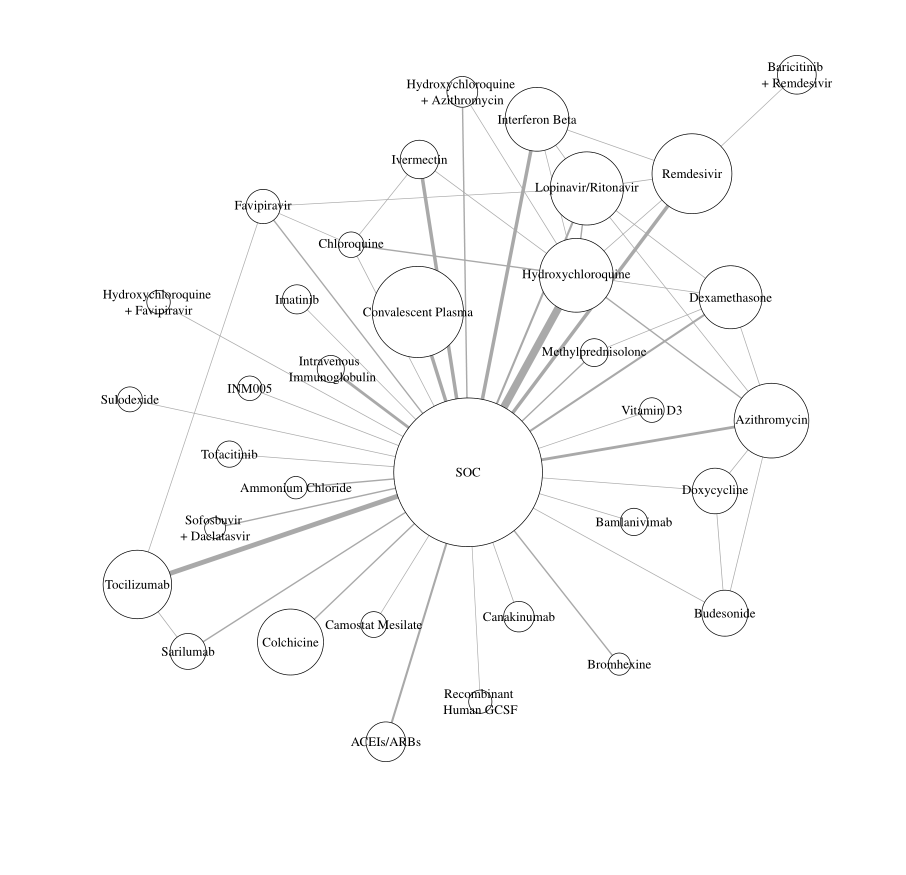
**

## **Figure S3. Network plot for mechanical ventilation.**

The width of the lines is proportional to the number of direct comparisons and the size of the node is proportional to the patients included.


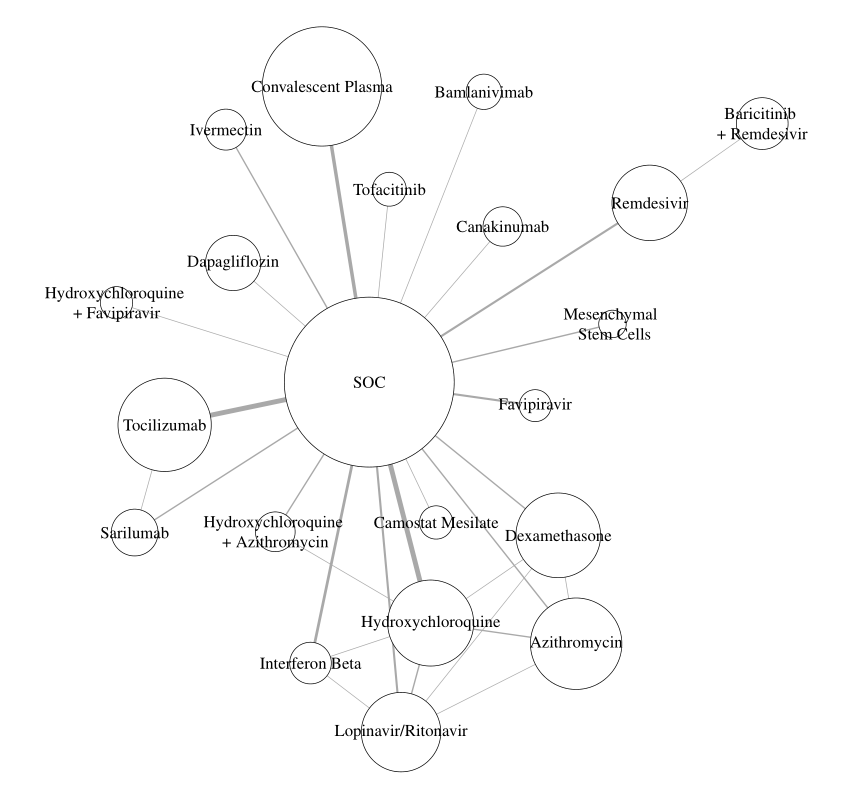


## **Figure S4. Network plot for discharge.**

The width of the lines is proportional to the number of direct comparisons and the size of the node is proportional to the patients included.


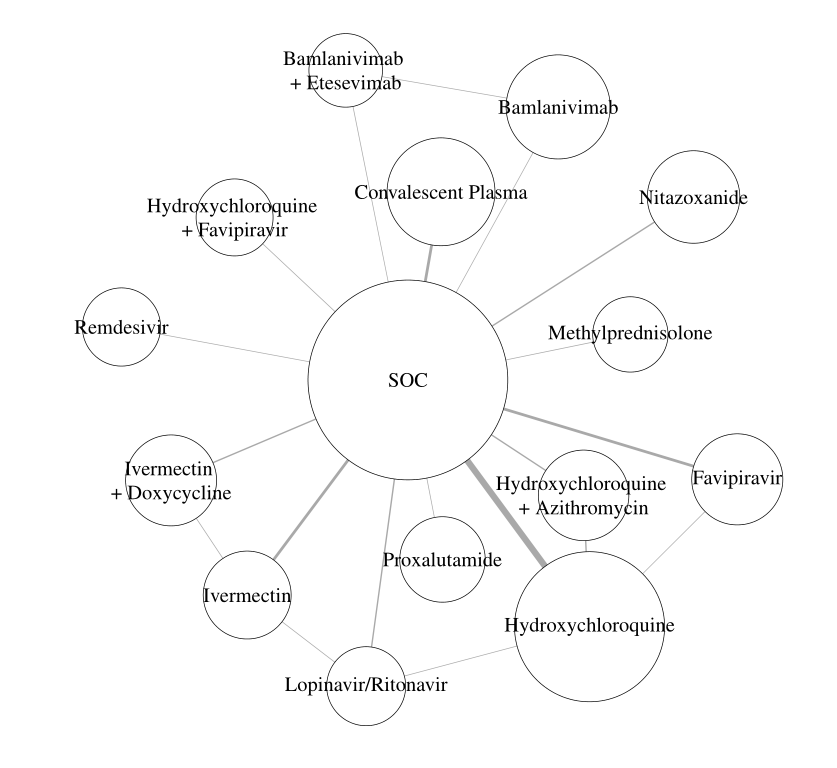


## **Figure S5. Network plot for viral clearance.**

The width of the lines is proportional to the number of direct comparisons and the size of the node is proportional to the patients included.


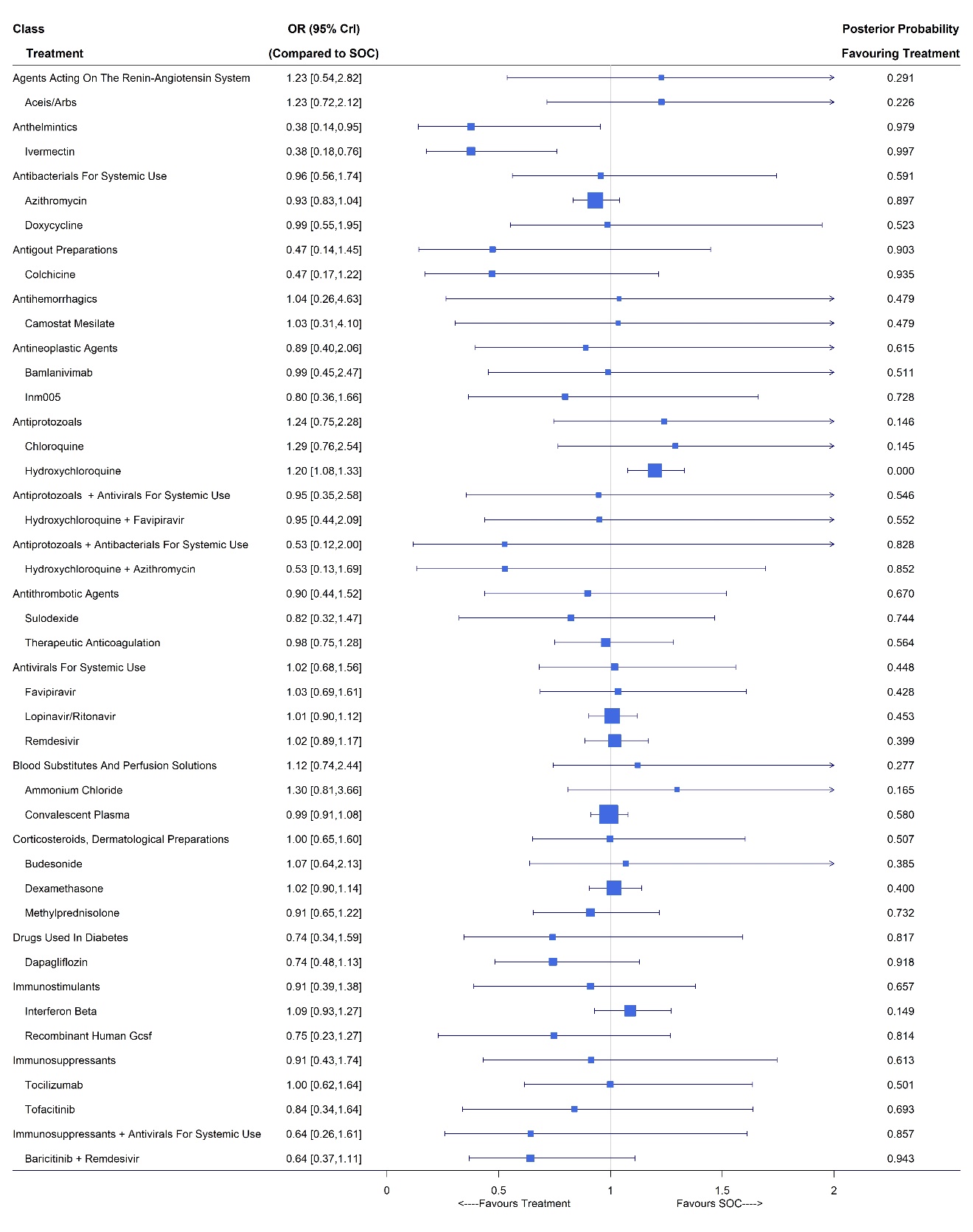


## **Figure S6. Subgroup analysis for mild/moderate COVID-19 patients: mortality under treatments compared with the standard of care (SOC).**

OR is the odds ratio and CrI represents credible interval.


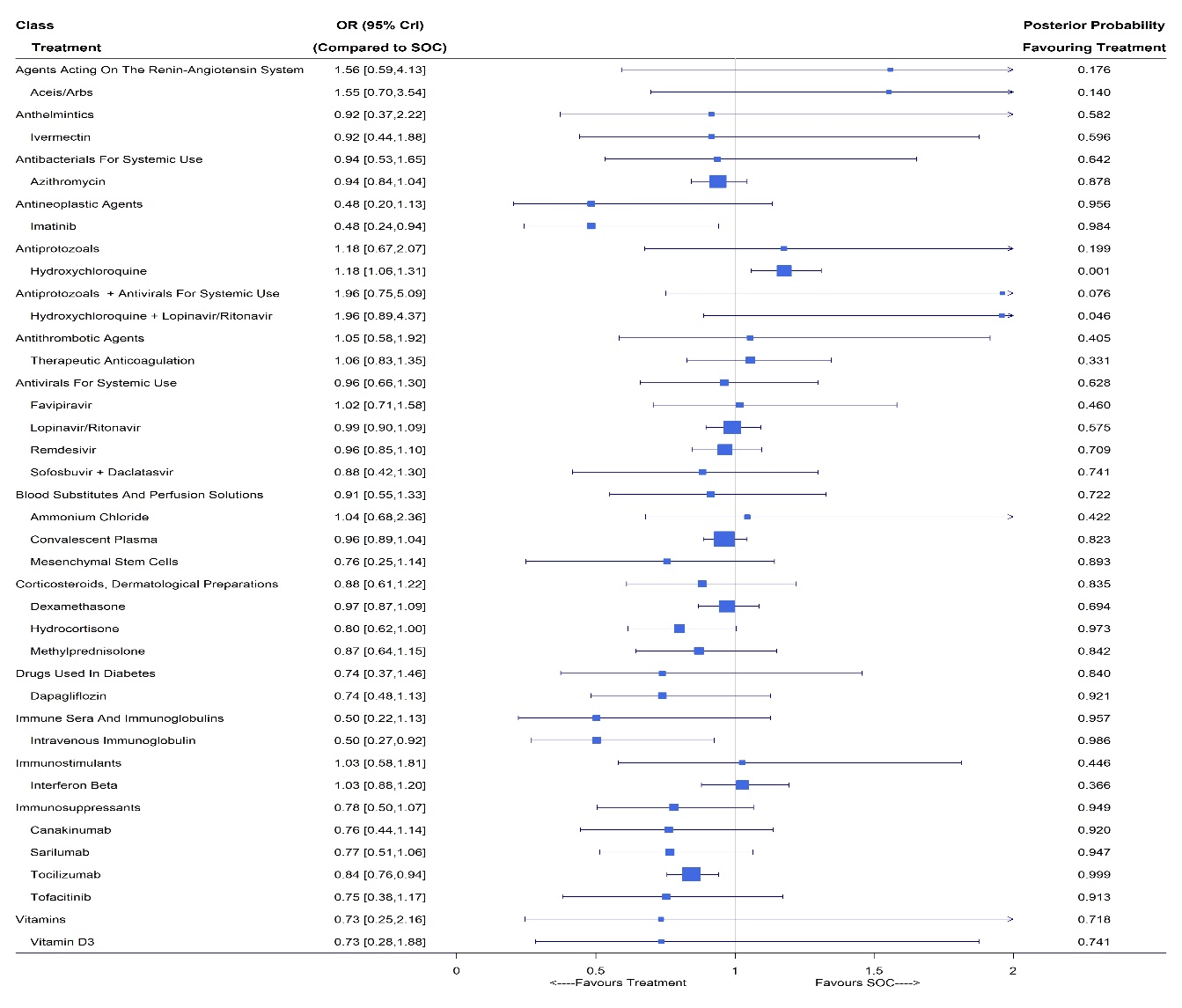


## **Figure S7. Subgroup analysis for severe COVID-19 patients: mortality under treatments compared with the standard of care (SOC).**

OR is the odds ratio and CrI represents credible interval.


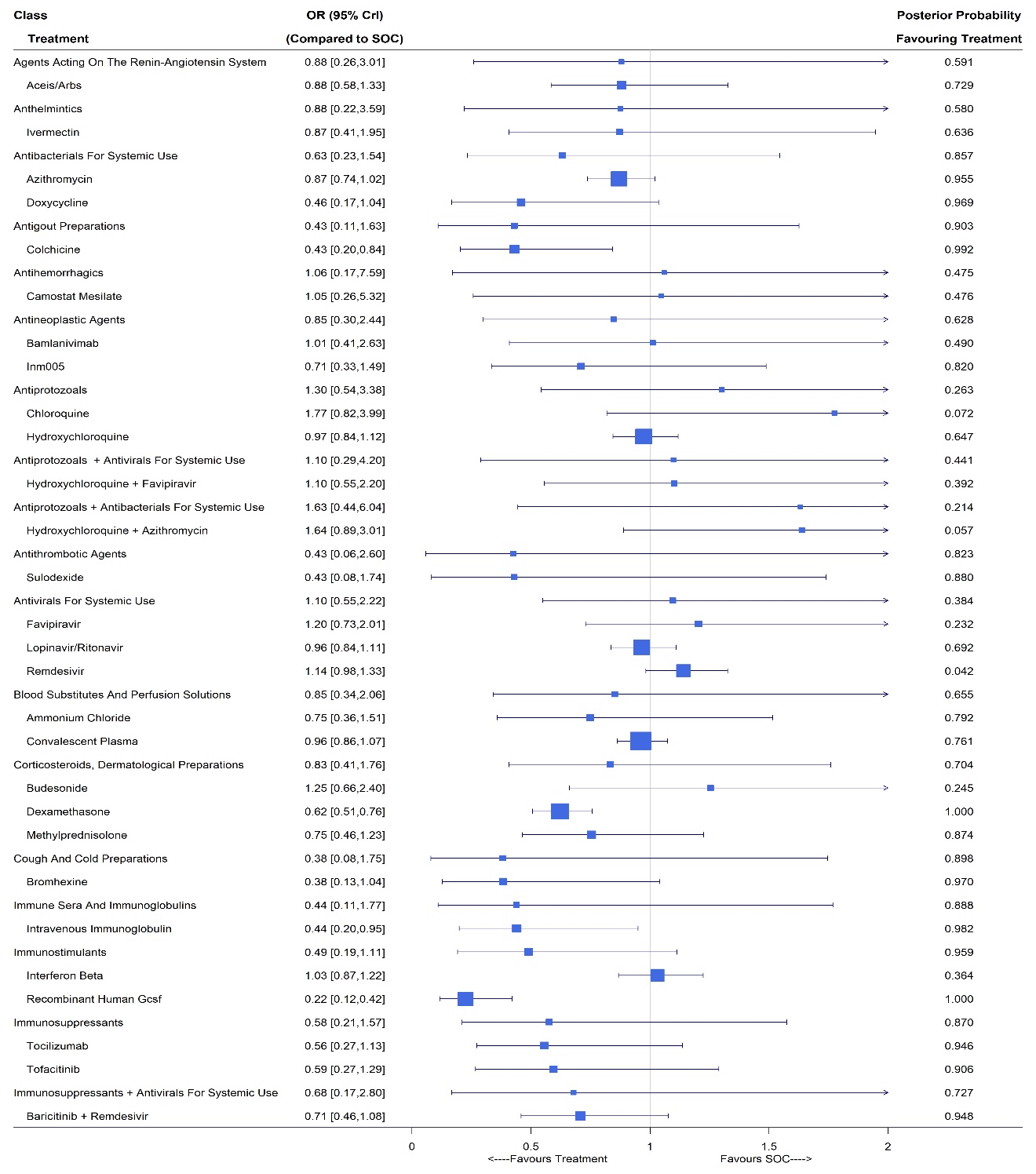


## **Figure S8. Subgroup analysis for mild/moderate COVID-19 patients: mechanical ventilation under treatments compared with the standard of care (SOC).**

OR is the odds ratio and CrI represents credible interval.


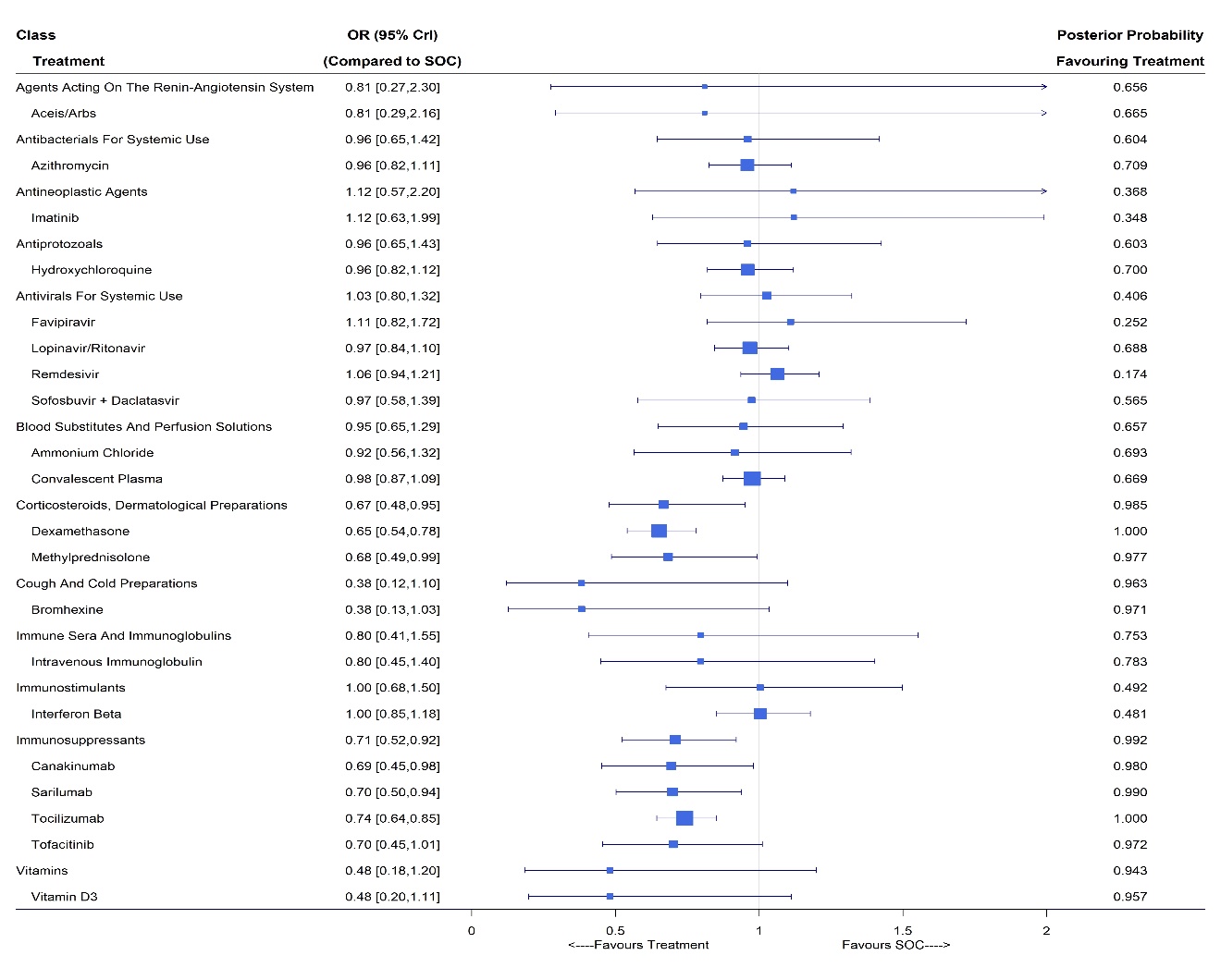


## **Figure S9. Subgroup analysis for severe COVID-19 patients: mechanical ventilation under treatments compared with the standard of care (SOC).**

OR is the odds ratio and CrI represents credible interval.


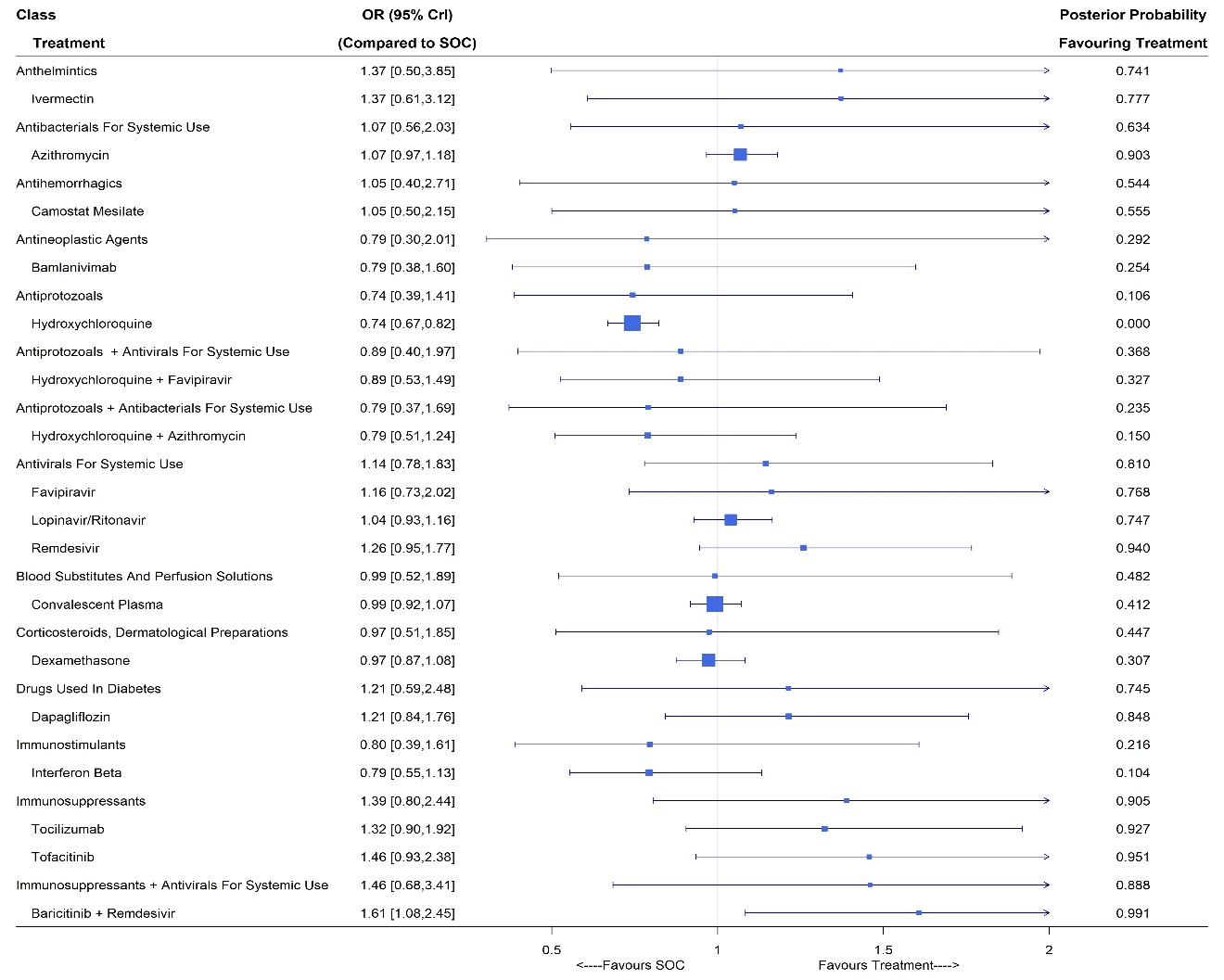


## **Figure S10. Subgroup analysis for mild/moderate COVID-19 patients: discharge under treatments compared with the standard of care (SOC).**

OR is the odds ratio and CrI represents credible interval.


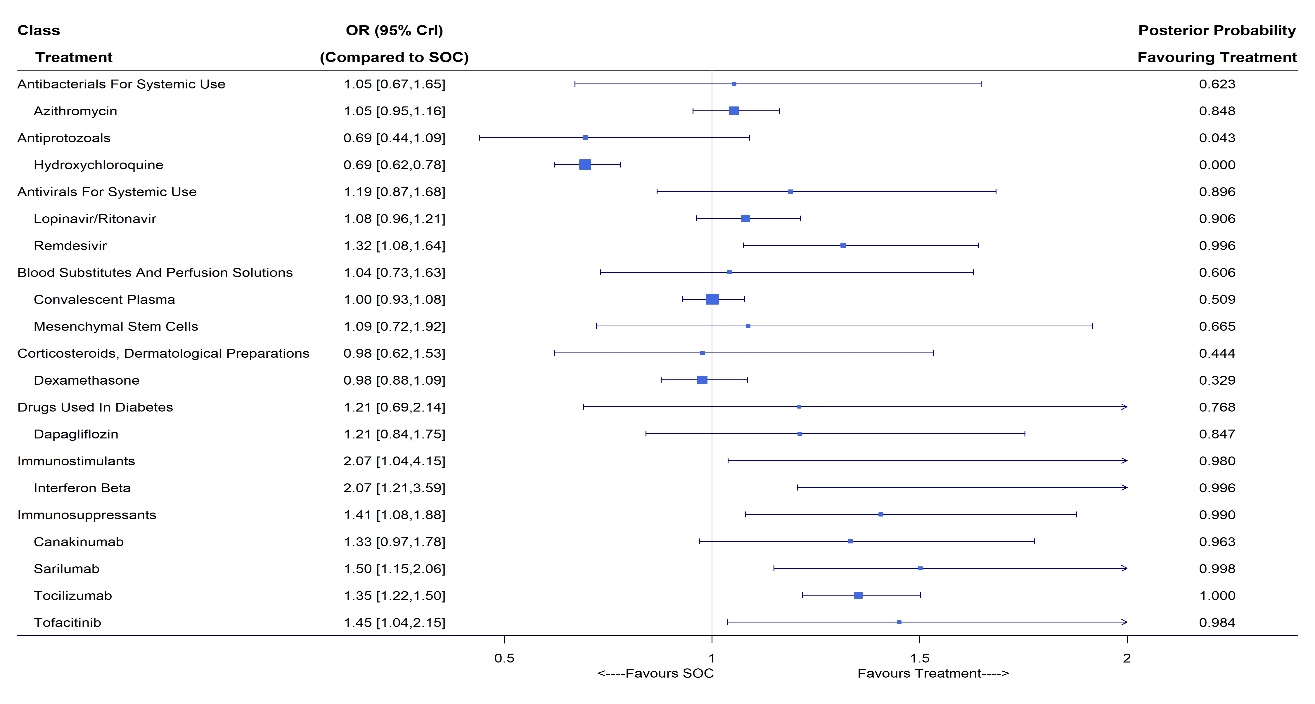


## **Figure S11. Subgroup analysis for severe COVID-19 patients: discharge under treatments compared with the standard of care (SOC).**

OR is the odds ratio and CrI represents credible interval.


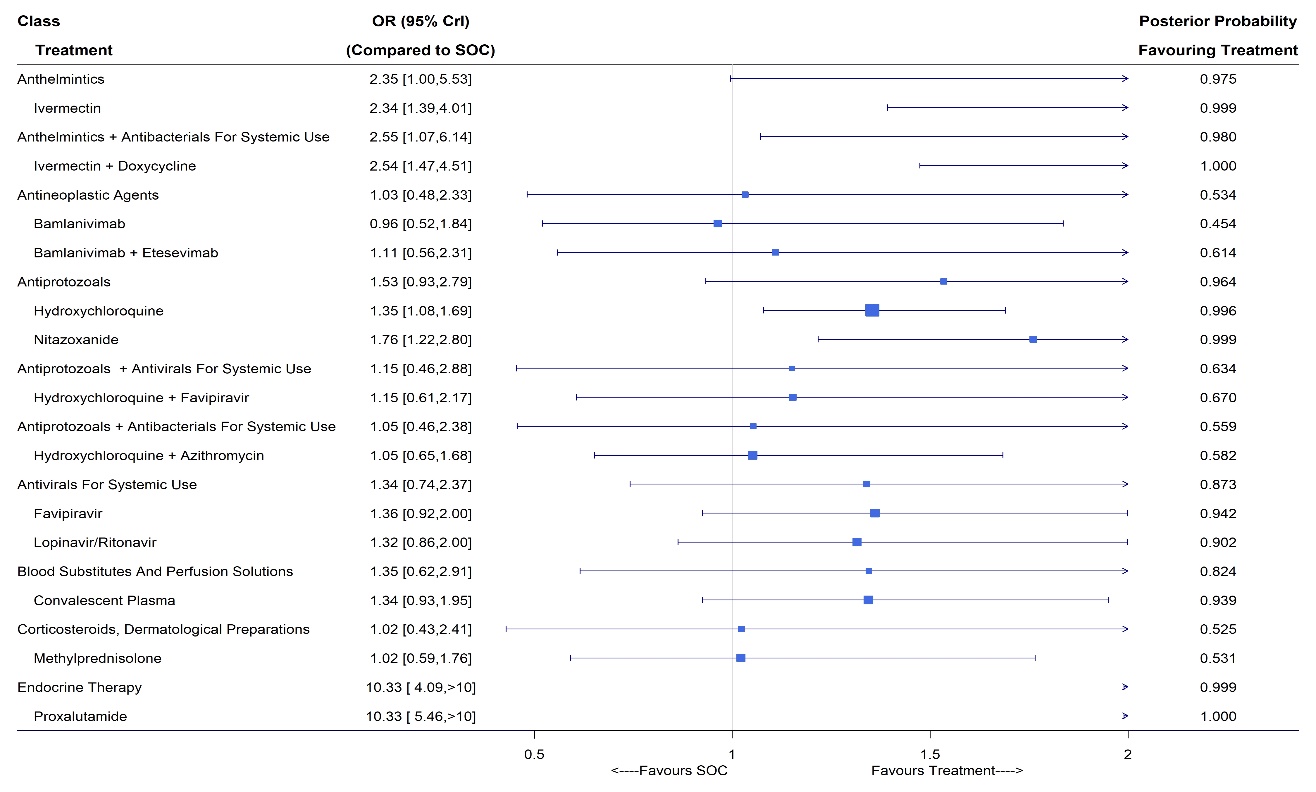


## **Figure S12. Subgroup analysis for mild/moderate COVID-19 patients: viral clearance under treatments compared with the standard of care (SOC).**

OR is the odds ratio and CrI represents credible interval.


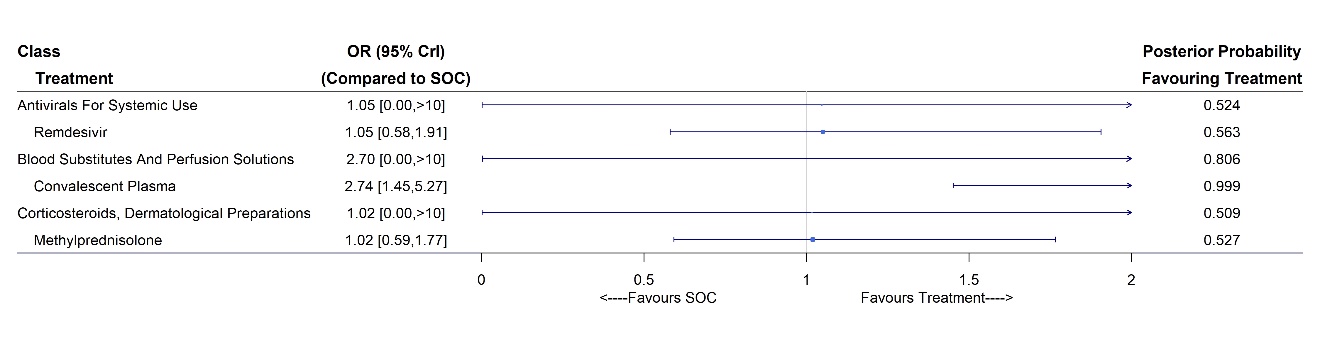


## **Figure S13. Subgroup analysis for severe COVID-19 patients: viral clearance under treatments compared with the standard of care (SOC).**

OR is the odds ratio and CrI represents credible interval.


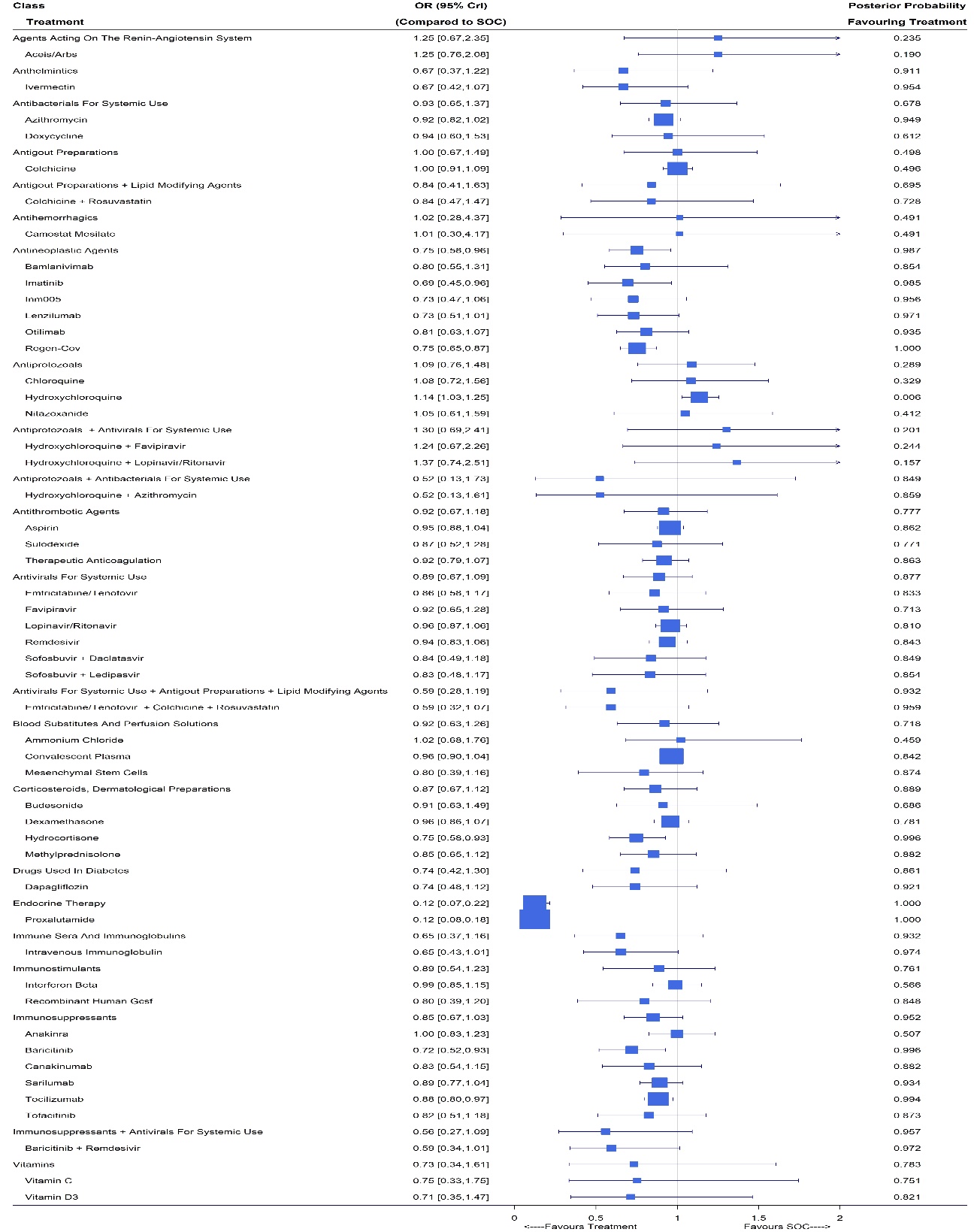


## **Figure S14. Exploratory analysis for both peer-reviewed and preprint studies: mortality under treatments compared with the standard of care (SOC).**

OR is the odds ratio and CrI represents credible interval.


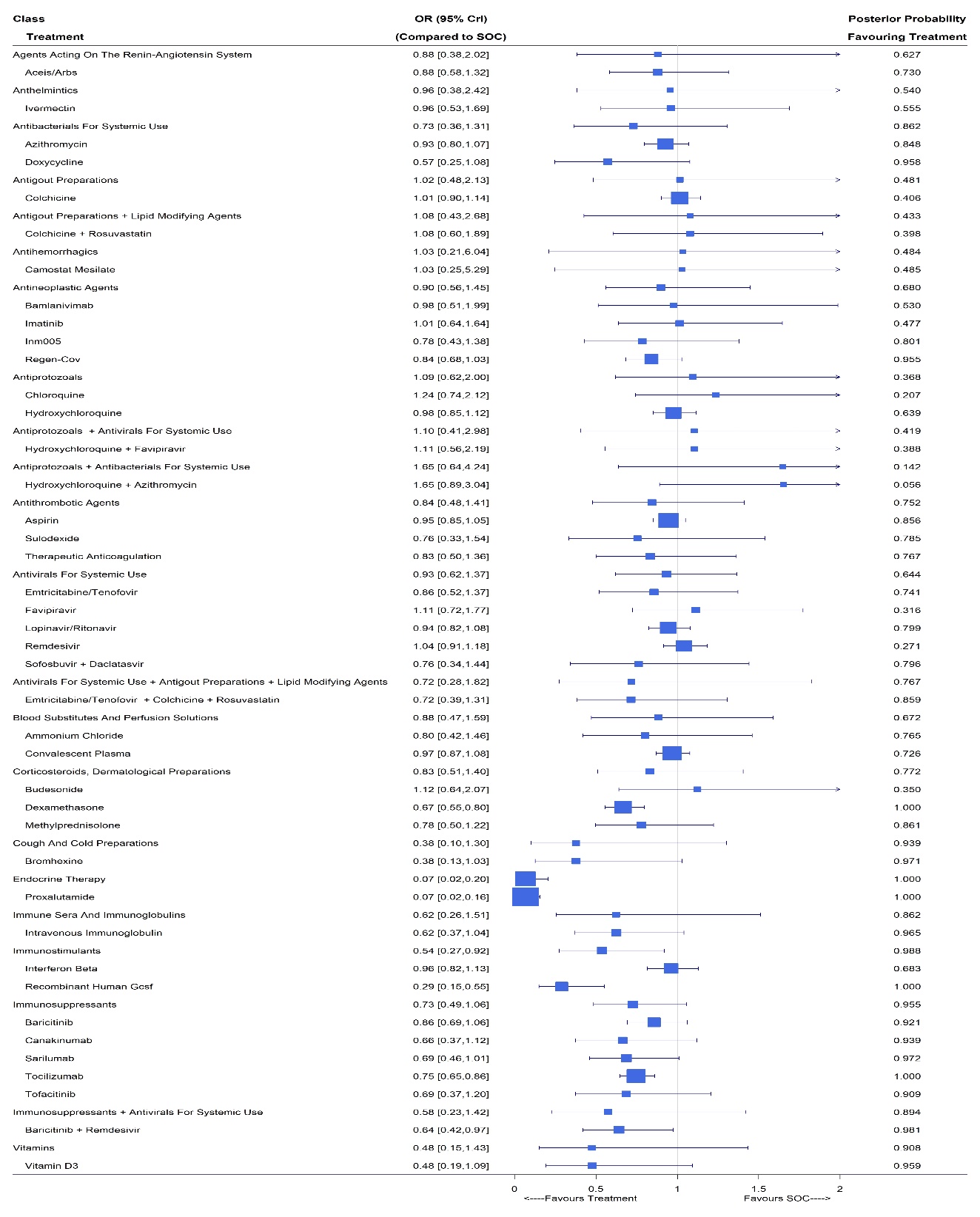


## **Figure S15. Exploratory analysis for both peer-reviewed and preprint studies: mechanical ventilation under treatments compared with the standard of care (SOC).**

OR is the odds ratio and CrI represents credible interval.


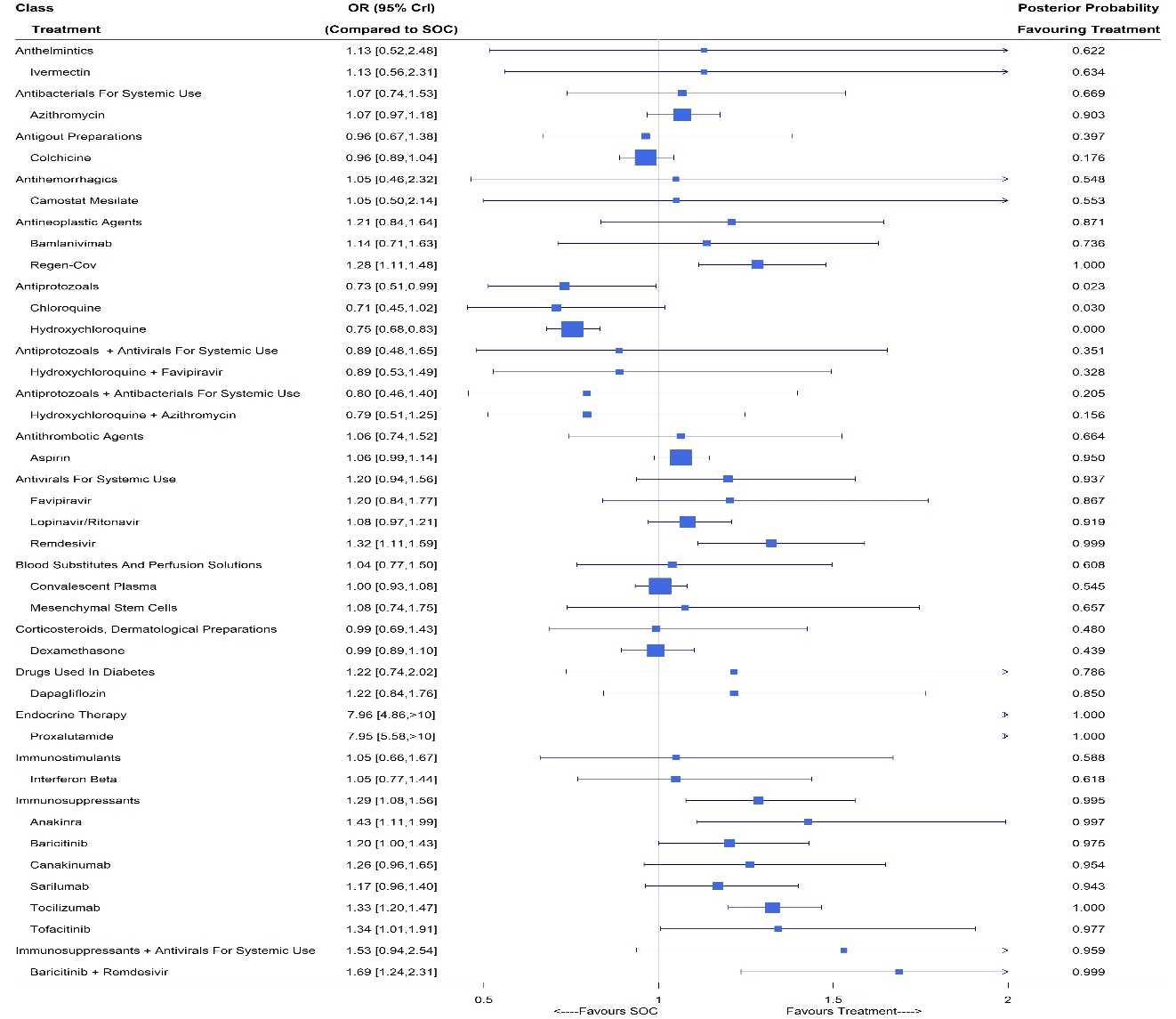


## **Figure S16. Exploratory analysis for both peer-reviewed and preprint studies: discharge under treatments compared with the standard of care (SOC).**

OR is the odds ratio and CrI represents credible interval.


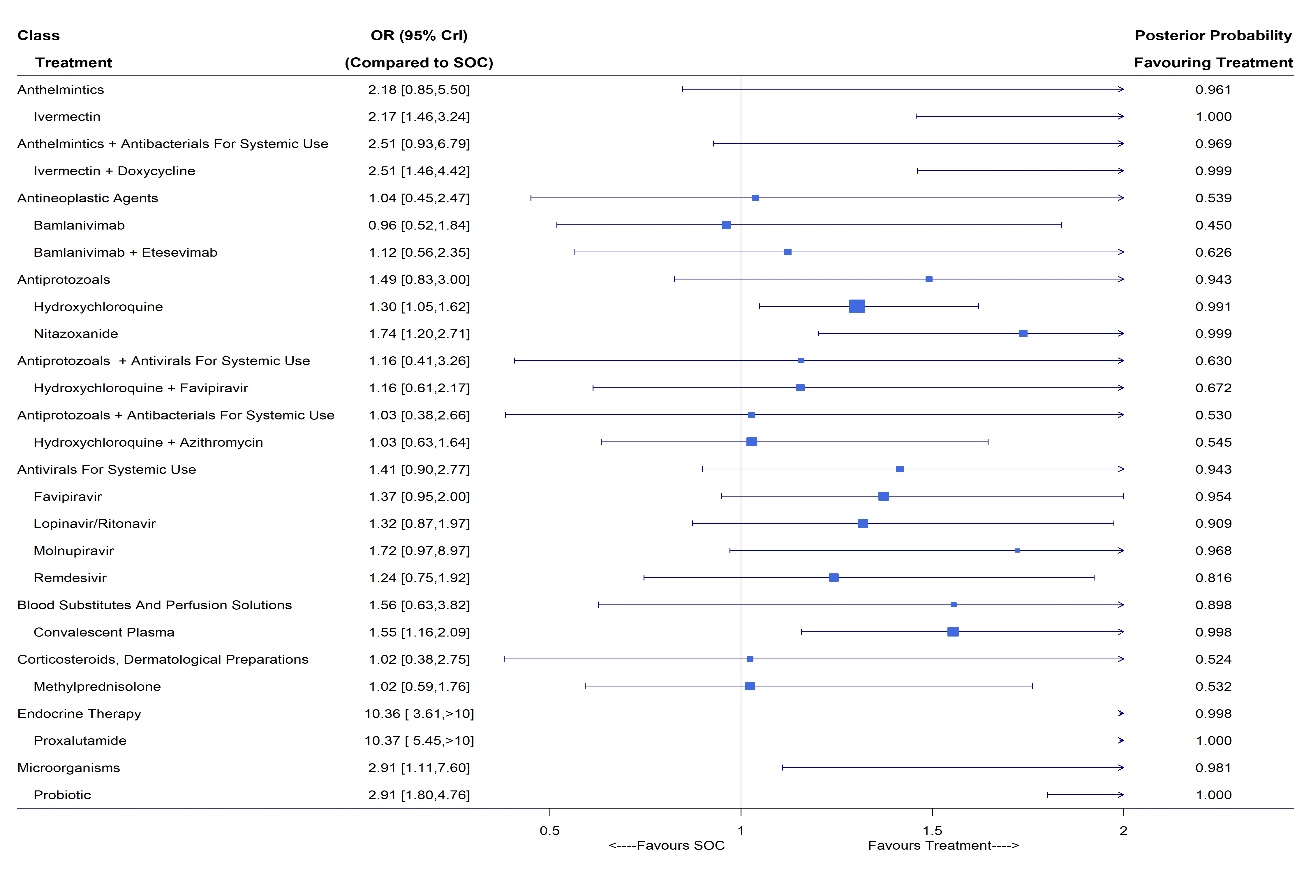


## **Figure S17. Exploratory analysis for both peer-reviewed and preprint studies: viral clearance under treatments compared with the standard of care (SOC).**

OR is the odds ratio and CrI represents credible interval.
